# Coffee consumption and associations with blood pressure, LDL-cholesterol and echocardiographic measures in the general population

**DOI:** 10.1038/s41598-023-31857-5

**Published:** 2023-03-22

**Authors:** Juliana Senftinger, Julius Nikorowitsch, Katrin Borof, Francisco Ojeda, Ghazal Aarabi, Thomas Beikler, Carola Mayer, Christian-Alexander Behrendt, Carolin Walther, Birgit-Christiane Zyriax, Raphael Twerenbold, Stefan Blankenberg, Jan-Per Wenzel

**Affiliations:** 1 Department of Cardiology, University Heart and Vascular Center Hamburg, University Medical Center Hamburg-Eppendorf, Hamburg, Germany; 2 Epidemiological Study Center, Hamburg, Germany; 3 University Center of Cardiovascular Science, University Heart and Vascular Center Hamburg, University Medical Center Hamburg-Eppendorf, Hamburg, Germany; 4 German Center for Cardiovascular Research (DZHK), Partner Site Hamburg/Kiel/Lübeck, Hamburg, Germany; 5 Department of Periodontics, Preventive and Restorative Dentistry, Hamburg, Germany; 6 Department of Neurology, University Medical Center Hamburg-Eppendorf, Hamburg, Germany; 7 Midwifery Science – Health Care Research and Prevention, Institute for Health Services in Dermatology and Nursing (IVDP), Hamburg, Germany; 8 Department of Vascular Medicine, German Aortic Center Hamburg, University Heart and Vascular Center Hamburg, University Medical Center Hamburg-Eppendorf, Hamburg, Germany

**Keywords:** Lifestyle modification, Dyslipidaemias, Heart failure, Hypertension, Vascular diseases

## Abstract

Coffee, next to water the most widespread beverage, is attributed both harmful and protective characteristics concerning cardiovascular health. This study aimed to evaluate associations of coffee consumption with cardiac biomarkers, echocardiographic, electrocardiographic parameters and major cardiovascular diseases. We performed a cross-sectional analysis of 9009 participants of the population-based Hamburg City Health Study (HCHS), enrolled between 2016 and 2018 median age 63 [IQR: 55; 69] years. Coffee consumption was classified into three groups: < 3 cups/day (low), 3–4 cups/day (moderate), > 4 cups/day (high). In linear regression analyses adjusted for age, sex, body mass index, diabetes, hypertension, smoking, and additives, high coffee consumption correlated with higher LDL-cholesterol (β = 5.92; 95% CI 2.95, 8.89; p < 0.001). Moderate and high coffee consumption correlated with lower systolic (β = − 1.91; 95% CI − 3.04, − 0.78; p = 0.001; high: β = − 3.06; 95% CI − 4.69, − 1.44; p < 0.001) and diastolic blood pressure (β = − 1.05; 95% CI − 1.67, − 0.43; p = 0.001; high: β = − 1.85; 95% CI − 2.74, − 0.96; p < 0.001). Different levels of coffee consumption did neither correlate with any investigated electrocardiographic or echocardiographic parameter nor with prevalent major cardiovascular diseases, including prior myocardial infarction and heart failure. In this cross-sectional analysis, high coffee consumption correlated with raised LDL-cholesterol levels and lower systolic and diastolic blood pressure. However, major cardiovascular diseases including heart failure and its diagnostic precursors were not associated with coffee consumption, connoting a neutral role of coffee in the context of cardiovascular health.

## Introduction

Coffee is one of the most widely consumed beverages around the world. Ever since consumption of coffee became vastly popular, the interest of its implications on health, and specifically the cardiovascular system, grew. First studies on coffee consumption and the risk of coronary artery disease (CAD) were already conducted in the 1960s leading to conflicting results^1^. Many studies have been published, attributing both protective and harmful characteristics to coffee in the context of the cardiovascular system^2–5^. Coffee is a complex liquid consisting of more than 1000 bioactive substances^6^. Most commonly, caffeine is regarded as the main driving component of mediating cardiovascular effects. Nevertheless, narrowing it down to a certain substance oversimplifies the versatile composition of coffee. Looking at coffee as a whole, several studies postulated a dose-dependent relationship of coffee consumption and cardiovascular diseases, e.g. low to moderate coffee consumption was shown to be associated with a reduced risk of heart failure whereas high coffee consumption reversed this trend^7–9^. However, an in-depth analysis of coffee consumption and its associations with cardiovascular diseases, especially heart failure and its possible precursors is lacking. Only few studies have evaluated the associations of coffee with cardiac functional parameters measured by echocardiography or electrocardiography^10,11^.

Trying to fill this gap, in the present study we analyze the association of coffee consumption and the cardiovascular system as a whole, integrating lifestyle-related behaviour, comorbidities, biomarkers, electrocardiographic and echocardiographic data, and finally major cardiovascular diseases in a large sample of the general population.

## Methods

### Study setting

**Figure 1:**
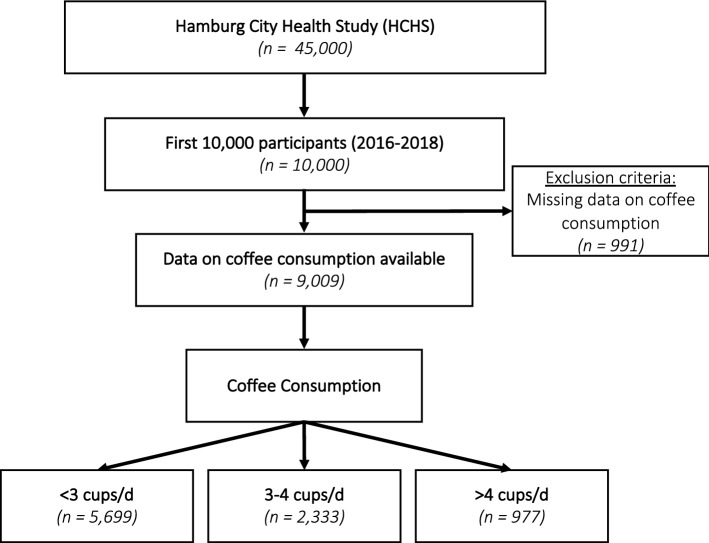
Study PRISMA. From a total of 10,000 subjects 9009 provided data on coffee consumption. The 9009 subjects were then stratified by their coffee consumption measured as cups per day*.*

Data from the first 10,000 participants from the Hamburg City Health Study (HCHS, www.hchs.hamburg) served as the base for this analysis. The HCHS (clinicaltrials.gov: NCT03934957), located in Hamburg, Germany, is an ongoing, prospective, single-centre, long-term, and randomly selected population-based cohort study which aims at investigating the interactions of socioeconomic risk factors, modern imaging techniques, physiological measurements, and clinical variables^12^. Our study population included a subset of the first 10,000 HCHS participants. Subjects with missing data on coffee consumption were excluded. Our final cohort comprised 9009 subjects (Fig. 1).

The research protocol of the study was approved by the HCHS steering board and the local ethics committee (PV5131, State of Hamburg Chamber of Medical Practitioners). All participants gave a written informed consent. The investigation conforms with the principles outlined in the Declaration of Helsinki.

### Laboratory, clinical and questionnaire data

All measurements were conducted between 2016 and 2018 at a baseline visit at the HCHS Epidemiological Study Centre Hamburg-Eppendorf, Hamburg, following the published HCHS protocol^12^. Cholesterol levels were directly measured in blood samples drawn at the day of examination under fasting conditions. N-terminal pro-B-type natriuretic peptide (NT-proBNP) was measured in serum samples drawn at the day of examination and stored at − 80 °C in a dedicated blood biobank (immunoassay by Alere NT-proBNP for ARCHITECT, Abbott Diagnostics, measurement ranges between 8.2 and 35,000 ng/l). A digital 12-lead electrocardiogram (ECG) combined with a 2-min rhythm strip was acquired from each participant. The durations of wave intervals were calculated electronically and double-checked manually. Further ECG analysis, i.e., on conduction disturbances and underlying rhythm, was conducted by a trained physician. Arterial hypertension was defined as a systolic blood pressure > 140 mmHg, a diastolic blood pressure > 90 mmHg, or the use of antihypertensive drugs. For the assessment of medication, subjects were asked to bring their medication or a list of prescribed drugs to the day of their baseline visit. Before, during and after the baseline visit extensive self-completion questionnaires concerning nutrition, lifestyle, medical, and family history as well as health care research patterns, occupational history and environmental data were completed. Information on dietary intake was collected by validated questionnaires developed for the European Prospective Investigation into Cancer and Nutrition (EPIC) study. The participants were asked how many cups of coffee they drink regarding the last 12 months (1 cup equals 150 ml). Coffee consumption was categorized in the following categories: never, 1 per month, 2 per month, 1 per week, 4–6 per week, 1–2 per day, 3–4 per day, 5–6 per day, 7–8 per day, 9–10 per day, 11 or more per day. We then summarized the categories into 3 groups: < 3 cups/day, 3–4 cups/day, > 4 cups/day. Further questions concerning coffee consumption included additives like milk, sugar, and honey.

Medical history, smoking status, tea, and carbonated drinks consumption were detected by standardized, self-reported questionnaires. Atrial fibrillation was considered present if reported by questionnaire or 12-lead electrocardiogram or both. Diabetes mellitus was determined by a fasting glucose level of ≥ 126 mg/dl, or the use of antidiabetic drugs. CAD was defined as suffering from one or more of the following conditions: history of myocardial infarction, percutaneous coronary intervention (PCI) or coronary bypass surgery. The dichotomized variable PAD was derived from structured anamnesis data, self-based questionnaire, and baseline examination. All participants were asked if they had experienced any history of intermittent claudication, ischemic rest pain, or ischemic wound healing disorders. At the study center, the ankle-brachial-index (ABI) was measured in both legs and cut off for diagnosis were values < 0.9.

### Transthoracic echocardiography

Transthoracic echocardiogram (TTE) examinations were systematically performed at the baseline visit using state-of-the-art cardiac ultrasound equipment (Siemens Acuson SC2000 Prime, Siemens Healthineers, Erlangen, Germany). Images were acquired and analysed by trained and internally certified medical professionals (cardiologists, sonographers) as previously published by our group. For continuous quality assessment, every 100th TTE exam was analysed twice. Left sided volumes and ejection fraction (LVEF) were calculated from the apical four- and two-chamber view using the method of disks summation. Left-sided diameters were measured in parasternal long-axis view. Mitral inflow pattern was assessed in apical four-chamber view by placing pulsed-wave (PW) Doppler sample volume between mitral leaflet tips. PW tissue Doppler imaging (TDI) e’ velocity was measured in apical four-chamber view by placing the sample volume at the lateral and septal basal regions. Tricuspid annular plane systolic excursion (TAPSE) was obtained by M-mode echocardiography in the apical four-chamber view.

### Definition of heart failure

For the classification of subjects Heart Failure (HF) the 2021 ESC Guidelines for the diagnosis and treatment of acute and chronic HF were applied and modified^13^. HF was classified in two groups: heart failure with mildly-reduced and reduced ejection fraction (HF(m)rEF) as well as heart failure with preserved ejection fraction (HFpEF). Subjects had to show a combination of signs/symptoms, laboratory data, and echocardiographic criteria. Self-reported history of HF and/or the following medication were seen as equivalent if no symptoms or signs were detectable: betablockers, ACE-inhibitors (ACEi), angiotensin receptor blockers (ARB), angiotensin receptor neprilysin inhibitors (ARNI) mineralocorticoid receptor antagonists (MRA), Sodium-glucose Cotransporter-2 (SGLT2) inhibitors, and loop diuretics for HF(m)rEF and only loop diuretics for HFpEF. Oedema were evaluated by physical examination by medical professionals. Dyspnoea, history of HF, and medication were assessed by standardized self-reported questionnaires. All subjects with LVEF < 50% and symptoms or signs of HF were classified as HF with reduced and mildly reduced ejection fraction (HF(m)rEF), instead of differing between heart failure with mildly reduced ejection fraction (HFmrEF, LVEF 41–49%) and heart failure with reduced ejection fraction (HFrEF, LVEF < 40%). Subjects were classified in the HFpEF group if they showed LVEF ≥ 50%, symptoms or signs of HF, and either at least two or more echocardiographic signs of cardiac structural of functional abnormalities or the combination of NT-proBNP levels exceeding 125 ng/l (sinus rhythm) or 365 ng/l (atrial fibrillation) and at least one or more echocardiographic signs of cardiac structural of functional abnormalities. Echocardiographic signs of cardiac structural or functional abnormalities were defined as: left ventricular hypertrophy: LV mass indexed to BSA ≥ 95 g/m^2^ for women, ≥ 115 g/m^2^ for men, left atrial enlargement: left atrial volume index (LAVI) > 34 ml/m^2^ (sinus rhythm) and > 40 ml/m^2^ (atrial fibrillation), E/e’ ratio > 9, and tricuspid regurgitation velocity (Vmax) > 2.8 m/s. HF in general describes all subjects with either HF(m)rEF or HFpEF.

### Statistical analyses

Continuous variables are presented as median and interquartile range, and categorical variables are presented as absolute numbers and percentages. Comparisons between the different coffee groups were performed using Kruskal–Wallis test or chi-squared test. For the analysis of the association between coffee consumption and continuous laboratory, echocardiographic, electrocardiographic outcome parameters as well as blood pressure we used multivariable linear regression models. Adjustment was performed for age, sex, BMI, diabetes, arterial hypertension, and smoking. For systolic and diastolic blood pressure, in line with Tobin et al.^14^, no adjustment for arterial hypertension was performed, instead values for treated individuals were imputed by adding 15 mmHg and 10 mmHg respectively to the measured blood pressure. Furthermore, logistic models were used for binary echocardiographic and electrocardiographic parameters as well as cardiovascular diseases. No correction for multiple testing was applied^15^. A p-value of < 0.05 was considered as statistically significant. All tests were two tailed. Data analysis was performed using R version 3.5.1.

## Results

### Study population

The included 9009 subjects (Fig. 1) of the first 10,000 HCHS participants showed the expected characteristics of a middle-aged European population. 4610 (51.2%) were women with a median age of 63 [IQR: 55; 69] years, and a median BMI of 26.01 [IQR: 23.5; 29.1] kg/m^2^.

Arterial hypertension was present in 5637 (62.6%) subjects, diabetes in 694 (7.7%) subjects. 1731 (19.3%) subjects were smokers. The median LVEF was 58.5% [IQR: 55.5, 61.8]. 8552 (94.9%) subjects consumed coffee. Of those, 5699 (63.3%) subjects consumed less than three cups of coffee per day (low), 2333 (25.9%) 3–4 cups per day (moderate) and 977 (10.8%) more than four cups per day (high). With a rising amount of coffee consumption, subjects were more likely to be men, younger, smokers, and showed higher LDL-levels and BMIs compared to those with lower coffee consumption. Moderate coffee consumers demonstrated the lowest prevalence of diabetes while no relevant interclass differences were observed for prior myocardial infarction, prevalent coronary artery disease (CAD), and peripheral artery disease (PAD) (Table 1).

**Table 1:** Baseline characteristics of the study population.

	Coffee consumption				
	Overall	Low < 3 cups/day	Moderate 3–4 cups/day	High > 4 cups/day	p-value
N (%)	9009	5699	2333	977	
Demographics + biological data					
Age	63.0 {55.0, 69.0}	64.0 {57.0, 70.0}	60.0 {54.0, 67.0}	59.0 {53.0, 66.0}	< 0.001
Female	4610 {51.2}	3099 {54.4}	1148 {49.2}	363 {37.2}	< 0.001
BMI kg/m^2^	26.1 {23.5, 29.1}	26.0 {23.4, 29.1}	26.0 {23.5, 29.1}	26.7 {24.7, 29.5}	< 0.001
Obesity (BMI > 30 kg/m^2^)	1694 (19.9)	1047 (19.4)	445 (20.1)	202 (22.0)	0.193
Smoking current	1731 {19.3}	827 {14.6}	558 {24.0}	346 {35.5}	< 0.001
Cardiovascular diseases					
Arterial hypertension	5637 {65.6}	3732 {68.3}	1346 {61.0}	559 {60.8}	< 0.001
Diabetes mellitus	694 {8.4}	478 {9.1}	146 {6.8}	70 {7.8}	0.006
Heart failure		203 {5.1}	59 {3.6}	31 {4.5}	0.047
Myocardial infarction	266 {3.0}	176 {3.1}	56 {2.4}	34 {3.5}	0.147
CAD	582 {8.7}	395 {9,4}	125 {7.1}	62 {8.4}	0.016
PAD	827 {91.7}	528 {20.0}	213 {19.3}	86 {19.1}	0.833
Laboratories					
Cholesterol, mg/dl	208.0 {181.0, 237.0}	208.0 {181.0, 237.0}	208.0 {182.0, 237.0}	207.0 {182.0, 237.0}	0.947
LDL, mg/dl	121.0 {96.0, 146.0}	119.0 {95.0, 144.0}	122.0 {97.0, 146.0}	124.0 {100.5, 149.0}	< 0.001
HDL, mg/dl	62.0 {50.0, 76.0}	63.0 {51.0, 77.0}	63.0 {50.0, 76.0}	57.0 {47.0, 70.0}	< 0.001
NT-proBNP, ng/l	80.0 {44.0, 145.0}	88.0 {49.0, 159.0}	70.0 {38.0, 126.0}	62.0 {34.0, 116.0}	< 0.001
Hemoglobin, g/dl	14.3 {13.6, 15.1}	14.3 {13.5, 15.1}	14.3 {13.6, 15.1}	14.6 {13.9, 15.3}	< 0.001
Medication					
ACEi/ARBs	1820 {21.2}	1207 {22.1}	434 {19.6}	179 {19.4}	0.023
Beta blockers	1475 {17.5}	1034 {18.9}	316 {14.3}	125 {13.5}	< 0.001
Diuretics	173 {2.0}	122 {2.2}	35 {1.6}	16 {1.7}	0.153
Lipid lowering drugs	1542 {17.9}	1059 {19.4}	340 {15.4}	143 {15.5.}	< 0.001
Additives					
Milk	5966 {69.8}	3803 {72.4}	1549 {66.7}	614 {63.0}	< 0.001
Sugar	1126 {13.2}	738 {14.0}	281 {12.1}	107 {11.0}	0.007
Honey	76 {0.9}	60 {1.1}	15 {0.6}	1 {0.1}	0.002
Sweetener	419 {4.9}	261 {5.0}	104 {4.5}	54 {5.5}	0.411
No additives	2842 {33.2}	1566 {29.8}	855 {36.8}	421 {43.2}	< 0.001
Black/green tea					
Never	1480 {16.6}	872 {15.4}	423 {18.3}	185 {19.1}	< 0.001
1–3/week	4150 {46.4}	2326 {41.1}	1261 {54.5}	563 {58.0}	< 0.001
≥ 4/week	3311 {37.0}	2457 {43.4}	631 {27.3}	223 {23.0}	< 0.001
Carbonated drinks					
Never	4338 {48.5}	2886 {51.0}	1060 {45.7}	392 {40.6}	< 0.001
1–3/week	4031 {45.1}	2439 {43.1}	1098 {47.3}	494 {51.1}	< 0.001
≥ 4/week	576 {6.4}	333 {5.9}	163 {7.0}	80 {8.3}	< 0.001
Decaffeinated coffee					0.131
< 3/week	776 {97.5}	464 {98.1}	231 {96.7}	81 {96.4}	
3–4/week	12 {1.5}	4 {0.8}	7 {2.9}	1 {1.2}	
> 4/week	8 {1.0}	5 {1.1}	1 {0.4}	2 {2.4}	

Continuous variables are presented as median and interquartile range, and categorical variables are presented as absolute numbers and percentages.

*ACEi* angiotensin-converting enzyme inhibitor, *ARB* angiotensin receptor blocker, *BMI* body mass index, *ACE* angiotensin-converting enzyme inhibitor, *CAD* coronary artery disease, *HDL* high-density lipoprotein, *LDL* Low-density lipoprotein, *NT-proBNP* N-terminal pro-B-type natriuretic peptide, *PAD* peripheral artery disease.

### Coffee consumption and biomarkers and common cardiovascular risk factors

In multivariable linear regression analysis adjusted for age, sex, BMI, diabetes, arterial hypertension, smoking, additives, and lipid-lowering drugs, high coffee consumption was associated with raised LDL-cholesterol levels indicated by a beta of 5.92 (95% CI 2.95, 8.89, p < 0.001) (Table 2, Supplementary Table 2).

**Table 2:** Linear regression analysis of laboratories as well as echocardiographic and electrocardiographic parameters with moderate and high coffee consumption.

	Coffee consumption			
	Moderate (3–4 cups/day)		High (> 4 cups/day)	
	beta (95% CI)	p-value	beta (95% CI)	p-value
Laboratory				
Total cholesterol, mg/dl	1.09 (− 1.14, 3.33)	0.337	4.78 (1.56, 8.0)	0.004
LDL, mg/dl	1.63 (− 0.42, 3.68)	0.119	5.92 (2.95, 8.89)	< 0.001
HDL, mg/dl	0.57 (− 0.32, 1.46)	0.207	− 0.83 (− 2.11, 0.45)	0.206
NT-proBNP, ng/l	− 0.06 (− 0.11, − 0.02)	0.005	− 0.09 (− 0.15, − 0.02)	0.013
Vital signs				
Heart rate, bpm	− 0.62 (− 1.24, 0.01)	0.052	− 0.02 (− 0.92, 0.89)	0.969
Systolic blood pressure, mmHg	− 1.91 (− 3.04, − 0.78	0.001	− 3.06 (− 4.69, − 1.44)	< 0.001
Diastolic blood pressure, mmHg	− 1.05 {− 1.67, − 0.43}	0.001	− 1.85 {− 2.74, − 0.96}	< 0.001
Electrocardiography				
PQ interval, ms	0.42 (− 1.12, 1.96)	0.592	− 0.68 (− 2.89, 1.53)	0.548
QRS interval, ms	0.80 (0.01, 1.59)	0.048	− 0.43 (− 1.58, 0.71)	0.458
QTc interval, ms	− 3.18 (− 6.41, 0.05)	0.053	− 3.81 (− 8.49, 0.88)	0.111
Echocardiography				
LVEF, %	0.20 (− 0.12, 0.53)	0.238	0.12 (− 0.37, 0.60)	0.634
LV mass index, g/m^2^	− 0.06 (− 1.30, 1.17)	0.919	0.13 (− 1.65, 1.90)	0.887
E/e' mean ratio	− 0.10 (− 0.23, 0.03)	0.138	− 0.09 (− 0.27, 0.10)	0.347
TR Vmax, m/s	− 0.01 (− 0.03, 0.02)	0.623	− 0.01 (− 0.06, 0.03)	0.527
TAPSE, mm	0.16 (− 0.15, 0.47	0.322	0.27 (− 0.18, 0.71)	0.239
LASV, ml	− 0.06 (− 1.30, 1.17)	0.919	0.13 (− 1.65, 1.90)	0.887

Mild coffee consumption (< 3 cups/day) served as a reference. Adjustment was performed for age, sex, BMI, diabetes, arterial hypertension, smoking, and additives. For systolic and diastolic blood pressure no adjustment for arterial hypertension was performed, instead values for treated individuals were imputed by adding 15 mmHg and 10 mmHg respectively to the measured blood pressure. For cholesterol, LDL, and HDL additional adjustment for lipid-lowering drugs was performed. NT-proBNP was transformed with the natural logarithm. Abbreviations as in Table 1: *LASV* left atrial systolic volume, *LVEF* left ventricular ejection fraction, *TR Vmax* peak tricuspid regurgitation velocity; *TAPSE* tricuspid annular plane systolic excursion, *LASV* left atrial systolic volume.

Additionally, high coffee consumption demonstrated associations with total cholesterol with a beta of 4.78 (95% CI 1.56, 8.0; p = 0.004) and obesity (BMI ≥ 30 kg/m^2^) with an odds ratio (OR) of 1.32 (95% CI 1.08, 1.62; p = 0.008) (Tables 2 and 3, Supplementary Table 1).

**Table 3:** Logistic regression analysis of electrocardiographic findings, comorbidities, and cardiovascular diseases with moderate and high coffee consumption.

	Moderate (3–4 cups/day)		High (> 4 cups/day)	
	OR (95% CI)	p-value	OR (95% CI)	p-value
Electrocardiography				
LBBB	0.91 (0.68, 1.21)	0.527	0.73 (0.46, 1.11)	0.152
AV block	1.13 (0.88, 1.44)	0.345	0.82 (0.54, 1.19)	0.310
Atrial fibrillation	0.95 (0.73, 1.22)	0.699	0.69 (0.45, 1.04)	0.088
Comorbidities and cardiovascular diseases				
Diabetes mellitus	0.85 (0.67, 1.07)	0.166	0.91 (0.66, 1.23)	0.544
Obesity (BMI ≥ 30 kg/m^2^)	1.13 (0.97, 1.30)	0.115	1.32 (1.08, 1.62)	0.008
Coronary artery disease	0.93 (0.72, 1.19)	0.564	1.04 (0.72, 1.46)	0.832
Peripheral artery disease	1.20 (0.97, 1.48)	0.088	1.06 (0.78, 1.44)	0.693
Heart failure	0.85 (0.60, 1.20)	0.364	1.03 (0.62, 1.64)	0.912
HFpEF	0.77 (0.45, 1.25	0.308	0.91 (0.41, 1.80)	0.796
HF(m)rEF	0.95 (0.59–1.49)	0.837	1.14 (0.58–2.05)	0.690

Mild coffee consumption (< 3 cups/day) served as a reference. Adjustment was performed for age, sex, BMI, diabetes, arterial hypertension, smoking, and additives.

*AF* atrial fibrillation, *LBBB* left bundle branch block, *RBBB* right bundle branch block, *AV block* atrioventricular block, *HFpEF* heart failure with preserved ejection fraction, *HF(m)rEF* heart failure with (mildly) reduced ejection fraction.

### Coffee consumption and ECG/TTE variables

No relevant associations of coffee consumption with ECG parameters were detected in regression analysis. In line, neither morphological nor functional echocardiographic parameters correlated with coffee consumption (Table 2).

### Coffee consumption and blood pressure and cardiovascular diseases

In linear regression analysis, adjusted for age, sex, BMI, diabetes, smoking, and additives, moderate and high coffee consumption correlated with lower systolic (moderate: beta = − 1.91; 95% CI − 3.04, − 0.78; p = 0.001; high: beta = − 3.06; 95% CI − 4.69, − 1.44; p < 0.001) and diastolic blood pressure (moderate: beta = − 1.05; 95% CI − 1.67, − 0.43; p = 0.001; high: beta − 1.85; 95% CI − 2.74, − 0.96; p < 0.001) (Table 2, Supplementary Tables 5 and 6).

In contrast, coffee consumption showed no associations with CAD, and PAD. In our population, a total of 605 subjects were identified with the diagnosis of heart failure (Table 1). Nevertheless, neither heart failure in general nor differentiating into HFpEF and HF(m)rEF demonstrated significant associations with coffee consumption. In contrast, NT-proBNP was inversely associated with moderate (beta = − 0.06; 95% CI − 0.11, − 0.02; p = 0.005) and high (beta = − 0.09; 95% CI − 0.15, − 0.02; p = 0.013) coffee consumption (Table 2, Supplementary Table 4).

### Simultaneous consumption of caffeine-containing drinks, dietary patterns, and sex-specific differences

In order to address potential confounding by black and green tea as well as caffeinated soft-drinks we performed sensitivity analyses, excluding all subjects with simultaneous consumption of coffee and green and black tea. Since the coincidence of coffee and tea consumption is extremely high, this led to significant reduction of sample size and statistical power (n = 1480). High coffee consumption still demonstrated a trend towards associations with LDL and an inverse trend towards associations with systolic and diastolic bp lacking statistical significance. (Supplementary Tables 132–156). To exclude potential bias caused be the consumption of certain food components, additional adjustment for specific diets (vegetarian diet, vegan diet) was performed revealing no changes in the detected associations of coffee consumption (Supplementary Tables 157–180).

Sex-specific stratification of all our multivariable regression analyses as well as sensitivity analyses separated by sex showed no differences regarding our key findings (Supplementary Tables 80–131).

### Decaffeinated coffee consumption

From the overall cohort 807 subjects consumed decaffeinated coffee. Of those, 481 subjects consumed less than 3 cups/day, 241 subjects 3–4 cups/day, and 85 more than 4 cups/day. In linear regression analysis, adjusted for age, sex, BMI, diabetes, smoking, and additives, moderate and high decaffeinated coffee consumption correlated with lower diastolic (moderate: beta = − 2.05; 95% CI − 4.05, − 0.05; p = 0.045; high: beta − 3.79; 95% CI − 6.87, − 0.71; p < 0.001) and moderate decaffeinated coffee consumption with lower systolic blood pressure (moderate: beta = − 4.17; 95% CI − 7.88, − 0.45; p = 0.028; high: beta = − 5.01; 95% CI − 10.72, 0.69; p = 0.085) (Supplementary Tables 57–79). No further associations between decaffeinated coffee consumption and the assessed biomarkers, cardiovascular diseases, and ECG/TTE variables were detected.

## Discussion

In this study we demonstrate that coffee consumption was not associated with altered cardiac function and morphology, heart failure, and most of its risk factors. However, we observed an association with higher LDL-cholesterol levels and an inverse association with systolic and diastolic blood pressure.

Coffee is a complex liquid containing a multitude of compounds that could affect cardiovascular health including caffeine and polyphenols^16,17^. Whereas in earlier studies, detrimental effects of coffee consumption on cardiovascular health were promoted, recent studies favor a neutral or positive effect of moderate coffee consumption^2–5^.

The number of studies investigating associations of coffee consumption with echocardiographic parameters are scarce. Acute coffee intake seems to have no impact on cardiac function measured by echocardiography in healthy subjects^18^. Yet, in patients with CAD, coffee intake led to a decrease in left ventricular function, as well as a mild diastolic dysfunction possibly mediated by vasoconstriction and missing cardiac reserve in these patients^10^. In our community-based study, we did not depict relevant correlations of systolic or diastolic function with coffee consumption. In contrast, the CARDIA study indicated that low-to-moderate daily coffee consumption from early adulthood to middle age was associated with better LV systolic and diastolic function^11^. Additionally, several studies have suggested a favorable cardiovascular outcome and less heart failure for low- to moderate coffee consumption^7,9,19^. Accordingly, we observed a weak inverse association of coffee consumption with NT-proBNP. However, in our cross-sectional study there were no relevant associations of coffee consumption with heart failure or echocardiographic and electrocardiographic detectable HF precursors.

In line with most studies, we did not detect associations of coffee consumption with neither atrial fibrillation nor any other measured ECG time interval^20^. Only few studies addressed the topic of coffee consumption and ECG changes. In young healthy adults, moderate caffeine consumption showed no effect on the PR, QRS, QT and QTc intervals^21,22^. Supportive of these findings, we were not able to depict any associations between coffee consumption and ECG variables. Nevertheless, some studies reported beneficial effects of coffee consumption on atrial fibrillation^23^. Although caffeine induces the release of metanephrines and raises calcium sensitivity of the myocardium, our study showed no association of coffee consumption and atrial fibrillation^24,25^.

In line with previous observations, moderate and high coffee consumption was associated with increasing LDL-cholesterol levels^26^. Several studies on coffee consumption and lipids proposed that diterpenes, which are highly prevalent in unfiltered coffee, are the main drivers of a coffee-mediated increase in cholesterol levels^27,28^. In vitro, diterpenes mediated a reduction of LDL receptor activity^29^. Since the LDL receptor is responsible for the endocytic process of Apo B- and Apo E-containing lipoproteins, its suppression consequently leads to an extracellular accumulation of cholesterol. However, possible coffee-induced elevations of LDL-cholesterol were not accompanied by a rise in the prevalence of cardiovascular diseases such as coronary artery disease or peripheral artery disease.

Data on the effect of coffee on blood pressure are inconsistent^30^. Whereas several studies demonstrated an association of coffee consumption with elevated blood pressure, other studies were not able to reproduce any influence of coffee consumption on blood pressure^31,32^. Another meta-analysis even demonstrated a linear association between increasing coffee consumption and decreased risk of hypertension^33^. Possible explanations for these contradicting results might be attributed to differences in population genetics. Caffeine is mainly metabolized by Cytochrome P450 1A2. Variations in the CYP1A2 allele lead to a slower metabolization of caffeine and are associated with an increased risk for hypertension^34^. However, even the consumption of decaffeinated caffeine showed the same negative association with systolic and diastolic blood pressure, challenging the role of caffeine as the main driver of the described associations. The positive association with LDL-cholesterol and negative association with blood pressure might support the hypothesis of counterbalancing effects of coffee consumption on cardiovascular health.

### Limitations

The HCHS includes a sample from the middle-aged population of the German city of Hamburg with subjects mainly of Caucasian ascend. Accordingly, translations of our results to other ethnic groups should be done with caution. Since the amount of subjects suffering from heart failure was limited (n = 293 subjects), we decided to alter ESC HF Guidelines and consider HFrEF and HFmrEF as a joint HF(m)rEF group. This brings up the need for further studies, with larger sample sizes of subjects suffering from HF, allowing the distinction of HFmrEF and HFrEF.

As our study design is cross-sectional, only descriptions of associations but no causal claims can be made. Furthermore, all subjects have to answer the questionnaires by memory. Being asked about the last 12 months' behaviors and habits can always be distorted by either wrong recollection or deliberate misinformation.

Coffee is a highly complex beverage containing more than 1000 compounds acting as myriad bioactive substances. Conclusions about which substance, e.g. caffeine, derived antioxidants or diterpene alcohols, is responsible for the investigated effects, cannot be made.

Finally, coffee consumption might be associated with certain dietary patterns. While our regression analysis accounted for major dietary factors, the possibility of undetected confounding by additional nutritive and non-nutritive components cannot be completely ruled out.

## Conclusion

Our study provides new data on the associations of coffee consumption with cardiovascular health: LDL-cholesterol was positively, systolic and diastolic blood pressure inversely associated. Coffee consumption was not associated with cardiovascular diseases or altered cardiac structure or function suggesting possibly counterbalancing, neutral effects of coffee on cardiovascular health.

## Supplementary Information

Supplementary Tables.

## Data Availability

The data underlying this article cannot be shared publicly due to the privacy of individuals that participated in the study. The data will be shared on reasonable request to the corresponding author.
