# Supplementary material for: Coffee consumption and associations with blood pressure, LDL-cholesterol and echocardiographic measures in the general population
Source: Sci Rep. 2023 Mar 22;13:4668. doi: 10.1038/s41598-023-31857-5 (PMC10033706; doi:10.1038/s41598-023-31857-5)
Supplement: Supplementary file 1 — Supplementary Tables. [file 41598_2023_31857_MOESM1_ESM.docx]

**Supplements - Coffee consumption and its association with cardiovascular risk factors, cardiac morphology and function and cardiovascular diseases in the general population**

|  | | A)Total cholesterol | | B)Total cholesterol | | C)Total cholesterol | | D)Total cholesterol | | E)Total cholesterol | | F)Total cholesterol | |
| --- | --- | --- | --- | --- | --- | --- | --- | --- | --- | --- | --- | --- | --- |
|  | | *Estimates* | *p* | *Estimates* | *p* | *Estimates* | *p* | *Estimates* | *p* | *Estimates* | *p* | *Estimates* | *p* |
| Coffee consumption <3 cups/day | | 11.44 (7.21 – 15.67) | <0.001 | 8.31 (3.96 – 12.67) | <0.001 |  |  |  |  |  |  | 9.94 (5.01 – 14.86) | <0.001 |
| Coffee consumption 3-4 cups/day | | 10.99 (6.57 – 15.41) | <0.001 | 9.34 (4.79 – 13.88) | <0.001 | 1.19 (-1.04 – 3.42) | 0.295 | 1.09 (-1.14 – 3.33) | 0.337 | 4.76 (2.20 – 7.32) | <0.001 | 11.24 (5.86 – 16.62) | <0.001 |
| Coffee consumption > 4 cups/day | | 10.83 (5.97 – 15.68) | <0.001 | 12.45 (7.43 – 17.47) | <0.001 | 4.91 (1.70 – 8.12) | 0.003 | 4.78 (1.56 – 8.00) | 0.004 | 7.77 (4.49 – 11.06) | <0.001 | 11.25 (4.82 – 17.69) | 0.001 |
| Age | |  |  | 0.10 (-0.02 – 0.21) | 0.096 | 0.10 (-0.03 – 0.22) | 0.122 | 0.10 (-0.03 – 0.22) | 0.129 | 0.41 (0.30 – 0.52) | <0.001 | 0.07 (-0.15 – 0.29) | 0.509 |
| Female | |  |  | 20.53 (18.71 – 22.35) | <0.001 | 21.42 (19.48 – 23.36) | <0.001 | 21.37 (19.43 – 23.30) | <0.001 | 18.61 (16.87 – 20.35) | <0.001 | 22.88 (19.49 – 26.27) | <0.001 |
| Diabetes mellitus | |  |  | -20.42 (-23.83 – -17.02) | <0.001 | -20.62 (-24.26 – -16.98) | <0.001 | -20.87 (-24.52 – -17.22) | <0.001 | -13.90 (-17.21 – -10.59 | <0.001 | -20.53 (-26.69 – -14.36) | <0.001 |
| Arterial hypertension | |  |  | 1.85 (-0.22 – 3.91) | 0.079 | 2.18 (-0.00 – 4.37) | 0.050 | 2.32 (0.12 – 4.51) | 0.038 | 4.64 (2.66 – 6.61) | <0.001 | 3.13 (-0.74 – 6.99) | 0.113 |
| Current smoking | |  |  | -1.55 (-3.88 – 0.78) | 0.191 | -2.57 (-5.06 – -0.08) | 0.043 | -2.45 (-4.95 – 0.04) | 0.054 | -0.52 (-2.74 – 1.71) | 0.648 | -2.02 (-6.41 – 2.38) | 0.369 |
| BMI | |  |  | -0.25 (-0.46 – -0.05) | 0.017 | -0.28 (-0.50 – -0.06) | 0.013 | -0.29 (-0.51 – -0.07) | 0.010 | -0.09 (-0.29 – 0.11 | 0.366 | -0.27 (-0.65 – 0.11) | 0.159 |
| Additives | **Milk** |  |  |  |  | -2.19 (-4.38 – -0.00) | 0.049 |  |  |  |  |  |  |
|  | **Milk + Sugar** |  |  |  |  | -1.33 (-4.94 – 2.29) | 0.471 |  |  |  |  |  |  |
|  | **Milk + Sweetener** |  |  |  |  | -5.88 (-11.35 – -0.42) | 0.035 |  |  |  |  |  |  |
|  | **Sugar** |  |  |  |  | -0.57 (-8.26 – 7.12) | 0.884 |  |  |  |  |  |  |
|  | **Sweetener** |  |  |  |  | 4.24 (-4.59 – 13.07) | 0.347 |  |  |  |  |  |  |
|  | **Binary** |  |  |  |  |  |  | -2.18 (-4.29 – -0.07) | 0.043 |  |  |  |  |
| Coffee consumption 1-2 cups/day | |  |  |  |  |  |  |  |  | 5.09 (2.79 – 7.39) | <0.001 |  |  |
| Lipid lowering drugs | |  |  |  |  |  |  |  |  | -33.54 (-35.93 – -31.14) | <0.001 |  |  |

**Supplementary Table 1. Multivariable linear regression analysis for the association of moderate/high coffee consumption and total cholesterol.** Mild coffee consumption (<3 cups/d) served as the reference group. Adjustment was performed for: A) unadjusted; B) main cardiovascular risk factors; C) additional adjustment for additives (separately), D) additional adjustment for additives (binary), E) additional adjustment for lipid lowering drugs, F) same adjustment as for group A, but only for subjects who consumed coffee without any additives.

|  | | A)LDL | | B)LDL | | C)LDL | | D)LDL | | E)LDL | | F)LDL | |
| --- | --- | --- | --- | --- | --- | --- | --- | --- | --- | --- | --- | --- | --- |
|  | | *Estimates* | *p* | *Estimates* | *p* | *Estimates* | *p* | *Estimates* | *p* | *Estimates* | *p* | *Estimates* | *p* |
| Coffee consumption <3 cups/day | | 7.01 (3.22, 10.81) | <0.001 | 5.35 (1.35, 9.35) | 0.009 |  |  |  |  |  |  | 5.03 (0.54, 9.52) | 0.028 |
| Coffee consumption 3-4 cups/day | | 8.55 (4.58, 12.51) | <0.001 | 7.02 (2.85, 11.19) | 0.001 | 1.82 (-0.23, 3.87) | 0.081 | 1.63 (-0.42, 3.68) | 0.119 | 4.76 (2.20, 7.32) | <0.001 | 7.24 (2.33, 12.15) | 0.004 |
| Coffee consumption >4 cups/day] | | 11.10 (6.74, 15.45) | <0.001 | 10.62 (6.00, 15.23) | <0.001 | 6.09 (3.13, 9.05) | <0.001 | 5.92 (2.95, 8.89) | <0.001 | 7.77 (4.49, 11.06) | <0.001 | 9.14 (3.29, 14.99) | 0.002 |
| Age | |  |  | -0.01 (-0.11, 0.10) | 0.894 | 0.01 (-0.10, 0.12) | 0.851 | 0.01 (-0.11, 0.12) | 0.924 | 0.41 (0.30, 0.52) | <0.001 | 0.04 (-0.16, 0.24) | 0.720 |
| Female | |  |  | 8.31 (6.64, 9.97) | <0.001 | 9.20 (7.41, 10.98) | <0.001 | 9.04 (7.26, 10.82) | <0.001 | 18.61 (16.87, 20.35) | <0.001 | 10.20 (7.12, 13.28) | <0.001 |
| Diabetes mellitus | |  |  | -21.91 (-25.08, -18.73) | <0.001 | -22.04 (-25.44, -18.65) | <0.001 | -22.27 (-25.67, -18.86) | <0.001 | -13.90 (-17.21, -10.59) | <0.001 | -23.58 (-29.28, -17.89) | <0.001 |
| Arterial hypertension | |  |  | 0.18 (-1.71, 2.07) | 0.852 | 0.32 (-1.69, 2.33) | 0.755 | 0.44 (-1.58, 2.45) | 0.671 | 4.64 (2.66, 6.61) | <0.001 | 0.67 (-2.83, 4.18) | 0.706 |
| Current smoking | |  |  | 0.21 (-1.93 ,2.36) | 0.844 | -0.70 (-3.00, 1.59) | 0.548 | -0.48 (-2.77, 1.82) | 0.683 | -0.52 (-2.74, 1.71) |  | -1.42 (-5.44, 2.60) | 0.488 |
| BMI | |  |  | 0.35 (0.16, 0.54) | <0.001 | 0.34 (0.14, 0.54) | 0.001 | 0.34 (0.14, 0.54) | 0.001 | -0.09 (-0.29, 0.11) |  | 0.38 (0.04, 0.73) | 0.031 |
| Additives | **Milk** |  |  |  |  | -0.36 (-2.36, 1.65) | 0.727 |  |  |  |  |  |  |
|  | **Milk + Sugar** |  |  |  |  | 1.94 (-1.39, 5.26) | 0.253 |  |  |  |  |  |  |
|  | **Milk + Sweetener** |  |  |  |  | -2.45 (-7.52, 2.62) | 0.343 |  |  |  |  |  |  |
|  | **Sugar** |  |  |  |  | 2.02 (-5.05, 9.09) | 0.575 |  |  |  |  |  |  |
|  | **Sweetener** |  |  |  |  | 3.24 (-4.89, 11.36) | 0.435 |  |  |  |  |  |  |
|  | **Binary** |  |  |  |  |  |  | -0.07 (-2.00, 1.87) | 0.947 |  |  |  |  |
| Coffee consumption 1-2 cups/day | |  |  |  |  |  |  |  |  | 5.09 (2.79, 7.39) | <0.001 |  |  |
| Lipid lowering drugs | |  |  |  |  |  |  |  |  | -33.54 (-35.93, -31.14) | <0.001 |  |  |

**Supplementary Table 2. Multivariable linear regression analysis for the association of moderate/high coffee consumption and LDL.** Mild coffee consumption (<3 cups/d) served as the reference group. Adjustment was performed for: A) unadjusted; B) main cardiovascular risk factors; C) additional adjustment for additives (separately), D) additional adjustment for additives (binary), E) additional adjustment for lipid lowering drugs, F) same adjustment as for group A, but only for subjects who consumed coffee without any additives.

|  | | A)HDL | | B)HDL | | C)HDL | | D)HDL | | E)HDL | | F)HDL | |
| --- | --- | --- | --- | --- | --- | --- | --- | --- | --- | --- | --- | --- | --- |
|  | | *Estimates* | *p* | *Estimates* | *p* | *Estimates* | *p* | *Estimates* | *p* | *Estimates* | *p* | *Estimates* | *p* |
| Coffee consumption <3 cups/day] | | 4.61 (2.67, 6.56) | <0.001 | 3.28 (1.56, 5.00) | <0.001 |  |  |  |  |  |  | 5.32 (3.37, 7.27) | <0.001 |
| Coffee consumption 3-4 cups/day | | 3.29 (1.26, 5.32) | 0.001 | 3.87 (2.08, 5.66) | <0.001 | 0.50 (-0.39, 1.38) | 0.270 | 0.57 (-0.32, 1.46) | 0.207 | 4.76 (2.20, 7.32) | <0.001 | 5.02 (2.88, 7.15) | <0.001 |
| Coffee consumption > 4 cups/day | | -1.35 (-3.58, 0.88) | 0.237 | 2.89 (0.91, 4.87) | 0.004 | -0.86 (-2.13, 0.42) | 0.188 | -0.83 (-2.11, 0.45) | 0.206 | 7.77 (4.49, 11.06) | <0.001 | 5.00 (2.45, 7.55) | <0.001 |
| Age | |  |  | 0.15 (0.11, 0.20) | <0.001 | 0.13 (0.08, 0.18) | <0.001 | 0.14 (0.09, 0.19) | <0.001 | 0.41 (0.30, 0.52) | <0.001 | 0.11 (0.03, 0.20) | 0.011 |
| Female | |  |  | 15.25 (14.53, 15.97) | <0.001 | 15.20 (14.43, 15.97) | <0.001 | 15.38 (14.61, 16.15) | <0.001 | 18.61 (16.87, 20.35) | <0.001 | 15.57 (14.23, 16.92) | <0.001 |
| Diabetes mellitus | |  |  | -6.21 (-7.55, -4.86) | <0.001 | -6.47 (-7.92, -5.03) | <0.001 | -6.47 (-7.92, -5.02) | <0.001 | -13.90 (-17.21, -10.59) | <0.001 | -6.43 (-8.87, -3.98) | <0.001 |
| Arterial hypertension | |  |  | -0.41 (-1.23, 0.40) | 0.320 | -0.21 (-1.07, 0.66) | 0.642 | -0.14 (-1.01, 0.74) | 0.761 | 4.64 (2.66, 6.61) | <0.001 | -0.18 (-1.71, 1.35) | 0.821 |
| Current smoking | |  |  | -5.60 (-6.52, -4.68) | <0.001 | -5.54 (-6.53, -4.55) | <0.001 | -5.63 (-6.62, -4.64) | <0.001 | -0.52 (-2.74, 1.71) | 0.648 | -5.73 (-7.47, -3.98) | <0.001 |
| BMI | |  |  | -1.36 (-1.44, -1.28) | <0.001 | -1.37 (-1.46, -1.28) | <0.001 | -1.38 (-1.47, -1.29) | <0.001 | -0.09 (-0.29, 0.11) | 0.366 | -1.38 (-1.53, -1.22) | <0.001 |
| Additives | **Milk** |  |  |  |  | -1.98 (-2.84, -1.11) | <0.001 |  |  |  |  |  |  |
|  | **Milk + Sugar** |  |  |  |  | -4.29 (-5.73, -2.85) | <0.001 |  |  |  |  |  |  |
|  | **Milk + Sweetener** |  |  |  |  | -4.28 (-6.45, -2.11) | <0.001 |  |  |  |  |  |  |
|  | **Sugar** |  |  |  |  | -4.03 (-7.08, -0.98) | 0.010 |  |  |  |  |  |  |
|  | **Sweetener** |  |  |  |  | 0.62 (-2.89, 4.13) | 0.729 |  |  |  |  |  |  |
|  | **Binary** |  |  |  |  |  |  | -2.42 (-3.25, -1.58) | <0.001 |  |  |  |  |
| Coffee consumption 1-2 cups/day | |  |  |  |  |  |  |  |  | 5.09 (2.79, 7.39) | <0.001 |  |  |
| Lipid lowering drugs | |  |  |  |  |  |  |  |  | -33.54 (-35.93, -31.14 | <0.001 |  |  |

**Supplementary Table 3. Multivariable linear regression analysis for the association of moderate/high coffee consumption and HDL.** Mild coffee consumption (<3 cups/d) served as the reference group. Adjustment was performed for: A) unadjusted; B) main cardiovascular risk factors; C) additional adjustment for additives (separately), D) additional adjustment for additives (binary), E) additional adjustment for lipid lowering drugs, F) same adjustment as for group A, but only for subjects who consumed coffee without any additives.

|  | | A) NT pro BNP | | B) NT pro BNP | | C) NT pro BNP | | D) NT pro BNP | | E) NT pro BNP | |
| --- | --- | --- | --- | --- | --- | --- | --- | --- | --- | --- | --- |
|  | | *Estimates* | *p* | *Estimates* | *p* | *Estimates* | *p* | *Estimates* | *p* | *Estimates* | *p* |
| Coffee consumption < 3 cups/day | | 0.08 (-0.01, 0.18) | 0.087 | -0.12 (-0.21, -0.03) | 0.009 |  |  |  |  | -0.09 (-0.19, 0.01) | 0.077 |
| Coffee consumption 3-4 cups/day | | -0.15 (-0.25, -0.05) | 0.003 | -0.17 (-0.27, -0.08) | <0.001 | -0.07 (-0.11, -0.02) | 0.004 | -0.06 (-0.11, -0.02) | 0.008 | -0.16 (-0.27, -0.04) | 0.006 |
| Coffee consumption >4 cups/day | | -0.24 (-0.35, -0.13) | <0.001 | -0.18 (-0.29, -0.08) | 0.001 | -0.08 (-0.15, -0.02) | 0.015 | -0.09 (-0.15, -0.02) | 0.013 | -0.22 (-0.35, -0.08) | 0.001 |
| Age | |  |  | 0.05 (0.05, 0.05) | <0.001 | 0.05 (0.04, 0.05) | <0.001 | 0.05 (0.04, 0.05) | <0.001 | 0.05 (0.04, 0.05) | <0.001 |
| Female | |  |  | 0.39 (0.36, 0.43) | <0.001 | 0.38 (0.34, 0.42) | <0.001 | 0.38 (0.34, 0.42) | <0.001 | 0.37 (0.30, 0.44) | <0.001 |
| Diabetes mellitus | |  |  | -0.03 (-0.10, 0.04) | 0.463 | -0.04 (-0.11, 0.04) | 0.366 | -0.03 (-0.10, 0.05) | 0.464 | 0.11 (-0.02, 0.24) | 0.091 |
| Arterial hypertension | |  |  | 0.17 (0.13, 0.21) | <0.001 | 0.18 (0.13, 0.22) | <0.001 | 0.18 (0.14, 0.23) | <0.001 | 0.19 (0.11, 0.27) | <0.001 |
| Current smoking | |  |  | 0.07 (0.02, 0.12) | 0.003 | 0.08 (0.03, 0.13) | 0.002 | 0.08 (0.03, 0.13) | 0.003 | 0.08 (-0.01, 0.17) | 0.072 |
| BMI | |  |  | -0.01 (-0.01, -0.01) | <0.001 | -0.01 (-0.02, -0.01) | <0.001 | -0.01 (-0.02, -0.01) | <0.001 | -0.01 (-0.02, -0.01) | 0.001 |
| Additives | **Milk** |  |  |  |  | -0.00 (-0.05, 0.04) | 0.935 |  |  |  |  |
|  | **Milk + Sugar** |  |  |  |  | -0.08 (-0.15, -0.00) | 0.049 |  |  |  |  |
|  | **Milk + Sweetener** |  |  |  |  | -0.08 (-0.19, 0.04) | 0.189 |  |  |  |  |
|  | **Sugar** |  |  |  |  | -0.08 (-0.24, 0.08) | 0.327 |  |  |  |  |
|  | **Sweetener** |  |  |  |  | -0.09 (-0.27, 0.10) | 0.352 |  |  |  |  |
|  | **Binary** |  |  |  |  |  |  | -0.02 (-0.06, 0.03) | 0.449 |  |  |

**Supplementary Table 4. Multivariable linear regression analysis for the association of moderate/high coffee consumption and NT proBNP.** Mild coffee consumption (<3 cups/d) served as the reference group. Adjustment was performed for: A) unadjusted; B) main cardiovascular risk factors; C) additional adjustment for additives (separately), D) additional adjustment for additives (binary), E) same adjustment as for group A, but only for subjects who consumed coffee without any additives. Abbreviations as in Table 1.

|  | | A)SBP | | B)SBP | | C)SBP | | D)SBP | | E)SBP | |
| --- | --- | --- | --- | --- | --- | --- | --- | --- | --- | --- | --- |
|  | | *Estimates* | *p* | *Estimates* | *p* | *Estimates* | *p* | *Estimates* | *p* | *Estimates* | *p* |
| Coffee consumption <3 cups/day | | 4.65 (2.40, 6.89) | <0.001 | 2.61 (0.42, 4.80) | 0.019 |  |  |  |  | 3.58 (1.08, 6.07) | 0.005 |
| Coffee consumption 3-4 cups/day | | 0.07 (-2.28, 2.42) | 0.953 | 0.81 (-1.48, 3.09) | 0.489 | -1.95 (-3.08, -0.83) | 0.001 | -1.91 (-3.04, -0.78) | 0.001 | 1.85 (-0.87, 4.58) | 0.182 |
| Coffee consumption >4 cups/day | | -0.47 (-3.06, 2.13) | 0.724 | -0.23 (-2.75, 2.29) | 0.858 | -3.25 (-4.87, -1.63) | <0.001 | -3.06 (-4.69, -1.44) | <0.001 | -1.28 (-4.53, 1.98) | 0.442 |
| Age | |  |  | 0.83 (0.77, 0.88) | <0.001 | 0.80 (0.74, 0.86) | <0.001 | 0.80 (0.74, 0.86) | <0.001 | 0.80 (0.70, 0.91) | <0.001 |
| Female | |  |  | -5.08 (-5.98, -4.17) | <0.001 | -5.17 (-6.14, -4.19) | <0.001 | -5.08 (-6.05, -4.10) | <0.001 | -5.34 (-7.04, -3.63) | <0.001 |
| Diabetes mellitus | |  |  | 3.96 (2.25, 5.67) | <0.001 | 4.03 (2.18, 5.87) | <0.001 | 4.21 (2.36, 6.06) | <0.001 | 2.40 (-0.74, 5.54) | 0.135 |
| Current smoking | |  |  | -1.66 (-2.82, -0.49) | 0.005 | -1.71 (-2.96, -0.45) | 0.008 | -1.88 (-3.13, -0.62) | 0.003 | -2.49 (-4.71, -0.27) | 0.028 |
| BMI | |  |  | 0.73 (0.63, 0.83) | <0.001 | 0.67 (0.56, 0.78) | <0.001 | 0.68 (0.57, 0.79) | <0.001 | 0.80 (0.61, 0.98) | <0.001 |
| Additives | **Milk** |  |  |  |  | -0.76 (-1.86, 0.35) | 0.178 |  |  |  |  |
|  | **Mlk + Sugar** |  |  |  |  | -2.54 (-4.36, -0.72) | 0.006 |  |  |  |  |
|  | **Milk + Sweetener** |  |  |  |  | 0.84 (-1.90, 3.57) | 0.548 |  |  |  |  |
|  | **Sugar** |  |  |  |  | -1.86 (-5.72, 2.00) | 0.344 |  |  |  |  |
|  | **Sweetener** |  |  |  |  | 3.27 (-1.22, 7.75) | 0.154 |  |  |  |  |
|  | **Binary** |  |  |  |  |  |  | -0.87 (-1.93, 0.20) | 0.111 |  |  |

**Supplementary Table 5. Multivariable linear regression analysis for the association of moderate/high coffee consumption and SBP.** Mild coffee consumption (<3 cups/d) served as the reference group. Adjustment was performed for: A) unadjusted; B) main cardiovascular risk factors; C) additional adjustment for additives (separately), D) additional adjustment for additives (binary), E) same adjustment as for group A, but only for subjects who consumed coffee without any additives. Abbreviations as in Table 1.

|  | | A)DBP | | B)DBP | | C)DBP | | D)DBP | | E)DBP | |
| --- | --- | --- | --- | --- | --- | --- | --- | --- | --- | --- | --- |
|  | | *Estimates* | *p* | *Estimates* | *p* | *Estimates* | *p* | *Estimates* | *p* | *Estimates* | *p* |
| Coffee consumption < 3 cups/day | | 1.60 (0.44, 2.77) | 0.007 | 1.81 (0.61, 3.01) | 0.003 |  |  |  |  | 2.39 (1.02, 3.76) | 0.001 |
| Coffee consumption 3-4 cups/day | | 0.48 (-0.74, 1.69) | 0.442 | 0.87 (-0.38, 2.12) | 0.172 | -1.05 (-1.67, -0.43) | 0.001 | -1.05 (-1.67, -0.43) | 0.001 | 1.41 (-0.08, 2.91) | 0.064 |
| Coffee consumption >4 cups/day | | 0.74 (-0.60, 2.08) | 0.278 | 0.39 (-0.99, 1.77) | 0.582 | -1.87 (-2.76, -0.98) | <0.001 | -1.85 (-2.74, -0.96) | <0.001 | -0.36 (-2.15, 1.42) | 0.692 |
| Age | |  |  | 0.05 (0.02, 0.08) | <0.001 | 0.03 (0.00, 0.07) | 0.050 | 0.03 (0.00, 0.07) | 0.038 | 0.04 (-0.02, 0.10) | 0.203 |
| Female | |  |  | -3.00 (-3.50, -2.50) | <0.001 | -3.11 (-3.65, -2.58) | <0.001 | -3.08 (-3.62, -2.55) | <0.001 | -3.40 (-4.34, -2.47) | <0.001 |
| Diabetes mellitus | |  |  | -0.10 (-1.03, 0.84) | 0.839 | 0.06 (-0.95, 1.08) | 0.900 | 0.20 (-0.82, 1.21) | 0.704 | -0.94 (-2.66, 0.79) | 0.286 |
| Current smoking | |  |  | -0.92 (-1.56, -0.28) | 0.005 | -0.85 (-1.54,-0.16) | 0.016 | -0.90 (-1.59, -0.21) | 0.011 | -1.68 (-2.90, -0.46) | 0.007 |
| BMI | |  |  | 0.48 (0.42, 0.53) | <0.001 | 0.44 (0.38, 0.50) | <0.001 | 0.45 (0.39, 0.51) | <0.001 | 0.44 (0.34, 0.54) | <0.001 |
| Additives | **Milk** |  |  |  |  | -0.37 (-0.97, 0.24) | 0.235 |  |  |  |  |
|  | **Milk + Sugar** |  |  |  |  | -1.11 (-2.11, -0.11) | 0.030 |  |  |  |  |
|  | **Milk + Sweetener** |  |  |  |  | 0.06 (-1.44, 1.57) | 0.933 |  |  |  |  |
|  | **Sugar** |  |  |  |  | -0.57 (-2.69, 1.55) | 0.599 |  |  |  |  |
|  | **Sweetener** |  |  |  |  | 0.49 (-1.97, 2.96) | 0.694 |  |  |  |  |
|  | **Binary** |  |  |  |  |  |  | -0.42 (-1.01, 0.16) | 0.155 |  |  |

**Supplementary Table 6. Multivariable linear regression analysis for the association of moderate/high coffee consumption and DBP.** Mild coffee consumption (<3 cups/d) served as the reference group. Adjustment was performed for: A) unadjusted; B) main cardiovascular risk factors; C) additional adjustment for additives (separately), D) additional adjustment for additives (binary), E) same adjustment as for group A, but only for subjects who consumed coffee without any additives. Abbreviations as in Table 1.

|  | | A) Total cholesterol | | B) Total cholesterol | | C) Total cholesterol | | D) Total cholesterol | | E) Total cholesterol | | F) Total cholesterol | | |
| --- | --- | --- | --- | --- | --- | --- | --- | --- | --- | --- | --- | --- | --- | --- |
|  | | *Estimates* | *p* | *Estimates* | *p* | *Estimates* | *p* | *Estimates* | *p* | *Estimates* | *p* | *Estimates* | *p* |
| Coffee consumption 3-4 cups/day | | 3.42 (-3.30, 10.15) | 0.318 | 3.05 (-3.84, 9.94) | 0.385 | 2.04 (-5.07, 9.15) | 0.573 | 2.08 (-5.04, 9.19) | 0.567 | 2.82 (-3.69, 9.34) | 0.395 | 4.48 (-8.49, 17.45) | 0.496 |
| Coffee consumption > 4 cups/day | | -2.71 (-12.80, 7.37) | 0.598 | 2.11 (-8.22, 12.43) | 0.689 | 1.70 (-9.31, 12.71) | 0.762 | 1.49 (-9.54, 12.51) | 0.791 | 3.02 (-6.72, 12.77) | 0.543 | 0.37 (-16.89, 17.64) | 0.966 |
| Age | |  |  | -0.17 (-0.56, 0.22) | 0.391 | -0.21 (-0.62, 0.20) | 0.316 | -0.20 (-0.62, 0.21) | 0.327 | 0.22 (-0.16, 0.60) | 0.248 | -0.52 (-1.30, 0.26) | 0.190 |
| Female | |  |  | 22.99 (16.76, 29.22) | **<0.001** | 23.04 (16.54, 29.54) | **<0.001** | 23.21 (16.73, 29.70) | **<0.001** | 21.28 (15.39, 27.18) | **<0.001** | 22.86 (11.18, 34.54) | **<0.001** |
| Diabetes mellitus | |  |  | -31.16 (-41.92, -20.40) | **<0.001** | -29.55 (-41.16, -17.95) | **<0.001** | -30.61 (-42.25, -18.96) | **<0.001** | 20.80 (-31.31, -10.28) | **<0.001** | -27.76 (-51.41, -4.12) | **0.022** |
| Arterial hypertension | |  |  | 8.39 (1.10, 15.67) | **0.024** | 7.21 (-0.36, 14.77) | 0.062 | 6.61 (-0.93, 14.14) | 0.086 | 11.57 (4.66, 18.48) | **0.001** | 13.21 (-0.65, 27.06) | 0.062 |
| Current smoking | |  |  | -5.38 (-13.06, 2.30) | 0.170 | -5.61 (-13.68 ,2.46) | 0.173 | -4.96 (-13.01, 3.10) | 0.227 | -3.01 (-10.28, 4.26) | 0.416 | -5.87 (-20.86, 9.12) | 0.441 |
| BMI | |  |  | -0.00 (-0.70, 0.70) | 0.995 | 0.08 (-0.66, 0.83) | 0.824 | 0.09 (-0.65, 0.84) | 0.806 | -0.00 (-0.66, 0.66) | 0.992 | -0.56 (-1.85, 0.72) | 0.388 |
| Additives | **Milk** |  |  |  |  | -5.54 (-12.46, 1.37) | 0.116 |  |  |  |  |  |  |
| **Milk + Sugar** |  |  |  |  | 3.41 (-11.36, 18.18) | 0.651 |  |  |  |  |  |  |
| **Milk + Sweetener** |  |  |  |  | -2.27 (-19.90, 15.36) | 0.800 |  |  |  |  |  |  |
| **Sugar** |  |  |  |  | 0.22 (-39.21, 39.65) | 0.991 |  |  |  |  |  |  |
| **Sweetener** |  |  |  |  | 1.96 (-22.41, 26.34) | 0.874 |  |  |  |  |  |  |
| **Binary** |  |  |  |  |  |  | -4.64 (-11.30, 2.03) | 0.172 |  |  |  |  |
| Lipid lowering drugs | |  |  |  |  |  |  |  |  | -35.67 (-43.28, -28.06) | **<0.001** |  |  |

**Supplementary Table 7. Multivariable linear regression analysis for the association of moderate/high coffee consumption and total cholesterol.** For this analysis only subjects without simultaneous consumption of caffeinated drinks were included. Mild coffee consumption (<3 cups/d) served as the reference group. Adjustment was performed for: A) unadjusted; B) main cardiovascular risk factors; C) additional adjustment for additives (separately), D) additional adjustment for additives (binary), E) additional adjustment for lipid lowering drugs, F) same adjustment as for group A, but only for subjects who consumed coffee without any additives.

|  | | A)LDL | | B)LDL | | C)LDL | | D)LDL | | E)LDL | | F)LDL | | |
| --- | --- | --- | --- | --- | --- | --- | --- | --- | --- | --- | --- | --- | --- | --- |
|  | | *Estimates* | *p* | *Estimates* | *p* | *Estimates* | *p* | *Estimates* | *p* | *Estimates* | *p* | *Estimates* | *p* |  |
| Coffee consumption 3-4 cups/day | | 3.19 (-2.73, 9.12) | 0.290 | 2.74 (-3.42, 8.91) | 0.382 | 1.53 (-4.84, 7.90) | 0.637 | 1.37 (-5.02, 7.76) | 0.674 | 2.53 (-3.29, 8.36) | 0.393 | 6.03 (-5.49, 17.54) | 0.304 |  |
| Coffee consumption > 4 cups/day | | 4.10 (-4.81, 13.01) | 0.366 | 6.49 (-2.79, 15.77) | 0.170 | 6.52 (-3.41, 16.45) | 0.198 | 5.99 (-3.95, 15.94) | 0.237 | 7.09 (-1.66, 15.84) | 0.112 | 4.78 (-10.55, 20.12) | 0.539 |  |
| Age | |  |  | -0.17 (-0.52, 0.19) | 0.356 | -0.19 (-0.56, 0.18) | 0.305 | -0.20 (-0.57, 0.16) | 0.276 | 0.19 (-0.15, 0.52) | 0.284 | -0.40 (-1.09, 0.30) | 0.260 |  |
| Female | |  |  | 9.69 (4.10, 15.27) | 0.001 | 9.51 (3.66, 15.36) | 0.001 | 9.44 (3.60, 15.28) | 0.002 | 8.18 (2.89, 13.46) | 0.002 | 9.01 (-1.36, 19.38) | 0.088 |  |
| Diabetes mellitus | |  |  | -31.57 (-41.34, -21.81) | <0.001 | -29.52 (-40.09, -18.96) | <0.001 | -29.61 (-40.17, -19.05) | <0.001 | -22.12 (-31.66, -12.58) | <0.001 | -29.42 (-50.42, -8.41) | 0.006 |  |
| Arterial hypertension | |  |  | 5.50 (-1.02, 12.02) | 0.098 | 4.23 (-2.55, 11.00) | 0.221 | 3.82 (-2.95, 10.60) | 0.268 | 8.33 (2.15, 14.51) | 0.008 | 8.25 (-4.05, 20.56) | 0.187 |  |
| Current smoking | |  |  | -2.66 (-9.55, 4.24) | 0.449 | -3.06 (-10.31, 4.18) | 0.407 | -2.21 (-9.45, 5.04) | 0.550 | -0.40 (-6.92, 6.12) | 0..904 | -1.15 (-14.46, 12.16) | 0.865 |  |
| BMI | |  |  | 0.73 (0.10, 1.36) | 0.022 | 0.84 (0.17, 1.51) | 0.014 | 0.85 (0.18, 1.53) | 0.013 | 0.73 (0.13, 1.32) | 0.016 | 0.20 (-0.94, 1.35) | 0.724 |  |
| Additives | **Mlik** |  |  |  |  | -2.00 (-8.19, 4.20) | 0.527 |  |  |  |  |  |  |  |
|  | **Milk + Sugar** |  |  |  |  | 5.63 (-7.59, 18.85) | 0.403 |  |  |  |  |  |  |  |
|  | **Milk + Sweetener** |  |  |  |  | 1.12 (-14.96, 17.20) | 0.891 |  |  |  |  |  |  |  |
|  | **Sugar** |  |  |  |  | 17.56 (-17.71, 52.84) | 0.329 |  |  |  |  |  |  |  |
|  | **Sweetener** |  |  |  |  | 1.01 (-21.78, 23.79) | 0.931 |  |  |  |  |  |  |  |
|  | **Binary** |  |  |  |  |  |  | -1.05 (-7.04 , 4.94) | 0.731 |  |  |  |  |  |
| Lipid lowering drugs | |  |  |  |  |  |  |  |  | -4.57 (-7.85, -1.30) | 0.006 |  |  |  |

**Supplementary Table 8. Multivariable linear regression analysis for the association of moderate/high coffee consumption and LDL including sensitivity analysis excluding consumption of carbonated drinks.** For this analysis only subjects without simultaneous consumption of caffeinated drinks were included. Mild coffee consumption (<3 cups/d) served as the reference group. Adjustment was performed for: A) unadjusted; B) main cardiovascular risk factors; C) additional adjustment for additives (separately), D) additional adjustment for additives (binary), E) additional adjustment for lipid lowering drugs, F) same adjustment as for group A, but only for subjects who consumed coffee without any additives. . Abbreviations as in Table 1.

|  | | A)HDL | | B)HDL | | C)HDL | | D)HDL | | E)HDL | | F)HDL | |
| --- | --- | --- | --- | --- | --- | --- | --- | --- | --- | --- | --- | --- | --- |
|  | | *Estimates* | *p* | *Estimates* | *p* | *Estimates* | *p* | *Estimates* | *p* | *Estimates* | *p* | *Estimates* | *p* |
| Coffee consumption 3-4 cups/day | | 0.84 (-2.26, 3.93) | 0.595 | 1.59 (-1.23, 4.40) | 0.269 | 1.59 (-1.35, 4.53) | 0.287 | 1.69 (-1.27, 4.65) | 0.262 | 1.60 (-1.21, 4.41) | 0.263 | -0.43 (-6.09, 5.22) | 0.880 |
| Coffee consumption > 4 cups/day | | -6.32 (-10.96, -1.68) | 0.008 | -1.69 (-5.91, 2.52) | 0.431 | -2.70 (-7.26, 1.85) | 0.244 | -2.85 (-7.44, 1.73) | 0.221 | -1.54 (-5.74, 2.66) | 0.473 | -1.46 (-8.99, 6.07) | 0.702 |
| Age | |  |  | 0.00 (-0.16, 0.16) | 0.975 | -0.05 (-0.22, 0.12) | 0.580 | -0.04 (-0.21, 0.13) | 0.662 | 0.05 (-0.11, 0.22) | 0.530 | -0.12 (-0.46, 0.22) | 0.482 |
| Female | |  |  | 15.10 (12.55, 17.64) | <0.001 | 14.81 (12.13, 17.50) | <0.001 | 14.96 (12.26, 17.65) | <0.001 | 14.91 (12.37, 17.44) | <0.001 | 15.12 (10.02, 20.21) | <0.001 |
| Diabetes mellitus | |  |  | -5.44 (-9.83, -1.05) | 0.015 | -5.09 (-9.90, -0.29) | 0.038 | -5.40 (-10.24, -0.56) | 0.029 | -4.29 (-8.82, 0.23) | 0.063 | -8.38 (-18.70, 1.93) | 0.110 |
| Arterial hypertension | |  |  | -0.28 (-3.25, 2.70) | 0.855 | 0.09 (-3.04, 3.22) | 0.953 | 0.02 (-3.11, 3.16) | 0.988 | 0.13 (-2.84, 3.11) | 0.929 | 3.11 (-2.93, 9.16) | 0.311 |
| Current smoking | |  |  | -6.50 (-9.63, -3.36) | <0.001 | -6.06 (-9.40, -2.73) | <0.001 | -6.18 (-9.52, -2.83) | <0.001 | -6.18 (-9.31, -3.05) | <0.001 | -9.22 (-15.75, -2.68) | 0.006 |
| BMI | |  |  | -1.37 (-1.65, -1.08) | <0.001 | -1.42 (-1.72, -1.11) | <0.001 | -1.43 (-1.74, -1.12) | <0.001 | -1.36 (-1.65, -1.08) | <0.001 | -1.40 (-1.96, -0.84) | <0.001 |
| Additives | **Milk** |  |  |  |  | -4.28 (-7.14, -1.42) | 0.003 |  |  |  |  |  |  |
| **Milk + Sugar** |  |  |  |  | -4.40 (-10.51, 1.71) | 0.158 |  |  |  |  |  |  |
| **Milk + Sweetener** |  |  |  |  | -7.58 (-14.88, -0.29) | 0.041 |  |  |  |  |  |  |
| **Sugar** |  |  |  |  | -13.30 (-29.60, 3.01) | 0.110 |  |  |  |  |  |  |
| **Sweetener** |  |  |  |  | -4.32 (-14.40, 5.76) | 0.401 |  |  |  |  |  |  |
| **Binary** |  |  |  |  |  |  | -4.63 (-7.40, -1.86) | 0.001 |  |  |  |  |
| Lipid lowering drugs | |  |  |  |  |  |  |  |  | -4.57 (-7.85, -1.30) | 0.006 |  |  |

**Supplementary Table 9. Multivariable linear regression analysis for the association of moderate/high coffee consumption and HDL including sensitivity analysis excluding consumption of carbonated drinks.** For this analysis only subjects without simultaneous consumption of caffeinated drinks were included. Mild coffee consumption (<3 cups/d) served as the reference group. Adjustment was performed for: A) unadjusted; B) main cardiovascular risk factors; C) additional adjustment for additives (separately), D) additional adjustment for additives (binary), E) additional adjustment for lipid lowering drugs, F) same adjustment as for group A, but only for subjects who consumed coffee without any additives. Abbreviations as in Table 1.

|  | | A)NT proBNP | | B)NT proBNP | | C)NT proBNP | | D)NT proBNP | | E)NT proBNP | | |
| --- | --- | --- | --- | --- | --- | --- | --- | --- | --- | --- | --- | --- |
|  | | *Estimates* | *p* | *Estimates* | *p* | *Estimates* | *p* | *Estimates* | *p* | *Estimates* | *p* |
| Coffee consumption 3-4 cups/day | | -0.19 (-0.33, -0.04) | 0.010 | -0.04 (-0.18, 0.10) | 0.583 | -0.02 (-0.16, 0.12) | 0.759 | -0.01 (-0.16, 0.13) | 0.841 | 0.19 (-0.06, 0.44) | 0.134 |
| Coffee consumption > 4 cups/day | | -0.33 (-0.55, -0.12) | 0.003 | -0.07 (-0.28, 0.13) | 0.482 | -0.07 (-0.29, 0.15) | 0.548 | -0.06 (-0.28, 0.16) | 0.610 | 0.08 (-0.25, 0.42) | 0.619 |
| Age | |  |  | 0.05 (0.04, 0.06) | <0.001 | 0.05 (0.04, 0.06) | <0.001 | 0.05 (0.04 – 0.06) | <0.001 | 0.05 (0.04, 0.07) | <0.001 |
| Female | |  |  | 0.35 (0.23, 0.47) | <0.001 | 0.34 (0.21, 0.47) | <0.001 | 0.34 (0.21, 0.47) | <0.001 | 0.37 (0.14, 0.59) | 0.002 |
| Diabetes mellitus | |  |  | 0.01 (-0.20, 0.23) | 0.895 | -0.03 (-0.26, 0.20) | 0.824 | -0.04 (-0.27, 0.19) | 0.712 | 0.30 (-0.14, 0.73) | 0.181 |
| Arterial hypertension | |  |  | 0.11 (-0.04, 0.25) | 0.156 | 0.10 (-0.05, 0.26) | 0.176 | 0.11 (-0.04, 0.26) | 0.143 | 0.01 (-0.26, 0.28) | 0.942 |
| Current smoking | |  |  | 0.01 (-0.14, 0.16) | 0.881 | 0.04 (-0.12, 0.20) | 0.657 | 0.03 (-0.13, 0.20) | 0.673 | 0.14 (-0.15, 0.43) | 0.343 |
| BMI | |  |  | -0.01 (-0.02, 0.01) | 0.351 | -0.01 (-0.02, 0.01) | 0.269 | -0.01 (-0.02, 0.01) | 0.254 | -0.00 (-0.03, 0.02) | 0.942 |
| Additives | **Milk** |  |  |  |  | -0.00 (-0.14, 0.14) | 0.996 |  |  |  |  |
| **Milk + Sugar** |  |  |  |  | -0.17 (-0.47, 0.13) | 0.258 |  |  |  |  |
| **Milk + Sweetener** |  |  |  |  | -0.36 (-0.71, -0.01) | 0.046 |  |  |  |  |
| **Sugar** |  |  |  |  | 0.16 (-0.63, 0.95) | 0.688 |  |  |  |  |
| **Sweetener** |  |  |  |  | -0.05 (-0.54, 0.43) | 0.831 |  |  |  |  |
| **Binary** |  |  |  |  |  |  | -0.03 (-0.17, 0.10) | 0.617 |  |  |

**Supplementary Table 10 Multivariable linear regression analysis for the association of moderate/high coffee consumption and NT proBNP with sensitivity analysis excluding consumption of carbonated drinks.** For this analysis only subjects without simultaneous consumption of caffeinated drinks were included. Mild coffee consumption (<3 cups/d) served as the reference group. Adjustment was performed for: A) unadjusted; B) main cardiovascular risk factors; C) additional adjustment for additives (separately), D) additional adjustment for additives (binary), E) same adjustment as for group A, but only for subjects who consumed coffee without any additives. Abbreviations as in Table 1.

|  | | A)SBP | | B)SBP | | C)SBP | | D)SBP | | E)SBP | |
| --- | --- | --- | --- | --- | --- | --- | --- | --- | --- | --- | --- |
|  | | *Estimates* | *p* | *Estimates* | *p* | *Estimates* | *p* | *Estimates* | *p* | *Estimates* | *p* |
| Coffee consumption 3-4 cups/day | | -5.05 (-8.66, -1.43) | 0.006 | -3.70 (-7.21, -0.18) | 0.040 | -3.93 (-7.63, -0.23) | 0.037 | -4.17 (-7.88, -0.45) | 0.028 | -4.96 (-11.18, 1.26) | 0.117 |
| Coffee consumption >4 cups/day] | | -6.28 (-11.60, -0.96) | 0.021 | -4.79 (-10.02, 0.44) | 0.073 | -5.78 (-11.46 – -0.10) | 0.046 | -5.01 (-10.72, 0.69) | 0.085 | -7.78 (-15.96, 0.41) | 0.062 |
| Age | |  |  | 0.88 (0.69, 1.07) | <0.001 | 0.86 (0.65, 1.06) | <0.001 | 0.87 (0.67, 1.08) | <0.001 | 0.73 (0.38, 1.09) | <0.001 |
| Female | |  |  | -4.80 (-7.97, -1.63) | 0.003 | -4.71 (-8.07, -1.34) | 0.006 | -4.84 (-8.21, -1.47) | 0.005 | -5.59 (-11.19, 0.01) | 0.050 |
| Diabetes mellitus | |  |  | 5.87 (0.37, 11.37) | 0.036 | 4.53 (-1.49, 10.56) | 0.140 | 4.37 (-1.69, 10.44) | 0.157 | 1.87 (-9.08 – 12.82) | 0.737 |
| Current smoking | |  |  | -1.21 (-5.14 – 2.72) | 0.545 | -1.87 (-6.09, 2.34) | 0.382 | -1.98 (-6.19, 2.23) | 0.357 | -0.01 (-7.13, 7.11) | 0.998 |
| BMI | |  |  | 0.42 (0.08, 0.76) | 0.016 | 0.46 (0.09 – 0.83) | 0.015 | 0.44 (0.07, 0.81) | 0.020 | 0.72 (0.14, 1.29) | 0.014 |
| Additives | **Milk** |  |  |  |  | 0.21 (-3.38, 3.80) | 0.909 |  |  |  |  |
| **Milk + Sugar** |  |  |  |  | -1.46 (-9.10, 6.18) | 0.708 |  |  |  |  |
| **Milk + Sweetener** |  |  |  |  | 0.57 (-8.54, 9.69) | 0.902 |  |  |  |  |
| **Sugar** |  |  |  |  | 15.78 (-4.64, 36.20) | 0.130 |  |  |  |  |
| **Sweetener** |  |  |  |  | 8.13 (-5.10, 21.35) | 0.228 |  |  |  |  |
| **Binary** |  |  |  |  |  |  | 0.35 (-3.11, 3.82) | 0.841 |  |  |

**Supplementary Table 11. Multivariable linear regression analysis for the association of moderate/high coffee consumption and systolic blood pressure with sensitivity analysis excluding consumption of carbonated drinks.** For this analysis only subjects without simultaneous consumption of caffeinated drinks were included. Mild coffee consumption (<3 cups/d) served as the reference group. Adjustment was performed for: A) unadjusted; B) main cardiovascular risk factors; C) additional adjustment for additives (separately), D) additional adjustment for additives (binary), E) same adjustment as for group A, but only for subjects who consumed coffee without any additives. Abbreviations as in Table 1.

|  | | A)DBP | | B)DBP | | C)DBP | | D)DBP | | E)DBP | | |
| --- | --- | --- | --- | --- | --- | --- | --- | --- | --- | --- | --- | --- |
|  | | *Estimates* | *p* | *Estimates* | *p* | *Estimates* | *p* | *Estimates* | *p* | *Estimates* | *p* |
| Coffee consumption 3-4 cups/day | | -1.64 (-3.47, 0.20) | 0.080 | -1.60 (-3.53, 0.33) | 0.105 | -1.82 (-3.83, 0.19) | 0.075 | -2.05 (-4.05, -0.05) | 0.045 | -1.79 (-5.22, 1.65) | 0.306 |
| Coffee consumption > 4 cups/day | | -2.35 (-5.05, 0.35) | 0.088 | -2.66 (-5.54, 0.22) | 0.070 | -3.80 (-6.89, -0.71) | 0.016 | -3.79 (-6.87, -0.71) | 0.016 | -4.82 (-9.37, -0.27) | 0.038 |
| Age | |  |  | 0.05 (-0.05, 0.16) | 0.313 | 0.02 (-0.09, 0.13) | 0.692 | 0.04 (-0.07, 0.15) | 0.487 | 0.01 (-0.18, 0.21) | 0.911 |
| Female | |  |  | -3.18 (-4.92, -1.44) | <0.001 | -3.48 (-5.31, -1.65) | <0.001 | -3.45 (-5.27, -1.64) | <0.001 | -4.31 (-7.41, -1.21) | 0.007 |
| Diabetes mellitus | |  |  | -0.37 (-3.39, 2.65) | 0.809 | -0.44 (-3.72, 2.83) | 0.790 | -0.42 (-3.69, 2.85) | 0.801 | -0.64 (-6.70, 5.42) | 0.836 |
| Current smoking | |  |  | -1.43 (-3.60, 0.73) | 0.193 | -1.35 (-3.64, 0.94) | 0.248 | -1.39 (-3.67, 0.88) | 0.230 | -2.99 (-6.94, 0.96) | 0.137 |
| BMI | |  |  | 0.29 (0.10, 0.47) | 0.003 | 0.27 (0.07, 0.47) | 0.008 | 0.24 (0.04, 0.44) | 0.016 | 0.23 (-0.08, 0.54) | 0.145 |
| Additives | **Milk** |  |  |  |  | 0.48 (-1.47, 2.43) | 0.630 |  |  |  |  |
| **Milk + Sugar** |  |  |  |  | -1.87 (-6.03, 2.28) | 0.377 |  |  |  |  |
| **Milk + Sweetener** |  |  |  |  | -0.37 (-5.33, 4.58) | 0.882 |  |  |  |  |
| **Sugar** |  |  |  |  | 2.89 (-8.22, 14.00) | 0.609 |  |  |  |  |
| **Sweetener** |  |  |  |  | 2.98 (-4.21, 10.18) | 0.416 |  |  |  |  |
| **Binary** |  |  |  |  |  |  | 0.22 (-1.65 – 2.09) | 0.819 |  |  |

**Supplementary Table 12. Multivariable linear regression analysis for the association of moderate/high coffee consumption and diastolic blood pressure with sensitivity analysis excluding consumption of carbonated drinks.** For this analysis only subjects without simultaneous consumption of caffeinated drinks were included. Mild coffee consumption (<3 cups/d) served as the reference group. Adjustment was performed for: A) unadjusted; B) main cardiovascular risk factors; C) additional adjustment for additives (separately), D) additional adjustment for additives (binary), E) same adjustment as for group A, but only for subjects who consumed coffee without any additives. Abbreviations as in Table 1.

|  | | A)Heart rate | | B)Heart rate | | C)Heart rate | | D)Heart rate | | E)Heart rate | | |
| --- | --- | --- | --- | --- | --- | --- | --- | --- | --- | --- | --- | --- |
|  | | *Estimates* | *p* | *Estimates* | *p* | *Estimates* | *p* | *Estimates* | *p* | *Estimates* | *p* |
| Coffee consumption 3-4 cups/day | | -0.36 (-2.13, 1.41) | 0.690 | -0.12 (-1.97, 1.72) | 0.896 | -0.16 (-2.12, 1.79) | 0.869 | -0.27 (-2.22, 1.69) | 0.789 | 0.82 (-2.70, 4.35) | 0.645 |
| Coffee consumption >4 cups/day | | -0.67 (-3.29, 1.96) | 0.617 | 0.01 (-2.74, 2.76) | 0.994 | 0.87 (-2.14, 3.88) | 0.572 | 0.43 (-2.58, 3.43) | 0.781 | 1.10 (-3.57, 5.77) | 0.643 |
| Age | |  |  | 0.07 (-0.04, 0.17) | 0.201 | 0.07 (-0.04, 0.19) | 0.197 | 0.08 (-0.03 – 0.19) | 0.173 | 0.04 (-0.17, 0.25) | 0.717 |
| Female | |  |  | 2.93 (1.26, 4.60) | 0.001 | 3.20 (1.41, 4.98) | <0.001 | 3.22 (1.44, 4.99) | <0.001 | 5.77 (2.59, 8.95) | <0.001 |
| Diabetes mellitus | |  |  | 1.07 (-1.79, 3.93) | 0.463 | 1.31 (-1.84, 4.46) | 0.414 | 1.66 (-1.48, 4.81) | 0.299 | 1.06 (-4.99, 7.10) | 0.731 |
| Arterial hypertension | |  |  | 0.07 (-1.89, 2.02) | 0.945 | 0.29 (-1.79, 2.37) | 0.783 | 0.12 (-1.94, 2.19) | 0.906 | 0.06 (-3.71, 3.83) | 0.976 |
| Current smoking | |  |  | 0.55 (-1.52, 2.62) | 0.601 | 0.62 (-1.61 – 2.85) | 0.586 | 1.09 (-1.12, 3.30) | 0.334 | 0.94 (-3.11, 4.99) | 0.649 |
| BMI | |  |  | 0.37 (0.18, 0.55) | <0.001 | 0.39 (0.19, 0.59) | <0.001 | 0.38 (0.18, 0.58) | <0.001 | 0.51 (0.17, 0.85) | 0.003 |
| Additives | **Milk** |  |  |  |  | 0.49 (-1.41, 2.38) | 0.612 |  |  |  |  |
| **Milk + Sugar** |  |  |  |  | 1.25 (-2.80, 5.30) | 0.544 |  |  |  |  |
| **Milk + Sweetener** |  |  |  |  | 4.29 (-0.54, 9.11) | 0.081 |  |  |  |  |
| **Sugar** |  |  |  |  | -4.74 (-15.54, 6.06) | 0.389 |  |  |  |  |
| **Sweetener** |  |  |  |  | 5.35 (-1.65, 12.35) | 0.134 |  |  |  |  |
| **Binary** |  |  |  |  |  |  | 0.66 (-1.16, 2.47) | 0.479 |  |  |

**Supplementary Table 13. Multivariable linear regression analysis for the association of moderate/high coffee consumption and heart rate with sensitivity analysis excluding consumption of carbonated drinks.** For this analysis only subjects without simultaneous consumption of caffeinated drinks were included. Mild coffee consumption (<3 cups/d) served as the reference group. Adjustment was performed for: A) unadjusted; B) main cardiovascular risk factors; C) additional adjustment for additives (separately), D) additional adjustment for additives (binary), E) same adjustment as for group A, but only for subjects who consumed coffee without any additives. Abbreviations as in Table 1.

|  | | A)PQ interval | | B)’PQ interval | | C)PQ interval | | D)PQ interval | | E)PQ interval | | |
| --- | --- | --- | --- | --- | --- | --- | --- | --- | --- | --- | --- | --- |
|  | | *Estimates* | *p* | *Estimates* | *p* | *Estimates* | *p* | *Estimates* | *p* | *Estimates* | *p* |
| Coffee consumption 3-4 cups/day | | -3.50 (-8.17, 1.17) | 0.141 | -1.20 (-6.19, 3.80) | 0.639 | -0.63 (-5.84, 4.58) | 0.811 | -0.85 (-6.10, 4.40) | 0.751 | -11.09 (-20.40, -1.77) | 0.020 |
| Coffee consumption >4 cups/day | | -1.86 (-8.81,5.09) | 0.600 | -0.75 (-8.29, 6.79) | 0.846 | 0.13 (-8.06, 8.32) | 0.975 | -0.22 (-8.50, 8.06) | 0.958 | -9.36 (-22.22, 3.50) | 0.153 |
| Age | |  |  | 0.67 (0.39, 0.95) | <0.001 | 0.64 (0.34, 0.94) | <0.001 | 0.65 (0.35, 0.95) | <0.001 | 1.16 (0.60 , 1.72) | <0.001 |
| Female | |  |  | -9.88 (-14.39, -5.37) | <0.001 | -9.63 (-14.39, -4.86) | <0.001 | -9.78 (-14.58, -4.99) | <0.001 | -17.08 (-25.54, -8.62) | <0.001 |
| Diabetes mellitus | |  |  | 2.40 (-5.17, 9.97) | 0.534 | -0.44 (-8.70, 7.83) | 0.917 | -0.64 (-8.99, 7.72) | 0.881 | 3.42 (-12.16, 19.01) | 0.665 |
| Arterial hypertension | |  |  | 1.22 (-4.07, 6.50) | 0.652 | 1.65 (-3.92, 7.22) | 0.560 | 1.44 (-4.15, 7.04) | 0.613 | -1.14 (-10.94, 8.67) | 0.819 |
| Current smoking | |  |  | -2.39 (-7.97, 3.19) | 0.400 | -1.38 (-7.35, 4.59) | 0.649 | -2.03 (-8.04, 3.98) | 0.507 | -2.98 (-13.52, 7.55) | 0.577 |
| BMI | |  |  | 0.34 (-0.17, 0.84) | 0.192 | 0.39 (-0.16, 0.93) | 0.163 | 0.34 (-0.21, 0.89) | 0.222 | 0.04 (-0.84, 0.92) | 0.924 |
| Additives | **Milk** |  |  |  |  | -2.13 (-7.20, 2.95) | 0.410 |  |  |  |  |
| **Milk + Sugar** |  |  |  |  | -0.05 (-10.84, 10.74) | 0.992 |  |  |  |  |
| **Milk + Sweetener** |  |  |  |  | -11.98 (-24.94, 0.97) | 0.070 |  |  |  |  |
| **Sugar** |  |  |  |  | -11.91 (-43.80, 19.99) | 0.464 |  |  |  |  |
| **Sweetener** |  |  |  |  | -18.23 (-36.15, -0.32) | 0.046 |  |  |  |  |
| **Binary** |  |  |  |  |  |  | -2.89 (-7.82, 2.05) | 0.251 |  |  |

**Supplementary Table 14. Multivariable linear regression analysis for the association of moderate/high coffee consumption and PQ-interval with sensitivity analysis excluding consumption of carbonated drinks.** For this analysis only subjects without simultaneous consumption of caffeinated drinks were included. Mild coffee consumption (<3 cups/d) served as the reference group. Adjustment was performed for: A) unadjusted; B) main cardiovascular risk factors; C) additional adjustment for additives (separately), D) additional adjustment for additives (binary), E) same adjustment as for group A, but only for subjects who consumed coffee without any additives. Abbreviations as in Table 1.

|  | | A)QRS interval | | B)QRS interval | | C)QRS interval | | D)QRS interval | | E)QRS interval | | |
| --- | --- | --- | --- | --- | --- | --- | --- | --- | --- | --- | --- | --- |
|  | | *Estimates* | *p* | *Estimates* | *p* | *Estimates* | *p* | *Estimates* | *p* | *Estimates* | *p* |
| Coffee consumption 3-4 cups/day | | -0.13 (-2.44, 2.19) | 0.915 | 0.11 (-2.26, 2.49) | 0.925 | 0.00 (-2.48, 2.48) | 0.997 | -0.01 (-2.49, 2.47) | 0.991 | 2.15 (-2.41, 6.70) | 0.354 |
| Coffee consumption >4 cups/day | | 0.33 (-3.17, 3.83) | 0.852 | -0.96 (-4.60, 2.68) | 0.605 | -1.43 (-5.39, 2.52) | 0.477 | -1.96 (-5.93, 2.00) | 0.331 | 1.43 (-5.05, 7.90) | 0.665 |
| Age | |  |  | 0.13 (-0.00, 0.27) | 0.055 | 0.09 (-0.06, 0.23) | 0.233 | 0.10 (-0.04, 0.24) | 0.161 | 0.18 (-0.09, 0.46) | 0.195 |
| Female | |  |  | -8.56 (-10.71, -6.40) | <0.001 | -9.33 (-11.61, -7.06) | <0.001 | -9.10 (-11.37, -6.83) | <0.001 | -12.05 (-16.25, -7.85) | <0.001 |
| Diabetes mellitus | |  |  | -2.76 (-6.39, 0.87) | 0.135 | -2.38 (-6.34, 1.57) | 0.237 | -1.94 (-5.90, 2.03) | 0.338 | -6.01 (-13.66, 1.64) | 0.123 |
| Arterial hypertension | |  |  | -0.22 (-2.76, 2.31) | 0.862 | -0.32 (-2.99, 2.35) | 0.813 | -0.34 (-3.00, 2.32) | 0.800 | -2.84 (-7.78, 2.11) | 0.259 |
| Current smoking | |  |  | -0.27 (-2.95, 2.41) | 0.843 | 0.21 (-2.65, 3.07) | 0.887 | 0.37 (-2.48, 3.23) | 0.797 | -3.20 (-8.51, 2.11) | 0.237 |
| BMI | |  |  | 0.21 (-0.03, 0.46) | 0.083 | 0.19 (-0.07, 0.45) | 0.154 | 0.17 (-0.09 0.43) | 0.198 | 0.03 (-0.40, 0.47) | 0.882 |
| Additives | **Milk** |  |  |  |  | -0.63 (-3.05, 1.79) | 0.611 |  |  |  |  |
| **Milk + Sugar** |  |  |  |  | -4.64 (-9.85, 0.58) | 0.081 |  |  |  |  |
| **Milk + Sweetener** |  |  |  |  | -3.25 (-9.51, 3.01) | 0.308 |  |  |  |  |
| **Sugar** |  |  |  |  | -7.57 (-23.00, 7.87) | 0.336 |  |  |  |  |
| **Sweetener** |  |  |  |  | 6.31 (-2.35, 14.98) | 0.153 |  |  |  |  |
| **Binary** |  |  |  |  |  |  | -1.10 (-3.43, 1.23) | 0.355 |  |  |

**Supplementary Table 15. Multivariable linear regression analysis for the association of moderate/high coffee consumption and QRS-interval with sensitivity analysis excluding consumption of carbonated drinks.** For this analysis only subjects without simultaneous consumption of caffeinated drinks were included. Mild coffee consumption (<3 cups/d) served as the reference group. Adjustment was performed for: A) unadjusted; B) main cardiovascular risk factors; C) additional adjustment for additives (separately), D) additional adjustment for additives (binary), E) same adjustment as for group A, but only for subjects who consumed coffee without any additives. Abbreviations as in Table 1.

|  | | A)Q Tc interval | | B)Q Tc interval | | C)Q Tc interval | | D)Q Tc interval | | E)Q Tc interval | | |
| --- | --- | --- | --- | --- | --- | --- | --- | --- | --- | --- | --- | --- |
|  | | *Estimates* | *p* | *Estimates* | *p* | *Estimates* | *p* | *Estimates* | *p* | *Estimates* | *p* |
| Coffee consumption 3-4 cups/day | | -9.11 (-13.63, -4.60) | <0.001 | -8.23 (-13.21, -3.24) | 0.001 | -9.01 (-14.33, -3.70) | 0.001 | -9.00 (-14.32, -3.68) | 0.001 | -8.49 (-19.66, 2.68) | 0.135 |
| Coffee consumption >4 cups/day | | -4.87 (-11.94, 2.21) | 0.177 | -4.47 (-12.33, 3.38) | 0.264 | -3.78 (-12.45, 4.90) | 0.393 | -5.05 (-13.78, 3.67) | 0.256 | -4.03 (-19.92, 11.86) | 0.617 |
| Age | |  |  | 0.16 (-0.12, 0.44) | 0.256 | 0.11 (-0.20, 0.41) | 0.481 | 0.10 (-0.21, 0.40) | 0.529 | -0.34 (-1.01, 0.33) | 0.317 |
| Female | |  |  | 4.73 (0.19, 9.27) | 0.041 | 4.29 (-0.61, 9.18) | 0.086 | 4.61 (-0.27, 9.50) | 0.064 | 7.72 (-2.51, 17.95) | 0.138 |
| Diabetes mellitus | |  |  | 1.81 (-5.94, 9.56) | 0.647 | 2.59 (-6.10, 11.28) | 0.558 | 3.35 (-5.37, 12.08) | 0.450 | 9.76 (-9.30, 28.82) | 0.314 |
| Arterial hypertension | |  |  | 0.65 (-4.66, 5.96) | 0.811 | 1.52 (-4.18, 7.21) | 0.601 | 1.41 (-4.27, 7.08) | 0.627 | 1.12 (-10.93, 13.16) | 0.855 |
| Current smoking | |  |  | 2.36 (-3.28, 8.01) | 0.411 | 2.90 (-3.25, 9.06) | 0.355 | 3.90 (-2.25, 10.05) | 0.214 | 3.91 (-8.77, 16.58) | 0.544 |
| BMI | |  |  | 0.54 (0.03, 1.05) | 0.036 | 0.57 (0.01, 1.13) | 0.045 | 0.59 (0.04, 1.15) | 0.037 | 0.67 (-0.37, 1.72) | 0.205 |
| Additives | **Milk** |  |  |  |  | 0.32 (-4.87, 5.50) | 0.905 |  |  |  |  |
| **Milk + Sugar** |  |  |  |  | 2.03 (-9.03, 13.10) | 0.718 |  |  |  |  |
| **Milk + Sweetener** |  |  |  |  | -0.55 (-14.23, 13.14) | 0.937 |  |  |  |  |
| **Sugar** |  |  |  |  | 3.80 (-28.37, 35.97) | 0.817 |  |  |  |  |
| **Sweetener** |  |  |  |  | 11.37 (-8.74, 31.48) | 0.267 |  |  |  |  |
| **Binary** |  |  |  |  |  |  | 0.55 (-4.46, 5.57) | 0.828 |  |  |

**Supplementary Table 16. Multivariable linear regression analysis for the association of moderate/high coffee consumption and QTc-interval with sensitivity analysis excluding consumption of carbonated drinks.** For this analysis only subjects without simultaneous consumption of caffeinated drinks were included. Mild coffee consumption (<3 cups/d) served as the reference group. Adjustment was performed for: A) unadjusted; B) main cardiovascular risk factors; C) additional adjustment for additives (separately), D) additional adjustment for additives (binary), E) same adjustment as for group A, but only for subjects who consumed coffee without any additives. Abbreviations as in Table 1.

|  | | A)LVEF | | B)LVEF | | C)LVEF | | D)LVEF | | E)LVEF | | |
| --- | --- | --- | --- | --- | --- | --- | --- | --- | --- | --- | --- | --- |
|  | | *Estimates* | *p* | *Estimates* | *p* | *Estimates* | *p* | *Estimates* | *p* | *Estimates* | *p* |
| Coffee consumption 3-4 cups/day | | 0.24 (-0.69, 1.18) | 0.609 | 0.33 (-0.69, 1.36) | 0.524 | 0.34 (-0.76, 1.43) | 0.546 | 0.33 (-0.77, 1.43) | 0.555 | 0.43 (-1.51, 2.36) | 0.662 |
| Coffee consumption >4 cups/day | | -0.30 (-1.68, 1.08) | 0.667 | -0.06 (-1.57, 1.45) | 0.939 | -0.68 (-2.34, 0.98) | 0.424 | -0.57 (-2.25, 1.10) | 0.503 | -1.76 (-4.41, 0.89) | 0.191 |
| Age | |  |  | 0.01 (-0.05, 0.06) | 0.844 | 0.01 (-0.06, 0.07) | 0.849 | 0.00 (-0.06, 0.07) | 0.895 | 0.06 (-0.06 – 0.17) | 0.327 |
| Female | |  |  | 2.09 (1.16, 3.01) | <0.001 | 1.81 (0.82, 2.81) | <0.001 | 1.80 (0.80, 2.80) | <0.001 | 1.46 (-0.26, 3.18) | 0.095 |
| Diabetes mellitus | |  |  | -2.25 (-3.98, -0.52) | 0.011 | -2.16 (-4.08, -0.24) | 0.028 | -2.21 (-4.14, -0.28) | 0.025 | -4.00 (-8.20, 0.19) | 0.061 |
| Arterial hypertension | |  |  | 0.18 (-0.88, 1.23) | 0.744 | 0.05 (-1.09, 1.19) | 0.933 | 0.04 (-1.09, 1.17) | 0.944 | 1.23 (-0.74, 3.20) | 0.221 |
| Current smoking | |  |  | 0.90 (-0.27 – 2.06) | 0.131 | 0.85 (-0.41, 2.11) | 0.187 | 0.80 (-0.46 – 2.06) | 0.213 | -0.22 (-2.62, 2.18) | 0.858 |
| BMI | |  |  | -0.09 (-0.20, 0.02) | 0.094 | -0.06 (-0.18, 0.06) | 0.308 | -0.05 (-0.17, 0.06) | 0.357 | -0.14 (-0.35, 0.07) | 0.195 |
| Additives | **Milk** |  |  |  |  | 0.20 (-0.86, 1.25) | 0.716 |  |  |  |  |
| **Milk + Sugar** |  |  |  |  | 0.81 (-1.60, 3.21) | 0.510 |  |  |  |  |
| **Milk + Sweetener** |  |  |  |  | 0.08 (-2.79, 2.96) | 0.955 |  |  |  |  |
| **Sugar** |  |  |  |  | -1.96 (-8.02, 4.11) | 0.526 |  |  |  |  |
| **Sweetener** |  |  |  |  | -0.48 (-4.84, 3.88) | 0.828 |  |  |  |  |
| **Binary** |  |  |  |  |  |  | 0.24 (-0.79 – 1.27) | 0.645 |  |  |

**Supplementary Table 17. Multivariable linear regression analysis for the association of moderate/high coffee consumption and LVEF with sensitivity analysis excluding consumption of carbonated drinks.** For this analysis only subjects without simultaneous consumption of caffeinated drinks were included. Mild coffee consumption (<3 cups/d) served as the reference group. Adjustment was performed for: A) unadjusted; B) main cardiovascular risk factors; C) additional adjustment for additives (separately), D) additional adjustment for additives (binary), E) same adjustment as for group A, but only for subjects who consumed coffee without any additives. Abbreviations as in Table 1.

|  | | A)LV mass index | | B)LV mass index | | C)LV mass index | | D)LV mass index | | E)LV mass index | |
| --- | --- | --- | --- | --- | --- | --- | --- | --- | --- | --- | --- |
|  | | *Estimates* | *p* | *Estimates* | *p* | *Estimates* | *p* | *Estimates* | *p* | *Estimates* | *p* |
| Coffee consumption 3-4 cups/day | | 1.15 (-2.78, 5.09) | 0.566 | 0.18 (-3.47, 3.83) | 0.922 | 0.50 (-3.35, 4.35) | 0.799 | 0.41 (-3.47, 4.30) | 0.835 | -0.04 (-6.59, 6.50) | 0.990 |
| Coffee consumption <4 cups/day | | 0.63 (-5.01, 6.27) | 0.826 | -2.46 (-7.77, 2.85) | 0.364 | -3.08 (-9.02, 2.87) | 0.310 | -3.90 (-9.85, 2.05) | 0.198 | -1.10 (-10.05, 7.86) | 0.809 |
| Age | |  |  | 0.30 (0.09, 0.51) | 0.004 | 0.31 (0.09, 0.53) | 0.006 | 0.30 (0.08, 0.52) | 0.007 | 0.26 (-0.14, 0.65) | 0.204 |
| Female | |  |  | -17.64 (-20.95, -14.32) | <0.001 | -17.45 (-21.00, -13.90) | <0.001 | -17.27 (-20.84, -13.71) | <0.001 | -19.35 (-25.22, -13.48) | <0.001 |
| Diabetes mellitus | |  |  | 2.10 (-3.85, 8.05) | 0.488 | 2.36 (-4.17, 8.89) | 0.478 | 1.57 (-5.04, 8.18) | 0.641 | 20.94 (7.08, 34.80) | 0.003 |
| Arterial hypertension | |  |  | 1.21 (-2.57, 4.99) | 0.530 | 0.64 (-3.41, 4.70) | 0.756 | 0.67 (-3.38, 4.71) | 0.746 | 1.01 (-5.71, 7.73) | 0.767 |
| Current smoking | |  |  | -0.33 (-4.47, 3.81) | 0.874 | 0.78 (-3.71, 5.26) | 0.733 | 0.37 (-4.14, 4.88) | 0.871 | 3.84 (-4.45, 12.13) | 0.361 |
| BMI | |  |  | 0.63 (0.25, 1.02) | 0.001 | 0.68 (0.27, 1.10) | 0.001 | 0.64 (0.22, 1.06) | 0.003 | 0.60 (-0.11, 1.32) | 0.098 |
| Additives | Milk |  |  |  |  | -2.29 (-6.02, 1.45) | 0.229 |  |  |  |  |
| Milk + Sugar |  |  |  |  | -3.20 (-12.03, 5.63) | 0.476 |  |  |  |  |
| Milk + Sweetener |  |  |  |  | -13.11 (-24.83, -1.40) | 0.028 |  |  |  |  |
| Sugar |  |  |  |  | -20.53 (-56.49, 15.43) | 0.262 |  |  |  |  |
| Sweetener |  |  |  |  | -10.06 (-22.36, 2.25) | 0.109 |  |  |  |  |
| Binary |  |  |  |  |  |  | -3.08 (-6.76, 0.59) | 0.100 |  |  |

**Supplementary Table 13. Multivariable linear regression analysis for the association of moderate/high coffee consumption and LV mass index with sensitivity analysis excluding consumption of carbonated drinks.** For this analysis only subjects without simultaneous consumption of caffeinated drinks were included. Mild coffee consumption (<3 cups/d) served as the reference group. Adjustment was performed for: A) unadjusted; B) main cardiovascular risk factors; C) additional adjustment for additives (separately), D) additional adjustment for additives (binary), E) same adjustment as for group A, but only for subjects who consumed coffee without any additives. Abbreviations as in Table 1.

|  | | A) E/e’ mean ratio | | B)E/e’ mean ratio | | C)E/e’ mean ratio | | D)E/e’ mean ratio | | E)E/e’ mean ratio | | |
| --- | --- | --- | --- | --- | --- | --- | --- | --- | --- | --- | --- | --- |
| *Predictors* | | *Estimates* | *p* | *Estimates* | *p* | *Estimates* | *p* | *Estimates* | *p* | *Estimates* | *p* |  |
| Coffee consumption 3-4 cups/day | | -0.36 (-0.75, 0.03) | 0.072 | -0.22 (-0.61, 0.17) | 0.263 | -0.27 (-0.69, 0.14) | 0.199 | -0.24 (-0.65, 0.18) | 0.265 | -0.37 (-1.13, 0.39) | 0.337 |  |
| Coffee consumption >4 cups/day | | -0.49 (-1.08, 0.10) | 0.103 | -0.41 (-1.01, 0.18) | 0.172 | -0.30 (-0.96, 0.36) | 0.374 | -0.27 (-0.93, 0.39) | 0.415 | -0.54 (-1.63, 0.55) | 0.327 |  |
| Age | |  |  | 0.06 (0.03, 0.08) | <0.001 | 0.06 (0.03, 0.08) | <0.001 | 0.06 (0.03, 0.08) | <0.001 | 0.06 (0.01, 0.10) | 0.017 |  |
| Female | |  |  | 0.71 (0.35, 1.07) | <0.001 | 0.79 (0.41, 1.18) | <0.001 | 0.81 (0.42, 1.19) | <0.001 | 1.05 (0.35, 1.74) | 0.003 |  |
| Diabetes mellitus | |  |  | 0.12 (-0.52, 0.75) | 0.717 | 0.05 (-0.66, 0.75) | 0.900 | 0.04 (-0.66, 0.75) | 0.905 | -0.01 (-1.47, 1.45) | 0.990 |  |
| Arterial hypertension | |  |  | 0.54 (0.12, 0.95) | 0.011 | 0.50 (0.06, 0.95) | 0.027 | 0.54 (0.10, 0.98) | 0.016 | 0.46 (-0.35, 1.26) | 0.264 |  |
| Current smoking | |  |  | 0.64 (0.20, 1.08) | 0.005 | 0.79 (0.31, 1.27) | 0.001 | 0.79 (0.31, 1.26) | 0.001 | 1.40 (0.46, 2.34) | 0.004 |  |
| BMI | |  |  | 0.06 (0.02, 0.10) | 0.003 | 0.06 (0.02, 0.11) | 0.004 | 0.07 (0.02, 0.11) | 0.002 | 0.16 (0.08, 0.24) | <0.001 |  |
| Additives | **Milk** |  |  |  |  | -0.17 (-0.58, 0.24) | 0.419 |  |  |  |  |  |
|  | **Milk + Sugar** |  |  |  |  | -0.11 (-1.02, 0.79) | 0.803 |  |  |  |  |  |
|  | **Milk + Sweetener** |  |  |  |  | 0.61 (-0.46, 1.69) | 0.261 |  |  |  |  |  |
|  | **Sugar** |  |  |  |  | 0.04 (-2.23, 2.31) | 0.975 |  |  |  |  |  |
|  | **Sweetener** |  |  |  |  | -0.45 (-2.09, 1.18) | 0.587 |  |  |  |  |  |
|  | **Binary** |  |  |  |  |  |  | -0.12 (-0.52, 0.27) | 0.546 |  |  |  |

**Supplementary Table 18. Multivariable linear regression analysis for the association of moderate/high coffee consumption and E/e’ mean ratio with sensitivity analysis excluding consumption of carbonated drinks.** For this analysis only subjects without simultaneous consumption of caffeinated drinks were included. Mild coffee consumption (<3 cups/d) served as the reference group. Adjustment was performed for: A) unadjusted; B) main cardiovascular risk factors; C) additional adjustment for additives (separately), D) additional adjustment for additives (binary), E) same adjustment as for group A, but only for subjects who consumed coffee without any additives. Abbreviations as in Table 1.

|  | | A)TR Vmax in m/s | | B)TR Vmax in m/s | | C)TR Vmax in m/s | | D)TR Vmax in m/s | | E)TR Vmax in m/s | |
| --- | --- | --- | --- | --- | --- | --- | --- | --- | --- | --- | --- |
|  | | *Estimates* | *p* | *Estimates* | *p* | *Estimates* | *p* | *Estimates* | *p* | *Estimates* | *p* |
| Coffee consumption 3-4 cups/day | | 0.02 (-0.05, 0.09) | 0.603 | 0.03 (-0.05, 0.10) | 0.465 | 0.04 (-0.04, 0.12) | 0.337 | 0.04 (-0.04, 0.12) | 0.309 | -0.06 (-0.23, 0.10) | 0.453 |
| Coffee consumption >4 cups/day | | 0.05 (-0.06, 0.17) | 0.361 | 0.04 (-0.08, 0.16) | 0.512 | 0.05 (-0.09, 0.18) | 0.496 | 0.05 (-0.08, 0.18) | 0.450 | 0.00 (-0.27, 0.27) | 0.993 |
| Age | |  |  | 0.00 (0.00, 0.01) | 0.045 | 0.00 (-0.00, 0.01) | 0.071 | 0.00 (-0.00, 0.01) | 0.058 | 0.01 (-0.01, 0.02) | 0.257 |
| Female | |  |  | -0.02 (-0.09, 0.05) | 0.523 | -0.01 (-0.09, 0.06) | 0.711 | -0.01 (-0.09, 0.06) | 0.732 | -0.03 (-0.18, 0.11) | 0.653 |
| Diabetes mellitus | |  |  | -0.07 (-0.19, 0.05) | 0.229 | -0.06 (-0.19, 0.06) | 0.318 | -0.07 (-0.20, 0.05) | 0.257 | -0.28 (-0.64, 0.08) | 0.130 |
| Arterial hypertension | |  |  | 0.10 (0.02, 0.18) | 0.017 | 0.10 (0.02, 0.19) | 0.021 | 0.10 (0.01, 0.18) | 0.024 | 0.14 (-0.04, 0.31) | 0.125 |
| Current smoking | |  |  | 0.11 (0.00, 0.22) | 0.047 | 0.09 (-0.05, 0.22) | 0.194 | 0.08 (-0.05, 0.21) | 0.209 | 0.05 (-0.35, 0.46) | 0.796 |
| BMI | |  |  | 0.00 (-0.00, 0.01) | 0.307 | 0.01 (-0.00, 0.01) | 0.220 | 0.01 (-0.00, 0.01) | 0.202 | 0.01 (-0.01, 0.03) | 0.273 |
| Additives | **Milk** |  |  |  |  | -0.08 (-0.16, -0.01) | 0.030 |  |  |  |  |
| **Milk + Sugar** |  |  |  |  | -0.11 (-0.30, 0.08) | 0.247 |  |  |  |  |
| **Milk + Sweetener** |  |  |  |  | -0.07 (-0.29, 0.15) | 0.545 |  |  |  |  |
| **Sweetener** |  |  |  |  | 0.36 (0.04, 0.68) | 0.029 |  |  |  |  |
| **Binary** |  |  |  |  |  |  | -0.08 (-0.16, -0.01) | 0.029 |  |  |

**Supplementary Table 19. Multivariable linear regression analysis for the association of moderate/high coffee consumption and TR Vmax with sensitivity analysis excluding consumption of carbonated drinks.** For this analysis only subjects without simultaneous consumption of caffeinated drinks were included. Mild coffee consumption (<3 cups/d) served as the reference group. Adjustment was performed for: A) unadjusted; B) main cardiovascular risk factors; C) additional adjustment for additives (separately), D) additional adjustment for additives (binary), E) same adjustment as for group A, but only for subjects who consumed coffee without any additives. Abbreviations as in Table 1.

|  | | A)TAPSE in mm | | B)TAPSE in mm | | C)TAPSE in mm | | D)TAPSE in mm | | E)TAPSE in mm | |
| --- | --- | --- | --- | --- | --- | --- | --- | --- | --- | --- | --- |
|  | | *Estimates* | *p* | *Estimates* | *p* | *Estimates* | *p* | *Estimates* | *p* | *Estimates* | *p* |
| Coffee consumption 3-4 cups/day | | 0.60 (-0.30, 1.51) | 0.192 | 0.44 (-0.57, 1.45) | 0.393 | 0.43 (-0.64, 1.50) | 0.428 | 0.38 (-0.69, 1.44) | 0.489 | -0.72 (-2.56, 1.11) | 0.437 |
| Coffee consumption >4 cups/day | | 1.69 (0.41, 2.97) | 0.010 | 1.06 (-0.37, 2.50) | 0.145 | 1.14 (-0.45, 2.73) | 0.159 | 1.12 (-0.47, 2.70) | 0.166 | 0.79 (-1.65, 3.24) | 0.520 |
| Age | |  |  | -0.12 (-0.18, -0.06) | <0.001 | -0.14 (-0.20, -0.08) | <0.001 | -0.14 (-0.20, -0.08) | <0.001 | -0.19 (-0.30, -0.09) | <0.001 |
| Female | |  |  | -0.52 (-1.43, 0.40) | 0.266 | -0.52 (-1.50, 0.45) | 0.293 | -0.52 (-1.50, 0.45) | 0.291 | -1.50 (-3.12, 0.12) | 0.069 |
| Diabetes mellitus | |  |  | -1.17 (-2.88, 0.55) | 0.181 | -0.52 (-2.48, 1.44) | 0.604 | -0.48 (-2.47, 1.50) | 0.634 | 1.01 (-2.63, 4.66) | 0.583 |
| Arterial hypertension | |  |  | 0.53 (-0.52, 1.59) | 0.318 | 0.41 (-0.71, 1.53) | 0.472 | 0.37 (-0.75, 1.48) | 0.517 | 0.51 (-1.36, 2.38) | 0.589 |
| Current smoking | |  |  | -0.27 (-1.44, 0.89) | 0.647 | -0.15 (-1.41, 1.12) | 0.817 | -0.19 (-1.45, 1.07) | 0.771 | -1.01 (-3.50, 1.48) | 0.424 |
| BMI | |  |  | 0.05 (-0.05, 0.16) | 0.311 | 0.10 (-0.02, 0.21) | 0.095 | 0.09 (-0.02, 0.20) | 0.114 | 0.03 (-0.17, 0.23) | 0.767 |
| Additives | **Milk** |  |  |  |  | -0.26 (-1.31, 0.79) | 0.627 |  |  |  |  |
| **Milk + Sugar** |  |  |  |  | -1.04 (-3.52, 1.43) | 0.406 |  |  |  |  |
| **Milk + Sweetener** |  |  |  |  | -1.27 (-4.39, 1.85) | 0.423 |  |  |  |  |
| **Sugar** |  |  |  |  | -0.05 (-5.23, 5.14) | 0.986 |  |  |  |  |
| **Sweetener** |  |  |  |  | -0.86 (-4.34, 2.62) | 0.628 |  |  |  |  |
| **Binary** |  |  |  |  |  |  | -0.35 (-1.37, 0.68) | 0.506 |  |  |

**Supplementary Table 20. Multivariable linear regression analysis for the association of moderate/high coffee consumption and TAPSE with sensitivity analysis excluding consumption of carbonated drinks.** For this analysis only subjects without simultaneous consumption of caffeinated drinks were included. Mild coffee consumption (<3 cups/d) served as the reference group. Adjustment was performed for: A) unadjusted; B) main cardiovascular risk factors; C) additional adjustment for additives (separately), D) additional adjustment for additives (binary), E) same adjustment as for group A, but only for subjects who consumed coffee without any additives. Abbreviations as in Table 1.

|  | | A)LBB | | B)LBB | | C)LBB | | D)LBB | | E)LBB | |
| --- | --- | --- | --- | --- | --- | --- | --- | --- | --- | --- | --- |
|  | | *Odds Ratios* | *p* | *Odds Ratios* | *p* | *Odds Ratios* | *p* | *Odds Ratios* | *p* | *Odds Ratios* | *p* |
| Coffee consumption3-4 cups/day | | 1.40 (0.57, 3.30) | 0.445 | 1.65 (0.62, 4.19) | 0.302 | 1.58 (0.54, 4.42) | 0.384 | 1.46 (0.50, 4.04) | 0.470 | 2.13 (0.44, 11.45) | 0.346 |
| Coffee consumption > 4 cups/day | | 2.83 (0.97, 7.42) | 0.042 | 2.09 (0.54, 6.78) | 0.243 | 2.11 (0.51, 7.40) | 0.264 | 1.95 (0.47, 6.83) | 0.316 | 4.53 (0.84, 26.55) | 0.076 |
| Age | |  |  | 1.05 (0.99, 1.12) | 0.088 | 1.03 (0.97, 1.10) | 0.353 | 1.03 (0.97, 1.10) | 0.289 | 1.00 (0.92, 1.10) | 0.922 |
| Female | |  |  | 0.44 (0.17, 1.03) | 0.065 | 0.28 (0.09, 0.75) | 0.018 | 0.29 (0.09, 0.77) | 0.019 | 0.28 (0.04, 1.17) | 0.114 |
| Diabetes mellitus | |  |  | 0.34 (0.05, 1.32) | 0.176 | 0.44 (0.06, 1.86) | 0.329 | 0.44 (0.06, 1.85) | 0.327 |  |  |
| Arterial hypertension | |  |  | 2.12 (0.66, 9.55) | 0.254 | 1.79 (0.52, 8.36) | 0.397 | 1.86 (0.55, 8.62) | 0.360 | 1.75 (0.32, 14.27) | 0.550 |
| Current smoking | |  |  | 1.26 (0.43, 3.27) | 0.648 | 1.62 (0.53, 4.38) | 0.363 | 1.44 (0.47, 3.89) | 0.495 | 1.72 (0.39 – 6.65) | 0.444 |
| BMI | |  |  | 1.07 (0.98, 1.16) | 0.127 | 1.08 (0.98, 1.17) | 0.118 | 1.07 (0.97, 1.17) | 0.157 | 1.03 (0.91, 1.14) | 0.630 |
| Additives | **Milk** |  |  |  |  | 0.70 (0.28, 1.79) | 0.457 |  |  |  |  |
| **Binary** |  |  |  |  |  |  | 0.55 (0.22, 1.38) | 0.200 |  |  |

**Supplementary Table 21. Multivariable logistic regression analysis for the association of moderate/high coffee consumption and LBBB excluding consumption of carbonated drinks.** For this analysis only subjects without simultaneous consumption of caffeinated drinks were included. Mild coffee consumption (<3 cups/d) served as the reference group. Adjustment was performed for: A) unadjusted; B) main cardiovascular risk factors; C) additional adjustment for additives (separately), D) additional adjustment for additives (binary), E) same adjustment as for group A, but only for subjects who consumed coffee without any additives. Abbreviations as in Table 1.

|  | | A)AV block | | B)AV block | | C)AV block | | D)AV block | | E)AV block | |
| --- | --- | --- | --- | --- | --- | --- | --- | --- | --- | --- | --- |
|  | | *Odds Ratios* | *p* | *Odds Ratios* | *p* | *Odds Ratios* | *p* | *Odds Ratios* | *p* | *Odds Ratios* | *p* |
| Coffee consumption 3-4 cups/day | | 0.50 (0.22, 1.01) | 0.069 | 0.62 (0.25, 1.36) | 0.259 | 0.67 (0.27, 1.50) | 0.346 | 0.66 (0.26, 1.47) | 0.328 | 0.30 (0.06, 1.19) | 0.111 |
| Coffee consumption > 4 cups/day | | 0.48 (0.11, 1.36) | 0.226 | 0.76 (0.17, 2.43) | 0.676 | 0.91 (0.20, 3.07) | 0.891 | 0.85 (0.18, 2.85) | 0.810 | 0.21 (0.01, 1.45) | 0.178 |
| Age | |  |  | 1.10 (1.05, 1.17) | <0.001 | 1.10 (1.04, 1.17) | 0.001 | 1.10 (1.04, 1.17) | 0.001 | 1.10 (1.01, 1.21) | 0.043 |
| Female | |  |  | 0.46 (0.23, 0.92) | 0.030 | 0.48 (0.23, 0.98) | 0.047 | 0.48 (0.23, 0.98) | 0.047 | 0.10 (0.01, 0.41) | 0.005 |
| Diabetes mellitus | |  |  | 1.79 (0.72, 4.16) | 0.193 | 1.38 (0.47, 3.58) | 0.532 | 1.35 (0.46, 3.50) | 0.552 | 1.07 (0.13, 6.42) | 0.944 |
| Arterial hypertension | |  |  | 0.83 (0.35, 2.11) | 0.675 | 0.76 (0.31, 1.99) | 0.557 | 0.81 (0.34, 2.08) | 0.637 | 0.25 (0.05, 1.09) | 0.066 |
| Current smoking | |  |  | 0.70 (0.23, 1.77) | 0.485 | 0.88 (0.28, 2.29) | 0.815 | 0.82 (0.26, 2.12) | 0.704 | 0.96 (0.13, 4.64) | 0.964 |
| BMI | |  |  | 1.00 (0.92, 1.08) | 0.953 | 1.00 (0.91, 1.08) | 0.951 | 0.99 (0.91, 1.08) | 0.897 | 1.09 (0.94, 1.24) | 0.212 |
| Additives | **Milk** |  |  |  |  | 0.81 (0.40, 1.69) | 0.569 |  |  |  |  |
| **Milk + Sugar** |  |  |  |  | 0.58 (0.03, 3.24) | 0.608 |  |  |  |  |
| **Binary** |  |  |  |  |  |  | 0.73 (0.36, 1.51) | 0.391 |  |  |

**Supplementary Table 22. Multivariable logistic regression analysis for the association of moderate/high coffee consumption and AV block excluding consumption of carbonated drinks.** For this analysis only subjects without simultaneous consumption of caffeinated drinks were included. Mild coffee consumption (<3 cups/d) served as the reference group. Adjustment was performed for: A) unadjusted; B) main cardiovascular risk factors; C) additional adjustment for additives (separately), D) additional adjustment for additives (binary), E) same adjustment as for group A, but only for subjects who consumed coffee without any additives. Abbreviations as in Table 1.

|  | | A)Atrial_fibrillation | | B)Atrial_fibrillation | | C)Atrial_fibrillation | | D)Atrial_fibrillation | | E)Atrial_fibrillation | |
| --- | --- | --- | --- | --- | --- | --- | --- | --- | --- | --- | --- |
|  | | *Odds Ratios* | *p* | *Odds Ratios* | *p* | *Odds Ratios* | *p* | *Odds Ratios* | *p* | *Odds Ratios* | *p* |
| Coffee consumption 3-4 cups/day | | 1.23 (0.68, 2.19) | 0.479 | 1.90 (0.94, 3.78) | 0.068 | 1.62 (0.78, 3.33) | 0.188 | 1.62 (0.78, 3.31) | 0.185 | 1.40 (0.46, 4.21) | 0.549 |
| Coffee consumption > 4 cups/day | | 0.71 (0.21, 1.87) | 0.538 | 0.77 (0.17, 2.55) | 0.700 | 0.54 (0.11, 1.95) | 0.397 | 0.64 (0.13, 2.22) | 0.522 | 0.58 (0.07, 2.98) | 0.551 |
| Age | |  |  | 1.13 (1.07, 1.19) | <0.001 | 1.12 (1.06, 1.19) | <0.001 | 1.12 (1.07, 1.19) | <0.001 | 1.15 (1.06, 1.27) | 0.001 |
| Female | |  |  | 0.37 (0.18, 0.72) | 0.004 | 0.37 (0.18, 0.73) | 0.006 | 0.37 (0.18, 0.73) | 0.005 | 0.19 (0.05, 0.59) | 0.007 |
| Diabetes mellitus | |  |  | 0.90 (0.36, 2.06) | 0.816 | 0.88 (0.33, 2.12) | 0.789 | 0.92 (0.35, 2.19) | 0.852 | 0.90 (0.19, 3.66) | 0.887 |
| Arterial hypertension | |  |  | 2.17 (0.80, 7.63) | 0.167 | 1.97 (0.71, 7.00) | 0.232 | 2.05 (0.74, 7.26) | 0.204 |  |  |
| Current smoking | |  |  | 0.95 (0.38, 2.15) | 0.911 | 0.92 (0.35, 2.18) | 0.851 | 1.06 (0.42, 2.45) | 0.898 | 0.73 (0.14, 2.83) | 0.667 |
| BMI | |  |  | 1.08 (1.01, 1.16) | 0.023 | 1.09 (1.01, 1.17) | 0.024 | 1.09 (1.01, 1.17) | 0.017 | 1.11 (1.00, 1.23) | 0.045 |
| Additives | **Milk** |  |  |  |  | 0.58 (0.29, 1.16) | 0.121 |  |  |  |  |
| **Milk + Sweetener** |  |  |  |  | 0.56 (0.03, 3.35) | 0.602 |  |  |  |  |
| **Sweetener** |  |  |  |  | 2.62 (0.32, 14.69) | 0.306 |  |  |  |  |
| **Binary** |  |  |  |  |  |  | 0.60 (0.31, 1.17) | 0.128 |  |  |

**Supplementary Table 23. Multivariable logistic regression analysis for the association of moderate/high coffee consumption and atrial fibrillation excluding consumption of carbonated drinks.** For this analysis only subjects without simultaneous consumption of caffeinated drinks were included. Mild coffee consumption (<3 cups/d) served as the reference group. Adjustment was performed for: A) unadjusted; B) main cardiovascular risk factors; C) additional adjustment for additives (separately), D) additional adjustment for additives (binary), E) same adjustment as for group A, but only for subjects who consumed coffee without any additives. Abbreviations as in Table 1.

|  | | A)Diabetes | | B)Diabetes | | C)Diabetes | | D)Diabetes | | E)Diabetes | | |
| --- | --- | --- | --- | --- | --- | --- | --- | --- | --- | --- | --- | --- |
|  | | *Odds Ratios* | *p* | *Odds Ratios* | *p* | *Odds Ratios* | *p* | *Odds Ratios* | *p* | *Odds Ratios* | *p* |
| Coffee consumption 3-4 cups/day | | 0.93 (0.51, 1.61) | 0.788 | 1.27 (0.66, 2.37) | 0.456 | 1.45 (0.73, 2.83) | 0.280 | 1.59 (0.80, 3.11) | 0.176 | 2.62 (0.78, 8.98) | 0.115 |
| Coffee consumption >4 cups/day | | 1.59 (0.75, 3.15) | 0.201 | 1.74 (0.73, 3.90) | 0.195 | 2.00 (0.74, 5.03) | 0.151 | 2.21 (0.83, 5.52) | 0.098 | 0.97 (0.12, 5.34) | 0.974 |
| Age | |  |  | 1.06 (1.02, 1.10) | 0.005 | 1.08 (1.03, 1.13) | 0.001 | 1.08 (1.03, 1.13) | 0.002 | 1.09 (1.00, 1.20) | 0.055 |
| Female | |  |  | 0.41 (0.23, 0.72) | 0.002 | 0.52 (0.28, 0.94) | 0.032 | 0.54 (0.29, 0.97) | 0.043 | 0.51 (0.15, 1.56) | 0.249 |
| Arterial hypertension | |  |  | 2.97 (1.22, 8.91) | 0.029 | 4.43 (1.52, 18.90) | 0.017 | 4.47 (1.53, 19.07) | 0.016 | 2.77 (0.45, 53.74) | 0.357 |
| Current smoking | |  |  | 1.17 (0.59, 2.24) | 0.638 | 1.26 (0.59, 2.54) | 0.536 | 1.16 (0.54, 2.38) | 0.684 | 0.29 (0.03, 1.53) | 0.199 |
| BMI | |  |  | 1.16 (1.10, 1.23) | <0.001 | 1.14 (1.08, 1.21) | <0.001 | 1.15 (1.08, 1.22) | <0.001 | 1.21 (1.09, 1.37) | 0.001 |
| Additives | **Milk** |  |  |  |  | 1.65 (0.85, 3.35) | 0.147 |  |  |  |  |
| **Milk + Sugar** |  |  |  |  | 1.52 (0.22, 6.45) | 0.610 |  |  |  |  |
| **Milk + Sweetener** |  |  |  |  | 3.07 (0.75, 10.56) | 0.090 |  |  |  |  |
| **Sweetener** |  |  |  |  | 1.79 (0.21, 10.07) | 0.543 |  |  |  |  |
| **Binary** |  |  |  |  |  |  | 1.73 (0.91, 3.43) | 0.102 |  |  |

**Supplementary Table 24. Multivariable logistic regression analysis for the association of moderate/high coffee consumption and diabetes mellitus excluding consumption of carbonated drinks.** For this analysis only subjects without simultaneous consumption of caffeinated drinks were included. Mild coffee consumption (<3 cups/d) served as the reference group. Adjustment was performed for: A) unadjusted; B) main cardiovascular risk factors; C) additional adjustment for additives (separately), D) additional adjustment for additives (binary), E) same adjustment as for group A, but only for subjects who consumed coffee without any additives. Abbreviations as in Table 1.

|  | | A)Obesity | | B)Obesity | | C)Obesity | | D)Obesity | | E)Obesity | |
| --- | --- | --- | --- | --- | --- | --- | --- | --- | --- | --- | --- |
|  | | *Odds Ratios* | *p* | *Odds Ratios* | *p* | *Odds Ratios* | *p* | *Odds Ratios* | *p* | *Odds Ratios* | *p* |
| Coffee consumption 3-4 cups/day | | 0.62 (0.39, 0.95) | 0.032 | 0.55 (0.33, 0.90) | 0.021 | 0.57 (0.33, 0.95) | 0.035 | 0.53 (0.30, 0.90) | 0.022 | 0.48 (0.17, 1.20) | 0.133 |
| Coffee consumption > 4 cups/day | | 1.15 (0.64, 1.97) | 0.634 | 1.07 (0.55, 2.02) | 0.832 | 0.99 (0.48, 1.97) | 0.987 | 0.99 (0.47, 1.97) | 0.976 | 1.40 (0.47, 3.93) | 0.533 |
| Age | |  |  | 0.97 (0.95, 1.00) | 0.044 | 0.97 (0.95, 1.00) | 0.058 | 0.98 (0.95, 1.00) | 0.101 | 0.99 (0.94, 1.04) | 0.681 |
| Female | |  |  | 1.30 (0.86, 1.99) | 0.220 | 1.20 (0.77, 1.88) | 0.428 | 1.20 (0.77, 1.89) | 0.422 | 1.53 (0.70, 3.39) | 0.289 |
| Diabetes mellitus | |  |  | 4.49 (2.57, 7.87) | <0.001 | 4.39 (2.37, 8.14) | <0.001 | 4.41 (2.37, 8.18) | <0.001 | 10.69 (3.44, 37.02) | <0.001 |
| Arterial hypertension | |  |  | 3.52 (2.01, 6.44) | <0.001 | 3.40 (1.88, 6.45) | <0.001 | 3.10 (1.72, 5.87) | <0.001 | 6.99 (2.16, 32.25) | 0.004 |
| Current smoking | |  |  | 1.49 (0.91, 2.41) | 0.108 | 1.58 (0.93, 2.64) | 0.084 | 1.61 (0.94, 2.71) | 0.074 | 1.56 (0.61, 3.82) | 0.335 |
| Additives | **Milk** |  |  |  |  | 0.74 (0.46, 1.19) | 0.217 |  |  |  |  |
| **Milk + Sugar** |  |  |  |  | 0.97 (0.33, 2.51) | 0.956 |  |  |  |  |
| **Milk + Sweetener** |  |  |  |  | 0.78 (0.22, 2.34) | 0.677 |  |  |  |  |
| **Sugar** |  |  |  |  | 1.38 (0.06, 12.16) | 0.789 |  |  |  |  |
| **Sweetener** |  |  |  |  | 2.85 (0.70, 10.91) | 0.127 |  |  |  |  |
| **Binary** |  |  |  |  |  |  | 0.76 (0.49, 1.21) | 0.246 |  |  |

**Supplementary Table 25. Multivariable logistic regression analysis for the association of moderate/high coffee consumption and obesity excluding consumption of carbonated drinks.** For this analysis only subjects without simultaneous consumption of caffeinated drinks were included. Mild coffee consumption (<3 cups/d) served as the reference group. Adjustment was performed for: A) unadjusted; B) main cardiovascular risk factors; C) additional adjustment for additives (separately), D) additional adjustment for additives (binary), E) same adjustment as for group A, but only for subjects who consumed coffee without any additives. Abbreviations as in Table 1.

|  | | A)CAD | | B)CAD | | C)CAD | | D)CAD | | ECAD | |
| --- | --- | --- | --- | --- | --- | --- | --- | --- | --- | --- | --- |
|  | | *Odds Ratios* | *p* | *Odds Ratios* | *p* | *Odds Ratios* | *p* | *Odds Ratios* | *p* | *Odds Ratios* | *p* |
| Coffee consumption 3-4 cups/day | | 0.55 (0.28, 1.00) | 0.059 | 0.73 (0.35, 1.46) | 0.385 | 0.66 (0.30, 1.38) | 0.286 | 0.69 (0.32, 1.42) | 0.330 | 0.61 (0.16, 2.12) | 0.456 |
| Coffee consumption > 4 cups/day | | 0.87 (0.35, 1.93) | 0.754 | 0.99 (0.32, 2.71) | 0.989 | 0.59 (0.15, 1.87) | 0.401 | 0.85 (0.24, 2.54) | 0.779 | 0.74 (0.12, 3.52) | 0.712 |
| Age | |  |  | 1.08 (1.03, 1.13) | 0.001 | 1.08 (1.03, 1.14) | 0.001 | 1.09 (1.04, 1.14) | 0.001 | 1.06 (0.98, 1.16) | 0.155 |
| Female | |  |  | 0.15 (0.06, 0.30) | <0.001 | 0.16 (0.07, 0.33) | <0.001 | 0.17 (0.07, 0.35) | <0.001 | 0.14 (0.03, 0.49) | 0.005 |
| Diabetes mellitus | |  |  | 1.81 (0.83, 3.87) | 0.131 | 1.33 (0.54, 3.15) | 0.529 | 1.29 (0.53, 2.99) | 0.560 | 5.03 (0.93, 29.66) | 0.064 |
| Arterial hypertension | |  |  | 3.98 (1.48, 13.90) | 0.013 | 3.25 (1.18, 11.47) | 0.037 | 3.57 (1.32, 12.49) | 0.023 | 5.67 (0.94, 109.91) | 0.115 |
| Current smoking | |  |  | 1.22 (0.55, 2.60) | 0.611 | 1.32 (0.55, 2.98) | 0.515 | 1.30 (0.56, 2.86) | 0.531 | 2.51 (0.67, 9.23) | 0.163 |
| BMI | |  |  | 0.98 (0.91, 1.05) | 0.638 | 0.99 (0.91, 1.07) | 0.796 | 1.00 (0.92, 1.07) | 0.930 | 0.91 (0.77, 1.03) | 0.185 |
| Additives | **Milk** |  |  |  |  | 0.85 (0.43, 1.70) | 0.635 |  |  |  |  |
| **Milk + Sugar** |  |  |  |  | 0.38 (0.02, 2.31) | 0.384 |  |  |  |  |
| **Milk + Sweetener** |  |  |  |  | 0.37 (0.02, 2.13) | 0.359 |  |  |  |  |
| **Sweetener** |  |  |  |  | 8.14 (1.10, 57.95) | 0.035 |  |  |  |  |
| **Binary** |  |  |  |  |  |  | 0.88 (0.46, 1.73) | 0.714 |  |  |

**Supplementary Table 26. Multivariable logistic regression analysis for the association of moderate/high coffee consumption and CAD excluding consumption of carbonated drinks.** For this analysis only subjects without simultaneous consumption of caffeinated drinks were included. Mild coffee consumption (<3 cups/d) served as the reference group. Adjustment was performed for: A) unadjusted; B) main cardiovascular risk factors; C) additional adjustment for additives (separately), D) additional adjustment for additives (binary), E) same adjustment as for group A, but only for subjects who consumed coffee without any additives. Abbreviations as in Table 1.

|  | | A)PAD | | B)PAD | | C)PAD | | D)PAD | | E)PAD | |
| --- | --- | --- | --- | --- | --- | --- | --- | --- | --- | --- | --- |
|  | | *Odds Ratios* | *p* | *Odds Ratios* | *p* | *Odds Ratios* | *p* | *Odds Ratios* | *p* | *Odds Ratios* | *p* |
| Coffee consumption 3-4 cups/day | | 1.09 (0.64, 1.81) | 0.754 | 1.36 (0.76, 2.40) | 0.293 | 1.41 (0.76, 2.59) | 0.268 | 1.41 (0.77, 2.59) | 0.263 | 3.45 (1.01, 12.81) | 0.053 |
| Coffee consumption > 4 cups/day | | 1.01 (0.45, 2.12) | 0.980 | 1.10 (0.44, 2.56) | 0.839 | 1.11 (0.39, 2.89) | 0.837 | 1.01 (0.36, 2.60) | 0.985 | 6.13 (1.19, 35.37) | 0.033 |
| Age | |  |  | 1.06 (1.03, 1.10) | <0.001 | 1.07 (1.03, 1.11) | <0.001 | 1.07 (1.03, 1.11) | <0.001 | 1.16 (1.07, 1.29) | 0.001 |
| Female | |  |  | 1.48 (0.88, 2.52) | 0.144 | 1.71 (0.97, 3.03) | 0.064 | 1.72 (0.98, 3.04) | 0.059 | 2.99 (0.99, 9.98) | 0.061 |
| Diabetes mellitus | |  |  | 1.41 (0.60, 3.20) | 0.418 | 1.22 (0.47, 3.03) | 0.678 | 1.20 (0.47, 2.98) | 0.695 | 0.43 (0.07, 2.37) | 0.347 |
| Arterial hypertension | |  |  | 1.28 (0.69, 2.44) | 0.444 | 1.31 (0.67, 2.62) | 0.437 | 1.27 (0.65, 2.52) | 0.490 | 0.48 (0.11, 1.93) | 0.301 |
| Current smoking | |  |  | 1.56 (0.81, 2.95) | 0.177 | 1.69 (0.83, 3.39) | 0.141 | 1.67 (0.83, 3.32) | 0.145 | 3.85 (0.92 – 16.79) | 0.065 |
| BMI | |  |  | 1.05 (0.99, 1.12) | 0.095 | 1.05 (0.98, 1.13) | 0.129 | 1.05 (0.98, 1.12) | 0.139 | 1.23 (1.08, 1.43) | 0.003 |
| Additives | **Milk** |  |  |  |  | 1.03 (0.57, 1.89) | 0.919 |  |  |  |  |
|  | **Milk + Sugar** |  |  |  |  | 1.40 (0.34, 4.92) | 0.612 |  |  |  |  |
|  | **Milk + Sweetener** |  |  |  |  | 0.67 (0.09, 2.93) | 0.627 |  |  |  |  |
|  | **Sweetener** |  |  |  |  | 0.55 (0.02 ,5.28) | 0.632 |  |  |  |  |
|  | **Binary** |  |  |  |  |  |  | 1.00 (0.57, 1.80) | 0.996 |  |  |

**Supplementary Table 27. Multivariable logistic regression analysis for the association of moderate/high coffee consumption and PAD excluding consumption of carbonated drinks.** For this analysis only subjects without simultaneous consumption of caffeinated drinks were included. Mild coffee consumption (<3 cups/d) served as the reference group. Adjustment was performed for: A) unadjusted; B) main cardiovascular risk factors; C) additional adjustment for additives (separately), D) additional adjustment for additives (binary), E) same adjustment as for group A, but only for subjects who consumed coffee without any additives. Abbreviations as in Table 1.

|  | | A)Heart failure | | B)Heart failure | | C)Heart failure | | D)Heart failure | | E)Heart failure | |
| --- | --- | --- | --- | --- | --- | --- | --- | --- | --- | --- | --- |
|  | | *Odds Ratios* | *p* | *Odds Ratios* | *p* | *Odds Ratios* | *p* | *Odds Ratios* | *p* | *Odds Ratios* | *p* |
| Coffee consumption 3-4 cups/day | | 0.35 (0.08, 1.06) | 0.096 | 0.35 (0.05, 1.37) | 0.184 | 0.39 (0.06, 1.53) | 0.231 | 0.32 (0.05, 1.27) | 0.154 | 0.85 (0.04, 8.48) | 0.895 |
| Coffee consumption >4 cups/day | | 1.33 (0.37, 3.79) | 0.619 | 1.95 (0.47, 6.79) | 0.317 | 2.00 (0.46, 7.52) | 0.322 | 1.25 (0.24, 5.06) | 0.766 | 2.64 (0.20, 27.81) | 0.420 |
| Age | |  |  | 1.10 (1.02, 1.21) | 0.025 | 1.10 (1.02, 1.22) | 0.030 | 1.10 (1.01, 1.21) | 0.040 | 1.27 (1.05, 1.66) | 0.037 |
| Female | |  |  | 0.53 (0.18, 1.41) | 0.211 | 0.68 (0.23, 1.88) | 0.459 | 0.65 (0.22, 1.81) | 0.411 | 1.39 (0.20, 9.81) | 0.726 |
| Diabetes mellitus | |  |  | 3.93 (1.20, 11.81) | 0.017 | 3.19 (0.87, 10.30) | 0.061 | 4.23 (1.18, 13.60) | 0.018 | 8.42 (0.33, 120.84) | 0.120 |
| Arterial hypertension | |  |  | 3.63 (0.65, 68.35) | 0.230 | 4.80 (0.81, 93.74) | 0.155 | 3.47 (0.61, 65.71) | 0.249 |  |  |
| Current smoking | |  |  | 1.81 (0.50, 5.83) | 0.336 | 1.51 (0.39, 5.11) | 0.526 | 2.42 (0.64, 8.32) | 0.167 | 15.27 (1.58, 224.84) | 0.025 |
| BMI | |  |  | 1.09 (0.98, 1.20) | 0.087 | 1.08 (0.95, 1.21) | 0.215 | 1.07 (0.95, 1.19) | 0.272 | 1.05 (0.88, 1.24) | 0.545 |
| Additives | **Milk** |  |  |  |  | 0.95 (0.30, 3.12) | 0.927 |  |  |  |  |
| **Milk + Sugar** |  |  |  |  | 4.45 (0.51, 29.11) | 0.132 |  |  |  |  |
| **Milk + Sweetener** |  |  |  |  | 1.43 (0.06, 11.61) | 0.772 |  |  |  |  |
| **Sweetener** |  |  |  |  | 6.06 (0.22, 90.94) | 0.208 |  |  |  |  |
| **Binary** |  |  |  |  |  |  | 1.03 (0.35, 3.25) | 0.960 |  |  |

**Supplementary Table 28. Multivariable logistic regression analysis for the association of moderate/high coffee consumption and heart failure excluding consumption of carbonated drinks.** For this analysis only subjects without simultaneous consumption of caffeinated drinks were included. Mild coffee consumption (<3 cups/d) served as the reference group. Adjustment was performed for: A) unadjusted; B) main cardiovascular risk factors; C) additional adjustment for additives (separately), D) additional adjustment for additives (binary), E) same adjustment as for group A, but only for subjects who consumed coffee without any additives. Abbreviations as in Table 1.

|  | | A)HF(m)rEF | | B)HF(m)rEF | | C)HF(m)rEF | | D)HF(m)rEF | |
| --- | --- | --- | --- | --- | --- | --- | --- | --- | --- |
|  | | *Odds Ratios* | *p* | *Odds Ratios* | *p* | *Odds Ratios* | *p* | *Odds Ratios* | *p* |
| Coffee consumption 3-4 cups/day | | 0.51 (0.12, 1.67) | 0.313 | 0.48 (0.07, 2.00) | 0.368 | 0.54 (0.08, 2.33) | 0.457 | 0.45 (0.06, 1.88) | 0.322 |
| Coffee consumption > 4 cups/day | | 1.43 (0.32, 4.73) | 0.594 | 2.30 (0.45, 9.51) | 0.270 | 2.52 (0.44, 11.67) | 0.254 | 1.37 (0.18, 6.81) | 0.723 |
| Age | |  |  | 1.09 (1.00, 1.21) | 0.060 | 1.10 (1.01, 1.23) | 0.061 | 1.09 (1.00, 1.22) | 0.077 |
| Female | |  |  | 0.22 (0.05, 0.74) | 0.025 | 0.26 (0.06 – 0.92) | 0.054 | 0.26 (0.05, 0.89) | 0.048 |
| Diabetes mellitus | |  |  | 4.05 (1.06, 14.01) | 0.031 | 3.31 (0.75, 12.76) | 0.091 | 4.69 (1.10, 17.62) | 0.025 |
| Arterial hypertension | |  |  | 2.63 (0.44, 50.61) | 0.377 | 3.74 (0.58, 75.47) | 0.244 | 2.45 (0.40,47.62) | 0.418 |
| Current smoking | |  |  | 1.19 (0.23, 4.67) | 0.818 | 0.83 (0.14, 3.62) | 0.821 | 1.68 (0.32, 7.16) | 0.499 |
| BMI | |  |  | 1.07 (0.95, 1.21) | 0.249 | 1.04 (0.89, 1.20) | 0.638 | 1.02 (0.88, 1.18) | 0.746 |
| Additives | **Milk** |  |  |  |  | 1.09 (0.29, 4.58) | 0.895 |  |  |
| **Milk + Sugar** |  |  |  |  | 7.27 (0.74, 60.12) | 0.066 |  |  |
| **Milk + Sweetener** |  |  |  |  | 3.05 (0.14, 27.76) | 0.367 |  |  |
| **Sweetener** |  |  |  |  | 11.22 (0.37, 217.40) | 0.112 |  |  |
| **Binary** |  |  |  |  |  |  | 1.30 (0.38, 5.14) | 0.685 |

**Supplementary Table 29. Multivariable logistic regression analysis for the association of moderate/high coffee consumption and HF(m)rEF excluding consumption of carbonated drinks.** For this analysis only subjects without simultaneous consumption of caffeinated drinks were included. Mild coffee consumption (<3 cups/d) served as the reference group. Adjustment was performed for: A) unadjusted; B) main cardiovascular risk factors; C) additional adjustment for additives (separately), D) additional adjustment for additives (binary). Abbreviations as in Table 1.

|  | COFFEE CONSUMPTION | | | | |  |
| --- | --- | --- | --- | --- | --- | --- |
|  | **Overall** | **Not daily** | **1-2 cups/day** | **3-4 cups/day** | ≥ 4 cups/day | **p-value** |
| N (%) | 9009 | 1766 | 3933 | 2333 | 977 |  |
| DEMOGRAPHICS + BIOLOGICAL DATA | | | | | | |
| Age | 63.0 [55.0, 69.0] | 65.0 [56.0, 70.0] | 64.0 [57.0, 70.0] | 60.0 [54.0, 67.0] | 59.0 [53.0, 66.0] | <0.001 |
| Female | 4610 (51.2) | 868 (49.2) | 2231 (56.7) | 1148 (49.2) | 363 (37.2) | <0.001 |
| BMI kg/m2 | 26.1 [23.5, 29.1] | 26.0 [23.5, 29.1] | 26.0 [23.4, 29.1] | 26.0 [23.5, 29.1] | 26.7 [24.2, 29.5] | <0.001 |
| Obesity BMI >30 kg/m2 | 1694 (19.9) | 335 (20.0) | 712 (19.2) | 445 (20.1) | 202 (22.0) |  |
| Smoking current | 1731 (19.3) | 221 (12.6) | 606 (15.5) | 558 (24.0) | 346 (35.5) | <0.001 |
| CARDIOVASCULAR DISEASES | | | | | | |
| Arterial hypertension | 5637 (65.6) | 1133 (66.8) | 2599 (68.9) | 1346 (61.0) | 559 (60.8) | <0.001 |
| Diabetes mellitus | 694 ( 8.4) | 175 (10.7) | 303 ( 8.3) | 146 ( 6.8) | 70 ( 7.8) | <0.001 |
| Myocardial infarction | 266 ( 3.0) | 53 ( 3.0) | 123 ( 3.1) | 56 ( 2.4) | 34 ( 3.5) | 0.273 |
| CAD | 582 ( 8.7) | 134 (10.1) | 261 ( 9.1) | 125 ( 7.1) | 62 ( 8.4) | 0.023 |
| PAD | 827 (19.7) | 159 (19.3) | 369 (20.3) | 213 (19.3) | 86 (19.1) | 0.862 |
| LABORATORIES | | | | | | |
| Cholesterol, mg/dl | 208.0 [181.0, 237.0] | 202.0 [176.0, 231.0] | 211.0 [183.0, 239.0] | 208.0 [182.0, 237.0] | 207.0 [182.0, 237.0] | 0.947 |
| LDL, mg/dl | 121.0 [96.0, 146.0] | 116.0 [92.8, 141.0] | 121.0 [96.0, 147.0] | 122.0 [97.0, 146.0] | 124.0 [100.5, 149.0] | <0.001 |
| HDL, mg/dl | 62.0 [50.0, 76.0] | 61.0 [50.0, 75.0] | 64.0 [52.0, 79.0] | 63.0 [50.0, 76.0] | 57.0 [47.0, 70.0] | <0.001 |
| NT-proBNP, g/dl | 80.0 [44.0, 145.0] | 89.0 [49.0, 165.0] | 88.0 [49.0, 156.0] | 70.0 [38.0, 126.0] | 62.0 [34.0, 116.0] | <0.001 |
| Hemoglobin, g/dl | 14.3 [13.6, 15.1] | 14.3 [13.5, 15.1] | 14.2 [13.5, 15.0] | 14.3 [13.6, 15.1] | 14.6 [13.9, 15.3] | <0.001 |
| MEDICATION | | | | | | |
| ACEi/ ARBs | 1820 (21.2) | 383 (22.5) | 824 (21.9) | 434 (19.6) | 179 (19.4) | 0.049 |
| Beta blockers | 1475 (17.2) | 327 (19.2) | 707 (18.8) | 316 (14.3) | 125 (13.5) | <0.001 |
| Diuretics | 173 ( 2.0) | 43 ( 2.5) | 79 ( 2.1) | 35 ( 1.6) | 16 ( 1.7) | 0.182 |
| Lipid modifying drugs | 1542 (17.9) | 321 (18.9) | 738 (19.6) | 340 (15.4) | 143 (15.5) | <0.001 |
| ADDITIVES | | | | | | |
| Milk | 5966 (69.8) | 980 (73.2) | 2823 (72.1) | 1549 (66.7) | 614 (63.0) | <0.001 |
| Sugar | 1126 (13.2) | 263 (19.7) | 475 (12.1) | 281 (12.1) | 107 (11.0) | <0.001 |
| Honey | 76 ( 0.9) | 20 ( 1.5) | 40 ( 1.0) | 15 ( 0.6) | 1 ( 0.1) | 0.002 |
| Sweetener | 419 ( 4.9) | 72 ( 5.4) | 189 ( 4.8) | 104 ( 4.5) | 54 ( 5.5) | 0.486 |
| No additives | 2842 (33.2) | 368 (27.5) | 1198 (30.6) | 855 (36.8) | 421 (43.2) | <0.001 |
| BLACK/GREEN TEA | | | | | | |
| Never | 1480 (16.6) | 216 (12.3) | 656 (16.8) | 423 (18.3) | 185 (19.1) | <0.001 |
| 1-3/week | 4150 (46.4) | 502 (28.6) | 1824 (46.7) | 1261 (54.5) | 563 (58.0) | <0.001 |
| ≥4/week | 3311 (37.0) | 1035 (59.0) | 1422 (36.4) | 631 (27.3) | 223 (23.0) | <0.001 |
| CARBONATED DRINKS | | | | | | |
| Never | 4338 (48.5) | 896 (51.2) | 1990 (50.9) | 1060 (45.7) | 392 (40.6) | <0.001 |
| 1-3/week | 4031 (45.1) | 724 (41.3) | 1715 (43.9) | 1098 (47.3) | 494 (51.1) | <0.001 |
| ≥4/week | 576 ( 6.4) | 131 ( 7.5) | 202 ( 5.2) | 163 ( 7.0) | 80 ( 8.3) | <0.001 |

**Supplementary Table 30. Baseline characteristics** **of the study population with “not daily” as reference group.** Continuous variables are presented as median and interquartile range, and categorical variables are presented as absolute numbers and percentages. Abbreviations as in Table 1.

|  | | A)Total cholesterol | | B)Total cholesterol | | C)Total cholesterol | | D)Total cholesterol | | E)Total cholesterol | | F)Total cholesterol | |
| --- | --- | --- | --- | --- | --- | --- | --- | --- | --- | --- | --- | --- | --- |
|  | | *Estimates* | *p* | *Estimates* | *p* | *Estimates* | *p* | *Estimates* | *p* | *Estimates* | *p* | *Estimates* | *p* |
| Coffee consumption 1-2 cups/day | | 7.57 (5.22 – 9.93) | **<0.001** | 4.82 (2.41 – 7.24) | **<0.001** | 4.31 (1.56 – 7.06) | **0.002** | 4.13 (1.38 – 6.89) | **0.003** | 5.09 (2.79 – 7.39) | **<0.001** | 3.95 (-1.67 – 9.57) | 0.168 |
| Coffee consumption 3-4 cups/day | | 5.59 (3.00 – 8.19) | **<0.001** | 4.97 (2.29 – 7.66) | **<0.001** | 4.41 (1.38 – 7.44) | **0.004** | 4.18 (1.14 – 7.21) | **0.007** | 4.76 (2.20 – 7.32) | **<0.001** | 4.58 (-1.38 – 10.54) | 0.132 |
| Coffee consumption > 4 cups/day | | 5.43 (2.15 – 8.70) | **0.001** | 8.09 (4.65 – 11.54) | **<0.001** | 8.13 (4.32 – 11.94) | **<0.001** | 7.86 (4.04 – 11.68) | **<0.001** | 7.77 (4.49 – 11.06) | **<0.001** | 5.05 (-1.89 – 11.98) | 0.154 |
| Age | |  |  | 0.11 (-0.00 – 0.23) | 0.055 | 0.10 (-0.02 – 0.23) | 0.104 | 0.10 (-0.02 – 0.22) | 0.113 | 0.41 (0.30 – 0.52) | **<0.001** | 0.09 (-0.15 – 0.33) | 0.469 |
| Female | |  |  | 20.44 (18.62 – 22.26) | **<0.001** | 21.29 (19.34 – 23.23) | **<0.001** | 21.22 (19.28 – 23.16) | **<0.001** | 18.61 (16.87 – 20.35) | **<0.001** | 24.63 (20.95 – 28.32) | **<0.001** |
| Diabetes mellitus | |  |  | -20.33 (-23.73 – -16.92) | **<0.001** | -20.42 (-24.06 – -16.78) | **<0.001** | -20.70 (-24.35 – -17.05) | **<0.001** | -13.90 (-17.21 – -10.59) | **<0.001** | -23.35 (-30.10 – -16.61) | **<0.001** |
| Arterial hypertension | |  |  | 1.84 (-0.23 – 3.90) | 0.081 | 2.12 (-0.06 – 4.31) | 0.057 | 2.26 (0.07 – 4.46) | **0.043** | 4.64 (2.66 – 6.61) | **<0.001** | 3.71 (-0.48 – 7.89) | 0.083 |
| Current smoking | |  |  | -1.57 (-3.89 – 0.76) | 0.188 | -2.64 (-5.13 – -0.15) | **0.038** | -2.51 (-5.00 – -0.01) | **0.049** | -0.52 (-2.74 – 1.71) | 0.648 | -3.46 (-8.16 – 1.23) | 0.148 |
| BMI | |  |  | -0.25 (-0.46 – -0.05) | **0.016** | -0.28 (-0.50 – -0.06) | **0.013** | -0.29 (-0.51 – -0.07) | **0.010** | -0.09 (-0.29 – 0.11) | 0.366 | -0.35 (-0.77 – 0.06) | 0.092 |
| Additives | **Milk** |  |  |  |  | -2.18 (-4.36 – 0.01) | 0.051 |  |  |  |  |  |  |
|  | **Milk + Sugar** |  |  |  |  | -0.93 (-4.55 – 2.69) | 0.615 |  |  |  |  |  |  |
|  | **Milk + Sweetener** |  |  |  |  | -5.76 (-11.23 – -0.30) | **0.039** |  |  |  |  |  |  |
|  | **Sugar** |  |  |  |  | -0.43 (-8.12 – 7.25) | 0.912 |  |  |  |  |  |  |
|  | **Sweetener** |  |  |  |  | 4.43 (-4.40 – 13.26) | 0.325 |  |  |  |  |  |  |
|  | **Binary** |  |  |  |  |  |  | -2.11 (-4.22 – 0.00) | 0.050 |  |  |  |  |
| Lipid lowering drugs | |  |  |  |  |  |  |  |  | -33.54 (-35.93 – -31.14) | **<0.001** |  |  |

**Supplementary Table 31. Multivariable linear regression analysis for the association of milkd/moderate/high coffee consumption and total cholesterol.** “Not daily” coffee consumption (<1 cups/d) served as the reference group. Adjustment was performed for: A) unadjusted; B) main cardiovascular risk factors; C) additional adjustment for additives (separately), D) additional adjustment for additives (binary), E) additional adjustment for lipid lowering drugs, F) same adjustment as for group A, but only for subjects who consumed coffee without any additives. Abbreviations as in Table 1.

|  | | A)LDL | | B)LDL | | C)LDL | | D)LDL | | E)LDL | | F)LDL | |
| --- | --- | --- | --- | --- | --- | --- | --- | --- | --- | --- | --- | --- | --- |
|  | | *Estimates* | *p* | *Estimates* | *p* | *Estimates* | *p* | *Estimates* | *p* | *Estimates* | *p* | *Estimates* | *p* |
| Coffee consumption 1-2 cups/day | | 5.09 (2.98 – 7.21) | **<0.001** | 3.58 (1.37 – 5.80) | **0.002** | 3.54 (1.02 – 6.07) | **0.006** | 3.24 (0.71 – 5.77) | **0.012** | 3.83 (1.74 – 5.93) | **<0.001** | 4.03 (-1.11 – 9.17) | 0.124 |
| Coffee consumption 3-4 cups/day | | 5.55 (3.23 – 7.88) | **<0.001** | 4.55 (2.08 – 7.01) | **<0.001** | 4.48 (1.69 – 7.26) | **0.002** | 4.05 (1.26 – 6.84) | **0.004** | 4.31 (1.97 – 6.64) | **<0.001** | 5.51 (0.05 – 10.96) | **0.048** |
| Coffee consumption > 4 cups/day | | 8.11 (5.16 – 11.05) | **<0.001** | 8.15 (4.98 – 11.31) | **<0.001** | 8.74 (5.23 – 12.26) | **<0.001** | 8.34 (4.82 – 11.86) | **<0.001** | 7.75 (4.75 – 10.75) | **<0.001** | 7.79 (1.45 – 14.13) | **0.016** |
| Age | |  |  | 0.00 (-0.10 – 0.11) | 0.965 | 0.02 (-0.10 – 0.13) | 0.791 | 0.01 (-0.10 – 0.12) | 0.874 | 0.29 (0.19 – 0.39) | **<0.001** | 0.04 (-0.18 – 0.26) | 0.712 |
| Female | |  |  | 8.23 (6.56 – 9.90) | **<0.001** | 9.09 (7.31 – 10.88) | **<0.001** | 8.93 (7.15 – 10.71) | **<0.001** | 6.39 (4.80 – 7.97) | **<0.001** | 11.59 (8.23 – 14.95) | **<0.001** |
| Diabetes mellitus | |  |  | -21.82 (-25.00 – -18.65) | **<0.001** | -21.88 (-25.27 – -18.48) | **<0.001** | -22.13 (-25.54 – -18.72) | **<0.001** | -15.03 (-18.09 – -11.96) | **<0.001** | -25.74 (-31.99 – -19.49) | **<0.001** |
| Arterial hypertension | |  |  | 0.16 (-1.73 – 2.05) | 0.866 | 0.27 (-1.74 – 2.28) | 0.793 | 0.39 (-1.63 – 2.40) | 0.705 | 2.95 (1.15 – 4.75) | **0.001** | 1.48 (-2.34 – 5.29) | 0.448 |
| Current smoking | |  |  | 0.19 (-1.95 – 2.33) | 0.864 | -0.77 (-3.07 – 1.52) | 0.508 | -0.53 (-2.82 – 1.76) | 0.651 | 1.44 (-0.59 – 3.47) | 0.165 | -2.62 (-6.93 – 1.69) | 0.234 |
| BMI | |  |  | 0.35 (0.16 – 0.54) | **<0.001** | 0.34 (0.14 – 0.54) | **0.001** | 0.34 (0.14 – 0.54) | **0.001** | 0.50 (0.32 – 0.68) | **<0.001** | 0.33 (-0.05 – 0.70) | 0.089 |
| Additive | **Milk** |  |  |  |  | -0.34 (-2.35 – 1.67) | 0.739 |  |  |  |  |  |  |
|  | **Milk + Sugar** |  |  |  |  | 2.27 (-1.06 – 5.59) | 0.182 |  |  |  |  |  |  |
|  | **Milk + Sweetener** |  |  |  |  | -2.36 (-7.42 – 2.71) | 0.362 |  |  |  |  |  |  |
|  | **Sugar** |  |  |  |  | 2.14 (-4.92 – 9.21) | 0.552 |  |  |  |  |  |  |
|  | **Sweetener** |  |  |  |  | 3.37 (-4.76 – 11.49) | 0.416 |  |  |  |  |  |  |
|  | **Binary** |  |  |  |  |  |  | -0.01 (-1.94 – 1.93) | 0.994 |  |  |  |  |
| Lipid lowering drugs | |  |  |  |  |  |  |  |  | -32.87 (-35.06 – -30.68) | **<0.001** |  |  |

**Supplementary Table 32. Multivariable linear regression analysis for the association of mild/moderate/high coffee consumption and LDL including sensitivity analysis excluding consumption of carbonated drinks.** Not daily coffee consumption (<1 cup/d) served as the reference group. Adjustment was performed for: A) unadjusted; B) main cardiovascular risk factors; C) additional adjustment for additives (separately), D) additional adjustment for additives (binary), E) additional adjustment for lipid lowering drugs, F) same adjustment as for group A, but only for subjects who consumed coffee without any additives. . Abbreviations as in Table 1.

|  | | A)HDL | | B)HDL | | C)HDL | | D)HDL | | E)HDL | | F)HDL | |
| --- | --- | --- | --- | --- | --- | --- | --- | --- | --- | --- | --- | --- | --- |
|  | | *Estimates* | *p* | *Estimates* | *p* | *Estimates* | *p* | *Estimates* | *p* | *Estimates* | *p* | *Estimates* | *p* |
| Coffee consumption 1-2 cups/day | | 2.97 (1.88, 4.05) | **<0.001** | 1.55 (0.59, 2.50) | **0.001** | 0.84 (-0.25, 1.94) | 0.130 | 1.02 (-0.08, 2.12) | 0.068 | 1.54 (0.59, 2.49) | **0.001** | 0.03 (-2.25, 2.30) | 0.982 |
| Coffee consumption 3-4 cups/day | | 1.06 (-0.14, 2.25) | 0.082 | 1.91 (0.85, 2.97) | **<0.001** | 1.13 (-0.08, 2.33) | 0.066 | 1.33 (0.13, 2.54) | **0.030** | 1.95 (0.89, 3.00) | **<0.001** | -0.27 (-2.68, 2.14) | 0.824 |
| Coffee consumption >4 cups/day | | -3.58 (-5.09, -2.07) | **<0.001** | 0.93 (-0.43, 2.29) | 0.179 | -0.23 (-1.74, 1.29) | 0.770 | -0.07 (-1.59, 1.45) | 0.931 | 0.96 (-0.39, 2.32) | 0.162 | -0.25 (-3.06, 2.55) | 0.859 |
| Age | |  |  | 0.16 (0.11, 0.21) | **<0.001** | 0.14 (0.09, 0.18) | **<0.001** | 0.14 (0.09, 0.19) | **<0.001** | 0.18 (0.13, 0.22) | **<0.001** | 0.12 (0.02, 0.21) | **0.022** |
| Female | |  |  | 15.23 (14.51, 15.95) | **<0.001** | 15.17 (14.40, 15.95) | **<0.001** | 15.34 (14.57, 16.11) | **<0.001** | 15.17 (14.45, 15.89) | **<0.001** | 16.04 (14.55, 17.53) | **<0.001** |
| Diabetes mellitus | |  |  | -6.18 (-7.53, -4.84) | **<0.001** | -6.43 (-7.88, -4.99) | **<0.001** | -6.43 (-7.88, -4.98) | **<0.001** | -5.92 (-7.28, -4.55) | **<0.001** | -7.37 (-10.10, -4.64) | **<0.001** |
| Arterial hypertension | |  |  | -0.41 (-1.22, 0.40) | 0.324 | -0.22 (-1.09, 0.65) | 0.624 | -0.15 (-1.02, 0.72) | 0.737 | -0.28 (-1.09, 0.54) | 0.504 | -0.35 (-2.05, 1.34) | 0.682 |
| Current smoking | |  |  | -5.60 (-6.52, -4.68) | **<0.001** | -5.56 (-6.54, -4.57) | **<0.001** | -5.64 (-6.63 , -4.65) | **<0.001** | -5.61 (-6.53, -4.69) | **<0.001** | -5.67 (-7.57, -3.77) | **<0.001** |
| BMI | |  |  | -1.36 (-1.44, -1.28) | **<0.001** | -1.37 (-1.46, -1.28) | **<0.001** | -1.38 (-1.47, -1.29) | **<0.001** | -1.34 (-1.43, -1.26) | **<0.001** | -1.37 (-1.54, -1.20) | **<0.001** |
| Additives | **Milk** |  |  |  |  | -1.97 (-2.84, -1.11) | **<0.001** |  |  |  |  |  |  |
|  | **Milk + Sugar** |  |  |  |  | -4.21 (-5.65, -2.77) | **<0.001** |  |  |  |  |  |  |
|  | **Milk + Sweetener** |  |  |  |  | -4.26 (-6.43, -2.09) | **<0.001** |  |  |  |  |  |  |
|  | **Sugar** |  |  |  |  | -4.01 (-7.06, -0.95) | **0.010** |  |  |  |  |  |  |
|  | **Sweetener** |  |  |  |  | 0.66 (-2.85, 4.16) | 0.713 |  |  |  |  |  |  |
|  | **Binary** |  |  |  |  |  |  | -2.40 (-3.24, -1.56) | **<0.001** |  |  |  |  |
| Lipid lowering drugs | |  |  |  |  |  |  |  |  | -1.78 (-2.77, -0.79) | **<0.001** |  |  |

**Supplementary Table 33. Multivariable linear regression analysis for the association of mild/moderate/high coffee consumption and HDL including sensitivity analysis excluding consumption of carbonated drinks.** Not daily coffee consumption (<1 cup/d) served as the reference group. Adjustment was performed for: A) unadjusted; B) main cardiovascular risk factors; C) additional adjustment for additives (separately), D) additional adjustment for additives (binary), E) additional adjustment for lipid lowering drugs, F) same adjustment as for group A, but only for subjects who consumed coffee without any additives. . Abbreviations as in Table 1.

|  | | A)NT pro-BNP | | B)NT pro-BNP | | C)NT pro-BNP | | D)NT pro-BNP | | E)NT pro-BNP | |
| --- | --- | --- | --- | --- | --- | --- | --- | --- | --- | --- | --- |
|  | | *Estimates* | *p* | *Estimates* | *p* | *Estimates* | *p* | *Estimates* | *p* | *Estimates* | *p* |
| Coffee consumption1-2 cups/day | | -0.02 (-0.07, 0.03) | 0.462 | -0.07 (-0.12, -0.02) | **0.004** | -0.07 (-0.13, -0.01) | **0.018** | -0.06 (-0.12, -0.00) | **0.039** | -0.11 (-0.22, 0.01) | 0.075 |
| Coffee consumption 3-4 cups/day | | -0.24 (-0.30, -0.18) | **<0.001** | -0.11 (-0.17, -0.06) | **<0.001** | -0.12 (-0.18, -0.06) | **<0.001** | -0.11 (-0.17, -0.05) | **0.001** | -0.15 (-0.27, -0.02) | **0.019** |
| Coffee consumption > 4 cups/day | | -0.33 (-0.40, -0.26) | **<0.001** | -0.12 (-0.20, -0.05) | **0.001** | -0.14 (-0.22, -0.06) | **0.001** | -0.13 (-0.21, -0.05) | **0.001** | -0.21 (-0.35, -0.07) | **0.004** |
| Age | |  |  | 0.05 (0.04, 0.05) | **<0.001** | 0.05 (0.04, 0.05) | **<0.001** | 0.05 (0.04, 0.05) | **<0.001** | 0.05 (0.04, 0.05) | **<0.001** |
| Female | |  |  | 0.40 (0.36, 0.43) | **<0.001** | 0.38 (0.34, 0.42) | **<0.001** | 0.38 (0.34, 0.42) | **<0.001** | 0.37 (0.29, 0.45) | **<0.001** |
| Diabetes mellitus | |  |  | -0.03 (-0.10, 0.04) | 0.439 | -0.04 (-0.11, 0.04) | 0.325 | -0.03 (-0.11, 0.05) | 0.428 | 0.08 (-0.06, 0.22) | 0.267 |
| Arterial hypertension | |  |  | 0.17 (0.13, 0.21) | **<0.001** | 0.18 (0.13, 0.23) | **<0.001** | 0.18 (0.14, 0.23) | **<0.001** | 0.23 (0.14, 0.32) | **<0.001** |
| Current smoking | |  |  | 0.07 (0.02, 0.12) | **0.003** | 0.08 (0.03, 0.14) | **0.002** | 0.08 (0.03, 0.13) | **0.003** | 0.11 (0.01, 0.21) | **0.027** |
| BMI | |  |  | -0.01 (-0.01, -0.01) | **<0.001** | -0.01 (-0.02, -0.01) | **<0.001** | -0.01 (-0.02, -0.01) | **<0.001** | -0.01 (-0.02, -0.00) | **0.002** |
| Additives | **Milk** |  |  |  |  | -0.00 (-0.05, 0.04) | 0.926 |  |  |  |  |
|  | **Milk + Sugar** |  |  |  |  | -0.08 (-0.16, -0.01) | **0.033** |  |  |  |  |
|  | **Milk + Sweetener** |  |  |  |  | -0.08 (-0.19, 0.04) | 0.177 |  |  |  |  |
|  | **Sugar** |  |  |  |  | -0.08 (-0.24, 0.08) | 0.314 |  |  |  |  |
|  | **Sweetener** |  |  |  |  | -0.09 (-0.27, 0.09) | 0.337 |  |  |  |  |
|  | **Binary** |  |  |  |  |  |  | -0.02 (-0.06, 0.03) | 0.421 |  |  |

**Supplementary Table 34. Multivariable linear regression analysis for the association of moderate/high coffee consumption and NT proBNP.** Not daily coffee consumption (<1 cups/d) served as the reference group. Adjustment was performed for: A) unadjusted; B) main cardiovascular risk factors; C) additional adjustment for additives (separately), D) additional adjustment for additives (binary).. Abbreviations as in Table 1.

|  | | A)SBP | | B)SBP | | C)SBP | | D)SBP | | E)SBP | |
| --- | --- | --- | --- | --- | --- | --- | --- | --- | --- | --- | --- |
|  | | *Estimates* | *p* | *Estimates* | *p* | *Estimates* | *p* | *Estimates* | *p* | *Estimates* | *p* |
| Coffee consumption1-2 cups/day | | 1.24 (-0.03, 2.50) | 0.055 | 1.83 (0.62, 3.04) | **0.003** | 1.52 (0.14, 2.91) | **0.031** | 1.67 (0.28, 3.06) | **0.018** | 2.60 (-0.22, 5.42) | 0.071 |
| Coffee consumption3-4 cups/day | | -3.38 (-4.78, -1.99) | **<0.001** | -0.35 (-1.69, 1.00) | 0.611 | -0.82 (-2.34, 0.71) | 0.296 | -0.67 (-2.20, 0.86) | 0.391 | 0.17 (-2.82, 3.16) | 0.910 |
| Coffee consumption > 4 cups/day | | -3.92 (-5.70, -2.15) | **<0.001** | -1.38 (-3.11, 0.34) | 0.116 | -2.11 (-4.04, -0.19) | **0.031** | -1.82 (-3.75, 0.11) | 0.064 | -2.86 (-6.33, 0.62) | 0.107 |
| Age | |  |  | 0.83 (0.78, 0.89) | **<0.001** | 0.80 (0.74, 0.86) | **<0.001** | 0.81 (0.75, 0.86) | **<0.001** | 0.76 (0.64, 0.88) | **<0.001** |
| Female | |  |  | -5.12 (-6.03, -4.21) | **<0.001** | -5.21 (-6.19, -4.24) | **<0.001** | -5.13 (-6.11, -4.16) | **<0.001** | -4.97 (-6.81, -3.13) | **<0.001** |
| Diabetes mellitus | |  |  | 4.01 (2.30, 5.72) | **<0.001** | 4.10 (2.26, 5.95) | **<0.001** | 4.29 (2.43, 6.14) | **<0.001** | 2.23 (-1.19, 5.65) | 0.201 |
| Current smoking | |  |  | -1.67 (-2.83, -0.50) | **0.005** | -1.73 (-2.99, -0.48) | **0.007** | -1.90 (-3.16, -0.64) | **0.003** | -2.70 (-5.06, -0.34) | **0.025** |
| BMI | |  |  | 0.73 (0.63, 0.83) | **<0.001** | 0.67 (0.56, 0.78) | **<0.001** | 0.68 (0.57, 0.79) | **<0.001** | 0.72 (0.52, 0.91) | **<0.001** |
| Additives | **Milk** |  |  |  |  | -0.75 (-1.86, 0.35) | 0.180 |  |  |  |  |
|  | **Milk + Sugar** |  |  |  |  | -2.40 (-4.23, -0.58) | **0.010** |  |  |  |  |
|  | **Mik + Sweetener** |  |  |  |  | 0.88 (-1.85, 3.62) | 0.527 |  |  |  |  |
|  | **Sugar** |  |  |  |  | -1.80 (-5.66, 2.06) | 0.360 |  |  |  |  |
|  | **Sweetener** |  |  |  |  | 3.33 (-1.15, 7.82) | 0.145 |  |  |  |  |
|  | **Binary** |  |  |  |  |  |  | -0.84 (-1.90, 0.23) | 0.122 |  |  |

**Supplementary Table 35. Multivariable linear regression analysis for the association of moderate/high coffee consumption and systolic blood pressure.** Not daily coffee consumption (<1 cups/d) served as the reference group. Adjustment was performed for: A) unadjusted; B) main cardiovascular risk factors; C) additional adjustment for additives (separately), D) additional adjustment for additives (binary), E) same adjustment as for group A, but only for subjects who consumed coffee without any additives. Abbreviations as in Table 1.

|  | | A)DBP | | B)DBP | | C)DBP | | D)DBP | | E)DBP | |
| --- | --- | --- | --- | --- | --- | --- | --- | --- | --- | --- | --- |
|  | | *Estimates* | *p* | *Estimates* | *p* | *Estimates* | *p* | *Estimates* | *p* | *Estimates* | *p* |
| Coffee consumption 1-2 cups/day | | 0.78 (0.13, 1.44) | **0.019** | 1.15 (0.49, 1.82) | **0.001** | 0.90 (0.14, 1.66) | **0.021** | 0.93 (0.17, 1.69) | **0.017** | 0.67 (-0.88, 2.22) | 0.399 |
| Coffee consumption 3-4 cups/day | | -0.47 (-1.19, 0.25) | 0.198 | -0.01 (-0.75, 0.73) | 0.979 | -0.38 (-1.22, 0.46) | 0.376 | -0.36 (-1.20,  0.48) | 0.401 | -0.56 (-2.20, 1.08) | 0.504 |
| Coffee consumption > 4 cups/day | | -0.21 (-1.12, 0.71) | 0.655 | -0.49 (-1.44, 0.45) | 0.306 | -1.20 (-2.26, -0.15) | **0.025** | -1.16 (-2.22, -0.10) | **0.032** | -2.30 (-4.21, -0.39) | **0.018** |
| Age | |  |  | 0.06 (0.03, 0.09) | **<0.001** | 0.03 (0.00, 0.07) | **0.044** | 0.04 (0.00, 0.07) | **0.035** | -0.00 (-0.07, 0.06) | 0.933 |
| Female | |  |  | -3.03 (-3.52, -2.53) | **<0.001** | -3.14 (-3.67, -2.60) | **<0.001** | -3.11 (-3.65, -2.58) | **<0.001** | -3.32 (-4.32, -2.31) | **<0.001** |
| Diabetes mellitus | |  |  | -0.07 (-1.00, 0.87) | 0.890 | 0.11 (-0.90, 1.12) | 0.831 | 0.24 (-0.78, 1.26) | 0.645 | -0.72 (-2.59, 1.16) | 0.455 |
| Current smoking | |  |  | -0.93 (-1.57, -0.29) | **0.004** | -0.87 (-1.55, -0.18) | **0.014** | -0.91 (-1.60, -0.22) | **0.010** | -1.76 (-3.05, -0.46) | **0.008** |
| BMI | |  |  | 0.48 (0.42, 0.53) | **<0.001** | 0.44 (0.38, 0.50) | **<0.001** | 0.45 (0.39, 0.50) | **<0.001** | 0.39 (0.28, 0.49) | **<0.001** |
| Additives | **Milk** |  |  |  |  | -0.36 (-0.97, 0.24) | 0.238 |  |  |  |  |
|  | **Milk + Sugar** |  |  |  |  | -1.03 (-2.03, -0.02) | **0.045** |  |  |  |  |
|  | **Milk + Sweetener** |  |  |  |  | 0.09 (-1.41, -1.59) | 0.906 |  |  |  |  |
|  | **Sugar** |  |  |  |  | -0.53 (-2.65, 1.58) | 0.622 |  |  |  |  |
|  | **Sweetener** |  |  |  |  | 0.54 (-1.93, 3.00) | 0.670 |  |  |  |  |
|  | **Binary** |  |  |  |  |  |  | -0.41 (-0.99, 0.18) | 0.171 |  |  |

**Supplementary Table 36. Multivariable linear regression analysis for the association of mild/moderate/high coffee consumption and diastolic blood pressure.** Not daily coffee consumption (<1 cups/d) served as the reference group. Adjustment was performed for: A) unadjusted; B) main cardiovascular risk factors; C) additional adjustment for additives (separately), D) additional adjustment for additives (binary), E) same adjustment as for group A, but only for subjects who consumed coffee without any additives. Abbreviations as in Table 1.

|  | | A)Heart rate | | B)Heart rate | | C)Heart rate | | D)Heart rate | | E)Heart rate | |
| --- | --- | --- | --- | --- | --- | --- | --- | --- | --- | --- | --- |
|  | | *Estimates* | *p* | *Estimates* | *p* | *Estimates* | *p* | *Estimates* | *p* | *Estimates* | *p* |
| Coffee consumption 1-2 cups/day | | 0.57 (-0.07, 1.20) | 0.080 | 0.45 (-0.21, 1.12) | 0.181 | 0.37 (-0.40, 1.13) | 0.352 | 0.36 (-0.42, 1.13) | 0.366 | 0.45 (-1.13, 2.03) | 0.578 |
| Coffee consumption 3-4 cups/day | | -0.45 (-1.15, 0.25) | 0.210 | -0.17 (-0.91, 0.57) | 0.647 | -0.30 (-1.15, 0.54) | 0.482 | -0.36 (-1.20, 0.49) | 0.412 | 0.41 (-1.27, 2.08) | 0.634 |
| Coffee consumption > 4 cups/day | | 0.29 (-0.60, 1.18) | 0.522 | 0.32 (-0.63, 1.26) | 0.515 | 0.34 (-0.73, 1.41) | 0.536 | 0.25 (-0.82, 1.32) | 0.651 | 1.35 (-0.60, 3.30) | 0.175 |
| Age | |  |  | 0.03 (-0.00, 0.06) | 0.060 | 0.03 (-0.00, 0.07) | 0.054 | 0.03 (0.00, 0.07) | **0.048** | 0.03 (-0.04, 0.10) | 0.350 |
| Female | |  |  | 2.42 (1.92, 2.92) | **<0.001** | 2.49 (1.95, 3.04) | **<0.001** | 2.45 (1.91, 3.00) | **<0.001** | 3.13 (2.10, 4.17) | **<0.001** |
| Diabetes mellitus | |  |  | 3.53 (2.59, 4.46) | **<0.001** | 3.56 (2.55, 4.58) | **<0.001** | 3.59 (2.57, 4.61) | **<0.001** | 3.53 (1.64, 5.41) | **<0.001** |
| Arterial hypertension | |  |  | 1.45 (0.88, 2.01) | **<0.001** | 1.49 (0.87, 2.10) | **<0.001** | 1.44 (0.83, 2.06) | **<0.001** | 1.95 (0.78, 3.13) | **0.001** |
| Current smoking | |  |  | 1.31 (0.66, 1.95) | **<0.001** | 1.41 (0.71, 2.11) | **<0.001** | 1.46 (0.76, 2.16) | **<0.001** | 2.60 (1.28, 3.91) | **<0.001** |
| BMI | |  |  | 0.25 (0.19, 0.31) | **<0.001** | 0.25 (0.19,  0.32) | **<0.001** | 0.26 (0.19, 0.32) | **<0.001** | 0.24 (0.12, 0.35) | **<0.001** |
| Additives | **Milk** |  |  |  |  | -0.05 (-0.66, 0.57) | 0.881 |  |  |  |  |
|  | **Milk + Sugar** |  |  |  |  | 0.38 (-0.63, 1.39) | 0.463 |  |  |  |  |
|  | **Milk + Sweetener** |  |  |  |  | 1.16 (-0.36, 2.68) | 0.134 |  |  |  |  |
|  | **Sugar** |  |  |  |  | 0.96 (-1.18, 3.10) | 0.381 |  |  |  |  |
|  | **Sweetener** |  |  |  |  | -0.19 (-2.68, 2.30) | 0.883 |  |  |  |  |
|  | **Binary** |  |  |  |  |  |  | 0.08 (-0.51, 0.67) | 0.802 |  |  |

**Supplementary Table 37. Multivariable linear regression analysis for the association of mild/moderate/high coffee consumption and herat rate.** Not daily coffee consumption (<1 cups/d) served as the reference group. Adjustment was performed for: A) unadjusted; B) main cardiovascular risk factors; C) additional adjustment for additives (separately), D) additional adjustment for additives (binary), E) same adjustment as for group A, but only for subjects who consumed coffee without any additives. Abbreviations as in Table 1.

|  | | A)PQ interval | | B)PQ interval | | C)PQ interval | | D)PQ interval | | E)PQ interval | |
| --- | --- | --- | --- | --- | --- | --- | --- | --- | --- | --- | --- |
|  | | *Estimates* | *p* | *Estimates* | *p* | *Estimates* | *p* | *Estimates* | *p* | *Estimates* | *p* |
| Coffee consumption 1-2 cups/day | | -2.08 (-3.67, -0.49) | **0.011** | -0.99 (-2.63, 0.65) | 0.237 | -0.71 (-2.61, 1.20) | 0.467 | -0.61 (-2.52, 1.30) | 0.530 | 1.85 (-2.17, 5.87) | 0.367 |
| Coffee consumption 3-4 cups/day | | -3.15 (-4.90, -1.40) | **<0.001** | -0.78 (-2.60, 1.05) | 0.405 | -0.15 (-2.24, 1.94) | 0.887 | -0.04 (-2.13, 2.06) | 0.974 | 1.91 (-2.34, 6.16) | 0.379 |
| Coffee consumption >4 cups/day | | -2.96 (-5.17, -0.75) | **0.009** | -1.53 (-3.86, 0.80) | 0.199 | -1.29 (-3.91, 1.33) | 0.334 | -1.13 (-3.76, 1.50) | 0.398 | 0.58 (-4.37, 5.54) | 0.818 |
| Age | |  |  | 0.58 (0.50, 0.66) | **<0.001** | 0.60 (0.52, 0.69) | **<0.001** | 0.60 (0.52, 0.69) | **<0.001** | 0.72 (0.54, 0.89) | **<0.001** |
| Female | |  |  | -9.70 (-10.93, -8.46) | **<0.001** | -10.02 (-11.36, -8.68) | **<0.001** | -9.96 (-11.29, -8.62) | **<0.001** | -11.12 (-13.75, -8.49) | **<0.001** |
| Diabetes mellitus | |  |  | 1.44 (-0.88, 3.76) | 0.224 | 1.72 (-0.80, 4.24) | 0.182 | 1.59 (-0.95, 4.12) | 0.220 | 5.82 (1.03, 10.61) | **0.017** |
| Arterial hypertension | |  |  | 0.85 (-0.54, 2.25) | 0.229 | 0.92 (-0.58, 2.42) | 0.229 | 0.88 (-0.63, 2.39) | 0.252 | -0.18 (-3.13, 2.78) | 0.907 |
| Current smoking | |  |  | -2.61 (-4.18, -1.03) | **0.001** | -2.34 (-4.05, -0.63) | **0.007** | -2.50 (-4.21, -0.78) | **0.004** | -2.86 (-6.19, 0.48) | 0.093 |
| BMI | |  |  | 0.34 (0.20, 0.48) | **<0.001** | 0.33 (0.18, 0.48) | **<0.001** | 0.32 (0.17, 0.47) | **<0.001** | 0.20 (-0.09, 0.49) | 0.182 |
| Additives | **Milk** |  |  |  |  | 1.86 (0.35, 3.37) | **0.016** |  |  |  |  |
|  | **Milk + Sugar** |  |  |  |  | -0.17 (-2.67, 2.33) | 0.894 |  |  |  |  |
|  | **Milk + Sweetener** |  |  |  |  | -2.82 (-6.58, 0.93) | 0.141 |  |  |  |  |
|  | **Sugar** |  |  |  |  | 1.22 (-4.00, 6.43) | 0.647 |  |  |  |  |
|  | **Sweetener** |  |  |  |  | -1.49 (-7.60, 4.63) | 0.633 |  |  |  |  |
|  | **Binary** |  |  |  |  |  |  | 1.38 (-0.08, 2.84) | 0.063 |  |  |

**Supplementary Table 38. Multivariable linear regression analysis for the association of mild/moderate/high coffee consumption and PQ interval.** Not daily coffee consumption (<1 cups/d) served as the reference group. Adjustment was performed for: A) unadjusted; B) main cardiovascular risk factors; C) additional adjustment for additives (separately), D) additional adjustment for additives (binary), E) same adjustment as for group A, but only for subjects who consumed coffee without any additives. Abbreviations as in Table 1.

|  | | A)QRS interval | | B)QRS interval | | C)QRS interval | | D)QRS interval | | E)QRS interval | |
| --- | --- | --- | --- | --- | --- | --- | --- | --- | --- | --- | --- |
|  | | *Estimates* | *p* | *Estimates* | *p* | *Estimates* | *p* | *Estimates* | *p* | *Estimates* | *p* |
| Coffee consumption 1-2 cups/day | | -0.58 (-1.42, 0.26) | 0.177 | 0.05 (-0.81, 0.91) | 0.916 | 0.11 (-0.87, 1.09) | 0.821 | 0.09 (-0.89, 1.07) | 0.859 | 0.51 (-1.53, 2.56) | 0.622 |
| Coffee consumption 3-4 cups/day | | 0.17 (-0.76, 1.09) | 0.726 | 0.78 (-0.18, 1.74) | 0.110 | 0.88 (-0.20, 1.95) | 0.111 | 0.87 (-0.21, 1.95) | 0.115 | 1.42 (-0.74, 3.59) | 0.196 |
| Coffee consumption >4 cups/day | | -0.18 (-1.35, 1.00) | 0.770 | -0.70 (-1.93, 0.53) | 0.266 | -0.36 (-1.71, 1.00) | 0.603 | -0.37 (-1.72, 0.99) | 0.596 | 0.56 (-1.97, 3.09) | 0.666 |
| Age | |  |  | 0.12 (0.08, 0.16) | **<0.001** | 0.12 (0.08, 0.17) | **<0.001** | 0.12 (0.08, 0.17) | **<0.001** | 0.13 (0.04, 0.21) | **0.006** |
| Female | |  |  | -8.25 (-8.90, -7.61) | **<0.001** | -8.21 (-8.90, -7.51) | **<0.001** | -8.16 (-8.85, -7.47) | **<0.001** | -8.24 (-9.59, -6.90) | **<0.001** |
| Diabetes mellitus | |  |  | -0.75 (-1.96, 0.45) | 0.220 | -1.13 (-2.42, 0.15) | 0.085 | -1.18 (-2.46, 0.11) | 0.074 | -1.66 (-4.05, 0.74) | 0.175 |
| Arterial hypertension | |  |  | 1.08 (0.35, 1.82) | **0.004** | 0.87 (0.09, 1.64) | **0.029** | 0.84 (0.06, 1.62) | **0.034** | 0.96 (-0.57, 2.48) | 0.218 |
| Current smoking | |  |  | -1.07 (-1.90, -0.24) | **0.011** | -1.03 (-1.92, -0.15) | **0.022** | -1.10 (-1.99, -0.21) | **0.015** | -2.30 (-4.01, -0.59) | **0.009** |
| BMI | |  |  | 0.17 (0.10, 0.25) | **<0.001** | 0.16 (0.08, 0.23) | **<0.001** | 0.15 (0.07, 0.23) | **<0.001** | 0.20 (0.06, 0.35) | **0.007** |
| Additives | **Milk** |  |  |  |  | 0.41 (-0.37, 1.19) | 0.298 |  |  |  |  |
|  | **Milk + Sugar** |  |  |  |  | 0.19 (-1.10, 1.49) | 0.771 |  |  |  |  |
|  | **Milk + Sweetener** |  |  |  |  | -0.52 (-2.45, 1.42) | 0.602 |  |  |  |  |
|  | **Sugar** |  |  |  |  | -0.23 (-2.93, –2.48) | 0.870 |  |  |  |  |
|  | **Sweetener** |  |  |  |  | 0.64 (-2.46, 3.75) | 0.686 |  |  |  |  |
|  | **Binary** |  |  |  |  |  |  | 0.34 (-0.41, 1.09) | 0.375 |  |  |

**Supplementary Table 39. Multivariable linear regression analysis for the association of mild/moderate/high coffee consumption and QRS interval.** Not daily coffee consumption (<1 cups/d) served as the reference group. Adjustment was performed for: A) unadjusted; B) main cardiovascular risk factors; C) additional adjustment for additives (separately), D) additional adjustment for additives (binary), E) same adjustment as for group A, but only for subjects who consumed coffee without any additives. Abbreviations as in Table 1.

|  | | A)Q Tc interval | | B)Q Tc interval | | C)Q Tc interval | | D)Q Tc interval | | E)Q Tc interval | |
| --- | --- | --- | --- | --- | --- | --- | --- | --- | --- | --- | --- |
|  | | *Estimates* | *p* | *Estimates* | *p* | *Estimates* | *p* | *Estimates* | *p* | *Estimates* | *p* |
| Coffee consumption 1-2 cups/day | | -2.89 (-5.85, 0.06) | 0.055 | -3.07 (-6.33, 0.20) | 0.066 | -3.97 (-7.94, 0.01) | 0.051 | -4.11 (-8.10, -0.11) | **0.044** | -2.04 (-5.60, 1.53) | 0.263 |
| Coffee consumption 3-4 cups/day | | -5.82 (-9.07, -2.57) | **<0.001** | -4.73 (-8.36, -1.11) | **0.011** | -6.12 (-10.50, -1.75) | **0.006** | -6.24 (-10.62, -1.85) | **0.005** | -3.83 (-7.58, -0.07) | **0.046** |
| Coffee consumption > 4 cups/day | | -7.20 (-11.35, -3.06) | **0.001** | -5.89 (-10.56, -1.21) | **0.014** | -6.61 (-12.12, -1.09) | **0.019** | -6.85 (-12.39, -1.31) | **0.015** | -0.38 (-4.79, 4.03) | 0.865 |
| Age | |  |  | 0.35 (0.20, 0.51) | **<0.001** | 0.35 (0.17, 0.53) | **<0.001** | 0.34 (0.16, 0.52) | **<0.001** | 0.38 (0.23, 0.54) | **<0.001** |
| Female | |  |  | 5.19 (2.73, 7.66) | **<0.001** | 5.06 (2.26, 7.87) | **<0.001** | 5.09 (2.28, 7.90) | **<0.001** | 7.40 (5.07, 9.74) | **<0.001** |
| Diabetes mellitus | |  |  | 9.51 (4.92, 14.10) | **<0.001** | 10.18 (4.92, 15.45) | **<0.001** | 10.28 (4.99, 15.57) | **<0.001** | 7.67 (3.44, 11.90) | **<0.001** |
| Arterial hypertension | |  |  | 2.19 (-0.59, 4.98) | 0.122 | 1.78 (-1.36, 4.91) | 0.267 | 1.77 (-1.38, 4.93) | 0.271 | 2.42 (-0.22, 5.05) | 0.072 |
| Current smoking | |  |  | 0.57 (-2.58, 3.71) | 0.724 | 0.47 (-3.12, 4.06) | 0.799 | 0.43 (-3.17, 4.03) | 0.814 | 1.07 (-1.87, 4.02) | 0.475 |
| BMI | |  |  | 0.85 (0.57, 1.13) | **<0.001** | 0.89 (0.57, 1.20) | **<0.001** | 0.89 (0.57, 1.20) | **<0.001** | 0.37 (0.11, 0.63) | **0.005** |
| Additives | **Milk** |  |  |  |  | 1.70 (-1.47, 4.86) | 0.293 |  |  |  |  |
|  | **Milk + Sugar** |  |  |  |  | 1.75 (-3.48, 6.97) | 0.512 |  |  |  |  |
|  | **Milk + Sweetener** |  |  |  |  | -1.22 (-9.09, 6.66) | 0.762 |  |  |  |  |
|  | **Sugar** |  |  |  |  | 2.68 (-8.21, 13.58) | 0.629 |  |  |  |  |
|  | **Sweetener** |  |  |  |  | -4.70 (-17.59, 8.19) | 0.475 |  |  |  |  |
|  | **Binary** |  |  |  |  |  |  | 1.55 (-1.51, 4.61) | 0.322 |  |  |

**Supplementary Table 40. Multivariable linear regression analysis for the association of mild/moderate/high coffee consumption and QTc interval.** Not daily coffee consumption (<1 cups/d) served as the reference group. Adjustment was performed for: A) unadjusted; B) main cardiovascular risk factors; C) additional adjustment for additives (separately), D) additional adjustment for additives (binary), E) same adjustment as for group A, but only for subjects who consumed coffee without any additives. Abbreviations as in Table 1.

|  | | A)LVEF | | B)LVEF | | C)LVEF | | D)LVEF | | E)LVEF | |
| --- | --- | --- | --- | --- | --- | --- | --- | --- | --- | --- | --- |
|  | | *Estimates* | *p* | *Estimates* | *p* | *Estimates* | *p* | *Estimates* | *p* | *Estimates* | *p* |
| Coffee consumption 1-2 cups/day | | 0.22 (-0.12., 0.56) | 0.203 | 0.03 (-0.33, 0.39) | 0.852 | -0.08 (-0.49, 0.33) | 0.702 | -0.07 (-0.48, 0.34) | 0.738 | 0.31 (-0.50, 1.12) | 0.451 |
| Coffee consumption 3-4 cups/day | | 0.25 (-0.13, 0.62) | 0.199 | 0.22 (-0.18, 0.62) | 0.283 | 0.15 (-0.30, 0.60) | 0.519 | 0.15 (-0.31, 0.60) | 0.524 | 1.11 (0.25, 1.97) | **0.012** |
| Coffee consumption > 4 cups/day | | -0.03 (-0.50, 0.44) | 0.895 | 0.24 (-0.27, 0.75) | 0.353 | 0.07 (-0.50, 0.65) | 0.797 | 0.06 (-0.51, 0.64) | 0.826 | 0.32 (-0.68, 1.33) | 0.527 |
| Age | |  |  | -0.01 (-0.03, 0.01) | 0.280 | -0.01 (-0.03, 0.01) | 0.207 | -0.01 (-0.03, 0.01) | 0.235 | 0.00 (-0.03, 0.04) | 0.982 |
| Female | |  |  | 1.72 (1.45, 1.99) | **<0.001** | 1.70 (1.41, 1.99) | **<0.001** | 1.70 (1.41, 1.99) | **<0.001** | 1.58 (1.05, 2.10) | **<0.001** |
| Diabetes mellitus | |  |  | -0.79 (-1.30, -0.29) | **0.002** | -0.71 (-1.26, -0.17) | **0.011** | -0.70 (-1.25, -0.15) | **0.012** | -0.84 (-1.81, 0.13) | 0.088 |
| Arterial hypertension | |  |  | -0.39 (-0.69, -0.08) | **0.013** | -0.34 (-0.66, -0.01) | **0.041** | -0.32 (-0.64, 0.01) | 0.055 | -0.63 (-1.22, -0.04) | **0.037** |
| Current smoking | |  |  | -0.22 (-0.56, 0.13) | 0.222 | -0.24 (-0.61, 0.14) | 0.210 | -0.23 (-0.60, 0.15) | 0.230 | -0.33 (-1.02, 0.35) | 0.343 |
| BMI | |  |  | -0.12 (-0.15, -0.08) | **<0.001** | -0.12 (-0.15, -0.08) | **<0.001** | -0.12 (-0.15, -0.08) | **<0.001** | -0.11 (-0.17, -0.05) | **<0.001** |
| Additives | **Milk** |  |  |  |  | -0.04 (-0.37, 0.28) | 0.794 |  |  |  |  |
|  | **Milk + Sugar** |  |  |  |  | -0.02 (-0.57, 0.53) | 0.931 |  |  |  |  |
|  | **Milk + Sweetener** |  |  |  |  | 0.26 (-0.55, 1.06) | 0.530 |  |  |  |  |
|  | **Sugar** |  |  |  |  | -0.11 (-1.26, 1.04) | 0.851 |  |  |  |  |
|  | **Sweetener** |  |  |  |  | 0.02 (-1.32, 1.37) | 0.973 |  |  |  |  |
|  | **Binary** |  |  |  |  |  |  | -0.03 (-0.35, 0.29) | 0.859 |  |  |

**Supplementary Table 41. Multivariable linear regression analysis for the association of mild/moderate/high coffee consumption and LVEF** Not daily coffee consumption (<1 cups/d) served as the reference group. Adjustment was performed for: A) unadjusted; B) main cardiovascular risk factors; C) additional adjustment for additives (separately), D) additional adjustment for additives (binary), E) same adjustment as for group A, but only for subjects who consumed coffee without any additives. Abbreviations as in Table 1.

|  | | A)LV mass index | | B)LV mass index | | C)LV mass index | | D)LV mass index | | E)LV mass index | |
| --- | --- | --- | --- | --- | --- | --- | --- | --- | --- | --- | --- |
|  | | *Estimates* | *p* | *Estimates* | *p* | *Estimates* | *p* | *Estimates* | *p* | *Estimates* | *p* |
| Coffee consumption 1-2 cups/day | | -0.66 (-2.07, 0.74) | 0.354 | 0.27 (-1.05, 1.59) | 0.688 | -0.21 (-1.73, 1.30) | 0.783 | -0.14 (-1.65, 1.38) | 0.860 | -2.34 (-5.55, 0.88) | 0.154 |
| Coffee consumption 3-4 cups/day | | -0.41 (-1.96, 1.14) | 0.605 | 0.18 (-1.29, 1.66) | 0.808 | -0.33 (-2.00, 1.35) | 0.703 | -0.17 (-1.84, 1.51) | 0.846 | -3.23 (-6.65, 0.18) | 0.063 |
| Coffee consumption >4 cups/day | | 2.01 (0.08, 3.94) | **0.041** | 0.66 (-1.21, 2.53) | 0.487 | -0.21 (-2.31, 1.89) | 0.846 | 0.03 (-2.07, 2.13) | 0.980 | -2.16 (-6.10, 1.78) | 0.282 |
| Age | |  |  | 0.24 (0.18, 0.31) | **<0.001** | 0.23 (0.16, 0.30) | **<0.001** | 0.24 (0.17, 0.31) | **<0.001** | 0.21 (0.07, 0.35) | **0.003** |
| Female | |  |  | -13.71 (-14.71, -12.72) | **<0.001** | -13.88 (-14.96, -12.80) | **<0.001** | -13.71 (-14.78, -12.63) | **<0.001** | -14.53 (-16.62, -12.44) | **<0.001** |
| Diabetes mellitus | |  |  | -0.75 (-2.63, 1.14) | 0.438 | -1.50 (-3.56, 0.56) | 0.153 | -1.44 (-3.50, 0.62) | 0.171 | -2.10 (-6.10, 1.91) | 0.305 |
| Arterial hypertension | |  |  | 4.97 (3.85,  6.09) | **<0.001** | 4.93 (3.73, 6.13) | **<0.001** | 4.95 (3.75, 6.16) | **<0.001** | 4.56 (2.20, 6.93) | **<0.001** |
| Current smoking | |  |  | 1.66 (0.37, 2.96) | **0.012** | 2.01 (0.61, 3.41) | **0.005** | 1.81 (0.41, 3.21) | **0.011** | 2.79 (0.04, 5.53) | **0.046** |
| BMI | |  |  | 0.82 (0.71, 0.94) | **<0.001** | 0.80 (0.68, 0.92) | **<0.001** | 0.81 (0.68, 0.93) | **<0.001** | 1.01 (0.77, 1.25) | **<0.001** |
| Additives | **Milk** |  |  |  |  | -1.00 (-2.22, 0.22) | 0.108 |  |  |  |  |
|  | **Milk + Sugar** |  |  |  |  | -3.37 (-5.41, -1.33) | **0.001** |  |  |  |  |
|  | **Milk + Sweetener** |  |  |  |  | -2.46 (-5.52, 0.61) | 0.116 |  |  |  |  |
|  | **Sugar** |  |  |  |  | -2.02 (-6.23, 2.19) | 0.348 |  |  |  |  |
|  | **Sweetener** |  |  |  |  | -2.41 (-7.14, 2.32) | 0.318 |  |  |  |  |
|  | **Binary** |  |  |  |  |  |  | -1.39 (-2.57, -0.22) | **0.020** |  |  |

**Supplementary Table 42. Multivariable linear regression analysis for the association of mild/moderate/high coffee consumption and LV mass index.** Not daily coffee consumption (<1 cups/d) served as the reference group. Adjustment was performed for: A) unadjusted; B) main cardiovascular risk factors; C) additional adjustment for additives (separately), D) additional adjustment for additives (binary), E) same adjustment as for group A, but only for subjects who consumed coffee without any additives. Abbreviations as in Table 1.

|  | | A)E/e‘ ratio | | B)E/e‘ ratio | | C)E/e‘ ratio | | D)E/e‘ ratio | | E)E/e‘ ratio | |
| --- | --- | --- | --- | --- | --- | --- | --- | --- | --- | --- | --- |
|  | | *Estimates* | *p* | *Estimates* | *p* | *Estimates* | *p* | *Estimates* | *p* | *Estimates* | *p* |
| Coffee consumption 1-2 cups/day | | 0.06 (-0.08, 0.20) | 0.383 | -0.01 (-0.15, 0.13) | 0.896 | -0.00 (-0.16, 0.16) | 0.977 | 0.00 (-0.16, 0.16) | 0.976 | -0.24 (-0.59, 0.11) | 0.174 |
| Coffee consumption 3-4 cups/day | | -0.29 (-0.44, -0.14) | **<0.001** | -0.08 (-0.23, 0.07) | 0.309 | -0.10 (-0.28, 0.07) | 0.250 | -0.10 (-0.27, 0.08) | 0.287 | -0.35 (-0.72, 0.02) | 0.061 |
| Coffee consumption > 4 cups/day | | -0.30 (-0.49, -0.11) | **0.002** | -0.04 (-0.23, 0.16) | 0.708 | -0.10 (-0.32, 0.12) | 0.361 | -0.09 (-0.31, 0.13) | 0.439 | -0.17 (-0.60, 0.26) | 0.429 |
| Age | |  |  | 0.06 (0.06, 0.07) | **<0.001** | 0.06 (0.06, 0.07) | **<0.001** | 0.06 (0.06, 0.07) | **<0.001** | 0.07 (0.05, 0.08) | **<0.001** |
| Female | |  |  | 0.67 (0.57, 0.77) | **<0.001** | 0.63 (0.52, 0.74) | **<0.001** | 0.64 (0.52, 0.75) | **<0.001** | 0.64 (0.42, 0.87) | **<0.001** |
| Diabetes mellitus | |  |  | 0.61 (0.42 – 0.80) | **<0.001** | 0.62 (0.41, 0.83) | **<0.001** | 0.64 (0.43, 0.85) | **<0.001** | 1.01 (0.60, 1.42) | **<0.001** |
| Arterial hypertension | |  |  | 0.55 (0.43, 0.67) | **<0.001** | 0.55 (0.42, 0.67) | **<0.001** | 0.55 (0.42, 0.68) | **<0.001** | 0.48 (0.22, 0.73) | **<0.001** |
| Current smoking | |  |  | 0.31 (0.17, 0.44) | **<0.001** | 0.31 (0.17, 0.46) | **<0.001** | 0.31 (0.17, 0.45) | **<0.001** | 0.31 (0.02, 0.60) | **0.039** |
| BMI | |  |  | 0.04 (0.03, 0.06) | **<0.001** | 0.04 (0.03, 0.05) | **<0.001** | 0.04 (0.03, 0.06) | **<0.001** | 0.05 (0.03, 0.08) | **<0.001** |
| Additives | **Milk** |  |  |  |  | -0.04 (-0.17, 0.08) | 0.513 |  |  |  |  |
|  | **Milk + Sugar** |  |  |  |  | -0.17 (-0.38, 0.05) | 0.124 |  |  |  |  |
|  | **Milk + Sweetener** |  |  |  |  | 0.16 (-0.15, 0.48) | 0.311 |  |  |  |  |
|  | **Sugar** |  |  |  |  | -0.26 (-0.70, 0.18) | 0.250 |  |  |  |  |
|  | **Sweetener** |  |  |  |  | 0.34 (-0.17, 0.86) | 0.189 |  |  |  |  |
|  | **Binary** |  |  |  |  |  |  | -0.05 (-0.17, 0.08) | 0.449 |  |  |

**Supplementary Table 43. Multivariable linear regression analysis for the association of mild/moderate/high coffee consumption and E/e’ ratio.** Not daily coffee consumption (<1 cups/d) served as the reference group. Adjustment was performed for: A) unadjusted; B) main cardiovascular risk factors; C) additional adjustment for additives (separately), D) additional adjustment for additives (binary), E) same adjustment as for group A, but only for subjects who consumed coffee without any additives. Abbreviations as in Table 1.

|  | | A)TR Vmax in m/s | | B)TR Vmax in m/s | | C)TR Vmax in m/s | | D)TR Vmax in m/s | | E)TR Vmax in m/s | |
| --- | --- | --- | --- | --- | --- | --- | --- | --- | --- | --- | --- |
|  | | *Estimates* | *p* | *Estimates* | *p* | *Estimates* | *p* | *Estimates* | *p* | *Estimates* | *p* |
| Coffee consumption 1-2 cups/day | | -0.00 (-0.03, 0.02) | 0.747 | -0.02 (-0.05, 0.01) | 0.161 | -0.02 (-0.05, 0.01) | 0.275 | -0.02 (-0.05, 0.02) | 0.346 | -0.04 (-0.10, 0.03) | 0.294 |
| Coffee consumption 3-4 cups/day | | -0.03 (-0.06, 0.00) | 0.069 | -0.03 (-0.06, 0.01) | 0.117 | -0.02 (-0.06, 0.01) | 0.227 | -0.02 (-0.05, 0.02) | 0.322 | -0.09 (-0.16, -0.02) | **0.017** |
| Coffee consumption > 4 cups/day | | -0.04 (-0.08, 0.00) | 0.070 | -0.01 (-0.05, 0.03) | 0.591 | -0.03 (-0.08, 0.02) | 0.232 | -0.02 (-0.07, 0.02) | 0.315 | -0.06 (-0.15, 0.03) | 0.191 |
| Age | |  |  | 0.01 (0.00, 0.01) | **<0.001** | 0.00 (0.00, 0.01) | **<0.001** | 0.00 (0.00, 0.01) | **<0.001** | 0.01 (0.00, 0.01) | **0.002** |
| Female | |  |  | 0.01 (-0.01, 0.03) | 0.366 | 0.01 (-0.02, 0.03) | 0.537 | 0.01 (-0.02, 0.03) | 0.553 | 0.02 (-0.03, 0.06) | 0.517 |
| Diabetes mellitus | |  |  | 0.05 (0.00, 0.09) | **0.042** | 0.03 (-0.01, 0.08) | 0.165 | 0.04 (-0.01, 0.09) | 0.118 | -0.02 (-0.10, 0.07) | 0.739 |
| Arterial hypertension | |  |  | 0.05 (0.03, 0.08) | **<0.001** | 0.05 (0.03, 0.08) | **<0.001** | 0.05 (0.02, 0.08) | **<0.001** | 0.05 (-0.00, 0.10) | 0.063 |
| Current smoking | |  |  | -0.01 (-0.04, 0.02) | 0.588 | -0.01 (-0.04, 0.02) | 0.574 | -0.01 (-0.05, 0.02) | 0.460 | -0.01 (-0.07, 0.06) | 0.789 |
| BMI | |  |  | 0.00 (0.00, 0.01) | **0.031** | 0.00 (0.00, 0.01) | **0.009** | 0.00 (0.00, 0.01) | **0.007** | 0.00 (-0.00, 0.01) | 0.580 |
| Additives | **Milk** |  |  |  |  | -0.03 (-0.06, -0.00) | **0.020** |  |  |  |  |
|  | **Mik+ Sugar** |  |  |  |  | -0.04 (-0.09, 0.00) | 0.055 |  |  |  |  |
|  | **Milk + Sweetener** |  |  |  |  | 0.01 (-0.05, 0.08) | 0.714 |  |  |  |  |
|  | **Sugar** |  |  |  |  | -0.04 (-0.13, 0.05) | 0.407 |  |  |  |  |
|  | **Sweetener** |  |  |  |  | 0.11 (0.01, 0.22) | **0.036** |  |  |  |  |
|  | **Binary** |  |  |  |  |  |  | -0.03 (-0.06, -0.00) | **0.020** |  |  |

**Supplementary Table 44. Multivariable linear regression analysis for the association of mild/moderate/high coffee consumption and TR Vmax in m/s.** Not daily coffee consumption (<1 cups/d) served as the reference group. Adjustment was performed for: A) unadjusted; B) main cardiovascular risk factors; C) additional adjustment for additives (separately), D) additional adjustment for additives (binary), E) same adjustment as for group A, but only for subjects who consumed coffee without any additives. Abbreviations as in Table 1.

|  | | A)TAPSE in mm | | B)TAPSE in mm | | C)TAPSE in mm | | D)TAPSE in mm | | E)TAPSE in mm | |
| --- | --- | --- | --- | --- | --- | --- | --- | --- | --- | --- | --- |
|  | | *Estimates* | *p* | *Estimates* | *p* | *Estimates* | *p* | *Estimates* | *p* | *Estimates* | *p* |
| Coffee consumption 1-2 cups/day | | 0.02 (-0.30, 0.33) | 0.923 | 0.08 (-0.26, 0.42) | 0.644 | 0.16 (-0.23, 0.55) | 0.430 | 0.13 (-0.26, 0.52) | 0.511 | 0.24 (-0.55, 1.02) | 0.554 |
| Coffee consumption 3-4 cups/day | | 0.38 (0.03, 0.72) | **0.033** | 0.18 (-0.19, 0.56) | 0.340 | 0.28 (-0.15, 0.71) | 0.205 | 0.26 (-0.17, 0.69) | 0.242 | 0.47 (-0.37, 1.30) | 0.275 |
| Coffee consumption >4 cups/day | | 0.54 (0.12, 0.97) | **0.013** | 0.33 (-0.14, 0.80) | 0.173 | 0.38 (-0.15, 0.91) | 0.164 | 0.36 (-0.17, 0.90) | 0.179 | 0.30 (-0.65, 1.26) | 0.531 |
| Age | |  |  | -0.06 (-0.07, -0.04) | **<0.001** | -0.06 (-0.08, -0.05) | **<0.001** | -0.06 (-0.08, -0.05) | **<0.001** | -0.05 (-0.09, -0.02) | **0.002** |
| Female | |  |  | -0.74 (-1.00, -0.49) | **<0.001** | -0.73 (-1.00, -0.45) | **<0.001** | -0.72 (-0.99, -0.44) | **<0.001** | -0.72 (-1.23, -0.21) | **0.006** |
| Diabetes mellitus | |  |  | -1.02 (-1.51, -0.52) | **<0.001** | -0.87 (-1.41, -0.33) | **0.002** | -0.93 (-1.47, -0.39) | **0.001** | -1.29 (-2.28, -0.30) | **0.010** |
| Arterial hypertension | |  |  | -0.26 (-0.55, 0.02) | 0.068 | -0.27 (-0.57, 0.03) | 0.082 | -0.28 (-0.59, 0.02) | 0.070 | -0.76 (-1.33, -0.19) | **0.009** |
| Current smoking | |  |  | -0.42 (-0.75, -0.10) | **0.011** | -0.42 (-0.77, -0.07) | **0.019** | -0.41 (-0.76, -0.06) | **0.020** | -0.60 (-1.26, 0.06) | 0.077 |
| BMI | |  |  | 0.01 (-0.02, 0.04) | 0.492 | 0.02 (-0.01, 0.05) | 0.246 | 0.02 (-0.01, 0.05) | 0.244 | 0.04 (-0.02, 0.10) | 0.191 |
| Additives | **Milk** |  |  |  |  | -0.06 (-0.37, 0.25) | 0.715 |  |  |  |  |
|  | **Milk + Sugar** |  |  |  |  | 0.12 (-0.41, 0.64) | 0.664 |  |  |  |  |
|  | **Milk + Sweetener** |  |  |  |  | -0.23 (-0.98, 0.53) | 0.558 |  |  |  |  |
|  | **Sugar** |  |  |  |  | -0.33 (-1.38, 0.72) | 0.535 |  |  |  |  |
|  | **Sweetener** |  |  |  |  | 0.24 (-1.06, 1.54) | 0.719 |  |  |  |  |
|  | **Binary** |  |  |  |  |  |  | -0.05 (-0.35, 0.25) | 0.728 |  |  |

**Supplementary Table 45. Multivariable linear regression analysis for the association of mild/moderate/high coffee consumption and TAPSE in mm.** Not daily coffee consumption (<1 cups/d) served as the reference group. Adjustment was performed for: A) unadjusted; B) main cardiovascular risk factors; C) additional adjustment for additives (separately), D) additional adjustment for additives (binary), E) same adjustment as for group A, but only for subjects who consumed coffee without any additives. Abbreviations as in Table 1.

|  | | A)LBB | | B)LBB | | C)LBB | | D)LBB | | E)LBB | |
| --- | --- | --- | --- | --- | --- | --- | --- | --- | --- | --- | --- |
|  | | *Odds Ratios* | *p* | *Odds Ratios* | *p* | *Odds Ratios* | *p* | *Odds Ratios* | *p* | *Odds Ratios* | *p* |
| Coffee consumption 1-2 cups/day | | 1.04 (0.79, 1.38) | 0.788 | 1.15 (0.86, 1.56) | 0.362 | 1.12 (0.80, 1.59) | 0.523 | 1.10 (0.79, 1.56) | 0.571 | 1.70 (0.82, 4.00) | 0.182 |
| Coffee consumption 3-4 cups/day | | 0.86 (0.63, 1.18) | 0.347 | 1.03 (0.73, 1.45) | 0.880 | 0.99 (0.67, 1.46) | 0.942 | 0.98 (0.67, 1.45) | 0.915 | 1.30 (0.59, 3.18) | 0.535 |
| Coffee consumption >4 cups/day | | 0.94 (0.63, 1.39) | 0.771 | 0.88 (0.55, 1.37) | 0.581 | 0.79 (0.47, 1.30) | 0.360 | 0.78 (0.46, 1.28) | 0.337 | 1.09 (0.42, 2.95) | 0.860 |
| Age | |  |  | 1.03 (1.02, 1.05) | **<0.001** | 1.03 (1.01, 1.05) | **<0.001** | 1.03 (1.01, 1.05) | **<0.001** | 1.02 (0.99, 1.05) | 0.263 |
| Female | |  |  | 0.36 (0.28, 0.47) | **<0.001** | 0.35 (0.27, 0.46) | **<0.001** | 0.35 (0.27, 0.46) | **<0.001** | 0.45 (0.27, 0.73) | **0.002** |
| Diabetes mellitus | |  |  | 0.80 (0.53, 1.17) | 0.265 | 0.81 (0.51, 1.24) | 0.353 | 0.79 (0.50, 1.19) | 0.280 | 0.44 (0.15, 1.03) | 0.089 |
| Arterial hypertension | |  |  | 1.22 (0.92, 1.63) | 0.178 | 1.16 (0.86, 1.57) | 0.334 | 1.15 (0.86, 1.56) | 0.353 | 1.60 (0.89, 3.03) | 0.131 |
| Current smoking | |  |  | 0.87 (0.63, 1.19) | 0.405 | 0.94 (0.67, 1.31) | 0.732 | 0.94 (0.66, 1.30) | 0.706 | 1.21 (0.66, 2.12) | 0.513 |
| BMI | |  |  | 1.04 (1.01, 1.06) | **0.004** | 1.03 (1.01, 1.06) | **0.017** | 1.03 (1.00, 1.06) | **0.024** | 1.06 (1.01, 1.11) | **0.015** |
| Additives | **Milk** |  |  |  |  | 1.08 (0.82, 1.44) | 0.586 |  |  |  |  |
|  | **Milk + Sugar** |  |  |  |  | 1.15 (0.72, 1.77) | 0.550 |  |  |  |  |
|  | **Milk + Sweetener** |  |  |  |  | 0.72 (0.30, 1.49) | 0.417 |  |  |  |  |
|  | **Sugar** |  |  |  |  | 1.50 (0.61, 3.18) | 0.324 |  |  |  |  |
|  | **Sweetener** |  |  |  |  | 0.27 (0.02, 1.25) | 0.196 |  |  |  |  |
|  | **Binary** |  |  |  |  |  |  | 1.07 (0.82, 1.41) | 0.631 |  |  |

**Supplementary Table 46. Multivariable logistic regression analysis for the association of mild/moderate/high coffee consumption and LBBB.** Not daily coffee consumption (<1 cups/d) served as the reference group. Adjustment was performed for: A) unadjusted; B) main cardiovascular risk factors; C) additional adjustment for additives (separately), D) additional adjustment for additives (binary), E) same adjustment as for group A, but only for subjects who consumed coffee without any additives. Abbreviations as in Table 1.

|  | | A)AV block | | B)AV block | | C)AV block | | D)AV block | | E)AV block | |
| --- | --- | --- | --- | --- | --- | --- | --- | --- | --- | --- | --- |
|  | | *Odds Ratios* | *p* | *Odds Ratios* | *p* | *Odds Ratios* | *p* | *Odds Ratios* | *p* | *Odds Ratios* | *p* |
| Coffee consumption 1-2 cups/day | | 0.74 (0.59, 0.93) | **0.010** | 0.79 (0.61, 1.01) | 0.060 | 0.82 (0.62, 1.10) | 0.177 | 0.82 (0.62, 1.10) | 0.176 | 0.63 (0.37, 1.11) | 0.104 |
| Coffee consumption 3-4 cups/day | | 0.74 (0.57, 0.96) | **0.024** | 0.93 (0.70, 1.24) | 0.618 | 0.98 (0.72, 1.35) | 0.917 | 0.98 (0.72 1.35) | 0.916 | 0.73 (0.41, 1.33) | 0.294 |
| Coffee consumption >4 cups/day | | 0.57 (0.39, 0.81) | **0.002** | 0.71 (0.47, 1.06) | 0.100 | 0.71 (0.45, 1.09) | 0.122 | 0.71 (0.45, 1.09) | 0.128 | 0.81 (0.40, 1.60) | 0.541 |
| Age | |  |  | 1.08 (1.06, 1.09) | **<0.001** | 1.08 (1.06, 1.10) | **<0.001** | 1.08 (1.06, 1.10) | **<0.001** | 1.09 (1.06, 1.12) | **<0.001** |
| Female | |  |  | 0.36 (0.29, 0.45) | **<0.001** | 0.36 (0.28, 0.45) | **<0.001** | 0.36 (0.28, 0.45) | **<0.001** | 0.39 (0.25, 0.60) | **<0.001** |
| Diabetes mellitus | |  |  | 1.38 (1.02, 1.84) | **0.034** | 1.45 (1.05, 1.98) | **0.021** | 1.42 (1.03, 1.93) | **0.029** | 2.00 (1.15, 3.38) | **0.012** |
| Arterial hypertension | |  |  | 1.00 (0.78, 1.30) | 0.981 | 1.00 (0.77, 1.32) | 0.978 | 1.00 (0.77, 1.32) | 0.973 | 0.68 (0.43, 1.10) | 0.106 |
| Current smoking | |  |  | 0.77 (0.57, 1.04) | 0.094 | 0.85 (0.62, 1.15) | 0.306 | 0.84 (0.61, 1.14) | 0.279 | 0.81 (0.45, 1.40) | 0.478 |
| BMI | |  |  | 1.03 (1.01, 1.05) | **0.016** | 1.03 (1.00, 1.06) | **0.019** | 1.03 (1.00, 1.05) | **0.025** | 1.02 (0.98, 1.07) | 0.343 |
| Additives | **Milk** |  |  |  |  | 1.07 (0.84, 1.36) | 0.598 |  |  |  |  |
|  | **Milk + Sugar** |  |  |  |  | 1.07 (0.71, 1.59) | 0.739 |  |  |  |  |
|  | **Milk + Sweetener** |  |  |  |  | 0.60 (0.27, 1.15) | 0.156 |  |  |  |  |
|  | **Sugar** |  |  |  |  | 0.62 (0.18, 1.54) | 0.362 |  |  |  |  |
|  | **Sweetener** |  |  |  |  | 0.73 (0.22, 1.86) | 0.559 |  |  |  |  |
|  | **Binary** |  |  |  |  |  |  | 1.03 (0.82, 1.31) | 0.783 |  |  |

**Supplementary Table 47. Multivariable logistic regression analysis for the association of mild/moderate/high coffee consumption and AV block.** Not daily coffee consumption (<1 cups/d) served as the reference group. Adjustment was performed for: A) unadjusted; B) main cardiovascular risk factors; C) additional adjustment for additives (separately), D) additional adjustment for additives (binary), E) same adjustment as for group A, but only for subjects who consumed coffee without any additives. Abbreviations as in Table 1.

|  | | A)Atrial fibrillation | | B)Atrial fibrillation | | C)Atrialc fibrillation | | D)Atrial fibrillation | | E)Atrial fibrillation | |
| --- | --- | --- | --- | --- | --- | --- | --- | --- | --- | --- | --- |
|  | | *Odds Ratios* | *p* | *Odds Ratios* | *p* | *Odds Ratios* | *p* | *Odds Ratios* | *p* | *Odds Ratios* | *p* |
| Coffee consumption 1-2 cups/day | | 0.92 (0.73, 1.16) | 0.479 | 0.99 (0.77, 1.27) | 0.907 | 1.02 (0.77, 1.35) | 0.912 | 1.03 (0.78, 1.36) | 0.850 | 0.81 (0.49, 1.37) | 0.413 |
| Coffee consumption 3-4 cups/day | | 0.71 (0.54, 0.92) | **0.011** | 1.02 (0.76, 1.37) | 0.910 | 0.96 (0.69 , 1.33) | 0.802 | 0.97 (0.70, 1.35) | 0.854 | 0.71 (0.40, 1.28) | 0.250 |
| Coffee consumption >4 cups/day | | 0.47 (0.31, 0.69) | **<0.001** | 0.73 (0.46, 1.11) | 0.152 | 0.69 (0.43, 1.09) | 0.126 | 0.71 (0.44, 1.12) | 0.147 | 0.48 (0.21, 1.01) | 0.059 |
| Age | |  |  | 1.10 (1.08, 1.12) | **<0.001** | 1.10 (1.08, 1.11) | **<0.001** | 1.10 (1.08, 1.12) | **<0.001** | 1.10 (1.06, 1.13) | **<0.001** |
| Female | |  |  | 0.70 (0.57, 0.85) | **<0.001** | 0.64 (0.52, 0.79) | **<0.001** | 0.64 (0.51, 0.79) | **<0.001** | 0.38 (0.24, 0.57) | **<0.001** |
| Diabetes mellitus | |  |  | 1.27 (0.94, 1.69) | 0.110 | 1.23 (0.90, 1.67) | 0.191 | 1.24 (0.90, 1.68) | 0.170 | 1.29 (0.74, 2.18) | 0.357 |
| Arterial hypertension | |  |  | 3.23 (2.32, 4.61) | **<0.001** | 3.10 (2.21, 4.47) | **<0.001** | 3.08 (2.20, 4.45) | **<0.001** | 8.09 (3.57, 23.23) | **<0.001** |
| Current smoking | |  |  | 0.75 (0.55, 1.02) | 0.077 | 0.78 (0.56, 1.08) | 0.145 | 0.77 (0.55, 1.06) | 0.121 | 0.74 (0.39, 1.31) | 0.324 |
| BMI | |  |  | 1.03 (1.00, 1.05) | **0.018** | 1.03 (1.00, 1.05) | **0.029** | 1.03 (1.00, 1.05) | **0.037** | 1.00 (0.96, 1.05) | 0.891 |
| Additives | **Milk** |  |  |  |  | 0.89 (0.71, 1.13) | 0.352 |  |  |  |  |
|  | **Milk + Sugar** |  |  |  |  | 0.94 (0.62, 1.41) | 0.779 |  |  |  |  |
|  | **Milk + Sweetener** |  |  |  |  | 0.76 (0.40, 1.35) | 0.380 |  |  |  |  |
|  | **Sugar** |  |  |  |  | 0.59 (0.18, 1.50) | 0.329 |  |  |  |  |
|  | **Sweetener** |  |  |  |  | 0.92 (0.34, 2.06) | 0.856 |  |  |  |  |
|  | **Binary** |  |  |  |  |  |  | 0.89 (0.71, 1.12) | 0.322 |  |  |

**Supplementary Table 48. Multivariable logistic regression analysis for the association of mild/moderate/high coffee consumption and atrial firbillation.** Not daily coffee consumption (<1 cups/d) served as the reference group. Adjustment was performed for: A) unadjusted; B) main cardiovascular risk factors; C) additional adjustment for additives (separately), D) additional adjustment for additives (binary), E) same adjustment as for group A, but only for subjects who consumed coffee without any additives. Abbreviations as in Table 1.

|  | | A)Diabetes | | B)Diabetes | | C)Diabetes | | D)Diabetes | | E)Diabetes | |
| --- | --- | --- | --- | --- | --- | --- | --- | --- | --- | --- | --- |
|  | | *Odds Ratios* | *p* | *Odds Ratios* | *p* | *Odds Ratios* | *p* | *Odds Ratios* | *p* | *Odds Ratios* | *p* |
| Coffee consumption 1-2 cups/day | | 0.76 (0.63, 0.93) | **0.006** | 0.77 (0.62, 0.96) | **0.020** | 0.72 (0.57, 0.92) | **0.009** | 0.75 (0.59, 0.96) | **0.023** | 0.89 (0.55, 1.47) | 0.630 |
| Coffee consumption 3-4 cups/day | | 0.62 (0.49, 0.77) | **<0.001** | 0.72 (0.56, 0.93) | **0.013** | 0.66 (0.50, 0.88) | **0.005** | 0.69 (0.52, 0.92) | **0.012** | 0.77 (0.45, 1.33) | 0.341 |
| Coffee consumption >4 cups/day | | 0.71 (0.53, 0.94) | **0.020** | 0.72 (0.52, 0.99) | **0.047** | 0.68 (0.47, 0.97) | **0.036** | 0.74 (0.52, 1.06) | 0.101 | 0.75 (0.40, 1.40) | 0.365 |
| Age | |  |  | 1.05 (1.04, 1.06) | **<0.001** | 1.05 (1.04, 1.07) | **<0.001** | 1.06 (1.04, 1.07) | **<0.001** | 1.06 (1.04, 1.09) | **<0.001** |
| Female | |  |  | 0.62 (0.52, 0.74) | **<0.001** | 0.64 (0.53, 0.77) | **<0.001** | 0.66 (0.55, 0.80) | **<0.001** | 0.61 (0.43, 0.87) | **0.006** |
| Arterial hypertension | |  |  | 2.27 (1.75, 2.98) | **<0.001** | 2.41 (1.82, 3.25) | **<0.001** | 2.45 (1.84, 3.31) | **<0.001** | 1.66 (1.03, 2.77) | **0.043** |
| Current smoking | |  |  | 1.42 (1.14, 1.77) | **0.002** | 1.47 (1.16, 1.87) | **0.002** | 1.50 (1.18, 1.91) | **0.001** | 1.35 (0.86, 2.08) | 0.177 |
| BMI | |  |  | 1.16 (1.14, 1.18) | **<0.001** | 1.15 (1.13, 1.17) | **<0.001** | 1.15 (1.13, 1.17) | **<0.001** | 1.16 (1.13, 1.20) | **<0.001** |
| Additives | **Milk** |  |  |  |  | 0.99 (0.80, 1.22) | 0.913 |  |  |  |  |
|  | **Milk + Sugar** |  |  |  |  | 0.53 (0.34, 0.81) | **0.005** |  |  |  |  |
|  | **Milk + Sweetener** |  |  |  |  | 2.58 (1.76, 3.75) | **<0.001** |  |  |  |  |
|  | **Sugar** |  |  |  |  | 0.41 (0.12, 1.02) | 0.090 |  |  |  |  |
|  | **Sweetener** |  |  |  |  | 2.11 (1.12, 3.80) | **0.016** |  |  |  |  |
|  | **Binary** |  |  |  |  |  |  | 1.01 (0.82, 1.24) | 0.939 |  |  |

**Supplementary Table 49. Multivariable logistic regression analysis for the association of mild/moderate/high coffee consumption and diabetes mellitus.** Not daily coffee consumption (<1 cups/d) served as the reference group. Adjustment was performed for: A) unadjusted; B) main cardiovascular risk factors; C) additional adjustment for additives (separately), D) additional adjustment for additives (binary), E) same adjustment as for group A, but only for subjects who consumed coffee without any additives. Abbreviations as in Table 1.

|  | | A)Obesity | | B)Obesity | | C)Obesity | | D)Obesity | | E)Obesity | |
| --- | --- | --- | --- | --- | --- | --- | --- | --- | --- | --- | --- |
|  | | *Odds Ratios* | *p* | *Odds Ratios* | *p* | *Odds Ratios* | *p* | *Odds Ratios* | *p* | *Odds Ratios* | *p* |
| Coffee consumption 1-2 cups/day | | 0.95 (0.82, 1.10) | 0.486 | 0.99 (0.85, 1.17) | 0.945 | 1.02 (0.85, 1.22) | 0.850 | 1.01 (0.84, 1.22) | 0.896 | 1.05 (0.74, 1.52) | 0.778 |
| Coffee consumption 3-4 cups/day | | 1.01 (0.86, 1.18) | 0.913 | 1.13 (0.95, 1.35) | 0.176 | 1.14 (0.93, 1.39) | 0.201 | 1.14 (0.93, 1.39) | 0.214 | 1.09 (0.75, 1.60) | 0.657 |
| Coffee consumption >4 cups/day | | 1.13 (0.92, 1.37) | 0.233 | 1.32 (1.06, 1.65) | **0.013** | 1.33 (1.04, 1.69) | **0.024** | 1.33 (1.04, 1.70) | **0.022** | 1.37 (0.88, 2.11) | 0.160 |
| Age | |  |  | 0.98 (0.97, 0.99) | **<0.001** | 0.98 (0.97, 0.99) | **<0.001** | 0.98 (0.97, 0.99) | **<0.001** | 0.98 (0.97, 1.00) | **0.017** |
| Female | |  |  | 1.11 (0.99, 1.25) | 0.079 | 1.12 (0.99, 1.27) | 0.083 | 1.12 (0.99, 1.28) | 0.073 | 1.00 (0.79, 1.26) | 0.995 |
| Diabetes mellitus | |  |  | 3.84 (3.22, 4.56) | **<0.001** | 3.69 (3.06, 4.46) | **<0.001** | 3.81 (3.16, 4.60) | **<0.001** | 4.65 (3.30, 6.56) | **<0.001** |
| Arterial hypertension | |  |  | 3.28 (2.81, 3.83) | **<0.001** | 3.15 (2.68, 3.71) | **<0.001** | 3.18 (2.70, 3.75) | **<0.001** | 3.53 (2.62, 4.82) | **<0.001** |
| Current smoking | |  |  | 0.92 (0.79, 1.08) | 0.312 | 0.88 (0.74, 1.03) | 0.117 | 0.89 (0.75, 1.05) | 0.161 | 0.94 (0.69, 1.26) | 0.689 |
| Additives | **Milk** |  |  |  |  | 0.81 (0.70, 0.93) | **0.003** |  |  |  |  |
|  | **Milk + Sugar** |  |  |  |  | 0.76 (0.59, 0.98) | **0.034** |  |  |  |  |
|  | **Milk + Sweetener** |  |  |  |  | 1.33 (0.96, 1.81) | 0.082 |  |  |  |  |
|  | **Sugar** |  |  |  |  | 1.28 (0.78, 2.02) | 0.310 |  |  |  |  |
|  | **Sweetener** |  |  |  |  | 1.85 (1.12, 3.00) | **0.014** |  |  |  |  |
|  | **Binary** |  |  |  |  |  |  | 0.84 (0.73, 0.96) | **0.012** |  |  |

**Supplementary Table 50. Multivariable logistic regression analysis for the association of mild/moderate/high coffee consumption and obesity.** Not daily coffee consumption (<1 cups/d) served as the reference group. Adjustment was performed for: A) unadjusted; B) main cardiovascular risk factors; C) additional adjustment for additives (separately), D) additional adjustment for additives (binary), E) same adjustment as for group A, but only for subjects who consumed coffee without any additives. Abbreviations as in Table 1.

|  | | A)CAD | | B)CAD | | C)CAD | | D)CAD | | E)CAD | |
| --- | --- | --- | --- | --- | --- | --- | --- | --- | --- | --- | --- |
|  | | *Odds Ratios* | *p* | *Odds Ratios* | *p* | *Odds Ratios* | *p* | *Odds Ratios* | *p* | *Odds Ratios* | *p* |
| Coffee consumption 1-2 cups/day | | 0.89 (0.71, 1.11) | 0.279 | 0.91 (0.71, 1.17) | 0.443 | 0.88 (0.67, 1.17) | 0.365 | 0.89 (0.68, 1.18) | 0.425 | 0.85 (0.51, 1.46) | 0.550 |
| Coffee consumption 3-4 cups/day | | 0.68 (0.53, 0.88) | **0.003** | 0.89 (0.67, 1.18) | 0.419 | 0.83 (0.60, 1.15) | 0.269 | 0.86 (0.62, 1.18) | 0.341 | 0.73 (0.41, 1.30) | 0.280 |
| Coffee consumption >4 cups/day | | 0.82 (0.59, 1.12) | 0.214 | 0.98 (0.68, 1.41) | 0.926 | 0.95 (0.63, 1.42) | 0.810 | 0.96 (0.64, 1.43) | 0.834 | 0.96 (0.50, 1.82) | 0.891 |
| Age | |  |  | 1.08 (1.07, 1.10) | **<0.001** | 1.09 (1.07, 1.10) | **<0.001** | 1.09 (1.07, 1.10) | **<0.001** | 1.08 (1.05, 1.11) | **<0.001** |
| Female | |  |  | 0.33 (0.26, 0.41) | **<0.001** | 0.33 (0.26, 0.41) | **<0.001** | 0.33 (0.26, 0.42) | **<0.001** | 0.28 (0.17, 0.43) | **<0.001** |
| Diabetes mellitus | |  |  | 1.62 (1.24, 2.11) | **<0.001** | 1.51 (1.12, 2.02) | **0.007** | 1.55 (1.15, 2.07) | **0.003** | 2.04 (1.23, 3.34) | **0.005** |
| Arterial hypertension | |  |  | 4.63 (3.26, 6.78) | **<0.001** | 4.08 (2.84, 6.07) | **<0.001** | 4.07 (2.83, 6.05) | **<0.001** | 4.71 (2.45, 10.25) | **<0.001** |
| Current smoking | |  |  | 1.08 (0.82, 1.40) | 0.598 | 1.12 (0.83, 1.49) | 0.454 | 1.09 (0.81, 1.46) | 0.557 | 1.24 (0.75, 1.99) | 0.396 |
| BMI | |  |  | 1.04 (1.01, 1.06) | **0.001** | 1.04 (1.01, 1.06) | **0.003** | 1.04 (1.01, 1.06) | **0.002** | 1.03 (0.99, 1.07) | 0.140 |
| Additives | **Milk** |  |  |  |  | 0.93 (0.74, 1.17) | 0.532 |  |  |  |  |
|  | **Milk + Sugar** |  |  |  |  | 0.64 (0.40, 0.98) | **0.046** |  |  |  |  |
|  | **Milk + Sweetener** |  |  |  |  | 0.80 (0.43, 1.40) | 0.451 |  |  |  |  |
|  | **Sugar** |  |  |  |  | 0.58 (0.20, 1.37) | 0.259 |  |  |  |  |
|  | **Sweetener** |  |  |  |  | 1.33 (0.58, 2.79) | 0.473 |  |  |  |  |
|  | **Binary** |  |  |  |  |  |  | 0.89 (0.71, 1.11) | 0.290 |  |  |

**Supplementary Table 51. Multivariable logistic regression analysis for the association of mild/moderate/high coffee consumption and CAD.** Not daily coffee consumption (<1 cups/d) served as the reference group. Adjustment was performed for: A) unadjusted; B) main cardiovascular risk factors; C) additional adjustment for additives (separately), D) additional adjustment for additives (binary), E) same adjustment as for group A, but only for subjects who consumed coffee without any additives. Abbreviations as in Table 1.

|  | | A)Heart failure | | B)Heart failure | | C)Heart failure | | D)Heart failure | | E)Heart failure | |
| --- | --- | --- | --- | --- | --- | --- | --- | --- | --- | --- | --- |
|  | | *Odds Ratios* | *p* | *Odds Ratios* | *p* | *Odds Ratios* | *p* | *Odds Ratios* | *p* | *Odds Ratios* | *p* |
| Coffee consumption 1-2 cups/day | | 1.00 (0.74, 1.36) | 0.980 | 1.03 (0.74, 1.45) | 0.854 | 1.10 (0.76, 1.63) | 0.610 | 1.09 (0.75, 1.62) | 0.652 | 0.66 (0.34, 1.33) | 0.232 |
| Coffee consumption 3-4 cups/day | | 0.69 (0.48, 0.99) | **0.045** | 0.89 (0.60, 1.33) | 0.567 | 0.90 (0.57, 1.41) | 0.637 | 0.91 (0.58, 1.43) | 0.676 | 0.45 (0.20, 1.00) | 0.050 |
| Coffee consumption >4 cups/day | | 0.86 (0.55, 1.33) | 0.515 | 1.24 (0.75, 2.00) | 0.388 | 1.13 (0.64, 1.95) | 0.665 | 1.10 (0.62, 1.91) | 0.747 | 0.51 (0.18, 1.32) | 0.179 |
| Age | |  |  | 1.08 (1.06, 1.10) | **<0.001** | 1.08 (1.06, 1.10) | **<0.001** | 1.08 (1.06, 1.10) | **<0.001** | 1.10 (1.05, 1.15) | **<0.001** |
| Female | |  |  | 0.74 (0.57, 0.96) | **0.025** | 0.73 (0.55, 0.97) | **0.029** | 0.73 (0.55, 0.97) | **0.031** | 0.66 (0.38, 1.12) | 0.125 |
| Diabetes mellitus | |  |  | 1.87 (1.33, 2.59) | **<0.001** | 1.92 (1.33, 2.73) | **<0.001** | 1.89 (1.30, 2.69) | **0.001** | 2.45 (1.28, 4.52) | **0.005** |
| Arterial hypertension | |  |  | 4.28 (2.68, 7.29) | **<0.001** | 4.47 (2.69, 8.02) | **<0.001** | 4.41 (2.65, 7.90) | **<0.001** | 5.26 (2.09, 17.67) | **0.002** |
| Current smoking | |  |  | 1.35 (0.95, 1.89) | 0.088 | 1.38 (0.93, 1.99) | 0.097 | 1.35 (0.92, 1.96) | 0.118 | 2.20 (1.09, 4.27) | **0.023** |
| BMI | |  |  | 1.09 (1.06, 1.11) | **<0.001** | 1.09 (1.06, 1.12) | **<0.001** | 1.08 (1.05, 1.12) | **<0.001** | 1.06 (1.00, 1.12) | **0.031** |
| Additives | **Milk** |  |  |  |  | 1.05 (0.77, 1.45) | 0.750 |  |  |  |  |
|  | **Milk + Sugar** |  |  |  |  | 0.94 (0.51, 1.63) | 0.825 |  |  |  |  |
|  | **Milk + Sweetener** |  |  |  |  | 0.40 (0.14, 0.94) | 0.058 |  |  |  |  |
|  | **Sugar** |  |  |  |  | 0.90 (0.21, 2.63) | 0.862 |  |  |  |  |
|  | **Sweetener** |  |  |  |  | 2.13 (0.77, 5.06) | 0.109 |  |  |  |  |
|  | **Binary** |  |  |  |  |  |  | 0.99 (0.73, 1.35) | 0.931 |  |  |

**Supplementary Table 52. Multivariable logistic regression analysis for the association of mid/moderate/high coffee consumption and heart failure.** Not daily coffee consumption (<1 cups/d) served as the reference group. Adjustment was performed for: A) unadjusted; B) main cardiovascular risk factors; C) additional adjustment for additives (separately), D) additional adjustment for additives (binary), E) same adjustment as for group A, but only for subjects who consumed coffee without any additives. Abbreviations as in Table 1.

|  | | A)HF(m)rEF | | B)HF(m)rEF | | C)HF(m)rEF | | D)HF(m)rEF | | E)HF(m)rEF | |
| --- | --- | --- | --- | --- | --- | --- | --- | --- | --- | --- | --- |
|  | | *Odds Ratios* | *p* | *Odds Ratios* | *p* | *Odds Ratios* | *p* | *Odds Ratios* | *p* | *Odds Ratios* | *p* |
| Coffee consumption 1-2 cups/day | | 0.92 (0.61, 1.40) | 0.685 | 0.99 (0.64, 1.55) | 0.955 | 1.11 (0.67, 1.90) | 0.691 | 1.06 (0.64, 1.81) | 0.829 | 0.47 (0.19, 1.25) | 0.114 |
| Coffee consumption 3-4 cups/day | | 0.74 (0.46, 1.20) | 0.221 | 0.92 (0.55, 1.56) | 0.763 | 1.01 (0.56, 1.86) | 0.970 | 0.99 (0.55, 1.82) | 0.983 | 0.56 (0.20, 1.60) | 0.273 |
| Coffee consumption >4 cups/day | | 0.85 (0.46, 1.51) | 0.586 | 1.14 (0.59, 2.13) | 0.677 | 1.29 (0.62, 2.62) | 0.492 | 1.18 (0.56, 2.43) | 0.652 | 0.61 (0.15, 2.10) | 0.449 |
| Age | |  |  | 1.07 (1.04, 1.10) | **<0.001** | 1.07 (1.04, 1.10) | **<0.001** | 1.07 (1.04, 1.10) | **<0.001** | 1.09 (1.03, 1.16) | **0.006** |
| Female | |  |  | 0.38 (0.25, 0.55) | **<0.001** | 0.39 (0.25, 0.58) | **<0.001** | 0.40 (0.26, 0.60) | **<0.001** | 0.49 (0.21, 1.06) | 0.083 |
| Diabetes mellitus | |  |  | 1.71 (1.09, 2.60) | **0.016** | 1.76 (1.07, 2.80) | **0.021** | 1.69 (1.03, 2.70) | **0.033** | 2.03 (0.80, 4.74) | 0.116 |
| Arterial hypertension | |  |  | 4.95 (2.53, 11.20) | **<0.001** | 4.90 (2.39, 11.80) | **<0.001** | 4.88 (2.38, 11.75) | **<0.001** | 10.39 (2.15, 187.21) | **0.023** |
| Current smoking | |  |  | 1.06 (0.64, 1.69) | 0.807 | 1.11 (0.64, 1.84) | 0.685 | 1.12 (0.65, 1.85) | 0.660 | 1.28 (0.41, 3.35) | 0.641 |
| BMI | |  |  | 1.09 (1.05, 1.13) | **<0.001** | 1.08 (1.04, 1.12) | **<0.001** | 1.08 (1.03, 1.12) | **<0.001** | 1.04 (0.96, 1.12) | 0.261 |
| Additives | **Milk** |  |  |  |  | 1.27 (0.84, 1.98) | 0.272 |  |  |  |  |
|  | **Milk + Sugar** |  |  |  |  | 1.06 (0.47, 2.20) | 0.876 |  |  |  |  |
|  | **Milk + Sweetener** |  |  |  |  | 0.37 (0.06, 1.24) | 0.174 |  |  |  |  |
|  | **Sugar** |  |  |  |  | 0.58 (0.03, 2.85) | 0.599 |  |  |  |  |
|  | **Sweetener** |  |  |  |  | 3.14 (0.88, 8.73) | **0.045** |  |  |  |  |
|  | **Binary** |  |  |  |  |  |  | 1.17 (0.78, 1.81) | 0.454 |  |  |

**Supplementary Table 53. Multivariable logistic regression analysis for the association of mid/moderate/high coffee consumption and HF(m)rEF.** Not daily coffee consumption (<1 cups/d) served as the reference group. Adjustment was performed for: A) unadjusted; B) main cardiovascular risk factors; C) additional adjustment for additives (separately), D) additional adjustment for additives (binary), E) same adjustment as for group A, but only for subjects who consumed coffee without any additives. Abbreviations as in Table 1.

|  | | A)HFpEF | | B)HFpEF | | C)HFpEF | | D)HFpEF | | E)HFpEF | |
| --- | --- | --- | --- | --- | --- | --- | --- | --- | --- | --- | --- |
|  | | *Odds Ratios* | *p* | *Odds Ratios* | *p* | *Odds Ratios* | *p* | *Odds Ratios* | *p* | *Odds Ratios* | *p* |
| Coffee consumption 1-2 cups/day | | 1.09 (0.71, 1.73) | 0.706 | 1.09 (0.68, 1.78) | 0.738 | 1.07 (0.64, 1.85) | 0.811 | 1.10 (0.65, 1.92) | 0.739 | 0.91 (0.39, 2.42) | 0.847 |
| Coffee consumption 3-4 cups/day | | 0.64 (0.36, 1.10) | 0.108 | 0.87 (0.48, 1.58) | 0.650 | 0.79 (0.41, 1.51) | 0.466 | 0.82 (0.43, 1.59) | 0.553 | 0.37 (0.11, 1.22) | 0.105 |
| Coffee consumption >4 cups/day | | 0.88 (0.45, 1.65) | 0.688 | 1.34 (0.65, 2.68) | 0.412 | 0.92 (0.39, 2.07) | 0.854 | 0.97 (0.40, 2.19) | 0.948 | 0.45 (0.09, 1.75) | 0.272 |
| Age | |  |  | 1.09 (1.06, 1.12) | **<0.001** | 1.09 (1.06, 1.12) | **<0.001** | 1.09 (1.05, 1.12) | **<0.001** | 1.10 (1.04, 1.17) | **0.002** |
| Female | |  |  | 1.58 (1.09, 2.31) | **0.016** | 1.48 (1.00, 2.21) | 0.054 | 1.43 (0.96, 2.14) | 0.079 | 0.89 (0.43, 1.81) | 0.756 |
| Diabetes mellitus | |  |  | 1.92 (1.17, 3.04) | **0.008** | 1.98 (1.18, 3.23) | **0.007** | 2.01 (1.20, 3.26) | **0.006** | 2.65 (1.12, 5.89) | **0.020** |
| Arterial hypertension | |  |  | 3.62 (1.94, 7.56) | **<0.001** | 3.96 (1.99, 9.04) | **<0.001** | 3.87 (1.94, 8.83) | **<0.001** | 3.47 (1.18, 14.80) | **0.046** |
| Current smoking | |  |  | 1.69 (1.03, 2.67) | **0.030** | 1.69 (0.98, 2.78) | **0.048** | 1.60 (0.93, 2.66) | 0.077 | 3.20 (1.29, 7.45) | **0.009** |
| BMI | |  |  | 1.07 (1.03, 1.11) | **<0.001** | 1.08 (1.04, 1.13) | **<0.001** | 1.08 (1.04, 1.12) | **<0.001** | 1.06 (0.99, 1.14) | 0.078 |
| Additives | **Milk** |  |  |  |  | 0.85 (0.55, 1.32) | 0.454 |  |  |  |  |
|  | **Milk + Sugar** |  |  |  |  | 0.83 (0.33, 1.81) | 0.657 |  |  |  |  |
|  | **Milk + Sweetener** |  |  |  |  | 0.46 (0.11, 1.31) | 0.203 |  |  |  |  |
|  | **Sugar** |  |  |  |  | 1.15 (0.18, 4.10) | 0.852 |  |  |  |  |
|  | **Sweetener** |  |  |  |  | 1.08 (0.17, 3.85) | 0.918 |  |  |  |  |
|  | **Binary** |  |  |  |  |  |  | 0.81 (0.54, 1.25) | 0.338 |  |  |

**Supplementary Table 54. Multivariable logistic regression analysis for the association of mid/moderate/high coffee consumption and HFpEF.** Not daily coffee consumption (<1 cups/d) served as the reference group. Adjustment was performed for: A) unadjusted; B) main cardiovascular risk factors; C) additional adjustment for additives (separately), D) additional adjustment for additives (binary), E) same adjustment as for group A, but only for subjects who consumed coffee without any additives. Abbreviations as in Table 1.

|  | Total cholesterol | | LDL Cholesterol | | HDL cholesterol | | NT-proBNP | |
| --- | --- | --- | --- | --- | --- | --- | --- | --- |
|  | beta (95%CI) | p-value | beta (95%CI) | p-value | beta (95%CI) | p-value | beta (95%CI) | p-value |
| Moderate (3-4 cups/day) | 1.09 (-1.14, 3.33) | 0.337 | 1.63 (-0.42, 3.68) | 0.119 | 0.57 (-0.32, 1.46) | 0.207 | -0.06 (-0.11, -0.02) | 0.005 |
| High (>4 cups/day) | 4.78 (1.56, 8.00) | 0.004 | 5.92 (2.95, 8.89) | < 0.001 | -0.83 (-2.11, 0.45) | 0.206 | -0.09 (-0.15, -0.02) | 0.013 |
| *Age, years* | 0.10 {-0.03, 0.22} | 0.129 | 0.01 (-0.11, 0.12) | 0.924 | 0.14 {0.09, 0.19} | <0.001 | 0.05 {0.04, 0.05} | <0.001 |
| *Female Sex* | 21.37 {19.43, 23.30} | <0.001 | 9.04 (7.26, 10.82 | < 0.001 | 15.38 {14.61, 16.15} | <0.001 | 0.38 {0.34, 0.42} | <0.001 |
| *BMI, kg/m^2^* | -0.29 {-0.51, -0.07} | 0.010 | 0.35 (0.16, 0.54) | < 0.001 | -1.38 {-1.47, -1.29} | <0.001 | -0.01 {-0.02, 0.01} | <0.001 |
| *Arterial hypertension* | 2.32 {0.12, 4.51} | 0.038 | 0.44 (-1.58 – 2.45) | 0.671 | -0.14 {-1.01, 0.74} | 0.761 | 0.18 {0.14, 0.23} | <0.001 |
| *Diabetes* | -20.87 {-24.52, 17.22} | <0.001 | -22.27 (-25.67, -18.86) | < 0.001 | -6.47 {-7.92, -5.02} | <0.001 | -0.03 {-0.10,0.05} | 0.464 |
| *Smoking* | -2.45 {-4.95, 0.04} | 0.054 | -0.48 (-2.77, 1.82) | 0.683 | -5.63 {-6.62, -4.64} | <0.001 | 0.08 {0.03, 0.13} | 0.003 |
| *Lipid lowering drugs* | -33.54 {-35.93, 31.14} | <0.001 | -33.54 {-35.93, 31.14} | <0.001 | -33.54 {-35.93, 31.14} | <0.001 | - | - |
| *Additives* | -2.18 {-4.29, -0.07} | 0.043 | -0.07 {-2.00, 1.87} | 0.947 | -2.42 {-3.25, -1,58} | <0.001 | -0.02 {-0.06, 0.03} | 0.449 |

**Supplementary Table 55. Multivariable linear regression analysis for the association of moderate/high coffee consumption and LDL** **laboratory markers.** Mild coffee consumption (<3 cups/d) served as the reference group. Adjustment was performed for age, female sex, BMI, arterial hypertension, diabetes, smoking, lipid lowering drugs and additives. Abbreviations as in Table 1.

|  | Systolic blood pressure | | Diastolic blood pressure | | Heart rate | |
| --- | --- | --- | --- | --- | --- | --- |
|  | beta (95%CI) | p-value | beta (95%CI) | p-value | beta (95%CI) | p-value |
| Moderate (3-4 cups/day) | -1.91 (-3.04, -0.78) | 0.001 | -1.05 {-1.67, -0.43} | 0.001 | -0.62 (-1.24, 0.01) | 0.052 |
| High (>4 cups/day) | -3.06 (-4.69, -1.44) | <0.001 | -1.85 {-2.74, -0.96} | <0.001 | -0.02 (-0.92, 0.89) | 0.969 |
| *Age, years* | 0.80 (0.74, 0.86) | < 0.001 | 0.03 {0.00, 0.07} | 0.038 | 0.03 (-0.00, 0.07) | 0.050 |
| *Female Sex* | -5.08 (-6.05, -4.10) | < 0.001 | -3.08 {-3.62, -2.55} | <0.001 | 2.47 (1.92, 3.01) | <0.001 |
| *BMI, kg/m^2^* | 0.68 (0.57, 0.79) | < 0.001 | 0.45 {0.39, 0.51} | <0.001 | 0.26 (0.19, 0.32) | <0.001 |
| *Arterial hypertension* | - | - | - | - | 1.45 (0.84, 2.06) | <0.001 |
| *Diabetes* | 4.21 (2.36, 6.06) | < 0.001 | 0.20 {-0.82, 1.21} | 0.704 | 3.57 (2.56, 4.59) | <0.001 |
| *Smoking* | -1.88 (-3.13, -0.62) | 0.003 | -0.90 {-1.59, -0.21} | 0.011 | 1.46 (0.77, 2.16) | <0.001 |
| *Additives* | -0.87 {-1.93, 0.20} | 0.111 | -0.42 {-1.01, -0.16} | 0.155 | 0.07 (-0.52, 0.66) | 0.818 |

**Supplementary Table 56. Multivariable linear regression analysis for the association of moderate/high coffee consumption and vital signs.** Mild coffee consumption (<3 cups/d) served as the reference group. Adjustment was performed for age, female sex, BMI, diabetes, smoking and additives. Systolic blood pressure value for treated individuals was imputed by adding 15 mmHg to measured blood pressure. Abbreviations as in Table 1.

|  | | A)Total cholesterol | | B)Total cholesterol | | C)Total cholesterol | | DTotal cholesterol | | E)Total cholesterol | | F)Total cholesterol | |
| --- | --- | --- | --- | --- | --- | --- | --- | --- | --- | --- | --- | --- | --- |
|  | | *Estimates* | *p* | *Estimates* | *p* | *Estimates* | *p* | *Estimates* | *p* | *Estimates* | *p* | *Estimates* | *p* |
| Coffee consumption 3-4 cups/day | | 3.42 (-3.30 – 10.15) | 0.318 | 3.05 (-3.84 – 9.94) | 0.385 | 2.04 (-5.07 – 9.15) | 0.573 | 2.08 (-5.04 – 9.19) | 0.567 | 2.82 (-3.69 – 9.34) | 0.395 | 4.48 (-8.49 – 17.45) | 0.496 |
| Coffee consumption > 4 cups/day] | | -2.71 (-12.80 – 7.37) | 0.598 | 2.11 (-8.22 – 12.43) | 0.689 | 1.70 (-9.31 – 12.71) | 0.762 | 1.49 (-9.54 – 12.51) | 0.791 | 3.02 (-6.72 – 12.77) | 0.543 | 0.37 (-16.89 – 17.64) | 0.966 |
| Age | |  |  | -0.17 (-0.56 – 0.22) | 0.391 | -0.21 (-0.62 – 0.20) | 0.316 | -0.20 (-0.62 – 0.21) | 0.327 | 0.22 (-0.16 – 0.60) | 0.248 | -0.52 (-1.30 – 0.26) | 0.190 |
| Female | |  |  | 22.99 (16.76 – 29.22) | **<0.001** | 23.04 (16.54 – 29.54) | **<0.001** | 23.21 (16.73 – 29.70) | **<0.001** | 21.28 (15.39 – 27.18) | **<0.001** | 22.86 (11.18 – 34.54) | **<0.001** |
| Diabetes mellitus | |  |  | -31.16 (-41.92 – -20.40) | **<0.001** | -29.55 (-41.16 – -17.95) | **<0.001** | -30.61 (-42.25 – -18.96) | **<0.001** | -20.80 (-31.31 – -10.28) | **<0.001** | -27.76 (-51.41 – -4.12) | **0.022** |
| Arterial hypertension | |  |  | 8.39 (1.10 – 15.67) | **0.024** | 7.21 (-0.36 – 14.77) | 0.062 | 6.61 (-0.93 – 14.14) | 0.086 | 11.57 (4.66 – 18.48) | **0.001** | 13.21 (-0.65 – 27.06) | 0.062 |
| Current smoking | |  |  | -5.38 (-13.06 – 2.30) | 0.170 | -5.61 (-13.68 – 2.46) | 0.173 | -4.96 (-13.01 – 3.10) | 0.227 | -3.01 (-10.28 – 4.26) | 0.416 | -5.87 (-20.86 – 9.12) | 0.441 |
| BMI | |  |  | -0.00 (-0.70 – 0.70) | 0.995 | 0.08 (-0.66 – 0.83) | 0.824 | 0.09 (-0.65 – 0.84) | 0.806 | -0.00 (-0.66 – 0.66) | 0.992 | -0.56 (-1.85 – 0.72) | 0.388 |
| Additives | **Milk** |  |  |  |  | -5.54 (-12.46 – 1.37) | 0.116 |  |  |  |  |  |  |
| **Milk + Sugar** |  |  |  |  | 3.41 (-11.36 – 18.18) | 0.651 |  |  |  |  |  |  |
| **Milk +  Sweetener** |  |  |  |  | -2.27 (-19.90 – 15.36) | 0.800 |  |  |  |  |  |  |
| **Sugar** |  |  |  |  | 0.22 (-39.21 – 39.65) | 0.991 |  |  |  |  |  |  |
| **Sweetener** |  |  |  |  | 1.96 (-22.41 – 26.34) | 0.874 |  |  |  |  |  |  |
| **Binary** |  |  |  |  |  |  | -4.64 (-11.30 – 2.03) | 0.172 |  |  |  |  |
| Lipid lowering drugs | |  |  |  |  |  |  |  |  | -35.67 (-43.28 – -28.06) | **<0.001** |  |  |

**Supplementary Table 57. Multivariable linear regression analysis for the association of moderate/high coffee consumption and total cholesterol for decaffeinated coffee consumption.** Mild coffee consumption (<3 cups/d) served as the reference group. Adjustment was performed for: A) unadjusted; B) main cardiovascular risk factors; C) additional adjustment for additives (separately), D) additional adjustment for additives (binary), E) additional adjustment for lipid lowering drugs, F) same adjustment as for group A, but only for subjects who consumed coffee without any additives.

|  | | A)LDL | | B)LDL | | C)LDL | | D)LDL | | E)LDL | | F)LDL | |
| --- | --- | --- | --- | --- | --- | --- | --- | --- | --- | --- | --- | --- | --- |
|  | | *Estimates* | *p* | *Estimates* | *p* | *Estimates* | *p* | *Estimates* | *p* | *Estimates* | *p* | *Estimates* | *p* |
| Coffee consumption 3-4 cups/day | | 3.19 (-2.73 – 9.12) | 0.290 | 2.74 (-3.42 – 8.91) | 0.382 | 1.53 (-4.84 – 7.90) | 0.637 | 1.37 (-5.02 – 7.76) | 0.674 | 2.53 (-3.29 – 8.36) | 0.393 | 6.03 (-5.49 – 17.54) | 0.304 |
| Coffee consumption > 4 cups/day | | 4.10 (-4.81 – 13.01) | 0.366 | 6.49 (-2.79 – 15.77) | 0.170 | 6.52 (-3.41 – 16.45) | 0.198 | 5.99 (-3.95 – 15.94) | 0.237 | 7.09 (-1.66 – 15.84) | 0.112 | 4.78 (-10.55 – 20.12) | 0.539 |
| Age | |  |  | -0.17 (-0.52 – 0.19) | 0.356 | -0.19 (-0.56 – 0.18) | 0.305 | -0.20 (-0.57 – 0.16) | 0.276 | 0.19 (-0.15 – 0.52) | 0.284 | -0.40 (-1.09 – 0.30) | 0.260 |
| Female | |  |  | 9.69 (4.10 – 15.27) | **0.001** | 9.51 (3.66 – 15.36) | **0.001** | 9.44 (3.60 – 15.28) | **0.002** | 8.18 (2.89 – 13.46) | **0.002** | 9.01 (-1.36 – 19.38) | 0.088 |
| Diabetes mellitus | |  |  | -31.57 (-41.34 – -21.81) | **<0.001** | -29.52 (-40.09 – -18.96) | **<0.001** | -29.61 (-40.17 – -19.05) | **<0.001** | -22.12 (-31.66 – -12.58) | **<0.001** | -29.42 (-50.42 – -8.41) | **0.006** |
| Arterial hypertension | |  |  | 5.50 (-1.02 – 12.02) | 0.098 | 4.23 (-2.55 – 11.00) | 0.221 | 3.82 (-2.95 – 10.60) | 0.268 | 8.33 (2.15 – 14.51) | **0.008** | 8.25 (-4.05 – 20.56) | 0.187 |
| Current smoking | |  |  | -2.66 (-9.55 – 4.24) | 0.449 | -3.06 (-10.31 – 4.18) | 0.407 | -2.21 (-9.45 – 5.04) | 0.550 | -0.40 (-6.92 – 6.12) | 0.904 | -1.15 (-14.46 – 12.16) | 0.865 |
| BMI | |  |  | 0.73 (0.10 – 1.36) | **0.022** | 0.84 (0.17 – 1.51) | **0.014** | 0.85 (0.18 – 1.53) | **0.013** | 0.73 (0.13 – 1.32) | **0.016** | 0.20 (-0.94 – 1.35) | 0.724 |
| Additives | Milk |  |  |  |  | -2.00 (-8.19 – 4.20) | 0.527 |  |  |  |  |  |  |
|  | Milk + Sugar |  |  |  |  | 5.63 (-7.59 – 18.85) | 0.403 |  |  |  |  |  |  |
|  | Milk +  Sweetener |  |  |  |  | 1.12 (-14.96 – 17.20) | 0.891 |  |  |  |  |  |  |
|  | Sugar |  |  |  |  | 17.56 (-17.71 – 52.84) | 0.329 |  |  |  |  |  |  |
|  | Sweetener |  |  |  |  | 1.01 (-21.78 – 23.79) | 0.931 |  |  |  |  |  |  |
|  | Binary |  |  |  |  |  |  | -1.05 (-7.04 – 4.94) | 0.731 |  |  |  |  |
| Lipid lowering drugs | |  |  |  |  |  |  |  |  | -32.29 (-39.14 – -25.44) | **<0.001** |  |  |

**Supplementary Table 58. Multivariable linear regression analysis for the association of moderate/high coffee consumption and LDL for decaffeinated coffee consumption.** Mild coffee consumption (<3 cups/d) served as the reference group. Adjustment was performed for: A) unadjusted; B) main cardiovascular risk factors; C) additional adjustment for additives (separately), D) additional adjustment for additives (binary), E) additional adjustment for lipid lowering drugs, F) same adjustment as for group A, but only for subjects who consumed coffee without any additives.

|  | | A)HDL | | B)HDL | | C)HDL | | D)HDL | | E)HDL | | F)HDL | |
| --- | --- | --- | --- | --- | --- | --- | --- | --- | --- | --- | --- | --- | --- |
|  | | *Estimates* | *p* | *Estimates* | *p* | *Estimates* | *p* | *Estimates* | *p* | *Estimates* | *p* | *Estimates* | *p* |
| Coffee consumption 3 -4 cups/day | | 0.84 (-2.26 – 3.93) | 0.595 | 1.59 (-1.23 – 4.40) | 0.269 | 1.59 (-1.35 – 4.53) | 0.287 | 1.69 (-1.27 – 4.65) | 0.262 | 1.60 (-1.21 – 4.41) | 0.263 | -0.43 (-6.09 – 5.22) | 0.880 |
| Coffee consumption > 4 cups/day | | -6.32 (-10.96 – -1.68) | **0.008** | -1.69 (-5.91 – 2.52) | 0.431 | -2.70 (-7.26 – 1.85) | 0.244 | -2.85 (-7.44 – 1.73) | 0.221 | -1.54 (-5.74 – 2.66) | 0.473 | -1.46 (-8.99 – 6.07) | 0.702 |
| Age | |  |  | 0.00 (-0.16 – 0.16) | 0.975 | -0.05 (-0.22 – 0.12) | 0.580 | -0.04 (-0.21 – 0.13) | 0.662 | 0.05 (-0.11 – 0.22) | 0.530 | -0.12 (-0.46 – 0.22) | 0.482 |
| Female | |  |  | 15.10 (12.55 – 17.64) | **<0.001** | 14.81 (12.13 – 17.50) | **<0.001** | 14.96 (12.26 – 17.65) | **<0.001** | 14.91 (12.37 – 17.44) | **<0.001** | 15.12 (10.02 – 20.21) | **<0.001** |
| Diabetes mellitus | |  |  | -5.44 (-9.83 – -1.05) | **0.015** | -5.09 (-9.90 – -0.29) | **0.038** | -5.40 (-10.24 – -0.56) | **0.029** | -4.29 (-8.82 – 0.23) | 0.063 | -8.38 (-18.70 – 1.93) | 0.110 |
| Arterial hypertension | |  |  | -0.28 (-3.25 – 2.70) | 0.855 | 0.09 (-3.04 – 3.22) | 0.953 | 0.02 (-3.11 – 3.16) | 0.988 | 0.13 (-2.84 – 3.11) | 0.929 | 3.11 (-2.93 – 9.16) | 0.311 |
| Current smoking | |  |  | -6.50 (-9.63 – -3.36) | **<0.001** | -6.06 (-9.40 – -2.73) | **<0.001** | -6.18 (-9.52 – -2.83) | **<0.001** | -6.18 (-9.31 – -3.05) | **<0.001** | -9.22 (-15.75 – -2.68) | **0.006** |
| BMI | |  |  | -1.37 (-1.65 – -1.08) | **<0.001** | -1.42 (-1.72 – -1.11) | **<0.001** | -1.43 (-1.74 – -1.12) | **<0.001** | -1.36 (-1.65 – -1.08) | **<0.001** | -1.40 (-1.96 – -0.84) | **<0.001** |
| Additives | **Milk** |  |  |  |  | -4.28 (-7.14 – -1.42) | **0.003** |  |  |  |  |  |  |
| **Milk + Sugar** |  |  |  |  | -4.40 (-10.51 – 1.71) | 0.158 |  |  |  |  |  |  |
| **Milk + Sweetener** |  |  |  |  | -7.58 (-14.88 – -0.29) | **0.041** |  |  |  |  |  |  |
| **Sugar** |  |  |  |  | -13.30 (-29.60 – 3.01) | 0.110 |  |  |  |  |  |  |
| **Sweetener** |  |  |  |  | -4.32 (-14.40 – 5.76) | 0.401 |  |  |  |  |  |  |
| **Binary** |  |  |  |  |  |  | -4.63 (-7.40 – -1.86) | **0.001** |  |  |  |  |
| Lipid lowering drugs | |  |  |  |  |  |  |  |  | -4.57 (-7.85 – -1.30) | **0.006** |  |  |

**Supplementary Table 59. Multivariable linear regression analysis for the association of moderate/high coffee consumption and HDL for decaffeinated coffee consumption.** Mild coffee consumption (<3 cups/d) served as the reference group. Adjustment was performed for: A) unadjusted; B) main cardiovascular risk factors; C) additional adjustment for additives (separately), D) additional adjustment for additives (binary), E) additional adjustment for lipid lowering drugs, F) same adjustment as for group A, but only for subjects who consumed coffee without any additives.

|  | | A) NT pro BNP | | B) NT pro BNP | | C) NT pro BNP | | D) NT pro BNP | | E) NT pro BNP | |
| --- | --- | --- | --- | --- | --- | --- | --- | --- | --- | --- | --- |
|  | | *Estimates* | *p* | *Estimates* | *p* | *Estimates* | *p* | *Estimates* | *p* | *Estimates* | *p* |
| Coffee consumption 3-4 cups/day | | -0.19 (-0.33 – -0.04) | **0.010** | -0.04 (-0.18 – 0.10) | 0.583 | -0.02 (-0.16 – 0.12) | 0.759 | -0.01 (-0.16 – 0.13) | 0.841 | 0.19 (-0.06 – 0.44) | 0.134 |
| Coffee consumption >4 cups/day] | | -0.33 (-0.55 – -0.12) | **0.003** | -0.07 (-0.28 – 0.13) | 0.482 | -0.07 (-0.29 – 0.15) | 0.548 | -0.06 (-0.28 – 0.16) | 0.610 | 0.08 (-0.25 – 0.42) | 0.619 |
| Age | |  |  | 0.05 (0.04 – 0.06) | **<0.001** | 0.05 (0.04 – 0.06) | **<0.001** | 0.05 (0.04 – 0.06) | **<0.001** | 0.05 (0.04 – 0.07) | **<0.001** |
| Female | |  |  | 0.35 (0.23 – 0.47) | **<0.001** | 0.34 (0.21 – 0.47) | **<0.001** | 0.34 (0.21 – 0.47) | **<0.001** | 0.37 (0.14 – 0.59) | **0.002** |
| Diabetes mellitus | |  |  | 0.01 (-0.20 – 0.23) | 0.895 | -0.03 (-0.26 – 0.20) | 0.824 | -0.04 (-0.27 – 0.19) | 0.712 | 0.30 (-0.14 – 0.73) | 0.181 |
| Arterial hypertension | |  |  | 0.11 (-0.04 – 0.25) | 0.156 | 0.10 (-0.05 – 0.26) | 0.176 | 0.11 (-0.04 – 0.26) | 0.143 | 0.01 (-0.26 – 0.28) | 0.942 |
| Current smoking | |  |  | 0.01 (-0.14 – 0.16) | 0.881 | 0.04 (-0.12 – 0.20) | 0.657 | 0.03 (-0.13 – 0.20) | 0.673 | 0.14 (-0.15 – 0.43) | 0.343 |
| BMI | |  |  | -0.01 (-0.02 – 0.01) | 0.351 | -0.01 (-0.02 – 0.01) | 0.269 | -0.01 (-0.02 – 0.01) | 0.254 | -0.00 (-0.03 – 0.02) | 0.942 |
| Additives | **Milk** |  |  |  |  | -0.00 (-0.14 – 0.14) | 0.996 |  |  |  |  |
| **Milk + Sugar** |  |  |  |  | -0.17 (-0.47 – 0.13) | 0.258 |  |  |  |  |
| **Milk + Sweetener** |  |  |  |  | -0.36 (-0.71 – -0.01) | **0.046** |  |  |  |  |
| **Sugar** |  |  |  |  | 0.16 (-0.63 – 0.95) | 0.688 |  |  |  |  |
| **Sweetener** |  |  |  |  | -0.05 (-0.54 – 0.43) | 0.831 |  |  |  |  |
| **Binary** |  |  |  |  |  |  | -0.03 (-0.17 – 0.10) | 0.617 |  |  |

**Supplementary Table 60. Multivariable linear regression analysis for the association of moderate/high coffee consumption and NT proBNP for decaffeinated coffee.** Mild coffee consumption (<3 cups/d) served as the reference group. Adjustment was performed for: A) unadjusted; B) main cardiovascular risk factors; C) additional adjustment for additives (separately), D) additional adjustment for additives (binary), E) same adjustment as for group A, but only for subjects who consumed coffee without any additives. Abbreviations as in Table 1.

|  | | A)SBP | | B)SBP | | C)SBP | | D)SBP | | E)SBP | |
| --- | --- | --- | --- | --- | --- | --- | --- | --- | --- | --- | --- |
|  | | *Estimates* | *p* | *Estimates* | *p* | *Estimates* | *p* | *Estimates* | *p* | *Estimates* | *p* |
| Coffee consumption 3-4 cups/day | | -5.05 (-8.66 – -1.43) | **0.006** | -3.70 (-7.21 – -0.18) | **0.040** | -3.93 (-7.63 – -0.23) | **0.037** | -4.17 (-7.88 – -0.45) | **0.028** | -4.96 (-11.18 – 1.26) | 0.117 |
| Coffee consumption >4 cups/day | | -6.28 (-11.60 – -0.96) | **0.021** | -4.79 (-10.02 – 0.44) | 0.073 | -5.78 (-11.46 – -0.10) | **0.046** | -5.01 (-10.72 – 0.69) | 0.085 | -7.78 (-15.96 – 0.41) | 0.062 |
| Age | |  |  | 0.88 (0.69 – 1.07) | **<0.001** | 0.86 (0.65 – 1.06) | **<0.001** | 0.87 (0.67 – 1.08) | **<0.001** | 0.73 (0.38 – 1.09) | **<0.001** |
| Female | |  |  | -4.80 (-7.97 – -1.63) | **0.003** | -4.71 (-8.07 – -1.34) | **0.006** | -4.84 (-8.21 – -1.47) | **0.005** | -5.59 (-11.19 – 0.01) | 0.050 |
| Diabetes mellitus | |  |  | 5.87 (0.37 – 11.37) | **0.036** | 4.53 (-1.49 – 10.56) | 0.140 | 4.37 (-1.69 – 10.44) | 0.157 | 1.87 (-9.08 – 12.82) | 0.737 |
| Current smoking | |  |  | -1.21 (-5.14 – 2.72) | 0.545 | -1.87 (-6.09 – 2.34) | 0.382 | -1.98 (-6.19 – 2.23) | 0.357 | -0.01 (-7.13 – 7.11) | 0.998 |
| BMI | |  |  | 0.42 (0.08 – 0.76) | **0.016** | 0.46 (0.09 – 0.83) | **0.015** | 0.44 (0.07 – 0.81) | **0.020** | 0.72 (0.14 – 1.29) | **0.014** |
| Additives | **Milk** |  |  |  |  | 0.21 (-3.38 – 3.80) | 0.909 |  |  |  |  |
| **Milk + Sugar** |  |  |  |  | -1.46 (-9.10 – 6.18) | 0.708 |  |  |  |  |
| **Milk + Sweetener** |  |  |  |  | 0.57 (-8.54 – 9.69) | 0.902 |  |  |  |  |
| **Sugar** |  |  |  |  | 15.78 (-4.64 – 36.20) | 0.130 |  |  |  |  |
| **Sweetener** |  |  |  |  | 8.13 (-5.10 – 21.35) | 0.228 |  |  |  |  |
| **Binary** |  |  |  |  |  |  | 0.35 (-3.11 – 3.82) | 0.841 |  |  |

**Supplementary Table 61. Multivariable linear regression analysis for the association of moderate/high coffee consumption and SBP for decaffeinated coffee.** Mild coffee consumption (<3 cups/d) served as the reference group. Adjustment was performed for: A) unadjusted; B) main cardiovascular risk factors; C) additional adjustment for additives (separately), D) additional adjustment for additives (binary), E) same adjustment as for group A, but only for subjects who consumed coffee without any additives. Abbreviations as in Table 1.

|  | | A)DBP | | B)DBP | | C)DBP | | D)DBP | | E)DBP | |
| --- | --- | --- | --- | --- | --- | --- | --- | --- | --- | --- | --- |
|  | | *Estimates* | *p* | *Estimates* | *p* | *Estimates* | *p* | *Estimates* | *p* | *Estimates* | *p* |
| Coffee consumption 3-4 cups/day | | -1.64 (-3.47 – 0.20) | 0.080 | -1.60 (-3.53 – 0.33) | 0.105 | -1.82 (-3.83 – 0.19) | 0.075 | -2.05 (-4.05 – -0.05) | **0.045** | -1.79 (-5.22 – 1.65) | 0.306 |
| Coffee consumption >4 cups/day | | -2.35 (-5.05 – 0.35) | 0.088 | -2.66 (-5.54 – 0.22) | 0.070 | -3.80 (-6.89 – -0.71) | **0.016** | -3.79 (-6.87 – -0.71) | **0.016** | -4.82 (-9.37 – -0.27) | **0.038** |
| Age | |  |  | 0.05 (-0.05 – 0.16) | 0.313 | 0.02 (-0.09 – 0.13) | 0.692 | 0.04 (-0.07 – 0.15) | 0.487 | 0.01 (-0.18 – 0.21) | 0.911 |
| Female | |  |  | -3.18 (-4.92 – -1.44) | **<0.001** | -3.48 (-5.31 – -1.65) | **<0.001** | -3.45 (-5.27 – -1.64) | **<0.001** | -4.31 (-7.41 – -1.21) | **0.007** |
| Diabetes mellitus | |  |  | -0.37 (-3.39 – 2.65) | 0.809 | -0.44 (-3.72 – 2.83) | 0.790 | -0.42 (-3.69 – 2.85) | 0.801 | -0.64 (-6.70 – 5.42) | 0.836 |
| Current smoking | |  |  | -1.43 (-3.60 – 0.73) | 0.193 | -1.35 (-3.64 – 0.94) | 0.248 | -1.39 (-3.67 – 0.88) | 0.230 | -2.99 (-6.94 – 0.96) | 0.137 |
| BMI | |  |  | 0.29 (0.10 – 0.47) | **0.003** | 0.27 (0.07 – 0.47) | **0.008** | 0.24 (0.04 – 0.44) | **0.016** | 0.23 (-0.08 – 0.54) | 0.145 |
| Additives | **Milk** |  |  |  |  | 0.48 (-1.47 – 2.43) | 0.630 |  |  |  |  |
| **Milk + Sugar** |  |  |  |  | -1.87 (-6.03 – 2.28) | 0.377 |  |  |  |  |
| **Milk + Sweetener** |  |  |  |  | -0.37 (-5.33 – 4.58) | 0.882 |  |  |  |  |
| **Sugar** |  |  |  |  | 2.89 (-8.22 – 14.00) | 0.609 |  |  |  |  |
| **Sweetener** |  |  |  |  | 2.98 (-4.21 – 10.18) | 0.416 |  |  |  |  |
| **Binary** |  |  |  |  |  |  | 0.22 (-1.65 – 2.09) | 0.819 |  |  |

**Supplementary Table 62. Multivariable linear regression analysis for the association of moderate/high coffee consumption and DBP for decaffeinated coffee.** Mild coffee consumption (<3 cups/d) served as the reference group. Adjustment was performed for: A) unadjusted; B) main cardiovascular risk factors; C) additional adjustment for additives (separately), D) additional adjustment for additives (binary), E) same adjustment as for group A, but only for subjects who consumed coffee without any additives. Abbreviations as in Table 1.

|  | | A)Heart rate | | B)Heart rate | | C)Heart rate | | D)Heart rate | | E)Heart rate | |
| --- | --- | --- | --- | --- | --- | --- | --- | --- | --- | --- | --- |
|  | | *Estimates* | *p* | *Estimates* | *p* | *Estimates* | *p* | *Estimates* | *p* | *Estimates* | *p* |
| Coffee consumption3-4 cups/day | | -0.36 (-2.13 – 1.41) | 0.690 | -0.12 (-1.97 – 1.72) | 0.896 | -0.16 (-2.12 – 1.79) | 0.869 | -0.27 (-2.22 – 1.69) | 0.789 | 0.82 (-2.70 – 4.35) | 0.645 |
| Coffee consumption > 4 cups/day | | -0.67 (-3.29 – 1.96) | 0.617 | 0.01 (-2.74 – 2.76) | 0.994 | 0.87 (-2.14 – 3.88) | 0.572 | 0.43 (-2.58 – 3.43) | 0.781 | 1.10 (-3.57 – 5.77) | 0.643 |
| Age | |  |  | 0.07 (-0.04 – 0.17) | 0.201 | 0.07 (-0.04 – 0.19) | 0.197 | 0.08 (-0.03 – 0.19) | 0.173 | 0.04 (-0.17 – 0.25) | 0.717 |
| Female | |  |  | 2.93 (1.26 – 4.60) | **0.001** | 3.20 (1.41 – 4.98) | **<0.001** | 3.22 (1.44 – 4.99) | **<0.001** | 5.77 (2.59 – 8.95) | **<0.001** |
| Diabetes mellitus | |  |  | 1.07 (-1.79 – 3.93) | 0.463 | 1.31 (-1.84 – 4.46) | 0.414 | 1.66 (-1.48 – 4.81) | 0.299 | 1.06 (-4.99 – 7.10) | 0.731 |
| Arterial hypertension | |  |  | 0.07 (-1.89 – 2.02) | 0.945 | 0.29 (-1.79 – 2.37) | 0.783 | 0.12 (-1.94 – 2.19) | 0.906 | 0.06 (-3.71 – 3.83) | 0.976 |
| Current smoking | |  |  | 0.55 (-1.52 – 2.62) | 0.601 | 0.62 (-1.61 – 2.85) | 0.586 | 1.09 (-1.12 – 3.30) | 0.334 | 0.94 (-3.11 – 4.99) | 0.649 |
| BMI | |  |  | 0.37 (0.18 – 0.55) | **<0.001** | 0.39 (0.19 – 0.59) | **<0.001** | 0.38 (0.18 – 0.58) | **<0.001** | 0.51 (0.17 – 0.85) | **0.003** |
| Additives | Milk |  |  |  |  | 0.49 (-1.41 – 2.38) | 0.612 |  |  |  |  |
| Milk + Sugar |  |  |  |  | 1.25 (-2.80 – 5.30) | 0.544 |  |  |  |  |
| Milk + Sweetener |  |  |  |  | 4.29 (-0.54 – 9.11) | 0.081 |  |  |  |  |
| Sugar |  |  |  |  | -4.74 (-15.54 – 6.06) | 0.389 |  |  |  |  |
| Sweetener |  |  |  |  | 5.35 (-1.65 – 12.35) | 0.134 |  |  |  |  |
| Binary |  |  |  |  |  |  | 0.66 (-1.16 – 2.47) | 0.479 |  |  |

**Supplementary Table 63. Multivariable linear regression analysis for the association of moderate/high coffee consumption and heart rate for decaffeinated coffee.** Mild coffee consumption (<3 cups/d) served as the reference group. Adjustment was performed for: A) unadjusted; B) main cardiovascular risk factors; C) additional adjustment for additives (separately), D) additional adjustment for additives (binary), E) same adjustment as for group A, but only for subjects who consumed coffee without any additives. Abbreviations as in Table 1.

|  | | A)PQ interval | | B)PQ interval | | C)PQ interval | | D)PQ interval | | E)PQ interval | |
| --- | --- | --- | --- | --- | --- | --- | --- | --- | --- | --- | --- |
|  | | *Estimates* | *p* | *Estimates* | *p* | *Estimates* | *p* | *Estimates* | *p* | *Estimates* | *p* |
| Coffee consumption3-4 cups/day | | -3.50 (-8.17 – 1.17) | 0.141 | -1.20 (-6.19 – 3.80) | 0.639 | -0.63 (-5.84 – 4.58) | 0.811 | -0.85 (-6.10 – 4.40) | 0.751 | -11.09 (-20.40 – -1.77) | **0.020** |
| Coffee consumption > 4 cups/day | | -1.86 (-8.81 – 5.09) | 0.600 | -0.75 (-8.29 – 6.79) | 0.846 | 0.13 (-8.06 – 8.32) | 0.975 | -0.22 (-8.50 – 8.06) | 0.958 | -9.36 (-22.22 – 3.50) | 0.153 |
| Age | |  |  | 0.67 (0.39 – 0.95) | **<0.001** | 0.64 (0.34 – 0.94) | **<0.001** | 0.65 (0.35 – 0.95) | **<0.001** | 1.16 (0.60 – 1.72) | **<0.001** |
| Female | |  |  | -9.88 (-14.39 – -5.37) | **<0.001** | -9.63 (-14.39 – -4.86) | **<0.001** | -9.78 (-14.58 – -4.99) | **<0.001** | -17.08 (-25.54 – -8.62) | **<0.001** |
| Diabetes mellitus | |  |  | 2.40 (-5.17 – 9.97) | 0.534 | -0.44 (-8.70 – 7.83) | 0.917 | -0.64 (-8.99 – 7.72) | 0.881 | 3.42 (-12.16 – 19.01) | 0.665 |
| Arterial hypertension | |  |  | 1.22 (-4.07 – 6.50) | 0.652 | 1.65 (-3.92 – 7.22) | 0.560 | 1.44 (-4.15 – 7.04) | 0.613 | -1.14 (-10.94 – 8.67) | 0.819 |
| Current smoking | |  |  | -2.39 (-7.97 – 3.19) | 0.400 | -1.38 (-7.35 – 4.59) | 0.649 | -2.03 (-8.04 – 3.98) | 0.507 | -2.98 (-13.52 – 7.55) | 0.577 |
| BMI | |  |  | 0.34 (-0.17 – 0.84) | 0.192 | 0.39 (-0.16 – 0.93) | 0.163 | 0.34 (-0.21 – 0.89) | 0.222 | 0.04 (-0.84 – 0.92) | 0.924 |
| Additives | Milk |  |  |  |  | -2.13 (-7.20 – 2.95) | 0.410 |  |  |  |  |
| Milk + Sugar |  |  |  |  | -0.05 (-10.84 – 10.74) | 0.992 |  |  |  |  |
| Milk + Sweetener |  |  |  |  | -11.98 (-24.94 – 0.97) | 0.070 |  |  |  |  |
| Sugar |  |  |  |  | -11.91 (-43.80 – 19.99) | 0.464 |  |  |  |  |
| Sweetener |  |  |  |  | -18.23 (-36.15 – -0.32) | **0.046** |  |  |  |  |
| Binary |  |  |  |  |  |  | -2.89 (-7.82 – 2.05) | 0.251 |  |  |

**Supplementary Table 64. Multivariable linear regression analysis for the association of moderate/high coffee consumption and PQ interval for decaffeinated coffee.** Mild coffee consumption (<3 cups/d) served as the reference group. Adjustment was performed for: A) unadjusted; B) main cardiovascular risk factors; C) additional adjustment for additives (separately), D) additional adjustment for additives (binary), E) same adjustment as for group A, but only for subjects who consumed coffee without any additives. Abbreviations as in Table 1.

|  | | A)QRS interval | | B)QRS interval | | C)QRS interval | | D)QRS interval | | E)QRS interval | |
| --- | --- | --- | --- | --- | --- | --- | --- | --- | --- | --- | --- |
|  | | *Estimates* | *p* | *Estimates* | *p* | *Estimates* | *p* | *Estimates* | *p* | *Estimates* | *p* |
| Coffee consumption 3-4 cups/day | | -0.13 (-2.44 – 2.19) | 0.915 | 0.11 (-2.26 – 2.49) | 0.925 | 0.00 (-2.48 – 2.48) | 0.997 | -0.01 (-2.49 – 2.47) | 0.991 | 2.15 (-2.41 – 6.70) | 0.354 |
| Coffee consumption > 4 cups/day | | 0.33 (-3.17 – 3.83) | 0.852 | -0.96 (-4.60 – 2.68) | 0.605 | -1.43 (-5.39 – 2.52) | 0.477 | -1.96 (-5.93 – 2.00) | 0.331 | 1.43 (-5.05 – 7.90) | 0.665 |
| Age | |  |  | 0.13 (-0.00 – 0.27) | 0.055 | 0.09 (-0.06 – 0.23) | 0.233 | 0.10 (-0.04 – 0.24) | 0.161 | 0.18 (-0.09 – 0.46) | 0.195 |
| Female | |  |  | -8.56 (-10.71 – -6.40) | **<0.001** | -9.33 (-11.61 – -7.06) | **<0.001** | -9.10 (-11.37 – -6.83) | **<0.001** | -12.05 (-16.25 – -7.85) | **<0.001** |
| Diabetes mellitus | |  |  | -2.76 (-6.39 – 0.87) | 0.135 | -2.38 (-6.34 – 1.57) | 0.237 | -1.94 (-5.90 – 2.03) | 0.338 | -6.01 (-13.66 – 1.64) | 0.123 |
| Arterial hypertension | |  |  | -0.22 (-2.76 – 2.31) | 0.862 | -0.32 (-2.99 – 2.35) | 0.813 | -0.34 (-3.00 – 2.32) | 0.800 | -2.84 (-7.78 – 2.11) | 0.259 |
| Current smoking | |  |  | -0.27 (-2.95 – 2.41) | 0.843 | 0.21 (-2.65 – 3.07) | 0.887 | 0.37 (-2.48 – 3.23) | 0.797 | -3.20 (-8.51 – 2.11) | 0.237 |
| BMI | |  |  | 0.21 (-0.03 – 0.46) | 0.083 | 0.19 (-0.07 – 0.45) | 0.154 | 0.17 (-0.09 – 0.43) | 0.198 | 0.03 (-0.40 – 0.47) | 0.882 |
| Additives | **Milk** |  |  |  |  | -0.63 (-3.05 – 1.79) | 0.611 |  |  |  |  |
| **Milk + Sugar** |  |  |  |  | -4.64 (-9.85 – 0.58) | 0.081 |  |  |  |  |
| **Milk + Sweetener** |  |  |  |  | -3.25 (-9.51 – 3.01) | 0.308 |  |  |  |  |
| **Sugar** |  |  |  |  | -7.57 (-23.00 – 7.87) | 0.336 |  |  |  |  |
| **Sweetener** |  |  |  |  | 6.31 (-2.35 – 14.98) | 0.153 |  |  |  |  |
| **Binary** |  |  |  |  |  |  | -1.10 (-3.43 – 1.23) | 0.355 |  |  |

**Supplementary Table 65. Multivariable linear regression analysis for the association of moderate/high coffee consumption and QRS interval for decaffeinated coffee.** Mild coffee consumption (<3 cups/d) served as the reference group. Adjustment was performed for: A) unadjusted; B) main cardiovascular risk factors; C) additional adjustment for additives (separately), D) additional adjustment for additives (binary), E) same adjustment as for group A, but only for subjects who consumed coffee without any additives. Abbreviations as in Table 1.

|  | | A)QTc interval | | B)QTc interval | | C)QTc interval | | D)QTc interval | | E)Q Tc interval | |
| --- | --- | --- | --- | --- | --- | --- | --- | --- | --- | --- | --- |
|  | | *Estimates* | *p* | *Estimates* | *p* | *Estimates* | *p* | *Estimates* | *p* | *Estimates* | *p* |
| Coffee consumption 3-4 cups/day | | -9.11 (-13.63 – -4.60) | **<0.001** | -8.23 (-13.21 – -3.24) | **0.001** | -9.01 (-14.33 – -3.70) | **0.001** | -9.00 (-14.32 – -3.68) | **0.001** | -8.49 (-19.66 – 2.68) | 0.135 |
| Coffee consumption >4 cups/day | | -4.87 (-11.94 – 2.21) | 0.177 | -4.47 (-12.33 – 3.38) | 0.264 | -3.78 (-12.45 – 4.90) | 0.393 | -5.05 (-13.78 – 3.67) | 0.256 | -4.03 (-19.92 – 11.86) | 0.617 |
| Age | |  |  | 0.16 (-0.12 – 0.44) | 0.256 | 0.11 (-0.20 – 0.41) | 0.481 | 0.10 (-0.21 – 0.40) | 0.529 | -0.34 (-1.01 – 0.33) | 0.317 |
| Female | |  |  | 4.73 (0.19 – 9.27) | **0.041** | 4.29 (-0.61 – 9.18) | 0.086 | 4.61 (-0.27 – 9.50) | 0.064 | 7.72 (-2.51 – 17.95) | 0.138 |
| Diabetes mellitus | |  |  | 1.81 (-5.94 – 9.56) | 0.647 | 2.59 (-6.10 – 11.28) | 0.558 | 3.35 (-5.37 – 12.08) | 0.450 | 9.76 (-9.30 – 28.82) | 0.314 |
| Arterial hypertension | |  |  | 0.65 (-4.66 – 5.96) | 0.811 | 1.52 (-4.18 – 7.21) | 0.601 | 1.41 (-4.27 – 7.08) | 0.627 | 1.12 (-10.93 – 13.16) | 0.855 |
| Current smoking | |  |  | 2.36 (-3.28 – 8.01) | 0.411 | 2.90 (-3.25 – 9.06) | 0.355 | 3.90 (-2.25 – 10.05) | 0.214 | 3.91 (-8.77 – 16.58) | 0.544 |
| BMI | |  |  | 0.54 (0.03 – 1.05) | **0.036** | 0.57 (0.01 – 1.13) | **0.045** | 0.59 (0.04 – 1.15) | **0.037** | 0.67 (-0.37 – 1.72) | 0.205 |
| Additives | **Milk** |  |  |  |  | 0.32 (-4.87 – 5.50) | 0.905 |  |  |  |  |
| **Milk + Sugar** |  |  |  |  | 2.03 (-9.03 – 13.10) | 0.718 |  |  |  |  |
| **Milk + Sweetener** |  |  |  |  | -0.55 (-14.23 – 13.14) | 0.937 |  |  |  |  |
| **Sugar** |  |  |  |  | 3.80 (-28.37 – 35.97) | 0.817 |  |  |  |  |
| **Sweetener** |  |  |  |  | 11.37 (-8.74 – 31.48) | 0.267 |  |  |  |  |
| **Binary** |  |  |  |  |  |  | 0.55 (-4.46 – 5.57) | 0.828 |  |  |

**Supplementary Table 66. Multivariable linear regression analysis for the association of moderate/high coffee consumption and QTc interval for decaffeinated coffee.** Mild coffee consumption (<3 cups/d) served as the reference group. Adjustment was performed for: A) unadjusted; B) main cardiovascular risk factors; C) additional adjustment for additives (separately), D) additional adjustment for additives (binary), E) same adjustment as for group A, but only for subjects who consumed coffee without any additives. Abbreviations as in Table 1.

|  | | A)LVEF | | B)LVEF | | C)LVEF | | D)LVEF | | E)LVEF | |
| --- | --- | --- | --- | --- | --- | --- | --- | --- | --- | --- | --- |
|  | | *Estimates* | *p* | *Estimates* | *p* | *Estimates* | *p* | *Estimates* | *p* | *Estimates* | *p* |
| Coffee consumption 3-4 cups/day | | 0.24 (-0.69 – 1.18) | 0.609 | 0.33 (-0.69 – 1.36) | 0.524 | 0.34 (-0.76 – 1.43) | 0.546 | 0.33 (-0.77 – 1.43) | 0.555 | 0.43 (-1.51 – 2.36) | 0.662 |
| Coffee consumption > 4 cups/day | | -0.30 (-1.68 – 1.08) | 0.667 | -0.06 (-1.57 – 1.45) | 0.939 | -0.68 (-2.34 – 0.98) | 0.424 | -0.57 (-2.25 – 1.10) | 0.503 | -1.76 (-4.41 – 0.89) | 0.191 |
| Age | |  |  | 0.01 (-0.05 – 0.06) | 0.844 | 0.01 (-0.06 – 0.07) | 0.849 | 0.00 (-0.06 – 0.07) | 0.895 | 0.06 (-0.06 – 0.17) | 0.327 |
| Female | |  |  | 2.09 (1.16 – 3.01) | **<0.001** | 1.81 (0.82 – 2.81) | **<0.001** | 1.80 (0.80 – 2.80) | **<0.001** | 1.46 (-0.26 – 3.18) | 0.095 |
| Diabetes mellitus | |  |  | -2.25 (-3.98 – -0.52) | **0.011** | -2.16 (-4.08 – -0.24) | **0.028** | -2.21 (-4.14 – -0.28) | **0.025** | -4.00 (-8.20 – 0.19) | 0.061 |
| Arterial hypertension | |  |  | 0.18 (-0.88 – 1.23) | 0.744 | 0.05 (-1.09 – 1.19) | 0.933 | 0.04 (-1.09 – 1.17) | 0.944 | 1.23 (-0.74 – 3.20) | 0.221 |
| Current smoking | |  |  | 0.90 (-0.27 – 2.06) | 0.131 | 0.85 (-0.41 – 2.11) | 0.187 | 0.80 (-0.46 – 2.06) | 0.213 | -0.22 (-2.62 – 2.18) | 0.858 |
| BMI | |  |  | -0.09 (-0.20 – 0.02) | 0.094 | -0.06 (-0.18 – 0.06) | 0.308 | -0.05 (-0.17 – 0.06) | 0.357 | -0.14 (-0.35 – 0.07) | 0.195 |
| Additives | **Milk** |  |  |  |  | 0.20 (-0.86 – 1.25) | 0.716 |  |  |  |  |
| **Milk + Sugar** |  |  |  |  | 0.81 (-1.60 – 3.21) | 0.510 |  |  |  |  |
| **Milk + Sweetener** |  |  |  |  | 0.08 (-2.79 – 2.96) | 0.955 |  |  |  |  |
| **Sugar** |  |  |  |  | -1.96 (-8.02 – 4.11) | 0.526 |  |  |  |  |
| **Sweetener** |  |  |  |  | -0.48 (-4.84 – 3.88) | 0.828 |  |  |  |  |
| **Binary** |  |  |  |  |  |  | 0.24 (-0.79 – 1.27) | 0.645 |  |  |

**Supplementary Table 67. Multivariable linear regression analysis for the association of moderate/high coffee consumption and LVEF for decaffeinated coffee.** Mild coffee consumption (<3 cups/d) served as the reference group. Adjustment was performed for: A) unadjusted; B) main cardiovascular risk factors; C) additional adjustment for additives (separately), D) additional adjustment for additives (binary), E) same adjustment as for group A, but only for subjects who consumed coffee without any additives. Abbreviations as in Table 1.

|  | | A)LV mass index | | B)LV mass index | | C)LV mass index | | D)LV mass index | | E)LV mass index | |
| --- | --- | --- | --- | --- | --- | --- | --- | --- | --- | --- | --- |
|  | | *Estimates* | *p* | *Estimates* | *p* | *Estimates* | *p* | *Estimates* | *p* | *Estimates* | *p* |
| Coffee consumption 3-4 cups/day | | 1.15 (-2.78 – 5.09) | 0.566 | 0.18 (-3.47 – 3.83) | 0.922 | 0.50 (-3.35 – 4.35) | 0.799 | 0.41 (-3.47 – 4.30) | 0.835 | -0.04 (-6.59 – 6.50) | 0.990 |
| Coffee consumption >4 cups/day | | 0.63 (-5.01 – 6.27) | 0.826 | -2.46 (-7.77 – 2.85) | 0.364 | -3.08 (-9.02 – 2.87) | 0.310 | -3.90 (-9.85 – 2.05) | 0.198 | -1.10 (-10.05 – 7.86) | 0.809 |
| Age | |  |  | 0.30 (0.09 – 0.51) | **0.004** | 0.31 (0.09 – 0.53) | **0.006** | 0.30 (0.08 – 0.52) | **0.007** | 0.26 (-0.14 – 0.65) | 0.204 |
| Female | |  |  | -17.64 (-20.95 – -14.32) | **<0.001** | -17.45 (-21.00 – -13.90) | **<0.001** | -17.27 (-20.84 – -13.71) | **<0.001** | -19.35 (-25.22 – -13.48) | **<0.001** |
| Diabetes mellitus | |  |  | 2.10 (-3.85 – 8.05) | 0.488 | 2.36 (-4.17 – 8.89) | 0.478 | 1.57 (-5.04 – 8.18) | 0.641 | 20.94 (7.08 – 34.80) | **0.003** |
| Arterial hypertension | |  |  | 1.21 (-2.57 – 4.99) | 0.530 | 0.64 (-3.41 – 4.70) | 0.756 | 0.67 (-3.38 – 4.71) | 0.746 | 1.01 (-5.71 – 7.73) | 0.767 |
| Current smoking | |  |  | -0.33 (-4.47 – 3.81) | 0.874 | 0.78 (-3.71 – 5.26) | 0.733 | 0.37 (-4.14 – 4.88) | 0.871 | 3.84 (-4.45 – 12.13) | 0.361 |
| BMI | |  |  | 0.63 (0.25 – 1.02) | **0.001** | 0.68 (0.27 – 1.10) | **0.001** | 0.64 (0.22 – 1.06) | **0.003** | 0.60 (-0.11 – 1.32) | 0.098 |
| Additives | **Milk** |  |  |  |  | -2.29 (-6.02 – 1.45) | 0.229 |  |  |  |  |
| **Milk + Sugar** |  |  |  |  | -3.20 (-12.03 – 5.63) | 0.476 |  |  |  |  |
| **Milk + Sweetener** |  |  |  |  | -13.11 (-24.83 – -1.40) | **0.028** |  |  |  |  |
| **Sugar** |  |  |  |  | -20.53 (-56.49 – 15.43) | 0.262 |  |  |  |  |
| **Sweetener** |  |  |  |  | -10.06 (-22.36 – 2.25) | 0.109 |  |  |  |  |
| **Binary** |  |  |  |  |  |  | -3.08 (-6.76 – 0.59) | 0.100 |  |  |

**Supplementary Table 68 Multivariable linear regression analysis for the association of moderate/high coffee consumption and LV mass index for decaffeinated coffee.** Mild coffee consumption (<3 cups/d) served as the reference group. Adjustment was performed for: A) unadjusted; B) main cardiovascular risk factors; C) additional adjustment for additives (separately), D) additional adjustment for additives (binary), E) same adjustment as for group A, but only for subjects who consumed coffee without any additives. Abbreviations as in Table 1.

|  | | A)E/e‘ ratio | | B)E/e‘ ratio | | C)E/e‘ ratio | | D)E/e‘ ratio | | E)E/e‘ ratio | |
| --- | --- | --- | --- | --- | --- | --- | --- | --- | --- | --- | --- |
|  | | *Estimates* | *p* | *Estimates* | *p* | *Estimates* | *p* | *Estimates* | *p* | *Estimates* | *p* |
| Coffee consumption 3-4 cups/day | | -0.36 (-0.75 – 0.03) | 0.072 | -0.22 (-0.61 – 0.17) | 0.263 | -0.27 (-0.69 – 0.14) | 0.199 | -0.24 (-0.65 – 0.18) | 0.265 | -0.37 (-1.13 – 0.39) | 0.337 |
| Coffee consumption >4 cups/day | | -0.49 (-1.08 – 0.10) | 0.103 | -0.41 (-1.01 – 0.18) | 0.172 | -0.30 (-0.96 – 0.36) | 0.374 | -0.27 (-0.93 – 0.39) | 0.415 | -0.54 (-1.63 – 0.55) | 0.327 |
| Age | |  |  | 0.06 (0.03 – 0.08) | **<0.001** | 0.06 (0.03 – 0.08) | **<0.001** | 0.06 (0.03 – 0.08) | **<0.001** | 0.06 (0.01 – 0.10) | **0.017** |
| Female | |  |  | 0.71 (0.35 – 1.07) | **<0.001** | 0.79 (0.41 – 1.18) | **<0.001** | 0.81 (0.42 – 1.19) | **<0.001** | 1.05 (0.35 – 1.74) | **0.003** |
| Diabetes mellitus | |  |  | 0.12 (-0.52 – 0.75) | 0.717 | 0.05 (-0.66 – 0.75) | 0.900 | 0.04 (-0.66 – 0.75) | 0.905 | -0.01 (-1.47 – 1.45) | 0.990 |
| Arterial hypertension | |  |  | 0.54 (0.12 – 0.95) | **0.011** | 0.50 (0.06 – 0.95) | **0.027** | 0.54 (0.10 – 0.98) | **0.016** | 0.46 (-0.35 – 1.26) | 0.264 |
| Current smoking | |  |  | 0.64 (0.20 – 1.08) | **0.005** | 0.79 (0.31 – 1.27) | **0.001** | 0.79 (0.31 – 1.26) | **0.001** | 1.40 (0.46 – 2.34) | **0.004** |
| BMI | |  |  | 0.06 (0.02 – 0.10) | **0.003** | 0.06 (0.02 – 0.11) | **0.004** | 0.07 (0.02 – 0.11) | **0.002** | 0.16 (0.08 – 0.24) | **<0.001** |
| Additives | **Milk** |  |  |  |  | -0.17 (-0.58 – 0.24) | 0.419 |  |  |  |  |
|  | **Milk + Sugar** |  |  |  |  | -0.11 (-1.02 – 0.79) | 0.803 |  |  |  |  |
|  | **Milk + Sweetener** |  |  |  |  | 0.61 (-0.46 – 1.69) | 0.261 |  |  |  |  |
|  | **Sugar** |  |  |  |  | 0.04 (-2.23 – 2.31) | 0.975 |  |  |  |  |
|  | **Sweetener** |  |  |  |  | -0.45 (-2.09 – 1.18) | 0.587 |  |  |  |  |
|  | **Binary** |  |  |  |  |  |  | -0.12 (-0.52 – 0.27) | 0.546 |  |  |

**Supplementary Table 69 Multivariable linear regression analysis for the association of moderate/high coffee consumption and E/e’ ratio for decaffeinated coffee.** Mild coffee consumption (<3 cups/d) served as the reference group. Adjustment was performed for: A) unadjusted; B) main cardiovascular risk factors; C) additional adjustment for additives (separately), D) additional adjustment for additives (binary), E) same adjustment as for group A, but only for subjects who consumed coffee without any additives. Abbreviations as in Table 1.

|  | | A)TR Vmax in m/s | | B)TR Vmax in m/s | | C)TR Vmax in m/s | | D)TR Vmax in m/s | | E)TR Vmax in m/s | |
| --- | --- | --- | --- | --- | --- | --- | --- | --- | --- | --- | --- |
|  | | *Estimates* | *p* | *Estimates* | *p* | *Estimates* | *p* | *Estimates* | *p* | *Estimates* | *p* |
| Coffee consumption 3-4 cups/day | | 0.02 (-0.05 – 0.09) | 0.603 | 0.03 (-0.05 – 0.10) | 0.465 | 0.04 (-0.04 – 0.12) | 0.337 | 0.04 (-0.04 – 0.12) | 0.309 | -0.06 (-0.23 – 0.10) | 0.453 |
| Coffee consumption >4 cups/day | | 0.05 (-0.06 – 0.17) | 0.361 | 0.04 (-0.08 – 0.16) | 0.512 | 0.05 (-0.09 – 0.18) | 0.496 | 0.05 (-0.08 – 0.18) | 0.450 | 0.00 (-0.27 – 0.27) | 0.993 |
| Age | |  |  | 0.00 (0.00 – 0.01) | **0.045** | 0.00 (-0.00 – 0.01) | 0.071 | 0.00 (-0.00 – 0.01) | 0.058 | 0.01 (-0.01 – 0.02) | 0.257 |
| [Female | |  |  | -0.02 (-0.09 – 0.05) | 0.523 | -0.01 (-0.09 – 0.06) | 0.711 | -0.01 (-0.09 – 0.06) | 0.732 | -0.03 (-0.18 – 0.11) | 0.653 |
| Diabetes mellitus | |  |  | -0.07 (-0.19 – 0.05) | 0.229 | -0.06 (-0.19 – 0.06) | 0.318 | -0.07 (-0.20 – 0.05) | 0.257 | -0.28 (-0.64 – 0.08) | 0.130 |
| Arterial hypertension | |  |  | 0.10 (0.02 – 0.18) | **0.017** | 0.10 (0.02 – 0.19) | **0.021** | 0.10 (0.01 – 0.18) | **0.024** | 0.14 (-0.04 – 0.31) | 0.125 |
| Current smoking | |  |  | 0.11 (0.00 – 0.22) | **0.047** | 0.09 (-0.05 – 0.22) | 0.194 | 0.08 (-0.05 – 0.21) | 0.209 | 0.05 (-0.35 – 0.46) | 0.796 |
| BMI | |  |  | 0.00 (-0.00 – 0.01) | 0.307 | 0.01 (-0.00 – 0.01) | 0.220 | 0.01 (-0.00 – 0.01) | 0.202 | 0.01 (-0.01 – 0.03) | 0.273 |
| Additives | **Milk** |  |  |  |  | -0.08 (-0.16 – -0.01) | **0.030** |  |  |  |  |
| **Milk + Sugar** |  |  |  |  | -0.11 (-0.30 – 0.08) | 0.247 |  |  |  |  |
| **Milk+ Sweetener** |  |  |  |  | -0.07 (-0.29 – 0.15) | 0.545 |  |  |  |  |
| **Sweetener** |  |  |  |  | 0.36 (0.04 – 0.68) | **0.029** |  |  |  |  |
| **Binary** |  |  |  |  |  |  | -0.08 (-0.16 – -0.01) | **0.029** |  |  |

**Supplementary Table 70 Multivariable linear regression analysis for the association of moderate/high coffee consumption and TR Vmax in m/s for decaffeinated coffee.** Mild coffee consumption (<3 cups/d) served as the reference group. Adjustment was performed for: A) unadjusted; B) main cardiovascular risk factors; C) additional adjustment for additives (separately), D) additional adjustment for additives (binary), E) same adjustment as for group A, but only for subjects who consumed coffee without any additives. Abbreviations as in Table 1.

|  | | A)TAPSE in mm | | B)TAPSE in mm | | C)TAPSE in mm | | D)TAPSE in mm | | E)TAPSE in mm | |
| --- | --- | --- | --- | --- | --- | --- | --- | --- | --- | --- | --- |
|  | | *Estimates* | *p* | *Estimates* | *p* | *Estimates* | *p* | *Estimates* | *p* | *Estimates* | *p* |
| Coffee consumption 3-4 cups/day | | 0.60 (-0.30 – 1.51) | 0.192 | 0.44 (-0.57 – 1.45) | 0.393 | 0.43 (-0.64 – 1.50) | 0.428 | 0.38 (-0.69 – 1.44) | 0.489 | -0.72 (-2.56 – 1.11) | 0.437 |
| Coffee consumption >4 cups/day | | 1.69 (0.41 – 2.97) | **0.010** | 1.06 (-0.37 – 2.50) | 0.145 | 1.14 (-0.45 – 2.73) | 0.159 | 1.12 (-0.47 – 2.70) | 0.166 | 0.79 (-1.65 – 3.24) | 0.520 |
| Age | |  |  | -0.12 (-0.18 – -0.06) | **<0.001** | -0.14 (-0.20 – -0.08) | **<0.001** | -0.14 (-0.20 – -0.08) | **<0.001** | -0.19 (-0.30 – -0.09) | **<0.001** |
| Female | |  |  | -0.52 (-1.43 – 0.40) | 0.266 | -0.52 (-1.50 – 0.45) | 0.293 | -0.52 (-1.50 – 0.45) | 0.291 | -1.50 (-3.12 – 0.12) | 0.069 |
| Diabetes mellitus | |  |  | -1.17 (-2.88 – 0.55) | 0.181 | -0.52 (-2.48 – 1.44) | 0.604 | -0.48 (-2.47 – 1.50) | 0.634 | 1.01 (-2.63 – 4.66) | 0.583 |
| Arterial hypertension | |  |  | 0.53 (-0.52 – 1.59) | 0.318 | 0.41 (-0.71 – 1.53) | 0.472 | 0.37 (-0.75 – 1.48) | 0.517 | 0.51 (-1.36 – 2.38) | 0.589 |
| Current smoking | |  |  | -0.27 (-1.44 – 0.89) | 0.647 | -0.15 (-1.41 – 1.12) | 0.817 | -0.19 (-1.45 – 1.07) | 0.771 | -1.01 (-3.50 – 1.48) | 0.424 |
| BMI | |  |  | 0.05 (-0.05 – 0.16) | 0.311 | 0.10 (-0.02 – 0.21) | 0.095 | 0.09 (-0.02 – 0.20) | 0.114 | 0.03 (-0.17 – 0.23) | 0.767 |
| Additives | **Milk** |  |  |  |  | -0.26 (-1.31 – 0.79) | 0.627 |  |  |  |  |
| **Milk + Sugar** |  |  |  |  | -1.04 (-3.52 – 1.43) | 0.406 |  |  |  |  |
| **Milk + Sweetener** |  |  |  |  | -1.27 (-4.39 – 1.85) | 0.423 |  |  |  |  |
| **Sugar** |  |  |  |  | -0.05 (-5.23 – 5.14) | 0.986 |  |  |  |  |
| **Sweetener** |  |  |  |  | -0.86 (-4.34 – 2.62) | 0.628 |  |  |  |  |
| **Binary** |  |  |  |  |  |  | -0.35 (-1.37 – 0.68) | 0.506 |  |  |

**Supplementary Table 71 Multivariable linear regression analysis for the association of moderate/high coffee consumption and TAPSE in mm for decaffeinated coffee.** Mild coffee consumption (<3 cups/d) served as the reference group. Adjustment was performed for: A) unadjusted; B) main cardiovascular risk factors; C) additional adjustment for additives (separately), D) additional adjustment for additives (binary), E) same adjustment as for group A, but only for subjects who consumed coffee without any additives. Abbreviations as in Table 1.

|  | | A)LBB | | B)LBB | | C)LBB | | D)LBB | | E)LBB | |
| --- | --- | --- | --- | --- | --- | --- | --- | --- | --- | --- | --- |
|  | | *Odds Ratios* | *p* | *Odds Ratios* | *p* | *Odds Ratios* | *p* | *Odds Ratios* | *p* | *Odds Ratios* | *p* |
| Coffee consumption 3-4 cups/day | | 1.40 (0.57 – 3.30) | 0.445 | 1.65 (0.62 – 4.19) | 0.302 | 1.58 (0.54 – 4.42) | 0.384 | 1.46 (0.50 – 4.04) | 0.470 | 2.13 (0.44 – 11.45) | 0.346 |
| Coffee consumption >4 cups/day | | 2.83 (0.97 – 7.42) | **0.042** | 2.09 (0.54 – 6.78) | 0.243 | 2.11 (0.51 – 7.40) | 0.264 | 1.95 (0.47 – 6.83) | 0.316 | 4.53 (0.84 – 26.55) | 0.076 |
| Age | |  |  | 1.05 (0.99 – 1.12) | 0.088 | 1.03 (0.97 – 1.10) | 0.353 | 1.03 (0.97 – 1.10) | 0.289 | 1.00 (0.92 – 1.10) | 0.922 |
| Female | |  |  | 0.44 (0.17 – 1.03) | 0.065 | 0.28 (0.09 – 0.75) | **0.018** | 0.29 (0.09 – 0.77) | **0.019** | 0.28 (0.04 – 1.17) | 0.114 |
| Diabetes mellitus | |  |  | 0.34 (0.05 – 1.32) | 0.176 | 0.44 (0.06 – 1.86) | 0.329 | 0.44 (0.06 – 1.85) | 0.327 |  |  |
| Arterial hypertension | |  |  | 2.12 (0.66 – 9.55) | 0.254 | 1.79 (0.52 – 8.36) | 0.397 | 1.86 (0.55 – 8.62) | 0.360 | 1.75 (0.32 – 14.27) | 0.550 |
| Current smoking | |  |  | 1.26 (0.43 – 3.27) | 0.648 | 1.62 (0.53 – 4.38) | 0.363 | 1.44 (0.47 – 3.89) | 0.495 | 1.72 (0.39 – 6.65) | 0.444 |
| BMI | |  |  | 1.07 (0.98 – 1.16) | 0.127 | 1.08 (0.98 – 1.17) | 0.118 | 1.07 (0.97 – 1.17) | 0.157 | 1.03 (0.91 – 1.14) | 0.630 |
| Additives | **Milk** |  |  |  |  | 0.70 (0.28 – 1.79) | 0.457 |  |  |  |  |
| **Binary** |  |  |  |  |  |  | 0.55 (0.22 – 1.38) | 0.200 |  |  |

**Supplementary Table 72. Multivariable logistic regression analysis for the association of mild/moderate/high coffee consumption and LBBB for decaffeinated coffee.** Not daily coffee consumption (<1 cups/d) served as the reference group. Adjustment was performed for: A) unadjusted; B) main cardiovascular risk factors; C) additional adjustment for additives (separately), D) additional adjustment for additives (binary), E) same adjustment as for group A, but only for subjects who consumed coffee without any additives. Abbreviations as in Table 1.

|  | | A)AV_block | | B)AV_block | | C)AV block | | D)AV_block | | E)AV block | |
| --- | --- | --- | --- | --- | --- | --- | --- | --- | --- | --- | --- |
|  | | *Odds Ratios* | *p* | *Odds Ratios* | *p* | *Odds Ratios* | *p* | *Odds Ratios* | *p* | *Odds Ratios* | *p* |
| Coffee consumption 3-4 cups/day | | 0.50 (0.22 – 1.01) | 0.069 | 0.62 (0.25 – 1.36) | 0.259 | 0.67 (0.27 – 1.50) | 0.346 | 0.66 (0.26 – 1.47) | 0.328 | 0.30 (0.06 – 1.19) | 0.111 |
| Coffee consumption >4 cups/day | | 0.48 (0.11 – 1.36) | 0.226 | 0.76 (0.17 – 2.43) | 0.676 | 0.91 (0.20 – 3.07) | 0.891 | 0.85 (0.18 – 2.85) | 0.810 | 0.21 (0.01 – 1.45) | 0.178 |
| Age | |  |  | 1.10 (1.05 – 1.17) | **<0.001** | 1.10 (1.04 – 1.17) | **0.001** | 1.10 (1.04 – 1.17) | **0.001** | 1.10 (1.01 – 1.21) | **0.043** |
| Female | |  |  | 0.46 (0.23 – 0.92) | **0.030** | 0.48 (0.23 – 0.98) | **0.047** | 0.48 (0.23 – 0.98) | **0.047** | 0.10 (0.01 – 0.41) | **0.005** |
| Diabetes mellitus | |  |  | 1.79 (0.72 – 4.16) | 0.193 | 1.38 (0.47 – 3.58) | 0.532 | 1.35 (0.46 – 3.50) | 0.552 | 1.07 (0.13 – 6.42) | 0.944 |
| Arterial hypertension | |  |  | 0.83 (0.35 – 2.11) | 0.675 | 0.76 (0.31 – 1.99) | 0.557 | 0.81 (0.34 – 2.08) | 0.637 | 0.25 (0.05 – 1.09) | 0.066 |
| Current smoking | |  |  | 0.70 (0.23 – 1.77) | 0.485 | 0.88 (0.28 – 2.29) | 0.815 | 0.82 (0.26 – 2.12) | 0.704 | 0.96 (0.13 – 4.64) | 0.964 |
| BMI | |  |  | 1.00 (0.92 – 1.08) | 0.953 | 1.00 (0.91 – 1.08) | 0.951 | 0.99 (0.91 – 1.08) | 0.897 | 1.09 (0.94 – 1.24) | 0.212 |
| Additives | **Milk** |  |  |  |  | 0.81 (0.40 – 1.69) | 0.569 |  |  |  |  |
| **Milk + Sugar** |  |  |  |  | 0.58 (0.03 – 3.24) | 0.608 |  |  |  |  |
| **Binary** |  |  |  |  |  |  | 0.73 (0.36 – 1.51) | 0.391 |  |  |

**Supplementary Table 73. Multivariable logistic regression analysis for the association of mild/moderate/high coffee consumption and AV-block for decaffeinated coffee.** Not daily coffee consumption (<1 cups/d) served as the reference group. Adjustment was performed for: A) unadjusted; B) main cardiovascular risk factors; C) additional adjustment for additives (separately), D) additional adjustment for additives (binary), E) same adjustment as for group A, but only for subjects who consumed coffee without any additives. Abbreviations as in Table 1.

|  | | A)Atrial_fibrillation | | B)Atrial_fibrillation | | C)Atrial_fibrillation | | D)Atrial_fibrillation | | E)Atrial_fibrillation | |
| --- | --- | --- | --- | --- | --- | --- | --- | --- | --- | --- | --- |
|  | | *Odds Ratios* | *p* | *Odds Ratios* | *p* | *Odds Ratios* | *p* | *Odds Ratios* | *p* | *Odds Ratios* | *p* |
| Coffee consumption 3-4 cups/day | | 1.23 (0.68 – 2.19) | 0.479 | 1.90 (0.94 – 3.78) | 0.068 | 1.62 (0.78 – 3.33) | 0.188 | 1.62 (0.78 – 3.31) | 0.185 | 1.40 (0.46 – 4.21) | 0.549 |
| Coffee consumption >4 cups/day | | 0.71 (0.21 – 1.87) | 0.538 | 0.77 (0.17 – 2.55) | 0.700 | 0.54 (0.11 – 1.95) | 0.397 | 0.64 (0.13 – 2.22) | 0.522 | 0.58 (0.07 – 2.98) | 0.551 |
| Age | |  |  | 1.13 (1.07 – 1.19) | **<0.001** | 1.12 (1.06 – 1.19) | **<0.001** | 1.12 (1.07 – 1.19) | **<0.001** | 1.15 (1.06 – 1.27) | **0.001** |
| Female | |  |  | 0.37 (0.18 – 0.72) | **0.004** | 0.37 (0.18 – 0.73) | **0.006** | 0.37 (0.18 – 0.73) | **0.005** | 0.19 (0.05 – 0.59) | **0.007** |
| Diabetes mellitus | |  |  | 0.90 (0.36 – 2.06) | 0.816 | 0.88 (0.33 – 2.12) | 0.789 | 0.92 (0.35 – 2.19) | 0.852 | 0.90 (0.19 – 3.66) | 0.887 |
| Arterial hypertension | |  |  | 2.17 (0.80 – 7.63) | 0.167 | 1.97 (0.71 – 7.00) | 0.232 | 2.05 (0.74 – 7.26) | 0.204 |  |  |
| Current smoking | |  |  | 0.95 (0.38 – 2.15) | 0.911 | 0.92 (0.35 – 2.18) | 0.851 | 1.06 (0.42 – 2.45) | 0.898 | 0.73 (0.14 – 2.83) | 0.667 |
| BMI | |  |  | 1.08 (1.01 – 1.16) | **0.023** | 1.09 (1.01 – 1.17) | **0.024** | 1.09 (1.01 – 1.17) | **0.017** | 1.11 (1.00 – 1.23) | **0.045** |
| Additives | **Milk** |  |  |  |  | 0.58 (0.29 – 1.16) | 0.121 |  |  |  |  |
| **Milk + Sweetener** |  |  |  |  | 0.56 (0.03 – 3.35) | 0.602 |  |  |  |  |
| **Sweetener** |  |  |  |  | 2.62 (0.32 – 14.69) | 0.306 |  |  |  |  |
| **Binary** |  |  |  |  |  |  | 0.60 (0.31 – 1.17) | 0.128 |  |  |

**Supplementary Table 74. Multivariable logistic regression analysis for the association of mild/moderate/high coffee consumption and atrial fibrillation for decaffeinated coffee.** Not daily coffee consumption (<1 cups/d) served as the reference group. Adjustment was performed for: A) unadjusted; B) main cardiovascular risk factors; C) additional adjustment for additives (separately), D) additional adjustment for additives (binary), E) same adjustment as for group A, but only for subjects who consumed coffee without any additives. Abbreviations as in Table 1.

|  | | A)Diabetes | | B)Diabetes | | C)Diabetes | | D)Diabetes | | E)Diabetes | |
| --- | --- | --- | --- | --- | --- | --- | --- | --- | --- | --- | --- |
|  | | *Odds Ratios* | *p* | *Odds Ratios* | *p* | *Odds Ratios* | *p* | *Odds Ratios* | *p* | *Odds Ratios* | *p* |
| Coffee consumption 3-4 cups/day | | 0.93 (0.51 – 1.61) | 0.788 | 1.27 (0.66 – 2.37) | 0.456 | 1.45 (0.73 – 2.83) | 0.280 | 1.59 (0.80 – 3.11) | 0.176 | 2.62 (0.78 – 8.98) | 0.115 |
| Coffee consumption >4 cups/day | | 1.59 (0.75 – 3.15) | 0.201 | 1.74 (0.73 – 3.90) | 0.195 | 2.00 (0.74 – 5.03) | 0.151 | 2.21 (0.83 – 5.52) | 0.098 | 0.97 (0.12 – 5.34) | 0.974 |
| Age | |  |  | 1.06 (1.02 – 1.10) | **0.005** | 1.08 (1.03 – 1.13) | **0.001** | 1.08 (1.03 – 1.13) | **0.002** | 1.09 (1.00 – 1.20) | 0.055 |
| Female | |  |  | 0.41 (0.23 – 0.72) | **0.002** | 0.52 (0.28 – 0.94) | **0.032** | 0.54 (0.29 – 0.97) | **0.043** | 0.51 (0.15 – 1.56) | 0.249 |
| Arterial hypertension | |  |  | 2.97 (1.22 – 8.91) | **0.029** | 4.43 (1.52 – 18.90) | **0.017** | 4.47 (1.53 – 19.07) | **0.016** | 2.77 (0.45 – 53.74) | 0.357 |
| Current smoking | |  |  | 1.17 (0.59 – 2.24) | 0.638 | 1.26 (0.59 – 2.54) | 0.536 | 1.16 (0.54 – 2.38) | 0.684 | 0.29 (0.03 – 1.53) | 0.199 |
| BMI | |  |  | 1.16 (1.10 – 1.23) | **<0.001** | 1.14 (1.08 – 1.21) | **<0.001** | 1.15 (1.08 – 1.22) | **<0.001** | 1.21 (1.09 – 1.37) | **0.001** |
| Additives | **Milk** |  |  |  |  | 1.65 (0.85 – 3.35) | 0.147 |  |  |  |  |
| **Milk + Sugar** |  |  |  |  | 1.52 (0.22 – 6.45) | 0.610 |  |  |  |  |
| **Milk + Sweetener** |  |  |  |  | 3.07 (0.75 – 10.56) | 0.090 |  |  |  |  |
| **Sweetener** |  |  |  |  | 1.79 (0.21 – 10.07) | 0.543 |  |  |  |  |
| **Binary** |  |  |  |  |  |  | 1.73 (0.91 – 3.43) | 0.102 |  |  |

**Supplementary Table 75. Multivariable logistic regression analysis for the association of mild/moderate/high coffee consumption and diabetes for decaffeinated coffee.** Not daily coffee consumption (<1 cups/d) served as the reference group. Adjustment was performed for: A) unadjusted; B) main cardiovascular risk factors; C) additional adjustment for additives (separately), D) additional adjustment for additives (binary), E) same adjustment as for group A, but only for subjects who consumed coffee without any additives. Abbreviations as in Table 1.

|  | | A)Obesity | | B)Obesity | | C)Obesity | | D)Obesity | | E)Obesity | |
| --- | --- | --- | --- | --- | --- | --- | --- | --- | --- | --- | --- |
|  | | *Odds Ratios* | *p* | *Odds Ratios* | *p* | *Odds Ratios* | *p* | *Odds Ratios* | *p* | *Odds Ratios* | *p* |
| Coffee consumption 3-4 cups/day | | 0.62 (0.39 – 0.95) | **0.032** | 0.55 (0.33 – 0.90) | **0.021** | 0.57 (0.33 – 0.95) | **0.035** | 0.53 (0.30 – 0.90) | **0.022** | 0.48 (0.17 – 1.20) | 0.133 |
| Coffee consumption >4 cups/day | | 1.15 (0.64 – 1.97) | 0.634 | 1.07 (0.55 – 2.02) | 0.832 | 0.99 (0.48 – 1.97) | 0.987 | 0.99 (0.47 – 1.97) | 0.976 | 1.40 (0.47 – 3.93) | 0.533 |
| Age | |  |  | 0.97 (0.95 – 1.00) | **0.044** | 0.97 (0.95 – 1.00) | 0.058 | 0.98 (0.95 – 1.00) | 0.101 | 0.99 (0.94 – 1.04) | 0.681 |
| Female | |  |  | 1.30 (0.86 – 1.99) | 0.220 | 1.20 (0.77 – 1.88) | 0.428 | 1.20 (0.77 – 1.89) | 0.422 | 1.53 (0.70 – 3.39) | 0.289 |
| Diabetes mellitus | |  |  | 4.49 (2.57 – 7.87) | **<0.001** | 4.39 (2.37 – 8.14) | **<0.001** | 4.41 (2.37 – 8.18) | **<0.001** | 10.69 (3.44 – 37.02) | **<0.001** |
| Arterial hypertension | |  |  | 3.52 (2.01 – 6.44) | **<0.001** | 3.40 (1.88 – 6.45) | **<0.001** | 3.10 (1.72 – 5.87) | **<0.001** | 6.99 (2.16 – 32.25) | **0.004** |
| Current smoking | |  |  | 1.49 (0.91 – 2.41) | 0.108 | 1.58 (0.93 – 2.64) | 0.084 | 1.61 (0.94 – 2.71) | 0.074 | 1.56 (0.61 – 3.82) | 0.335 |
| Additives | **Milk** |  |  |  |  | 0.74 (0.46 – 1.19) | 0.217 |  |  |  |  |
| **Milk + Sugar** |  |  |  |  | 0.97 (0.33 – 2.51) | 0.956 |  |  |  |  |
| **Milk + Sweetener** |  |  |  |  | 0.78 (0.22 – 2.34) | 0.677 |  |  |  |  |
| **Sugar** |  |  |  |  | 1.38 (0.06 – 12.16) | 0.789 |  |  |  |  |
| **Sweetener** |  |  |  |  | 2.85 (0.70 – 10.91) | 0.127 |  |  |  |  |
| **Binary** |  |  |  |  |  |  | 0.76 (0.49 – 1.21) | 0.246 |  |  |

**Supplementary Table 76. Multivariable logistic regression analysis for the association of mild/moderate/high coffee consumption and obesity for decaffeinated coffee.** Not daily coffee consumption (<1 cups/d) served as the reference group. Adjustment was performed for: A) unadjusted; B) main cardiovascular risk factors; C) additional adjustment for additives (separately), D) additional adjustment for additives (binary), E) same adjustment as for group A, but only for subjects who consumed coffee without any additives. Abbreviations as in Table 1.

|  | | A)CAD | | B)CAD | | C)CAD | | D)CAD | | E)CAD | |
| --- | --- | --- | --- | --- | --- | --- | --- | --- | --- | --- | --- |
|  | | *Odds Ratios* | *p* | *Odds Ratios* | *p* | *Odds Ratios* | *p* | *Odds Ratios* | *p* | *Odds Ratios* | *p* |
| Coffee consumption 3-4 cups/day | | 0.55 (0.28 – 1.00) | 0.059 | 0.73 (0.35 – 1.46) | 0.385 | 0.66 (0.30 – 1.38) | 0.286 | 0.69 (0.32 – 1.42) | 0.330 | 0.61 (0.16 – 2.12) | 0.456 |
| Coffee consumption >4 cups/day | | 0.87 (0.35 – 1.93) | 0.754 | 0.99 (0.32 – 2.71) | 0.989 | 0.59 (0.15 – 1.87) | 0.401 | 0.85 (0.24 – 2.54) | 0.779 | 0.74 (0.12 – 3.52) | 0.712 |
| Age | |  |  | 1.08 (1.03 – 1.13) | **0.001** | 1.08 (1.03 – 1.14) | **0.001** | 1.09 (1.04 – 1.14) | **0.001** | 1.06 (0.98 – 1.16) | 0.155 |
| Female | |  |  | 0.15 (0.06 – 0.30) | **<0.001** | 0.16 (0.07 – 0.33) | **<0.001** | 0.17 (0.07 – 0.35) | **<0.001** | 0.14 (0.03 – 0.49) | **0.005** |
| Diabetes mellitus | |  |  | 1.81 (0.83 – 3.87) | 0.131 | 1.33 (0.54 – 3.15) | 0.529 | 1.29 (0.53 – 2.99) | 0.560 | 5.03 (0.93 – 29.66) | 0.064 |
| Arterial hypertension | |  |  | 3.98 (1.48 – 13.90) | **0.013** | 3.25 (1.18 – 11.47) | **0.037** | 3.57 (1.32 – 12.49) | **0.023** | 5.67 (0.94 – 109.91) | 0.115 |
| Current smoking | |  |  | 1.22 (0.55 – 2.60) | 0.611 | 1.32 (0.55 – 2.98) | 0.515 | 1.30 (0.56 – 2.86) | 0.531 | 2.51 (0.67 – 9.23) | 0.163 |
| BMI | |  |  | 0.98 (0.91 – 1.05) | 0.638 | 0.99 (0.91 – 1.07) | 0.796 | 1.00 (0.92 – 1.07) | 0.930 | 0.91 (0.77 – 1.03) | 0.185 |
| Additives | **Milk** |  |  |  |  | 0.85 (0.43 – 1.70) | 0.635 |  |  |  |  |
| **Milk + Sugar** |  |  |  |  | 0.38 (0.02 – 2.31) | 0.384 |  |  |  |  |
| **Milk + Sweetener** |  |  |  |  | 0.37 (0.02 – 2.13) | 0.359 |  |  |  |  |
| **Sweetener** |  |  |  |  | 8.14 (1.10 – 57.95) | **0.035** |  |  |  |  |
| **Binary** |  |  |  |  |  |  | 0.88 (0.46 – 1.73) | 0.714 |  |  |

**Supplementary Table 77 Multivariable logistic regression analysis for the association of mild/moderate/high coffee consumption and CAD for decaffeinated coffee.** Not daily coffee consumption (<1 cups/d) served as the reference group. Adjustment was performed for: A) unadjusted; B) main cardiovascular risk factors; C) additional adjustment for additives (separately), D) additional adjustment for additives (binary), E) same adjustment as for group A, but only for subjects who consumed coffee without any additives. Abbreviations as in Table 1.

|  | | A)Heart failure | | B)Heart failure | | C)Heart failure | | D)Heart failure | | E)Heart failure | |
| --- | --- | --- | --- | --- | --- | --- | --- | --- | --- | --- | --- |
|  | | *Odds Ratios* | *p* | *Odds Ratios* | *p* | *Odds Ratios* | *p* | *Odds Ratios* | *p* | *Odds Ratios* | *p* |
| Coffee consumption 3-4 cups/day | | 0.35 (0.08 – 1.06) | 0.096 | 0.35 (0.05 – 1.37) | 0.184 | 0.39 (0.06 – 1.53) | 0.231 | 0.32 (0.05 – 1.27) | 0.154 | 0.85 (0.04 – 8.48) | 0.895 |
| Coffee consumption >4 cups/day | | 1.33 (0.37 – 3.79) | 0.619 | 1.95 (0.47 – 6.79) | 0.317 | 2.00 (0.46 – 7.52) | 0.322 | 1.25 (0.24 – 5.06) | 0.766 | 2.64 (0.20 – 27.81) | 0.420 |
| Age | |  |  | 1.10 (1.02 – 1.21) | **0.025** | 1.10 (1.02 – 1.22) | **0.030** | 1.10 (1.01 – 1.21) | **0.040** | 1.27 (1.05 – 1.66) | **0.037** |
| Female | |  |  | 0.53 (0.18 – 1.41) | 0.211 | 0.68 (0.23 – 1.88) | 0.459 | 0.65 (0.22 – 1.81) | 0.411 | 1.39 (0.20 – 9.81) | 0.726 |
| Diabetes mellitus | |  |  | 3.93 (1.20 – 11.81) | **0.017** | 3.19 (0.87 – 10.30) | 0.061 | 4.23 (1.18 – 13.60) | **0.018** | 8.42 (0.33 – 120.84) | 0.120 |
| Arterial hypertension | |  |  | 3.63 (0.65 – 68.35) | 0.230 | 4.80 (0.81 – 93.74) | 0.155 | 3.47 (0.61 – 65.71) | 0.249 |  |  |
| Current smoking | |  |  | 1.81 (0.50 – 5.83) | 0.336 | 1.51 (0.39 – 5.11) | 0.526 | 2.42 (0.64 – 8.32) | 0.167 | 15.27 (1.58 – 224.84) | **0.025** |
| BMI | |  |  | 1.09 (0.98 – 1.20) | 0.087 | 1.08 (0.95 – 1.21) | 0.215 | 1.07 (0.95 – 1.19) | 0.272 | 1.05 (0.88 – 1.24) | 0.545 |
| Additives | **Milk** |  |  |  |  | 0.95 (0.30 – 3.12) | 0.927 |  |  |  |  |
| **Milk + Sugar** |  |  |  |  | 4.45 (0.51 – 29.11) | 0.132 |  |  |  |  |
| **Milk + Sweetener** |  |  |  |  | 1.43 (0.06 – 11.61) | 0.772 |  |  |  |  |
| **Sweetener** |  |  |  |  | 6.06 (0.22 – 90.94) | 0.208 |  |  |  |  |
| **Binary** |  |  |  |  |  |  | 1.03 (0.35 – 3.25) | 0.960 |  |  |

**Supplementary Table 78. Multivariable logistic regression analysis for the association of mild/moderate/high coffee consumption and heart failure for decaffeinated coffee.** Not daily coffee consumption (<1 cups/d) served as the reference group. Adjustment was performed for: A) unadjusted; B) main cardiovascular risk factors; C) additional adjustment for additives (separately), D) additional adjustment for additives (binary), E) same adjustment as for group A, but only for subjects who consumed coffee without any additives. Abbreviations as in Table 1.

|  | | A)HF(m)rEF | | B)HF(m)rEF | | C)HF(m)rEF | | D)HF(m)rEF | |
| --- | --- | --- | --- | --- | --- | --- | --- | --- | --- |
|  | | *Odds Ratios* | *p* | *Odds Ratios* | *p* | *Odds Ratios* | *p* | *Odds Ratios* | *p* |
| Coffee consumption 3-4 cups/day | | 0.51 (0.12 – 1.67) | 0.313 | 0.48 (0.07 – 2.00) | 0.368 | 0.54 (0.08 – 2.33) | 0.457 | 0.45 (0.06 – 1.88) | 0.322 |
| Coffee consumption >4 cups/day | | 1.43 (0.32 – 4.73) | 0.594 | 2.30 (0.45 – 9.51) | 0.270 | 2.52 (0.44 – 11.67) | 0.254 | 1.37 (0.18 – 6.81) | 0.723 |
| Age | |  |  | 1.09 (1.00 – 1.21) | 0.060 | 1.10 (1.01 – 1.23) | 0.061 | 1.09 (1.00 – 1.22) | 0.077 |
| Female | |  |  | 0.22 (0.05 – 0.74) | **0.025** | 0.26 (0.06 – 0.92) | 0.054 | 0.26 (0.05 – 0.89) | **0.048** |
| Diabetes mellitus | |  |  | 4.05 (1.06 – 14.01) | **0.031** | 3.31 (0.75 – 12.76) | 0.091 | 4.69 (1.10 – 17.62) | **0.025** |
| Arterial hypertension | |  |  | 2.63 (0.44 – 50.61) | 0.377 | 3.74 (0.58 – 75.47) | 0.244 | 2.45 (0.40 – 47.62) | 0.418 |
| Current smoking | |  |  | 1.19 (0.23 – 4.67) | 0.818 | 0.83 (0.14 – 3.62) | 0.821 | 1.68 (0.32 – 7.16) | 0.499 |
| BMI | |  |  | 1.07 (0.95 – 1.21) | 0.249 | 1.04 (0.89 – 1.20) | 0.638 | 1.02 (0.88 – 1.18) | 0.746 |
| Additives | **Milk** |  |  |  |  | 1.09 (0.29 – 4.58) | 0.895 |  |  |
| **Milk + Sugar** |  |  |  |  | 7.27 (0.74 – 60.12) | 0.066 |  |  |
| **Milk + Sweetener** |  |  |  |  | 3.05 (0.14 – 27.76) | 0.367 |  |  |
| **Sweetener** |  |  |  |  | 11.22 (0.37 – 217.40) | 0.112 |  |  |
| **Binary** |  |  |  |  |  |  | 1.30 (0.38 – 5.14) | 0.685 |

**Supplementary Table 79. Multivariable logistic regression analysis for the association of mild/moderate/high coffee consumption and HF(m)rEF for decaffeinated coffee.** Not daily coffee consumption (<1 cups/d) served as the reference group. Adjustment was performed for: A) unadjusted; B) main cardiovascular risk factors; C) additional adjustment for additives (separately), D) additional adjustment for additives (binary). Abbreviations as in Table 1.

|  | COFFEE CONSUMPTION | | | |  |
| --- | --- | --- | --- | --- | --- |
|  | **Overall** | **< 3 cups/day** | **3-4 cups/day** | **> 4 cups/day** | **p-values** |
| N (%) | 4610 | 3099 | 1148 | 363 |  |
| DEMOGRAPHICS + BIOLOGICAL DATA | | | | | |
| Age | 62.0 [55.0, 69.0] | 63.0 [56.0, 70.0] | 59.0 [53.0, 66.2] | 59.0 [52.0, 65.0] | <0.001 |
| BMI kg/m2 | 25.3 [22.6, 28.8] | 25.2 [22.6, 28.7] | 25.3 [22.7, 28.9] | 26.1 [23.1, 29.8] | 0.028 |
| Smoking current | 873 ( 19.0) | 430 ( 14.0) | 293 ( 25.6) | 150 ( 41.3) | <0.001 |
| CARDIOVASCULAR DISEASES | | | | | |
| Arterial hypertension | 2598 ( 59.0) | 1851 ( 62.3) | 575 ( 52.8) | 172 ( 49.7) | <0.001 |
| Diabetes mellitus | 262 (  6.2) | 187 (  6.6) | 55 (  5.3) | 20 (  5.9) | 0.319 |
| Myocardial_infarction | 53 (  1.2) | 37 (  1.2) | 12 (  1.0) | 4 (  1.1) | 0.915 |
| CAD | 132 (  4.0) | 97 (  4.4) | 28 (  3.4) | 7 (  2.7) | 0.227 |
| PAD | 428 ( 20.8) | 280 ( 20.4) | 110 ( 21.0) | 38 ( 22.9) | 0.746 |
| LABORATORIES | | | | | |
| Cholesterol, mg/dl | 218.0 [192.0, 246.0] | 218.0 [191.0, 245.5] | 217.0 [192.0, 244.0] | 216.5 [193.8, 251.2] | 0.775 |
| LDL, mg/dl | 124.0 [100.0, 149.0] | 123.0 [99.0, 148.0] | 124.0 [100.0, 148.0] | 126.0 [107.0, 154.0] | 0.078 |
| HDL, mg/dl | 71.0 [59.0, 84.0] | 72.0 [59.0, 85.0] | 71.0 [60.0, 83.0] | 68.0 [55.8, 80.0] | <0.001 |
| NT-proBNP, g/dl | 96.0 [57.0, 164.0] | 101.0 [60.0, 171.0] | 86.0 [53.2, 146.0] | 91.0 [49.0, 149.0] | <0.001 |
| Hemoglobin, g/dl | 13.7 [13.2, 14.3] | 13.7 [13.2, 14.3] | 13.7 [13.2, 14.3] | 13.9 [13.3, 14.4] | 0.053 |
| MEDICATION | | | | | |
| ACEi/ ARBs | 806 ( 18.2) | 572 ( 19.2) | 182 ( 16.7) | 52 ( 14.9) | 0.044 |
| Beta Blockers | 694 ( 15.7) | 507 ( 17.0) | 140 ( 12.8) | 47 ( 13.4) | 0.003 |
| Diuretics | 84 (  1.9) | 60 (  2.0) | 17 (  1.6) | 7 (  2.0) | 0.636 |
| Lipid modifying drugs | 600 ( 13.6) | 428 ( 14.3) | 140 ( 12.8) | 32 (  9.1) | 19 |
| ADDTIVES | | | | | |
| Milk | 3152 ( 72.0) | 2108 ( 73.3) | 800 ( 70.1) | 244 ( 67.4) | 0.016 |
| Sugar | 439 ( 10.0) | 303 ( 10.5) | 101 (  8.8) | 35 (  9.7) | 0.266 |
| Honey | 31 (  0.7) | 26 (  0.9) | 5 (  0.4) | 0 (  0.0) | 0.069 |
| Sweetener | 215 (  4.9) | 143 (  5.0) | 53 (  4.6) | 19 (  5.2) | 0.865 |
| No additives | 1378 ( 31.5) | 841 ( 29.2) | 393 ( 34.4) | 144 ( 39.8) | <0.001 |
| BLACK/GREEN TEA | | | | | |
| Never | 745 ( 16.3) | 469 ( 15.3) | 214 ( 18.8) | 62 ( 17.3) | <0.001 |
| 1-3/week | 2017 ( 44.2) | 1235 ( 40.2) | 585 ( 51.4) | 197 ( 54.9) | <0.001 |
| >4/week | 1806 ( 39.5) | 1367 ( 44.5) | 339 ( 29.8) | 100 ( 27.9) | <0.001 |
| CARBONATED DRINKS |  |  |  |  | 0.003 |
| Never | 2611 ( 57.1) | 1802 ( 58.7) | 629 ( 55.1) | 180 ( 50.3) |  |
| 1-3/week | 1789 ( 39.1) | 1161 ( 37.8) | 472 ( 41.4) | 156 ( 43.6) |  |
| ≥4/week | 171 (  3.7) | 109 (  3.5) | 40 (  3.5) | 22 (  6.1) |  |

**Supplementary Table 80. Baseline characteristics** **of the study population only female.** Continuous variables are presented as median and interquartile range, and categorical variables are presented as absolute numbers and percentages. Abbreviations as in Table 1.

|  | | A)HDL | | B)HDL | | C)HDL | | D)HDL | | E)HDL | | F)HDL | |
| --- | --- | --- | --- | --- | --- | --- | --- | --- | --- | --- | --- | --- | --- |
|  | | *Estimates* | *p* | *Estimates* | *p* | *Estimates* | *p* | *Estimates* | *p* | *Estimates* | *p* | *Estimates* | *p* |
| Coffee consumption 3-4 cups/day | | -0.23 (-1.53 – 1.07) | 0.730 | 1.01 (-0.28 – 2.30) | 0.126 | 0.66 (-0.70 – 2.02) | 0.341 | 0.69 (-0.67 – 2.06) | 0.318 | 1.05 (-0.25 – 2.34) | 0.113 | -0.44 (-3.07 – 2.20) | 0.746 |
| Coffee consumption >4 cups/day | | -4.09 (-6.17 – -2.01) | **<0.001** | -0.35 (-2.44 – 1.74) | 0.742 | -1.01 (-3.22 – 1.20) | 0.370 | -1.02 (-3.24 – 1.20) | 0.368 | -0.39 (-2.49 – 1.70) | 0.712 | -1.22 (-5.20 – 2.76) | 0.548 |
| Age | |  |  | 0.15 (0.08 – 0.22) | **<0.001** | 0.09 (0.02 – 0.17) | **0.019** | 0.10 (0.02 – 0.18) | **0.010** | 0.16 (0.09 – 0.23) | **<0.001** | 0.13 (-0.03 – 0.28) | 0.111 |
| Diabetes mellitus | |  |  | -6.33 (-8.73 – -3.94) | **<0.001** | -6.69 (-9.20 – -4.18) | **<0.001** | -6.76 (-9.29 – -4.22) | **<0.001** | -5.86 (-8.29 – -3.43) | **<0.001** | -6.70 (-11.64 – -1.75) | **0.008** |
| Arterial hypertension | |  |  | -0.39 (-1.61 – 0.84) | 0.536 | -0.06 (-1.35 – 1.23) | 0.924 | 0.02 (-1.28 – 1.32) | 0.974 | -0.28 (-1.50 – 0.95) | 0.657 | -0.88 (-3.44 – 1.67) | 0.497 |
| Current smoking | |  |  | -7.03 (-8.46 – -5.60) | **<0.001** | -6.97 (-8.51 – -5.44) | **<0.001** | -6.98 (-8.52 – -5.44) | **<0.001** | -6.92 (-8.36 – -5.49) | **<0.001** | -7.31 (-10.34 – -4.28) | **<0.001** |
| BMI | |  |  | -1.45 (-1.57 – -1.34) | **<0.001** | -1.45 (-1.58 – -1.33) | **<0.001** | -1.47 (-1.59 – -1.34) | **<0.001** | -1.45 (-1.57 – -1.33) | **<0.001** | -1.45 (-1.69 – -1.20) | **<0.001** |
| Additives | **Milk** |  |  |  |  | -2.67 (-4.01 – -1.33) | **<0.001** |  |  |  |  |  |  |
|  | **Milk + Sugar** |  |  |  |  | -4.19 (-6.62 – -1.77) | **0.001** |  |  |  |  |  |  |
|  | **Milk + Sweetener** |  |  |  |  | -4.64 (-7.95 – -1.33) | **0.006** |  |  |  |  |  |  |
|  | **Sugar** |  |  |  |  | -9.34 (-14.78 – -3.91) | **0.001** |  |  |  |  |  |  |
|  | **Sweetener** |  |  |  |  | -2.02 (-7.33 – 3.28) | 0.455 |  |  |  |  |  |  |
|  | **Binary** |  |  |  |  |  |  | -3.00 (-4.32 – -1.69) | **<0.001** |  |  |  |  |
| Lipid lowering drugs | |  |  |  |  |  |  |  |  | -1.53 (-3.22 – 0.17) | 0.078 |  |  |

**Supplementary Table 83. Multivariable linear regression analysis for the association of mild/moderate/high coffee consumption and HDL (only female).** “Not daily” coffee consumption (<1 cups/d) served as the reference group. Adjustment was performed for: A) unadjusted; B) main cardiovascular risk factors; C) additional adjustment for additives (separately), D) additional adjustment for additives (binary), E) additional adjustment for lipid lowering drugs, F) same adjustment as for group A, but only for subjects who consumed coffee without any additives. Abbreviations as in Table 1

|  | | A)NT pro-BNP | | B)NT pro-BNP | | C)NT pro-BNP | | D)NT pro-BNP | | E)NT pro-BNP | |
| --- | --- | --- | --- | --- | --- | --- | --- | --- | --- | --- | --- |
|  | | *Estimates* | *p* | *Estimates* | *p* | *Estimates* | *p* | *Estimates* | *p* | *Estimates* | *p* |
| Coffee consumption 3-4 cups/day | | -0.13 (-0.19 – -0.08) | **<0.001** | -0.03 (-0.09 – 0.02) | 0.261 | -0.05 (-0.11 – 0.01) | 0.122 | -0.05 (-0.10 – 0.01) | 0.136 | 0.01 (-0.11 – 0.12) | 0.929 |
| Coffee consumption >4 cups/day | | -0.15 (-0.25 – -0.06) | **0.001** | -0.03 (-0.12 – 0.06) | 0.553 | -0.03 (-0.12 – 0.07) | 0.606 | -0.02 (-0.12 – 0.07) | 0.649 | -0.11 (-0.28 – 0.06) | 0.205 |
| Age | |  |  | 0.04 (0.03 – 0.04) | **<0.001** | 0.04 (0.03 – 0.04) | **<0.001** | 0.04 (0.03 – 0.04) | **<0.001** | 0.03 (0.03 – 0.04) | **<0.001** |
| Diabetes mellitus | |  |  | -0.14 (-0.24 – -0.03) | **0.010** | -0.14 (-0.25 – -0.03) | **0.013** | -0.12 (-0.23 – -0.01) | **0.031** | -0.16 (-0.37 – 0.05) | 0.134 |
| Arterial hypertension | |  |  | 0.18 (0.12 – 0.23) | **<0.001** | 0.19 (0.13 – 0.24) | **<0.001** | 0.19 (0.13 – 0.24) | **<0.001** | 0.26 (0.15 – 0.37) | **<0.001** |
| Current smoking | |  |  | 0.00 (-0.06 – 0.07) | 0.878 | -0.02 (-0.08 – 0.05) | 0.641 | -0.02 (-0.08 – 0.05) | 0.636 | 0.06 (-0.07 – 0.18) | 0.391 |
| BMI | |  |  | -0.01 (-0.02 – -0.01) | **<0.001** | -0.01 (-0.02 – -0.01) | **<0.001** | -0.01 (-0.02 – -0.01) | **<0.001** | -0.02 (-0.03 – -0.01) | **0.001** |
| Additives | **Milk** |  |  |  |  | -0.02 (-0.08 – 0.04) | 0.551 |  |  |  |  |
|  | **Milk + Sugar** |  |  |  |  | 0.02 (-0.09 – 0.13) | 0.706 |  |  |  |  |
|  | **Milk + Sweetener** |  |  |  |  | -0.06 (-0.21 – 0.08) | 0.381 |  |  |  |  |
|  | **Sugar** |  |  |  |  | -0.16 (-0.40 – 0.08) | 0.186 |  |  |  |  |
|  | **Sweetener** |  |  |  |  | 0.01 (-0.22 – 0.24) | 0.919 |  |  |  |  |
|  | **Binary** |  |  |  |  |  |  | -0.02 (-0.08 – 0.04) | 0.515 |  |  |

**Supplementary Table 84. Multivariable linear regression analysis for the association of mild/moderate/high coffee consumption and NT proBNP (only female).** “Not daily” coffee consumption (<1 cups/d) served as the reference group. Adjustment was performed for: A) unadjusted; B) main cardiovascular risk factors; C) additional adjustment for additives (separately), D) additional adjustment for additives (binary), E) additional adjustment for lipid lowering drugs, F) same adjustment as for group A, but only for subjects who consumed coffee without any additives. Abbreviations as in Table 1

|  | | A)SBP | | B)SBP | | C)SBP | | D)SBP | | E)SBP | |
| --- | --- | --- | --- | --- | --- | --- | --- | --- | --- | --- | --- |
|  | | *Estimates* | *p* | *Estimates* | *p* | *Estimates* | *p* | *Estimates* | *p* | *Estimates* | *p* |
| Coffee consumption 3-4 cups/day | | -4.67 (-6.25 – -3.09) | **<0.001** | -1.22 (-2.74 – 0.29) | 0.114 | -1.73 (-3.34 – -0.13) | **0.034** | -1.76 (-3.36 – -0.15) | **0.032** | -0.35 (-3.53 – 2.82) | 0.826 |
| Coffee consumption >4 cups/day | | -5.89 (-8.39 – -3.38) | **<0.001** | -2.00 (-4.44 – 0.44) | 0.108 | -2.77 (-5.37 – -0.18) | **0.036** | -2.70 (-5.30 – -0.10) | **0.042** | -5.94 (-10.72 – -1.15) | **0.015** |
| Age | |  |  | 1.03 (0.96 – 1.11) | **<0.001** | 1.01 (0.92 – 1.09) | **<0.001** | 1.01 (0.92 – 1.09) | **<0.001** | 0.99 (0.81 – 1.17) | **<0.001** |
| Diabetes mellitus | |  |  | 4.00 (1.20 – 6.81) | **0.005** | 4.13 (1.16 – 7.09) | **0.006** | 4.19 (1.20 – 7.18) | **0.006** | -0.41 (-6.39 – 5.57) | 0.893 |
| Current smoking | |  |  | -2.56 (-4.24 – -0.88) | **0.003** | -2.99 (-4.80 – -1.18) | **0.001** | -3.04 (-4.86 – -1.23) | **0.001** | -4.73 (-8.38 – -1.07) | **0.011** |
| BMI | |  |  | 0.72 (0.59 – 0.85) | **<0.001** | 0.67 (0.53 – 0.81) | **<0.001** | 0.67 (0.53 – 0.81) | **<0.001** | 0.63 (0.35 – 0.92) | **<0.001** |
| Additives | **Milk** |  |  |  |  | -0.94 (-2.52 – 0.64) | 0.243 |  |  |  |  |
|  | **Milk + Sugar** |  |  |  |  | -0.76 (-3.61 – 2.09) | 0.603 |  |  |  |  |
|  | **Milk + Sweetener** |  |  |  |  | -0.77 (-4.67 – 3.14) | 0.699 |  |  |  |  |
|  | **Sugar** |  |  |  |  | -0.65 (-7.04 – 5.74) | 0.842 |  |  |  |  |
|  | **Sweetener** |  |  |  |  | 4.20 (-2.04 – 10.44) | 0.187 |  |  |  |  |
|  | **Binary** |  |  |  |  |  |  | -0.84 (-2.38 – 0.70) | 0.283 |  |  |

**Supplementary Table 85. Multivariable linear regression analysis for the association of moderate/high coffee consumption and systolic blood pressure (only female).** Not daily coffee consumption (<1 cups/d) served as the reference group. Adjustment was performed for: A) unadjusted; B) main cardiovascular risk factors; C) additional adjustment for additives (separately), D) additional adjustment for additives (binary), E) same adjustment as for group A, but only for subjects who consumed coffee without any additives. Abbreviations as in Table 1.

|  | | A)DBP | | B)DBP | | C)DBP | | D)DBP | | E)DBP | |
| --- | --- | --- | --- | --- | --- | --- | --- | --- | --- | --- | --- |
|  | | *Estimates* | *p* | *Estimates* | *p* | *Estimates* | *p* | *Estimates* | *p* | *Estimates* | *p* |
| Coffee consumption 3-4 cups/day | | -1.51 (-2.29 – -0.72) | **<0.001** | -0.94 (-1.75 – -0.12) | **0.024** | -1.20 (-2.06 – -0.34) | **0.006** | -1.22 (-2.09 – -0.36) | **0.005** | -0.60 (-2.25 – 1.06) | 0.480 |
| Coffee consumption >4 cups/day | | -1.23 (-2.48 – 0.03) | 0.055 | -0.84 (-2.15 – 0.47) | 0.209 | -1.53 (-2.92 – -0.13) | **0.032** | -1.56 (-2.96 – -0.16) | **0.029** | -3.90 (-6.40 – -1.40) | **0.002** |
| Age | |  |  | 0.13 (0.09 – 0.18) | **<0.001** | 0.11 (0.07 – 0.16) | **<0.001** | 0.11 (0.07 – 0.16) | **<0.001** | 0.06 (-0.03 – 0.16) | 0.182 |
| Diabetes mellitus | |  |  | 0.03 (-1.48 – 1.54) | 0.970 | -0.03 (-1.62 – 1.56) | 0.971 | 0.08 (-1.52 – 1.69) | 0.920 | -2.49 (-5.61 – 0.64) | 0.119 |
| Current smoking | |  |  | -1.46 (-2.36 – -0.56) | **0.002** | -1.42 (-2.39 – -0.45) | **0.004** | -1.44 (-2.41 – -0.46) | **0.004** | -2.86 (-4.77 – -0.95) | **0.003** |
| BMI | |  |  | 0.48 (0.41 – 0.55) | **<0.001** | 0.46 (0.39 – 0.54) | **<0.001** | 0.46 (0.38 – 0.53) | **<0.001** | 0.35 (0.20 – 0.50) | **<0.001** |
| Additives | **Milk** |  |  |  |  | -0.24 (-1.09 – 0.61) | 0.577 |  |  |  |  |
|  | **Milk + Sugar** |  |  |  |  | -0.19 (-1.72 – 1.34) | 0.806 |  |  |  |  |
|  | **Milk + Sweetener** |  |  |  |  | -1.48 (-3.58 – 0.62) | 0.167 |  |  |  |  |
|  | **Sugar** |  |  |  |  | -1.21 (-4.64 – 2.22) | 0.491 |  |  |  |  |
|  | **Sweetener** |  |  |  |  | 1.41 (-1.94 – 4.76) | 0.408 |  |  |  |  |
|  | **Binary** |  |  |  |  |  |  | -0.28 (-1.11 – 0.54) | 0.500 |  |  |

**Supplementary Table 86. Multivariable linear regression analysis for the association of moderate/high coffee consumption and diastolic blood pressure (only female).** Not daily coffee consumption (<1 cups/d) served as the reference group. Adjustment was performed for: A) unadjusted; B) main cardiovascular risk factors; C) additional adjustment for additives (separately), D) additional adjustment for additives (binary), E) same adjustment as for group A, but only for subjects who consumed coffee without any additives. Abbreviations as in Table 1.

|  | | A)Heart rate | | B)Heart rate | | C)Heart rate | | D)Heart rate | | E)Heart rate | |
| --- | --- | --- | --- | --- | --- | --- | --- | --- | --- | --- | --- |
|  | | *Estimates* | *p* | *Estimates* | *p* | *Estimates* | *p* | *Estimates* | *p* | *Estimates* | *p* |
| Coffee consumption 3-4 cups/day | | -0.89 (-1.61 – -0.17) | **0.015** | -0.63 (-1.41 – 0.15) | 0.112 | -0.75 (-1.57 – 0.08) | 0.076 | -0.82 (-1.65 – 0.00) | 0.051 | -0.09 (-1.69 – 1.51) | 0.913 |
| Coffee consumption >4 cups/day | | -0.12 (-1.26 – 1.03) | 0.841 | -0.37 (-1.63 – 0.88) | 0.562 | -0.19 (-1.53 – 1.14) | 0.777 | -0.32 (-1.66 – 1.03) | 0.645 | -0.16 (-2.58 – 2.26) | 0.898 |
| Age | |  |  | 0.07 (0.02 – 0.11) | **0.002** | 0.08 (0.03 – 0.12) | **0.001** | 0.08 (0.03 – 0.12) | **0.001** | 0.12 (0.02 – 0.21) | **0.015** |
| Diabetes mellitus | |  |  | 2.69 (1.25 – 4.12) | **<0.001** | 2.41 (0.89 – 3.92) | **0.002** | 2.46 (0.94 – 3.99) | **0.002** | 3.72 (0.74 – 6.70) | **0.014** |
| Arterial hypertension | |  |  | 0.58 (-0.15 – 1.32) | 0.121 | 0.50 (-0.28 – 1.28) | 0.211 | 0.44 (-0.35 – 1.23) | 0.273 | 0.61 (-0.94 – 2.15) | 0.443 |
| Current smoking | |  |  | 0.69 (-0.17 – 1.55) | 0.114 | 1.07 (0.14 – 2.00) | **0.024** | 1.15 (0.21 – 2.08) | **0.016** | 2.12 (0.28 – 3.96) | **0.024** |
| BMI | |  |  | 0.19 (0.12 – 0.26) | **<0.001** | 0.21 (0.13 – 0.28) | **<0.001** | 0.21 (0.13 – 0.28) | **<0.001** | 0.13 (-0.02 – 0.28) | 0.082 |
| Additives | **Milk** |  |  |  |  | -0.43 (-1.24 – 0.39) | 0.303 |  |  |  |  |
|  | **Milk + Sugar** |  |  |  |  | 0.44 (-1.03 – 1.90) | 0.560 |  |  |  |  |
|  | **Milk + Sweetener** |  |  |  |  | 0.32 (-1.69 – 2.32) | 0.756 |  |  |  |  |
|  | **Sugar** |  |  |  |  | 0.29 (-3.00 – 3.57) | 0.865 |  |  |  |  |
|  | **Sweetener** |  |  |  |  | 0.27 (-2.93 – 3.48) | 0.868 |  |  |  |  |
|  | **Binary** |  |  |  |  |  |  | -0.30 (-1.09 – 0.49) | 0.458 |  |  |

**Supplementary Table 867 Multivariable linear regression analysis for the association of mild/moderate/high coffee consumption and heart rate (only female).** Not daily coffee consumption (<1 cups/d) served as the reference group. Adjustment was performed for: A) unadjusted; B) main cardiovascular risk factors; C) additional adjustment for additives (separately), D) additional adjustment for additives (binary), E) same adjustment as for group A, but only for subjects who consumed coffee without any additives. Abbreviations as in Table 1

|  | | A)PQ interval | | B)PQ interval | | C)PQ interval | | D)PQ interva | | E)PQ interval | |
| --- | --- | --- | --- | --- | --- | --- | --- | --- | --- | --- | --- |
|  | | *Estimates* | *p* | *Estimates* | *p* | *Estimates* | *p* | *Estimates* | *p* | *Estimates* | *p* |
| Coffee consumption 3-4 cups/day | | -1.39 (-3.13 – 0.35) | 0.117 | 0.11 (-1.75 – 1.97) | 0.905 | 0.87 (-1.10 – 2.83) | 0.388 | 0.88 (-1.09 – 2.86) | 0.380 | 1.21 (-2.61 – 5.03) | 0.533 |
| Coffee consumption >4 cups/day | | -3.07 (-5.87 – -0.28) | **0.031** | -0.55 (-3.56 – 2.46) | 0.718 | 0.03 (-3.16 – 3.23) | 0.984 | 0.11 (-3.10 – 3.33) | 0.946 | -4.13 (-9.98 – 1.73) | 0.167 |
| Age | |  |  | 0.47 (0.37 – 0.57) | **<0.001** | 0.51 (0.40 – 0.62) | **<0.001** | 0.50 (0.39 – 0.61) | **<0.001** | 0.59 (0.36 – 0.82) | **<0.001** |
| Diabetes mellitus | |  |  | -1.46 (-4.89 – 1.97) | 0.403 | -0.26 (-3.91 – 3.38) | 0.887 | -0.38 (-4.06 – 3.30) | 0.839 | 0.82 (-6.15 – 7.79) | 0.817 |
| Arterial hypertension | |  |  | 1.61 (-0.14 – 3.36) | 0.071 | 1.53 (-0.34 – 3.40) | 0.108 | 1.50 (-0.38 – 3.39) | 0.117 | 0.71 (-2.98 – 4.41) | 0.705 |
| Current smoking | |  |  | -2.65 (-4.70 – -0.59) | **0.012** | -2.21 (-4.44 – 0.02) | 0.052 | -2.30 (-4.54 – -0.07) | **0.043** | -1.16 (-5.64 – 3.32) | 0.611 |
| BMI | |  |  | 0.35 (0.18 – 0.51) | **<0.001** | 0.35 (0.17 – 0.53) | **<0.001** | 0.34 (0.16 – 0.52) | **<0.001** | 0.39 (0.04 – 0.74) | **0.031** |
| Additives | **Milk** |  |  |  |  | 2.19 (0.25 – 4.14) | **0.027** |  |  |  |  |
|  | **Milk + Sugar** |  |  |  |  | 2.09 (-1.43 – 5.62) | 0.244 |  |  |  |  |
|  | **Milk + Sweetener** |  |  |  |  | -3.45 (-8.24 – 1.34) | 0.158 |  |  |  |  |
|  | **Sugar** |  |  |  |  | 5.08 (-2.69 – 12.84) | 0.200 |  |  |  |  |
|  | **Sweetener** |  |  |  |  | -2.80 (-10.66 – 5.06) | 0.485 |  |  |  |  |
|  | **Binary** |  |  |  |  |  |  | 1.93 (0.03 – 3.84) | **0.046** |  |  |

**Supplementary Table 88. Multivariable linear regression analysis for the association of mild/moderate/high coffee consumption and PQ interval (only female).** Not daily coffee consumption (<1 cups/d) served as the reference group. Adjustment was performed for: A) unadjusted; B) main cardiovascular risk factors; C) additional adjustment for additives (separately), D) additional adjustment for additives (binary), E) same adjustment as for group A, but only for subjects who consumed coffee without any additives. Abbreviations as in Table 1.

|  | | A)QRS interval | | B)QRS interval | | C)QRS interval | | D)QRS interval | | E)QRS interval | |
| --- | --- | --- | --- | --- | --- | --- | --- | --- | --- | --- | --- |
|  | | *Estimates* | *p* | *Estimates* | *p* | *Estimates* | *p* | *Estimates* | *p* | *Estimates* | *p* |
| Coffee consumption 3-4 cups/day | | 0.38 (-0.43 – 1.18) | 0.359 | 0.77 (-0.09 – 1.64) | 0.080 | 0.78 (-0.13 – 1.69) | 0.094 | 0.79 (-0.13 – 1.70) | 0.092 | 0.24 (-1.52 – 2.00) | 0.788 |
| Coffee consumption >4 cups/day | | -1.25 (-2.55 – 0.06) | 0.061 | -0.94 (-2.35 – 0.47) | 0.192 | -0.66 (-2.15 – 0.84) | 0.389 | -0.58 (-2.07 – 0.92) | 0.452 | -0.30 (-3.02 – 2.41) | 0.827 |
| Age | |  |  | 0.07 (0.02 – 0.12) | **0.003** | 0.07 (0.02 – 0.12) | **0.007** | 0.07 (0.02 – 0.12) | **0.008** | 0.12 (0.02 – 0.23) | **0.023** |
| Diabetes mellitus | |  |  | -0.64 (-2.24 – 0.95) | 0.429 | -0.70 (-2.38 – 0.99) | 0.418 | -0.69 (-2.39 – 1.00) | 0.422 | -2.11 (-5.32 – 1.10) | 0.197 |
| Arterial hypertension | |  |  | 0.93 (0.11 – 1.75) | **0.026** | 0.63 (-0.24 – 1.50) | 0.154 | 0.62 (-0.26 – 1.49) | 0.167 | 0.90 (-0.81 – 2.61) | 0.302 |
| Current smoking | |  |  | -0.98 (-1.94 – -0.02) | **0.046** | -1.23 (-2.27 – -0.19) | **0.020** | -1.34 (-2.38 – -0.30) | **0.012** | -2.67 (-4.74 – -0.59) | **0.012** |
| BMI | |  |  | 0.15 (0.07 – 0.22) | **<0.001** | 0.14 (0.06 – 0.22) | **0.001** | 0.14 (0.05 – 0.22) | **0.001** | 0.13 (-0.03 – 0.29) | 0.118 |
| Additives | **Milk** |  |  |  |  | 0.30 (-0.60 – 1.21) | 0.511 |  |  |  |  |
|  | **Milk + Sugar** |  |  |  |  | 0.92 (-0.72 – 2.56) | 0.273 |  |  |  |  |
|  | **Milk + Sweetener** |  |  |  |  | 0.43 (-1.81 – 2.67) | 0.706 |  |  |  |  |
|  | **Sugar** |  |  |  |  | -0.97 (-4.60 – 2.66) | 0.601 |  |  |  |  |
|  | **Sweetener** |  |  |  |  | -1.25 (-4.83 – 2.34) | 0.495 |  |  |  |  |
|  | **Binary** |  |  |  |  |  |  | 0.36 (-0.52 – 1.24) | 0.424 |  |  |

**Supplementary Table 89. Multivariable linear regression analysis for the association of mild/moderate/high coffee consumption and QRS interval (only female).** Not daily coffee consumption (<1 cups/d) served as the reference group. Adjustment was performed for: A) unadjusted; B) main cardiovascular risk factors; C) additional adjustment for additives (separately), D) additional adjustment for additives (binary), E) same adjustment as for group A, but only for subjects who consumed coffee without any additives. Abbreviations as in Table 1.

|  | | A)QTc interval | | B)QTc interval | | C)QTc interval | | D)QTc interval | | E)QTc interval | |
| --- | --- | --- | --- | --- | --- | --- | --- | --- | --- | --- | --- |
|  | | *Estimates* | *p* | *Estimates* | *p* | *Estimates* | *p* | *Estimates* | *p* | *Estimates* | *p* |
| Coffee consumption 3-4 cups/day | | -3.23 (-4.84 – -1.61) | **<0.001** | -2.35 (-4.06 – -0.64) | **0.007** | -2.73 (-4.53 – -0.92) | **0.003** | -2.82 (-4.63 – -1.01) | **0.002** | -2.97 (-6.26 – 0.32) | 0.077 |
| Coffee consumption >4 cups/day | | -1.53 (-4.17 – 1.11) | 0.256 | -1.09 (-3.90 – 1.71) | 0.444 | -0.69 (-3.67 – 2.28) | 0.647 | -0.69 (-3.68 – 2.30) | 0.650 | -0.01 (-5.06 – 5.05) | 0.998 |
| Age | |  |  | 0.33 (0.23 – 0.42) | **<0.001** | 0.33 (0.23 – 0.43) | **<0.001** | 0.32 (0.22 – 0.42) | **<0.001** | 0.31 (0.12 – 0.51) | **0.002** |
| Diabetes mellitus | |  |  | 3.55 (0.42 – 6.67) | **0.026** | 2.95 (-0.35 – 6.26) | 0.080 | 3.13 (-0.20 – 6.46) | 0.066 | 5.07 (-0.92 – 11.07) | 0.097 |
| Arterial hypertension | |  |  | 2.36 (0.74 – 3.98) | **0.004** | 1.83 (0.11 – 3.55) | **0.037** | 1.85 (0.12 – 3.58) | **0.036** | 1.57 (-1.62 – 4.75) | 0.335 |
| Current smoking | |  |  | 0.77 (-1.12 – 2.67) | 0.425 | 0.51 (-1.54 – 2.57) | 0.623 | 0.65 (-1.41 – 2.71) | 0.535 | -0.51 (-4.36 – 3.35) | 0.796 |
| BMI | |  |  | 0.46 (0.31 – 0.62) | **<0.001** | 0.45 (0.29 – 0.61) | **<0.001** | 0.46 (0.30 – 0.63) | **<0.001** | 0.34 (0.03 – 0.65) | **0.030** |
| Additives | **Milk** |  |  |  |  | -0.56 (-2.35 – 1.23) | 0.542 |  |  |  |  |
|  | **Milk + Sugar** |  |  |  |  | 1.56 (-1.67 – 4.79) | 0.343 |  |  |  |  |
|  | **Milk + Sweetener** |  |  |  |  | -0.02 (-4.49 – 4.44) | 0.992 |  |  |  |  |
|  | **Sugar** |  |  |  |  | 1.49 (-5.57 – 8.56) | 0.679 |  |  |  |  |
|  | **Sweetener** |  |  |  |  | -3.29 (-10.65 – 4.07) | 0.380 |  |  |  |  |
|  | **Binary** |  |  |  |  |  |  | -0.27 (-2.02 – 1.48) | 0.764 |  |  |

**Supplementary Table 90. Multivariable linear regression analysis for the association of mild/moderate/high coffee consumption and QTc interval (only female).** Not daily coffee consumption (<1 cups/d) served as the reference group. Adjustment was performed for: A) unadjusted; B) main cardiovascular risk factors; C) additional adjustment for additives (separately), D) additional adjustment for additives (binary), E) same adjustment as for group A, but only for subjects who consumed coffee without any additives. Abbreviations as in Table 1

|  | | A)LVEF | | B)LVEF | | C)LVEF | | D)LVEF | | E)LVEF | |
| --- | --- | --- | --- | --- | --- | --- | --- | --- | --- | --- | --- |
|  | | *Estimates* | *p* | *Estimates* | *p* | *Estimates* | *p* | *Estimates* | *p* | *Estimates* | *p* |
| Coffee consumption 3-4 cups/day | | 0.43 (0.05 – 0.82) | **0.028** | 0.60 (0.18 – 1.03) | **0.005** | 0.59 (0.14 – 1.04) | **0.010** | 0.60 (0.15 – 1.05) | **0.009** | 1.69 (0.84 – 2.54) | **<0.001** |
| Coffee consumption >4 cups/day | | 0.12 (-0.50 – 0.73) | 0.707 | 0.34 (-0.34 – 1.03) | 0.330 | 0.30 (-0.44 – 1.03) | 0.427 | 0.29 (-0.45 – 1.02) | 0.449 | 0.59 (-0.73 – 1.90) | 0.382 |
| Age | |  |  | 0.01 (-0.01 – 0.03) | 0.330 | 0.01 (-0.02 – 0.03) | 0.497 | 0.01 (-0.02 – 0.03) | 0.460 | 0.01 (-0.04 – 0.06) | 0.721 |
| Diabetes mellitus | |  |  | -0.47 (-1.26 – 0.32) | 0.242 | -0.37 (-1.21 – 0.47) | 0.386 | -0.38 (-1.23 – 0.46) | 0.373 | -0.89 (-2.46 – 0.68) | 0.265 |
| Arterial hypertension | |  |  | -0.61 (-1.01 – -0.21) | **0.003** | -0.61 (-1.03 – -0.18) | **0.005** | -0.58 (-1.01 – -0.15) | **0.008** | -1.10 (-1.92 – -0.28) | **0.009** |
| Current smoking | |  |  | -0.07 (-0.53 – 0.40) | 0.784 | -0.06 (-0.57 – 0.44) | 0.808 | -0.05 (-0.56 – 0.45) | 0.835 | -0.26 (-1.26 – 0.74) | 0.610 |
| BMI | |  |  | -0.10 (-0.14 – -0.06) | **<0.001** | -0.09 (-0.13 – -0.05) | **<0.001** | -0.10 (-0.14 – -0.06) | **<0.001** | -0.06 (-0.14 – 0.02) | 0.122 |
| Additives | **Milk** |  |  |  |  | 0.12 (-0.33 – 0.56) | 0.609 |  |  |  |  |
|  | **Milk + Sugar** |  |  |  |  | 0.01 (-0.80 – 0.82) | 0.982 |  |  |  |  |
|  | **Milk + Sweetener** |  |  |  |  | -0.41 (-1.49 – 0.68) | 0.461 |  |  |  |  |
|  | **Sugar** |  |  |  |  | 0.23 (-1.56 – 2.01) | 0.803 |  |  |  |  |
|  | **Sweetener** |  |  |  |  | 0.34 (-1.41 – 2.10) | 0.703 |  |  |  |  |
|  | **Binary** |  |  |  |  |  |  | 0.08 (-0.36 – 0.52) | 0.716 |  |  |

**Supplementary Table 91. Multivariable linear regression analysis for the association of mild/moderate/high coffee consumption and LVEF (only female).** Not daily coffee consumption (<1 cups/d) served as the reference group. Adjustment was performed for: A) unadjusted; B) main cardiovascular risk factors; C) additional adjustment for additives (separately), D) additional adjustment for additives (binary), E) same adjustment as for group A, but only for subjects who consumed coffee without any additives. Abbreviations as in Table 1.

|  | | A)LV mass index | | B)LV mass index | | C)LV mass index | | D)LV mass index | | E)LV mass index | |
| --- | --- | --- | --- | --- | --- | --- | --- | --- | --- | --- | --- |
|  | | *Estimates* | *p* | *Estimates* | *p* | *Estimates* | *p* | *Estimates* | *p* | *Estimates* | *p* |
| Coffee consumption 3-4 cups/day | | 0.58 (-0.80 – 1.97) | 0.408 | 1.33 (-0.08 – 2.74) | 0.065 | 1.06 (-0.44 – 2.55) | 0.166 | 1.07 (-0.42 – 2.56) | 0.159 | 0.13 (-2.93 – 3.18) | 0.936 |
| Coffee consumption >4 cups/day | | 0.99 (-1.18 – 3.16) | 0.373 | 1.20 (-1.05 – 3.46) | 0.294 | 1.28 (-1.13 – 3.68) | 0.299 | 1.43 (-0.99 – 3.85) | 0.247 | -1.15 (-5.80 – 3.49) | 0.626 |
| Age | |  |  | 0.22 (0.14 – 0.30) | **<0.001** | 0.21 (0.13 – 0.29) | **<0.001** | 0.21 (0.12 – 0.29) | **<0.001** | 0.28 (0.10 – 0.46) | **0.003** |
| Diabetes mellitus | |  |  | -2.04 (-4.72 – 0.64) | 0.136 | -2.34 (-5.19 – 0.52) | 0.109 | -2.05 (-4.92 – 0.81) | 0.160 | -2.28 (-8.11 – 3.56) | 0.444 |
| Arterial hypertension | |  |  | 5.18 (3.87 – 6.50) | **<0.001** | 4.92 (3.51 – 6.32) | **<0.001** | 4.93 (3.52 – 6.33) | **<0.001** | 5.62 (2.64 – 8.60) | **<0.001** |
| Current smoking | |  |  | 1.23 (-0.35 – 2.80) | 0.127 | 1.00 (-0.70 – 2.70) | 0.250 | 0.81 (-0.89 – 2.51) | 0.353 | 2.26 (-1.39 – 5.91) | 0.224 |
| BMI | |  |  | 0.58 (0.45 – 0.70) | **<0.001** | 0.58 (0.44 – 0.72) | **<0.001** | 0.59 (0.45 – 0.72) | **<0.001** | 0.64 (0.35 – 0.92) | **<0.001** |
| Additives | **Milk** |  |  |  |  | -0.63 (-2.11 – 0.86) | 0.409 |  |  |  |  |
|  | **Milk + Sugar** |  |  |  |  | -1.49 (-4.17 – 1.19) | 0.276 |  |  |  |  |
|  | **Milk + Sweetener** |  |  |  |  | -1.82 (-5.57 – 1.93) | 0.342 |  |  |  |  |
|  | **Sugar** |  |  |  |  | 0.51 (-5.24 – 6.27) | 0.861 |  |  |  |  |
|  | **Sweetener** |  |  |  |  | -2.94 (-8.37 – 2.48) | 0.287 |  |  |  |  |
|  | **Binary** |  |  |  |  |  |  | -0.75 (-2.20 – 0.70) | 0.308 |  |  |

**Supplementary Table 91. Multivariable linear regression analysis for the association of mild/moderate/high coffee consumption and LV mass index (only female).** Not daily coffee consumption (<1 cups/d) served as the reference group. Adjustment was performed for: A) unadjusted; B) main cardiovascular risk factors; C) additional adjustment for additives (separately), D) additional adjustment for additives (binary), E) same adjustment as for group A, but only for subjects who consumed coffee without any additives. Abbreviations as in Table 1.

|  | | A)E/e‘ ratio | | B)E/e‘ ratio | | C)E/e‘ ratio | | D)E/e‘ ratio | | E)E/e‘ ratio | |
| --- | --- | --- | --- | --- | --- | --- | --- | --- | --- | --- | --- |
|  | | *Estimates* | *p* | *Estimates* | *p* | *Estimates* | *p* | *Estimates* | *p* | *Estimates* | *p* |
| Coffee consumption 3-4 cups/day | | -0.31 (-0.48 – -0.15) | **<0.001** | -0.05 (-0.22 – 0.11) | 0.522 | -0.07 (-0.25 – 0.10) | 0.415 | -0.08 (-0.25 – 0.10) | 0.390 | 0.03 (-0.33 – 0.39) | 0.876 |
| Coffee consumption >4 cups/day | | -0.15 (-0.41 – 0.11) | 0.249 | 0.07 (-0.20 – 0.34) | 0.607 | 0.03 (-0.25 – 0.32) | 0.830 | 0.04 (-0.25 – 0.33) | 0.783 | 0.23 (-0.33 – 0.78) | 0.422 |
| Age | |  |  | 0.07 (0.06 – 0.08) | **<0.001** | 0.07 (0.06 – 0.08) | **<0.001** | 0.07 (0.06 – 0.08) | **<0.001** | 0.08 (0.06 – 0.10) | **<0.001** |
| Diabetes mellitus | |  |  | 0.50 (0.19 – 0.80) | **0.001** | 0.50 (0.18 – 0.82) | **0.002** | 0.50 (0.18 – 0.82) | **0.003** | 0.76 (0.11 – 1.41) | **0.022** |
| Arterial hypertension | |  |  | 0.55 (0.39 – 0.70) | **<0.001** | 0.57 (0.41 – 0.74) | **<0.001** | 0.57 (0.41 – 0.74) | **<0.001** | 0.57 (0.22 – 0.92) | **0.001** |
| Current smoking | |  |  | 0.32 (0.14 – 0.50) | **0.001** | 0.34 (0.15 – 0.54) | **0.001** | 0.35 (0.15 – 0.54) | **<0.001** | 0.57 (0.15 – 0.99) | **0.008** |
| BMI | |  |  | 0.02 (0.01 – 0.04) | **0.001** | 0.02 (0.01 – 0.04) | **0.009** | 0.02 (0.01 – 0.04) | **0.006** | 0.03 (-0.00 – 0.06) | 0.068 |
| Additives | **Milk** |  |  |  |  | -0.04 (-0.21 – 0.13) | 0.644 |  |  |  |  |
|  | **Milk + Sugar** |  |  |  |  | -0.16 (-0.47 – 0.16) | 0.328 |  |  |  |  |
|  | **Milk + Sweetener** |  |  |  |  | 0.21 (-0.22 – 0.65) | 0.336 |  |  |  |  |
|  | **Sugar** |  |  |  |  | -0.24 (-0.91 – 0.43) | 0.485 |  |  |  |  |
|  | **Sweetener** |  |  |  |  | 0.76 (0.09 – 1.43) | **0.027** |  |  |  |  |
|  | **Binary** |  |  |  |  |  |  | -0.03 (-0.20 – 0.14) | 0.713 |  |  |

**Supplementary Table 93. Multivariable linear regression analysis for the association of mild/moderate/high coffee consumption and E/e’ ratio (only female).** Not daily coffee consumption (<1 cups/d) served as the reference group. Adjustment was performed for: A) unadjusted; B) main cardiovascular risk factors; C) additional adjustment for additives (separately), D) additional adjustment for additives (binary), E) same adjustment as for group A, but only for subjects who consumed coffee without any additives. Abbreviations as in Table 1.

|  | | A)TR Vmax in m/s | | B)TR Vmax in m/s | | C)TR Vmax in m/s | | D)TR Vmax in m/s | | E)TR Vmax in m/s | |
| --- | --- | --- | --- | --- | --- | --- | --- | --- | --- | --- | --- |
|  | | *Estimates* | *p* | *Estimates* | *p* | *Estimates* | *p* | *Estimates* | *p* | *Estimates* | *p* |
| Coffee consumption 3-4 cups/day | | -0.03 (-0.06 – 0.01) | 0.110 | -0.01 (-0.04 – 0.02) | 0.564 | -0.01 (-0.04 – 0.03) | 0.686 | -0.00 (-0.04 – 0.03) | 0.778 | -0.06 (-0.12 – 0.01) | 0.086 |
| Coffee consumption >4 cups/day | | -0.04 (-0.09 – 0.01) | 0.146 | -0.00 (-0.06 – 0.05) | 0.893 | -0.03 (-0.09 – 0.03) | 0.387 | -0.02 (-0.09 – 0.04) | 0.442 | -0.05 (-0.16 – 0.06) | 0.363 |
| Age | |  |  | 0.00 (0.00 – 0.01) | **<0.001** | 0.00 (0.00 – 0.01) | **<0.001** | 0.00 (0.00 – 0.01) | **<0.001** | 0.00 (-0.00 – 0.01) | 0.259 |
| Diabetes mellitus | |  |  | 0.07 (0.01 – 0.13) | **0.029** | 0.07 (0.00 – 0.13) | **0.049** | 0.07 (0.00 – 0.14) | **0.039** | 0.00 (-0.12 – 0.12) | 0.962 |
| Arterial hypertension | |  |  | 0.07 (0.04 – 0.10) | **<0.001** | 0.07 (0.04 – 0.11) | **<0.001** | 0.07 (0.03 – 0.10) | **<0.001** | 0.11 (0.05 – 0.18) | **0.001** |
| Current smoking | |  |  | 0.01 (-0.03 – 0.05) | 0.521 | 0.01 (-0.03 – 0.05) | 0.591 | 0.01 (-0.03 – 0.05) | 0.655 | -0.02 (-0.10 – 0.06) | 0.601 |
| BMI | |  |  | 0.00 (0.00 – 0.01) | **0.012** | 0.00 (0.00 – 0.01) | **0.009** | 0.00 (0.00 – 0.01) | **0.005** | -0.00 (-0.01 – 0.01) | 0.746 |
| Additives | **Milk** |  |  |  |  | -0.04 (-0.07 – -0.00) | **0.040** |  |  |  |  |
|  | **Milk + Sugar** |  |  |  |  | -0.05 (-0.11 – 0.02) | 0.154 |  |  |  |  |
|  | **Milk + Sweetener** |  |  |  |  | 0.01 (-0.08 – 0.09) | 0.841 |  |  |  |  |
|  | **Sugar** |  |  |  |  | -0.14 (-0.26 – -0.01) | **0.031** |  |  |  |  |
|  | **Sweetener** |  |  |  |  | 0.13 (0.00 – 0.25) | **0.043** |  |  |  |  |
|  | **Binary** |  |  |  |  |  |  | -0.04 (-0.07 – -0.00) | **0.032** |  |  |

**Supplementary Table 94. Multivariable linear regression analysis for the association of mild/moderate/high coffee consumption and TR Vmax in m/s (only female).** Not daily coffee consumption (<1 cups/d) served as the reference group. Adjustment was performed for: A) unadjusted; B) main cardiovascular risk factors; C) additional adjustment for additives (separately), D) additional adjustment for additives (binary), E) same adjustment as for group A, but only for subjects who consumed coffee without any additives. Abbreviations as in Table 1.

|  | | A)TAPSE in mm | | B)TAPSE in mm | | C)TAPSE in mm | | D)TAPSE in mm | | E)TAPSE in mm | |
| --- | --- | --- | --- | --- | --- | --- | --- | --- | --- | --- | --- |
|  | | *Estimates* | *p* | *Estimates* | *p* | *Estimates* | *p* | *Estimates* | *p* | *Estimates* | *p* |
| Coffee consumption 3-4 cups/day | | 0.24 (-0.13 – 0.60) | 0.200 | 0.01 (-0.39 – 0.41) | 0.946 | -0.02 (-0.45 – 0.40) | 0.915 | -0.02 (-0.44 – 0.41) | 0.942 | 0.26 (-0.58 – 1.10) | 0.544 |
| Coffee consumption >4 cups/day | | 0.27 (-0.30 – 0.84) | 0.361 | 0.20 (-0.43 – 0.84) | 0.529 | 0.21 (-0.46 – 0.89) | 0.535 | 0.24 (-0.44 – 0.92) | 0.485 | -0.04 (-1.26 – 1.18) | 0.948 |
| Age | |  |  | -0.08 (-0.10 – -0.06) | **<0.001** | -0.09 (-0.11 – -0.07) | **<0.001** | -0.09 (-0.11 – -0.07) | **<0.001** | -0.10 (-0.15 – -0.05) | **<0.001** |
| Diabetes mellitus | |  |  | -0.71 (-1.50 – 0.09) | 0.081 | -0.64 (-1.48 – 0.20) | 0.133 | -0.61 (-1.45 – 0.22) | 0.151 | -0.92 (-2.53 – 0.70) | 0.264 |
| Arterial hypertension | |  |  | -0.14 (-0.51 – 0.23) | 0.464 | -0.10 (-0.50 – 0.30) | 0.616 | -0.12 (-0.52 – 0.28) | 0.546 | -0.74 (-1.53 – 0.06) | 0.070 |
| Current smoking | |  |  | -0.26 (-0.69 – 0.18) | 0.251 | -0.30 (-0.77 – 0.18) | 0.222 | -0.31 (-0.79 – 0.16) | 0.195 | -0.29 (-1.26 – 0.67) | 0.552 |
| BMI | |  |  | 0.01 (-0.03 – 0.04) | 0.729 | 0.01 (-0.03 – 0.04) | 0.791 | 0.01 (-0.03 – 0.05) | 0.754 | 0.02 (-0.06 – 0.10) | 0.687 |
| Additives | **Milk** |  |  |  |  | -0.13 (-0.56 – 0.29) | 0.541 |  |  |  |  |
|  | **Milk + Sugar** |  |  |  |  | 0.08 (-0.70 – 0.86) | 0.835 |  |  |  |  |
|  | **Milk + Sweetener** |  |  |  |  | 0.16 (-0.88 – 1.20) | 0.763 |  |  |  |  |
|  | **Sugar** |  |  |  |  | -1.71 (-3.34 – -0.09) | **0.039** |  |  |  |  |
|  | **Sweetener** |  |  |  |  | -0.57 (-2.19 – 1.06) | 0.494 |  |  |  |  |
|  | **Binary** |  |  |  |  |  |  | -0.13 (-0.54 – 0.29) | 0.549 |  |  |

**Supplementary Table 95. Multivariable linear regression analysis for the association of mild/moderate/high coffee consumption and TAPSE in mm (only female).** Not daily coffee consumption (<1 cups/d) served as the reference group. Adjustment was performed for: A) unadjusted; B) main cardiovascular risk factors; C) additional adjustment for additives (separately), D) additional adjustment for additives (binary), E) same adjustment as for group A, but only for subjects who consumed coffee without any additives. Abbreviations as in Table 1.

|  | | A)LASV in ml | | B)LASV in ml | | C)LASV in ml | | D)LASV in ml | | E)LASV in ml | |
| --- | --- | --- | --- | --- | --- | --- | --- | --- | --- | --- | --- |
|  | | *Estimates* | *p* | *Estimates* | *p* | *Estimates* | *p* | *Estimates* | *p* | *Estimates* | *p* |
| Coffee consumption 3-4 cups/day | | 0.56 (-0.13 – 1.26) | 0.111 | 0.71 (-0.02 – 1.44) | 0.058 | 0.75 (-0.03 – 1.52) | 0.058 | 0.79 (0.02 – 1.56) | **0.045** | 1.13 (-0.38 – 2.64) | 0.141 |
| Coffee consumption >4 cups/day | | 0.63 (-0.47 – 1.72) | 0.260 | 0.80 (-0.37 – 1.98) | 0.180 | 0.97 (-0.28 – 2.22) | 0.127 | 1.07 (-0.19 – 2.32) | 0.096 | 1.60 (-0.70 – 3.90) | 0.171 |
| Age | |  |  | 0.04 (-0.00 – 0.08) | 0.065 | 0.02 (-0.02 – 0.06) | 0.376 | 0.02 (-0.02 – 0.06) | 0.330 | 0.06 (-0.03 – 0.15) | 0.161 |
| Diabetes mellitus | |  |  | -1.31 (-2.69 – 0.07) | 0.063 | -0.95 (-2.42 – 0.52) | 0.204 | -0.91 (-2.39 – 0.57) | 0.226 | -1.64 (-4.45 – 1.18) | 0.254 |
| Arterial hypertension | |  |  | 1.37 (0.69 – 2.05) | **<0.001** | 1.45 (0.73 – 2.17) | **<0.001** | 1.53 (0.80 – 2.25) | **<0.001** | 0.87 (-0.55 – 2.30) | 0.228 |
| Current smoking | |  |  | -0.79 (-1.59 – 0.01) | 0.054 | -1.00 (-1.86 – -0.14) | **0.023** | -1.01 (-1.87 – -0.15) | **0.021** | -1.75 (-3.49 – -0.02) | **0.048** |
| BMI | |  |  | 0.13 (0.06 – 0.20) | **<0.001** | 0.12 (0.05 – 0.19) | **0.001** | 0.12 (0.04 – 0.19) | **0.001** | 0.10 (-0.04 – 0.23) | 0.157 |
| Additives | **Milk** |  |  |  |  | 0.17 (-0.60 – 0.95) | 0.664 |  |  |  |  |
|  | **Milk + Sugar** |  |  |  |  | -0.63 (-2.00 – 0.75) | 0.370 |  |  |  |  |
|  | **Milk + Sweetener** |  |  |  |  | 0.50 (-1.48 – 2.48) | 0.622 |  |  |  |  |
|  | **Sugar** |  |  |  |  | -0.00 (-3.07 – 3.06) | 0.998 |  |  |  |  |
|  | **Sweetener** |  |  |  |  | 1.12 (-1.74 – 3.97) | 0.442 |  |  |  |  |
|  | **Binary** |  |  |  |  |  |  | 0.13 (-0.62 – 0.89) | 0.727 |  |  |

**Supplementary Table 96 Multivariable linear regression analysis for the association of mild/moderate/high coffee consumption and LASV in ml (only female).** Not daily coffee consumption (<1 cups/d) served as the reference group. Adjustment was performed for: A) unadjusted; B) main cardiovascular risk factors; C) additional adjustment for additives (separately), D) additional adjustment for additives (binary), E) same adjustment as for group A, but only for subjects who consumed coffee without any additives. Abbreviations as in Table 1.

|  | | A)LBB | | B)LBB | | C)LBB | | D)LBB | | E)LBB | |
| --- | --- | --- | --- | --- | --- | --- | --- | --- | --- | --- | --- |
|  | | *Odds Ratios* | *p* | *Odds Ratios* | *p* | *Odds Ratios* | *p* | *Odds Ratios* | *p* | *Odds Ratios* | *p* |
| Coffee consumption 3-4 cups/day | | 0.95 (0.58 – 1.50) | 0.830 | 1.09 (0.64 – 1.80) | 0.729 | 1.01 (0.55 – 1.77) | 0.965 | 1.09 (0.62 – 1.83) | 0.756 | 0.52 (0.15 – 1.42) | 0.242 |
| Coffee consumption >4 cups/day | | 0.76 (0.29 – 1.62) | 0.515 | 0.84 (0.29 – 1.97) | 0.715 | 0.60 (0.14 – 1.73) | 0.411 | 0.52 (0.12 – 1.48) | 0.285 | 0.71 (0.11 – 2.76) | 0.664 |
| Age | |  |  | 1.05 (1.02 – 1.08) | **0.001** | 1.04 (1.00 – 1.07) | **0.036** | 1.04 (1.00 – 1.07) | **0.025** | 1.06 (1.00 – 1.13) | 0.058 |
| Diabetes mellitus | |  |  | 1.19 (0.53 – 2.39) | 0.642 | 1.33 (0.53 – 2.91) | 0.504 | 1.31 (0.56 – 2.73) | 0.499 |  |  |
| Arterial hypertension | |  |  | 1.67 (0.98 – 2.94) | 0.068 | 1.72 (0.95 – 3.26) | 0.082 | 1.58 (0.91 – 2.85) | 0.117 | 2.42 (0.85 – 8.73) | 0.128 |
| Current smoking | |  |  | 1.17 (0.63 – 2.04) | 0.609 | 1.24 (0.60 – 2.36) | 0.542 | 1.20 (0.61 – 2.18) | 0.573 | 1.90 (0.59 – 5.24) | 0.241 |
| BMI | |  |  | 1.03 (0.99 – 1.08) | 0.129 | 1.02 (0.96 – 1.07) | 0.537 | 1.03 (0.98 – 1.07) | 0.253 | 1.02 (0.94 – 1.10) | 0.608 |
| Additives | Milk |  |  |  |  | 0.86 (0.52 – 1.46) | 0.555 |  |  |  |  |
|  | Binary |  |  |  |  |  |  | 0.86 (0.53 – 1.45) | 0.569 |  |  |

**Supplementary Table 97. Multivariable logistic regression analysis for the association of mild/moderate/high coffee consumption and LBBB (only female).** Not daily coffee consumption (<1 cups/d) served as the reference group. Adjustment was performed for: A) unadjusted; B) main cardiovascular risk factors; C) additional adjustment for additives (separately), D) additional adjustment for additives (binary), E) same adjustment as for group A, but only for subjects who consumed coffee without any additives. Abbreviations as in Table 1.

|  | | A)AV_block | | B)AV_block | | C)AV block | | D)AV_block | | E)AV block | |
| --- | --- | --- | --- | --- | --- | --- | --- | --- | --- | --- | --- |
|  | | *Odds Ratios* | *p* | *Odds Ratios* | *p* | *Odds Ratios* | *p* | *Odds Ratios* | *p* | *Odds Ratios* | *p* |
| Coffee consumption 3-4 cups/day | | 1.13 (0.76 – 1.64) | 0.545 | 1.32 (0.85 – 2.03) | 0.205 | 1.47 (0.93 – 2.28) | 0.095 | 1.42 (0.90 – 2.20) | 0.123 | 1.00 (0.40 – 2.30) | 0.991 |
| Coffee consumption >4 cups/day | | 0.56 (0.22 – 1.18) | 0.170 | 0.88 (0.34 – 1.93) | 0.776 | 0.86 (0.29 – 2.01) | 0.749 | 0.80 (0.27 – 1.88) | 0.651 | 1.03 (0.23 – 3.37) | 0.969 |
| Age | |  |  | 1.07 (1.04 – 1.10) | **<0.001** | 1.07 (1.04 – 1.10) | **<0.001** | 1.07 (1.04 – 1.10) | **<0.001** | 1.08 (1.03 – 1.15) | **0.006** |
| Diabetes mellitus | |  |  | 0.84 (0.36 – 1.70) | 0.652 | 0.88 (0.36 – 1.87) | 0.760 | 0.97 (0.42 – 1.98) | 0.935 | 0.67 (0.10 – 2.53) | 0.609 |
| Arterial hypertension | |  |  | 0.92 (0.60 – 1.43) | 0.699 | 0.91 (0.58 – 1.47) | 0.704 | 0.89 (0.57 – 1.41) | 0.612 | 0.92 (0.41 – 2.21) | 0.854 |
| Current smoking | |  |  | 0.82 (0.45 – 1.41) | 0.502 | 0.94 (0.50 – 1.66) | 0.849 | 0.89 (0.47 – 1.56) | 0.694 | 1.86 (0.69 – 4.50) | 0.189 |
| BMI | |  |  | 1.03 (0.99 – 1.07) | 0.113 | 1.03 (0.98 – 1.07) | 0.259 | 1.03 (0.99 – 1.07) | 0.168 | 1.03 (0.95 – 1.11) | 0.434 |
| Additives | **Milk** |  |  |  |  | 0.98 (0.63 – 1.55) | 0.915 |  |  |  |  |
|  | **Milk + Sugar** |  |  |  |  | 1.08 (0.43 – 2.39) | 0.852 |  |  |  |  |
|  | **Binary** |  |  |  |  |  |  | 0.95 (0.62 – 1.49) | 0.818 |  |  |

**Supplementary Table 98. Multivariable logistic regression analysis for the association of mild/moderate/high coffee consumption and AV block (only female).** Not daily coffee consumption (<1 cups/d) served as the reference group. Adjustment was performed for: A) unadjusted; B) main cardiovascular risk factors; C) additional adjustment for additives (separately), D) additional adjustment for additives (binary), E) same adjustment as for group A, but only for subjects who consumed coffee without any additives. Abbreviations as in Table 1.

|  | | A)Atrial_fibrillation | | B)Atrial_fibrillation | | C)Atrial_fibrillation | | DAtrial_fibrillation | | E)Atrial_fibrillation | |
| --- | --- | --- | --- | --- | --- | --- | --- | --- | --- | --- | --- |
|  | | *Odds Ratios* | *p* | *Odds Ratios* | *p* | *Odds Ratios* | *p* | *Odds Ratios* | *p* | *Odds Ratios* | *p* |
| Coffee consumption 3-4 cups/day | | 0.81 (0.56 – 1.14) | 0.233 | 1.03 (0.69 – 1.51) | 0.879 | 0.93 (0.60 – 1.42) | 0.752 | 0.97 (0.63 – 1.46) | 0.903 | 0.60 (0.22 – 1.41) | 0.278 |
| Coffee consumption >4 cups/day | | 0.42 (0.18 – 0.84) | **0.027** | 0.71 (0.29 – 1.47) | 0.397 | 0.58 (0.20 – 1.33) | 0.245 | 0.57 (0.20 – 1.31) | 0.233 | 0.61 (0.09 – 2.29) | 0.529 |
| Age | |  |  | 1.11 (1.08 – 1.13) | **<0.001** | 1.09 (1.07 – 1.12) | **<0.001** | 1.10 (1.07 – 1.13) | **<0.001** | 1.09 (1.04 – 1.16) | **0.002** |
| Diabetes mellitus | |  |  | 1.02 (0.57 – 1.73) | 0.941 | 1.05 (0.56 – 1.84) | 0.865 | 1.16 (0.63 – 1.99) | 0.618 | 0.57 (0.09 – 2.14) | 0.472 |
| Arterial hypertension | |  |  | 2.80 (1.77 – 4.62) | **<0.001** | 2.35 (1.47 – 3.92) | **0.001** | 2.43 (1.53 – 4.04) | **<0.001** |  |  |
| Current smoking | |  |  | 0.95 (0.57 – 1.51) | 0.823 | 0.86 (0.48 – 1.46) | 0.601 | 0.91 (0.51 – 1.52) | 0.731 | 1.22 (0.39 – 3.14) | 0.697 |
| BMI | |  |  | 1.03 (0.99 – 1.06) | 0.104 | 1.03 (0.99 – 1.06) | 0.163 | 1.02 (0.99 – 1.06) | 0.204 | 1.04 (0.97 – 1.11) | 0.260 |
| Additives | **Milk** |  |  |  |  | 1.50 (1.01 – 2.29) | 0.052 |  |  |  |  |
|  | **Milk + Sweetener** |  |  |  |  | 1.08 (0.36 – 2.65) | 0.877 |  |  |  |  |
|  | **Sweetener** |  |  |  |  | 2.52 (0.70 – 7.08) | 0.108 |  |  |  |  |
|  | **Binary** |  |  |  |  |  |  | 1.45 (0.98 – 2.19) | 0.073 |  |  |

**Supplementary Table 99. Multivariable logistic regression analysis for the association of mild/moderate/high coffee consumption and atrial fibrillation (only female).** Not daily coffee consumption (<1 cups/d) served as the reference group. Adjustment was performed for: A) unadjusted; B) main cardiovascular risk factors; C) additional adjustment for additives (separately), D) additional adjustment for additives (binary), E) same adjustment as for group A, but only for subjects who consumed coffee without any additives. Abbreviations as in Table 1.

|  | | A)Diabetes | | B)Diabetes | | C)Diabetes | | D)Diabetes | | E)Diabetes | |
| --- | --- | --- | --- | --- | --- | --- | --- | --- | --- | --- | --- |
|  | | *Odds Ratios* | *p* | *Odds Ratios* | *p* | *Odds Ratios* | *p* | *Odds Ratios* | *p* | *Odds Ratios* | *p* |
| Coffee consumption 3-4 cups/day | | 0.79 (0.58 – 1.07) | 0.136 | 0.96 (0.68 – 1.34) | 0.808 | 0.90 (0.62 – 1.29) | 0.574 | 0.90 (0.62 – 1.28) | 0.555 | 1.14 (0.59 – 2.13) | 0.695 |
| Coffee consumption >4 cups/day | | 0.90 (0.54 – 1.41) | 0.661 | 0.96 (0.54 – 1.61) | 0.873 | 0.80 (0.42 – 1.43) | 0.471 | 0.86 (0.46 – 1.52) | 0.624 | 0.66 (0.19 – 1.86) | 0.473 |
| Age | |  |  | 1.06 (1.03 – 1.08) | **<0.001** | 1.06 (1.03 – 1.08) | **<0.001** | 1.06 (1.04 – 1.08) | **<0.001** | 1.07 (1.02 – 1.11) | **0.003** |
| Arterial hypertension | |  |  | 2.34 (1.58 – 3.57) | **<0.001** | 2.42 (1.59 – 3.79) | **<0.001** | 2.49 (1.63 – 3.93) | **<0.001** | 1.34 (0.66 – 2.90) | 0.440 |
| Current smoking | |  |  | 1.51 (1.03 – 2.16) | **0.029** | 1.61 (1.08 – 2.37) | **0.017** | 1.60 (1.07 – 2.35) | **0.020** | 1.12 (0.49 – 2.39) | 0.771 |
| BMI | |  |  | 1.17 (1.14 – 1.20) | **<0.001** | 1.16 (1.13 – 1.19) | **<0.001** | 1.16 (1.13 – 1.19) | **<0.001** | 1.19 (1.14 – 1.26) | **<0.001** |
| Additives | **Milk** |  |  |  |  | 1.05 (0.75 – 1.49) | 0.764 |  |  |  |  |
|  | **Milk + Sugar** |  |  |  |  | 0.92 (0.45 – 1.74) | 0.811 |  |  |  |  |
|  | **Milk + Sweetener** |  |  |  |  | 1.37 (0.68 – 2.61) | 0.348 |  |  |  |  |
|  | **Sweetener** |  |  |  |  | 1.25 (0.38 – 3.33) | 0.685 |  |  |  |  |
|  | **Binary** |  |  |  |  |  |  | 1.05 (0.76 – 1.47) | 0.765 |  |  |

**Supplementary Table 100.. Multivariable logistic regression analysis for the association of mild/moderate/high coffee consumption and diabetes (only female).** Not daily coffee consumption (<1 cups/d) served as the reference group. Adjustment was performed for: A) unadjusted; B) main cardiovascular risk factors; C) additional adjustment for additives (separately), D) additional adjustment for additives (binary), E) same adjustment as for group A, but only for subjects who consumed coffee without any additives. Abbreviations as in Table 1.

|  | | A)Obesity | | B)Obesity | | C)Obesity | | D)Obesity | | E)Obesity | |
| --- | --- | --- | --- | --- | --- | --- | --- | --- | --- | --- | --- |
|  | | *Odds Ratios* | *p* | *Odds Ratios* | *p* | *Odds Ratios* | *p* | *Odds Ratios* | *p* | *Odds Ratios* | *p* |
| Coffee consumption 3-4 cups/day | | 1.12 (0.94 – 1.33) | 0.208 | 1.20 (0.98 – 1.46) | 0.079 | 1.21 (0.97 – 1.49) | 0.084 | 1.18 (0.95 – 1.45) | 0.131 | 1.07 (0.71 – 1.59) | 0.752 |
| Coffee consumption >4 cups/day | | 1.40 (1.07 – 1.82) | **0.014** | 1.62 (1.19 – 2.20) | **0.002** | 1.61 (1.16 – 2.23) | **0.004** | 1.66 (1.19 – 2.29) | **0.002** | 1.79 (0.99 – 3.14) | **0.048** |
| Age | |  |  | 0.98 (0.97 – 0.99) | **<0.001** | 0.98 (0.96 – 0.99) | **<0.001** | 0.98 (0.96 – 0.99) | **<0.001** | 0.98 (0.96 – 1.01) | 0.177 |
| Diabetes mellitus | |  |  | 5.94 (4.48 – 7.91) | **<0.001** | 5.86 (4.34 – 7.94) | **<0.001** | 5.84 (4.32 – 7.91) | **<0.001** | 9.78 (5.45 – 18.14) | **<0.001** |
| Arterial hypertension | |  |  | 3.44 (2.80 – 4.25) | **<0.001** | 3.28 (2.65 – 4.10) | **<0.001** | 3.29 (2.65 – 4.10) | **<0.001** | 3.49 (2.30 – 5.41) | **<0.001** |
| Current smoking | |  |  | 0.88 (0.70 – 1.11) | 0.283 | 0.82 (0.64 – 1.04) | 0.106 | 0.83 (0.65 – 1.06) | 0.147 | 1.00 (0.62 – 1.57) | 0.986 |
| Additives | **Milk** |  |  |  |  | 0.85 (0.69 – 1.05) | 0.122 |  |  |  |  |
|  | **Milk + Sugar** |  |  |  |  | 1.04 (0.71 – 1.49) | 0.856 |  |  |  |  |
|  | **Milk + Sweetener** |  |  |  |  | 2.07 (1.32 – 3.20) | **0.001** |  |  |  |  |
|  | **Sugar** |  |  |  |  | 1.92 (0.89 – 3.95) | 0.084 |  |  |  |  |
|  | **Sweetener** |  |  |  |  | 1.57 (0.74 – 3.14) | 0.216 |  |  |  |  |
|  | **Binary** |  |  |  |  |  |  | 0.93 (0.76 – 1.14) | 0.481 |  |  |

**Supplementary Table 101. Multivariable logistic regression analysis for the association of mild/moderate/high coffee consumption and obesity (only female).** Not daily coffee consumption (<1 cups/d) served as the reference group. Adjustment was performed for: A) unadjusted; B) main cardiovascular risk factors; C) additional adjustment for additives (separately), D) additional adjustment for additives (binary), E) same adjustment as for group A, but only for subjects who consumed coffee without any additives. Abbreviations as in Table 1.

|  | | A)CAD | | B)CAD | | C)CAD | | D)CAD | | E)CAD | |
| --- | --- | --- | --- | --- | --- | --- | --- | --- | --- | --- | --- |
|  | | *Odds Ratios* | *p* | *Odds Ratios* | *p* | *Odds Ratios* | *p* | *Odds Ratios* | *p* | *Odds Ratios* | *p* |
| Coffee consumption 3-4 cups/day | | 0.75 (0.48 – 1.14) | 0.196 | 0.95 (0.58 – 1.51) | 0.837 | 0.90 (0.53 – 1.48) | 0.690 | 0.90 (0.53 – 1.48) | 0.692 | 0.66 (0.22 – 1.71) | 0.420 |
| Coffee consumption >4 cups/day | | 0.60 (0.25 – 1.22) | 0.202 | 0.78 (0.29 – 1.75) | 0.589 | 0.74 (0.25 – 1.78) | 0.545 | 0.75 (0.25 – 1.80) | 0.557 | 0.23 (0.01 – 1.43) | 0.188 |
| Age | |  |  | 1.10 (1.07 – 1.14) | **<0.001** | 1.10 (1.07 – 1.14) | **<0.001** | 1.10 (1.07 – 1.14) | **<0.001** | 1.17 (1.08 – 1.27) | **<0.001** |
| Diabetes mellitus | |  |  | 1.72 (0.94 – 3.00) | 0.064 | 1.70 (0.89 – 3.05) | 0.089 | 1.77 (0.93 – 3.19) | 0.067 | 1.69 (0.45 – 5.23) | 0.392 |
| Arterial hypertension | |  |  | 3.57 (1.97 – 7.03) | **<0.001** | 3.40 (1.83 – 6.94) | **<0.001** | 3.31 (1.78 – 6.76) | **<0.001** | 12.60 (2.52 – 229.49) | **0.015** |
| Current smoking | |  |  | 1.48 (0.86 – 2.46) | 0.143 | 1.35 (0.73 – 2.39) | 0.313 | 1.32 (0.70 – 2.34) | 0.366 | 4.02 (1.35 – 11.28) | **0.009** |
| BMI | |  |  | 1.02 (0.98 – 1.06) | 0.267 | 1.02 (0.98 – 1.06) | 0.333 | 1.02 (0.98 – 1.07) | 0.294 | 0.99 (0.90 – 1.07) | 0.762 |
| Additives | **Milk** |  |  |  |  | 1.05 (0.66 – 1.72) | 0.839 |  |  |  |  |
|  | **Milk + Sugar** |  |  |  |  | 0.92 (0.30 – 2.29) | 0.863 |  |  |  |  |
|  | **Milk + Sweetener** |  |  |  |  | 1.75 (0.63 – 4.22) | 0.241 |  |  |  |  |
|  | **Sweetener** |  |  |  |  | 1.78 (0.40 – 5.75) | 0.381 |  |  |  |  |
|  | **Binary** |  |  |  |  |  |  | 1.08 (0.69 – 1.75) | 0.738 |  |  |

**Supplementary Table 102. Multivariable logistic regression analysis for the association of mild/moderate/high coffee consumption and CAD (only female).** Not daily coffee consumption (<1 cups/d) served as the reference group. Adjustment was performed for: A) unadjusted; B) main cardiovascular risk factors; C) additional adjustment for additives (separately), D) additional adjustment for additives (binary), E) same adjustment as for group A, but only for subjects who consumed coffee without any additives. Abbreviations as in Table 1.

|  | | A)PAD | | B)PAD | | C)PAD | | D)PAD | | E)PAD | |
| --- | --- | --- | --- | --- | --- | --- | --- | --- | --- | --- | --- |
|  | | *Odds Ratios* | *p* | *Odds Ratios* | *p* | *Odds Ratios* | *p* | *Odds Ratios* | *p* | *Odds Ratios* | *p* |
| Coffee consumption 3-4 cups/day | | 1.04 (0.81 – 1.33) | 0.764 | 1.29 (0.98 – 1.71) | 0.071 | 1.36 (1.01 – 1.83) | **0.041** | 1.33 (0.99 – 1.78) | 0.059 | 1.31 (0.76 – 2.22) | 0.327 |
| Coffee consumption >4 cups/day | | 1.16 (0.78 – 1.69) | 0.456 | 1.32 (0.83 – 2.05) | 0.222 | 1.39 (0.84 – 2.22) | 0.184 | 1.34 (0.82 – 2.14) | 0.230 | 0.54 (0.17 – 1.38) | 0.230 |
| Age | |  |  | 1.04 (1.02 – 1.06) | **<0.001** | 1.04 (1.02 – 1.06) | **<0.001** | 1.04 (1.02 – 1.06) | **<0.001** | 1.06 (1.02 – 1.09) | **0.002** |
| Diabetes mellitus | |  |  | 1.62 (1.03 – 2.53) | **0.033** | 1.45 (0.89 – 2.32) | 0.127 | 1.47 (0.90 – 2.35) | 0.116 | 1.25 (0.48 – 3.08) | 0.642 |
| Arterial hypertension | |  |  | 1.58 (1.20 – 2.08) | **0.001** | 1.76 (1.32 – 2.37) | **<0.001** | 1.68 (1.26 – 2.26) | **<0.001** | 1.53 (0.91 – 2.62) | 0.117 |
| Current smoking | |  |  | 1.20 (0.87 – 1.62) | 0.259 | 1.32 (0.95 – 1.83) | 0.093 | 1.31 (0.94 – 1.81) | 0.103 | 1.36 (0.70 – 2.56) | 0.351 |
| BMI | |  |  | 1.04 (1.01 – 1.06) | **0.004** | 1.03 (1.01 – 1.06) | **0.016** | 1.04 (1.01 – 1.06) | **0.012** | 1.04 (0.99 – 1.10) | 0.130 |
| Additives | **Milk** |  |  |  |  | 0.97 (0.73 – 1.29) | 0.825 |  |  |  |  |
|  | **Milk + Sugar** |  |  |  |  | 0.79 (0.44 – 1.37) | 0.413 |  |  |  |  |
|  | **Milk + Sweetener** |  |  |  |  | 0.99 (0.46 – 2.01) | 0.979 |  |  |  |  |
|  | **Sweetener** |  |  |  |  | 1.91 (0.72 – 4.82) | 0.178 |  |  |  |  |
|  | **Binary** |  |  |  |  |  |  | 0.96 (0.73 – 1.27) | 0.777 |  |  |

**Supplementary Table 103. Multivariable logistic regression analysis for the association of mild/moderate/high coffee consumption and PAD (only female).** Not daily coffee consumption (<1 cups/d) served as the reference group. Adjustment was performed for: A) unadjusted; B) main cardiovascular risk factors; C) additional adjustment for additives (separately), D) additional adjustment for additives (binary), E) same adjustment as for group A, but only for subjects who consumed coffee without any additives. Abbreviations as in Table 1.

|  | | A)Heart failure | | B)Heart failure | | C)Heart failure | | D)Heart failure | | E)Heart failure | |
| --- | --- | --- | --- | --- | --- | --- | --- | --- | --- | --- | --- |
|  | | *Odds Ratios* | *p* | *Odds Ratios* | *p* | *Odds Ratios* | *p* | *Odds Ratios* | *p* | *Odds Ratios* | *p* |
| Coffee consumption 3-4 cups/day | | 0.75 (0.46 – 1.18) | 0.225 | 0.92 (0.53 – 1.53) | 0.766 | 0.84 (0.46 – 1.44) | 0.538 | 0.86 (0.48 – 1.49) | 0.608 | 0.73 (0.23 – 1.94) | 0.556 |
| Coffee consumption >4 cups/day | | 1.02 (0.49 – 1.90) | 0.952 | 1.36 (0.60 – 2.78) | 0.426 | 1.40 (0.58 – 3.00) | 0.412 | 1.41 (0.58 – 3.02) | 0.409 | 0.82 (0.12 – 3.29) | 0.806 |
| Age | |  |  | 1.08 (1.05 – 1.12) | **<0.001** | 1.07 (1.04 – 1.11) | **<0.001** | 1.08 (1.04 – 1.11) | **<0.001** | 1.10 (1.03 – 1.18) | **0.007** |
| Diabetes mellitus | |  |  | 2.87 (1.66 – 4.81) | **<0.001** | 2.60 (1.44 – 4.52) | **0.001** | 2.63 (1.46 – 4.58) | **0.001** | 3.28 (0.95 – 9.81) | **0.043** |
| Arterial hypertension | |  |  | 7.10 (3.29 – 18.54) | **<0.001** | 7.55 (3.29 – 21.84) | **<0.001** | 7.42 (3.24 – 21.50) | **<0.001** |  |  |
| Current smoking | |  |  | 1.49 (0.85 – 2.51) | 0.150 | 1.38 (0.74 – 2.45) | 0.283 | 1.35 (0.72 – 2.40) | 0.331 | 1.93 (0.59 – 5.40) | 0.233 |
| BMI | |  |  | 1.06 (1.01 – 1.10) | **0.007** | 1.07 (1.02 – 1.11) | **0.002** | 1.06 (1.02 – 1.10) | **0.006** | 1.01 (0.92 – 1.09) | 0.875 |
| Additives | **Milk** |  |  |  |  | 1.15 (0.70 – 1.94) | 0.584 |  |  |  |  |
|  | **Milk + Sugar** |  |  |  |  | 1.10 (0.36 – 2.82) | 0.850 |  |  |  |  |
|  | **Milk + Sweetener** |  |  |  |  | 0.67 (0.15 – 2.07) | 0.539 |  |  |  |  |
|  | **Sweetener** |  |  |  |  | 2.39 (0.52 – 8.11) | 0.199 |  |  |  |  |
|  | **Binary** |  |  |  |  |  |  | 1.09 (0.67 – 1.81) | 0.742 |  |  |

**Supplementary Table 104. Multivariable logistic regression analysis for the association of mild/moderate/high coffee consumption and heart failure (only female).** Not daily coffee consumption (<1 cups/d) served as the reference group. Adjustment was performed for: A) unadjusted; B) main cardiovascular risk factors; C) additional adjustment for additives (separately), D) additional adjustment for additives (binary), E) same adjustment as for group A, but only for subjects who consumed coffee without any additives. Abbreviations as in Table 1.

|  | | A)HF(m)rEF | | B)HF(m)rEF | | C)HF(m)rEF | | D)HF(m)rEF | | E)HF(m)rEF | |
| --- | --- | --- | --- | --- | --- | --- | --- | --- | --- | --- | --- |
|  | | *Odds Ratios* | *p* | *Odds Ratios* | *p* | *Odds Ratios* | *p* | *Odds Ratios* | *p* | *Odds Ratios* | *p* |
| Coffee consumption 3-4 cups/day | | 0.74 (0.31 – 1.55) | 0.451 | 1.00 (0.39 – 2.23) | 0.991 | 0.90 (0.33 – 2.15) | 0.831 | 0.90 (0.32 – 2.13) | 0.817 | 1.54 (0.31 – 6.47) | 0.560 |
| Coffee consumption > 4 cups/day | | 0.86 (0.20 – 2.44) | 0.803 | 1.45 (0.33 – 4.43) | 0.565 | 1.64 (0.37 – 5.13) | 0.449 | 1.58 (0.36 – 4.98) | 0.481 | 1.61 (0.08 – 11.07) | 0.676 |
| Age | |  |  | 1.05 (1.00 – 1.11) | **0.043** | 1.04 (0.99 – 1.10) | 0.131 | 1.04 (0.99 – 1.10) | 0.108 | 1.04 (0.95 – 1.15) | 0.422 |
| Diabetes mellitus | |  |  | 2.69 (1.11 – 6.02) | **0.021** | 2.41 (0.90 – 5.73) | 0.060 | 2.35 (0.88 – 5.64) | 0.068 | 1.22 (0.06 – 8.32) | 0.862 |
| Arterial hypertension | |  |  | 15.68 (3.24 – 282.73) | **0.007** | 14.83 (3.03 – 268.16) | **0.009** | 14.88 (3.04 – 269.02) | **0.009** |  |  |
| Current smoking | |  |  | 0.69 (0.20 – 1.85) | 0.503 | 0.75 (0.21 – 2.08) | 0.615 | 0.77 (0.22 – 2.12) | 0.647 | 0.60 (0.03 – 3.62) | 0.639 |
| BMI | |  |  | 1.06 (1.00 – 1.13) | **0.042** | 1.06 (0.99 – 1.13) | 0.074 | 1.06 (0.99 – 1.13) | 0.097 | 1.04 (0.90 – 1.17) | 0.579 |
| Additives | **Milk** |  |  |  |  | 1.06 (0.49 – 2.50) | 0.883 |  |  |  |  |
|  | **Milk + Sugar** |  |  |  |  | 0.56 (0.03 – 3.13) | 0.591 |  |  |  |  |
|  | **Milk + Sweetener** |  |  |  |  | 0.65 (0.03 – 3.65) | 0.689 |  |  |  |  |
|  | **Sweetener** |  |  |  |  | 2.39 (0.12 – 14.49) | 0.429 |  |  |  |  |
|  | **Binary** |  |  |  |  |  |  | 1.00 (0.47 – 2.32) | 0.999 |  |  |

**Supplementary Table 105. Multivariable logistic regression analysis for the association of mild/moderate/high coffee consumption and HF(m)rEF (only female).** Not daily coffee consumption (<1 cups/d) served as the reference group. Adjustment was performed for: A) unadjusted; B) main cardiovascular risk factors; C) additional adjustment for additives (separately), D) additional adjustment for additives (binary), E) same adjustment as for group A, but only for subjects who consumed coffee without any additives. Abbreviations as in Table 1.

|  | COFFEE CONSUMPTION | | | |  |
| --- | --- | --- | --- | --- | --- |
|  | **Overall** | **< 3 cups/day** | **3-4 cups/day** | **> 4 cups/day** | **p-values** |
| N(%) | 4399 | 2600 | 1185 | 614 |  |
| DEMOGRAPHICS + BIOLOGICAL DATA | | | | | |
| Age | 63.0 [56.0, 70.0] | 66.0 [58.0, 71.0] | 60.0 [54.0, 67.0] | 59.0 [53.0, 66.0] | <0.001 |
| BMI kg/m2 | 26.6 [24.5, 29.4] | 26.6 [24.5, 29.4] | 26.5 [24.4, 29.3] | 26.9 [24.7, 29.4] | 0.175 |
| Smoking current | 858 (19.6) | 397 (15.4) | 265 (22.4) | 196 (32.1) | <0.001 |
| CARDIOVASCULAR DISEASES | | | | | |
| Arterial hypertension | 3039 (72.5) | 1881 (75.3) | 771 (69.0) | 387 (67.5) | <0.001 |
| Diabetes mellitus | 432 (10.6) | 291 (12.0) | 91 ( 8.4) | 50 ( 8.9) | 0.002 |
| Myocardial_infarction | 213 ( 4.9) | 139 ( 5.4) | 44 ( 3.7) | 30 ( 4.9) | 0.088 |
| CAD | 450 (13.2) | 298 (14.8) | 97 (10.4) | 55 (11.5) | 0.003 |
| PAD | 399 (18.7) | 248 (19.5) | 103 (17.7) | 48 (16.8) | 0.454 |
| LABORATORIES | | | | | |
| Cholesterol, mg/dl | 199.0 [170.0, 225.0] | 197.0 [169.0, 223.0] | 200.0 [172.0, 227.0] | 202.0 [176.0, 228.0] | 0.003 |
| LDL, mg/dl | 117.0 [91.0, 141.0] | 115.0 [89.0, 140.0] | 119.0 [94.5, 144.0] | 123.0 [97.5, 145.5] | <0.001 |
| HDL, mg/dl | 53.5 [45.0, 64.0] | 54.0 [46.0, 65.0] | 54.0 [45.0, 65.0] | 52.0 [43.2, 62.0] | 0.006 |
| NT-proBNP, g/dl | 63.0 [34.0, 122.0] | 73.0 [39.0, 140.0] | 54.5 [30.0, 101.8] | 50.0 [30.0, 98.5] | <0.001 |
| Hemoglobin, g/dl | 15.0 [14.4, 15.6] | 15.0 [14.3, 15.6] | 15.0 [14.4, 15.6] | 15.0 [14.4, 15.6] | 0.790 |
| MEDICATION | | | | | |
| ACEi/ ARBs | 1014 (24.3) | 635 (25.6) | 252 (22.6) | 127 (22.2) | 0.063 |
| Beta Blockers | 781 (18.7) | 527 (21.2) | 176 (15.8) | 78 (13.6) | <0.001 |
| Diuretics | 89 ( 2.1) | 62 ( 2.5) | 18 ( 1.6) | 9 ( 1.6) | 0.141 |
| Lipid modifying drugs | 942 (22.6) | 631 (25.4) | 200 (17.9) | 111 (19.4) | <0.001 |
| ADDTIVES | | | | | |
| Milk | 2814 (67.5) | 1695 (71.2) | 749 (63.5) | 370 (60.4) | <0.001 |
| Sugar | 687 (16.5) | 435 (18.3) | 180 (15.3) | 72 (11.7) | <0.001 |
| Honey | 45 ( 1.1) | 34 ( 1.4) | 10 ( 0.8) | 1 ( 0.2) | 0.017 |
| Sweetener | 204 ( 4.9) | 118 ( 5.0) | 51 ( 4.3) | 35 ( 5.7) | 0.424 |
| No additives | 1464 (35.1) | 725 (30.5) | 462 (39.2) | 277 (45.2) | <0.001 |
| BLACK/GREEN TEA | | | | | |
| Never | 735 (16.8) | 403 (15.6) | 209 (17.8) | 123 (20.1) | <0.001 |
| 1-3/week | 2133 (48.8) | 1091 (42.2) | 676 (57.4) | 366 (59.8) | <0.001 |
| >4/week | 1505 (34.4) | 1090 (42.2) | 292 (24.8) | 123 (20.1) | <0.001 |
| CARBONATED DRINKS | | | | | |
| Never | 1727 (39.5) | 1084 (41.9) | 431 (36.5) | 212 (34.9) | 0.002 |
| 1-3/week | 2242 (51.3) | 1278 (49.4) | 626 (53.1) | 338 (55.6) | 0.002 |
| ≥4/week | 405 ( 9.3) | 224 ( 8.7) | 123 (10.4) | 58 ( 9.5) | 0.002 |

**Supplementary Table 106. Baseline characteristics** **of the study population only male.** Continuous variables are presented as median and interquartile range, and categorical variables are presented as absolute numbers and percentages. Abbreviations as in Table 1.

|  | | A)Total cholesterol | | B)Total cholesterol | | C)Total cholesterol | | D)Total cholesterol | | E)Total cholesterol | | F)Total cholesterol | |
| --- | --- | --- | --- | --- | --- | --- | --- | --- | --- | --- | --- | --- | --- |
|  | | *Estimates* | *p* | *Estimates* | *p* | *Estimates* | *p* | *Estimates* | *p* | *Estimates* | *p* | *Estimates* | *p* |
| Coffee consumption 3-4 cups/day | | 3.73 (0.94 – 6.52) | **0.009** | 0.46 (-2.48 – 3.41) | 0.757 | 0.19 (-2.93 – 3.30) | 0.906 | 0.29 (-2.83 – 3.41) | 0.854 | -0.84 (-3.61 – 1.94) | 0.554 | 0.68 (-4.91 – 6.27) | 0.810 |
| Coffee consumption >4 cups/da | | 5.62 (2.04 – 9.19) | **0.002** | 3.56 (-0.26 – 7.37) | 0.068 | 3.78 (-0.29 – 7.85) | 0.069 | 3.75 (-0.33 – 7.83) | 0.072 | 3.10 (-0.49 – 6.69) | 0.090 | 0.40 (-6.31 – 7.11) | 0.907 |
| Age | |  |  | -0.53 (-0.69 – -0.37) | **<0.001** | -0.54 (-0.72 – -0.37) | **<0.001** | -0.54 (-0.72 – -0.37) | **<0.001** | -0.20 (-0.35 – -0.05) | **0.011** | -0.40 (-0.72 – -0.07) | **0.016** |
| Diabetes mellitus | |  |  | -21.19 (-25.44 – -16.94) | **<0.001** | -20.76 (-25.37 – -16.15) | **<0.001** | -20.91 (-25.50 – -16.32) | **<0.001** | -15.17 (-19.25 – -11.10) | **<0.001** | -29.37 (-37.55 – -21.20) | **<0.001** |
| Arterial hypertension | |  |  | 0.58 (-2.40 – 3.55) | 0.703 | 1.58 (-1.59 – 4.75) | 0.328 | 1.82 (-1.36 – 4.99) | 0.262 | 4.40 (1.58 – 7.22) | **0.002** | 2.67 (-3.19 – 8.53) | 0.372 |
| Current smoking | |  |  | -0.44 (-3.67 – 2.79) | 0.789 | -1.65 (-5.10 – 1.80) | 0.348 | -1.94 (-5.39 – 1.51) | 0.271 | -0.11 (-3.16 – 2.93) | 0.941 | -1.97 (-8.20 – 4.25) | 0.534 |
| BMI | |  |  | -0.43 (-0.75 – -0.11) | **0.009** | -0.50 (-0.85 – -0.16) | **0.004** | -0.52 (-0.86 – -0.18) | **0.003** | -0.12 (-0.42 – 0.19) | 0.454 | -0.65 (-1.25 – -0.04) | **0.036** |
| Additives | **Milk** |  |  |  |  | 0.60 (-2.43 – 3.64) | 0.696 |  |  |  |  |  |  |
|  | **Milk + Sugar** |  |  |  |  | -1.59 (-6.26 – 3.07) | 0.503 |  |  |  |  |  |  |
|  | **Milk + Sweetener** |  |  |  |  | -3.21 (-10.91 – 4.48) | 0.413 |  |  |  |  |  |  |
|  | **Sugar** |  |  |  |  | 4.80 (-4.74 – 14.35) | 0.324 |  |  |  |  |  |  |
|  | **Sweetener** |  |  |  |  | -2.48 (-15.01 – 10.05) | 0.698 |  |  |  |  |  |  |
|  | **Binary** |  |  |  |  |  |  | 0.08 (-2.82 – 2.97) | 0.957 |  |  |  |  |
| Lipid lowering drugs | |  |  |  |  |  |  |  |  | -34.31 (-37.34 – -31.28) | **<0.001** |  |  |

**Supplementary Table 107. Multivariable linear regression analysis for the association of mild/moderate/high coffee consumption and total cholesterol (only male).** “Not daily” coffee consumption (<1 cups/d) served as the reference group. Adjustment was performed for: A) unadjusted; B) main cardiovascular risk factors; C) additional adjustment for additives (separately), D) additional adjustment for additives (binary), E) additional adjustment for lipid lowering drugs, F) same adjustment as for group A, but only for subjects who consumed coffee without any additives. Abbreviations as in Table 1

|  | | A)LDL | | B)LDL | | C)LDL | | D)LDL | | E)LDL | | F)LDL | |
| --- | --- | --- | --- | --- | --- | --- | --- | --- | --- | --- | --- | --- | --- |
|  | | *Estimates* | *p* | *Estimates* | *p* | *Estimates* | *p* | *Estimates* | *p* | *Estimates* | *p* | *Estimates* | *p* |
| Coffee consumption 3-4 cups/day | | 4.73 (2.17 – 7.29) | **<0.001** | 1.32 (-1.37 – 4.02) | 0.337 | 1.27 (-1.57 – 4.10) | 0.382 | 1.18 (-1.66 – 4.02) | 0.415 | 0.02 (-2.49 – 2.53) | 0.990 | 2.25 (-2.88 – 7.38) | 0.390 |
| Coffee consumption >4 cups/day | | 7.04 (3.75 – 10.32) | **<0.001** | 4.39 (0.90 – 7.89) | **0.014** | 4.82 (1.10 – 8.54) | **0.011** | 4.68 (0.95 – 8.41) | **0.014** | 3.82 (0.57 – 7.08) | **0.021** | 3.14 (-3.01 – 9.29) | 0.317 |
| Age | |  |  | -0.52 (-0.67 – -0.38) | **<0.001** | -0.53 (-0.69 – -0.38) | **<0.001** | -0.54 (-0.69 – -0.38) | **<0.001** | -0.21 (-0.34 – -0.07) | **0.004** | -0.29 (-0.59 – 0.01) | 0.055 |
| Diabetes mellitus | |  |  | -22.81 (-26.77 – -18.85) | **<0.001** | -22.25 (-26.53 – -17.97) | **<0.001** | -22.44 (-26.70 – -18.17) | **<0.001** | -16.01 (-19.77 – -12.25) | **<0.001** | -30.61 (-38.24 – -22.99) | **<0.001** |
| Arterial hypertension | |  |  | -1.16 (-3.88 – 1.56) | 0.403 | -0.42 (-3.31 – 2.47) | 0.775 | -0.24 (-3.14 – 2.65) | 0.870 | 2.72 (0.17 – 5.27) | **0.037** | -0.42 (-5.78 – 4.93) | 0.877 |
| Current smoking | |  |  | 0.38 (-2.58 – 3.34) | 0.802 | -0.48 (-3.64 – 2.67) | 0.764 | -0.50 (-3.65 – 2.65) | 0.757 | 1.15 (-1.61 – 3.91) | 0.414 | -1.82 (-7.56 – 3.93) | 0.535 |
| BMI | |  |  | -0.14 (-0.43 – 0.15) | 0.354 | -0.17 (-0.48 – 0.14) | 0.282 | -0.19 (-0.50 – 0.12) | 0.237 | 0.16 (-0.12 – 0.43) | 0.258 | -0.18 (-0.74 – 0.37) | 0.518 |
| Additives | **Milk** |  |  |  |  | 1.69 (-1.07 – 4.46) | 0.231 |  |  |  |  |  |  |
|  | **Milk + Sugar** |  |  |  |  | 1.85 (-2.40 – 6.11) | 0.393 |  |  |  |  |  |  |
|  | **Milk + Sweetener** |  |  |  |  | -1.44 (-8.55 – 5.67) | 0.692 |  |  |  |  |  |  |
|  | **Sugar** |  |  |  |  | 4.47 (-4.18 – 13.12) | 0.311 |  |  |  |  |  |  |
|  | **Sweetener** |  |  |  |  | -3.49 (-14.98 – 8.01) | 0.552 |  |  |  |  |  |  |
|  | **Binary** |  |  |  |  |  |  | 1.55 (-1.08 – 4.19) | 0.248 |  |  |  |  |
| Lipid lowering drugs | |  |  |  |  |  |  |  |  | -33.92 (-36.67 – -31.17) | **<0.001** |  |  |

**Supplementary Table 108. Multivariable linear regression analysis for the association of mild/moderate/high coffee consumption and LDL (only male).** “Not daily” coffee consumption (<1 cups/d) served as the reference group. Adjustment was performed for: A) unadjusted; B) main cardiovascular risk factors; C) additional adjustment for additives (separately), D) additional adjustment for additives (binary), E) additional adjustment for lipid lowering drugs, F) same adjustment as for group A, but only for subjects who consumed coffee without any additives. Abbreviations as in Table 1

|  | | A)HDL | | B)HDL | | C)HDL | | D)HDL | | E)HDL | | F)HDL | |
| --- | --- | --- | --- | --- | --- | --- | --- | --- | --- | --- | --- | --- | --- |
|  | | *Estimates* | *p* | *Estimates* | *p* | *Estimates* | *p* | *Estimates* | *p* | *Estimates* | *p* | *Estimates* | *p* |
| Coffee consumption3-4 cups/day | | -0.04 (-1.09 – 1.02) | 0.942 | 0.82 (-0.24 – 1.87) | 0.128 | 0.48 (-0.65 – 1.60) | 0.405 | 0.60 (-0.53 – 1.72) | 0.299 | 0.85 (-0.20 – 1.89) | 0.111 | -0.04 (-2.18 – 2.11) | 0.974 |
| Coffee consumption >4 cups/day | | -1.98 (-3.33 – -0.63) | **0.004** | 0.08 (-1.28 – 1.44) | 0.909 | -0.62 (-2.08 – 0.85) | 0.410 | -0.56 (-2.04 – 0.91) | 0.453 | 0.16 (-1.19 – 1.51) | 0.816 | 0.38 (-2.20 – 2.95) | 0.773 |
| Age | |  |  | 0.18 (0.12 – 0.23) | **<0.001** | 0.18 (0.11 – 0.24) | **<0.001** | 0.18 (0.12 – 0.24) | **<0.001** | 0.20 (0.14 – 0.26) | **<0.001** | 0.11 (-0.02 – 0.23) | 0.085 |
| Diabetes mellitus | |  |  | -6.32 (-7.84 – -4.80) | **<0.001** | -6.50 (-8.16 – -4.84) | **<0.001** | -6.44 (-8.09 – -4.78) | **<0.001** | -6.18 (-7.71 – -4.65) | **<0.001** | -7.78 (-10.91 – -4.64) | **<0.001** |
| Arterial hypertension | |  |  | -0.34 (-1.40 – 0.73) | 0.535 | -0.28 (-1.42 – 0.86) | 0.633 | -0.22 (-1.37 – 0.93) | 0.707 | -0.17 (-1.23 – 0.89) | 0.756 | 0.10 (-2.15 – 2.35) | 0.931 |
| Current smoking | |  |  | -4.09 (-5.24 – -2.93) | **<0.001** | -4.05 (-5.29 – -2.80) | **<0.001** | -4.25 (-5.50 – -3.01) | **<0.001** | -4.21 (-5.36 – -3.07) | **<0.001** | -4.09 (-6.48 – -1.70) | **0.001** |
| BMI | |  |  | -1.22 (-1.33 – -1.10) | **<0.001** | -1.24 (-1.36 – -1.12) | **<0.001** | -1.24 (-1.36 – -1.11) | **<0.001** | -1.18 (-1.29 – -1.07) | **<0.001** | -1.26 (-1.49 – -1.02) | **<0.001** |
| Additives | **Milk** |  |  |  |  | -1.34 (-2.43 – -0.24) | **0.017** |  |  |  |  |  |  |
|  | **Milk + Sugar** |  |  |  |  | -4.22 (-5.90 – -2.54) | **<0.001** |  |  |  |  |  |  |
|  | **Milk + Sweetener** |  |  |  |  | -3.99 (-6.76 – -1.21) | **0.005** |  |  |  |  |  |  |
|  | **Sugar** |  |  |  |  | -0.64 (-4.08 – 2.80) | 0.715 |  |  |  |  |  |  |
|  | **Sweetener** |  |  |  |  | 3.42 (-1.10 – 7.93) | 0.138 |  |  |  |  |  |  |
|  | **Binary** |  |  |  |  |  |  | -1.88 (-2.92 – -0.83) | **<0.001** |  |  |  |  |
| Lipid lowering drugs | |  |  |  |  |  |  |  |  | -2.05 (-3.18 – -0.91) | **<0.001** |  |  |

**Supplementary Table 109. Multivariable linear regression analysis for the association of mild/moderate/high coffee consumption and HDL (only male).** “Not daily” coffee consumption (<1 cups/d) served as the reference group. Adjustment was performed for: A) unadjusted; B) main cardiovascular risk factors; C) additional adjustment for additives (separately), D) additional adjustment for additives (binary), E) additional adjustment for lipid lowering drugs, F) same adjustment as for group A, but only for subjects who consumed coffee without any additives. Abbreviations as in Table 1

|  | | A)NT pro-BNP | | B)NT pro-BNP | | C)NT pro-BNP | | D)NT pro-BNP | | E)NT pro-BNP | |
| --- | --- | --- | --- | --- | --- | --- | --- | --- | --- | --- | --- |
|  | | *Estimates* | *p* | *Estimates* | *p* | *Estimates* | *p* | *Estimates* | *p* | *Estimates* | *p* |
| Coffee consumption 3-4 cups/day | | -0.29 (-0.36 – -0.22) | **<0.001** | -0.08 (-0.15 – -0.01) | **0.021** | -0.07 (-0.15 – -0.00) | **0.042** | -0.07 (-0.14 – 0.00) | 0.064 | -0.11 (-0.24 – 0.02) | 0.089 |
| Coffee consumption >4 cups/day | | -0.34 (-0.43 – -0.25) | **<0.001** | -0.09 (-0.17 – 0.00) | 0.053 | -0.10 (-0.20 – -0.01) | **0.028** | -0.11 (-0.20 – -0.02) | **0.023** | -0.14 (-0.29 – 0.01) | 0.075 |
| Age | |  |  | 0.06 (0.05 – 0.06) | **<0.001** | 0.06 (0.05 – 0.06) | **<0.001** | 0.06 (0.05 – 0.06) | **<0.001** | 0.06 (0.05 – 0.06) | **<0.001** |
| Diabetes mellitus | |  |  | 0.02 (-0.08 – 0.12) | 0.677 | 0.01 (-0.10 – 0.11) | 0.897 | 0.00 (-0.10 – 0.11) | 0.926 | 0.19 (0.00 – 0.37) | **0.044** |
| Arterial hypertension | |  |  | 0.18 (0.11 – 0.25) | **<0.001** | 0.20 (0.13 – 0.27) | **<0.001** | 0.20 (0.13 – 0.28) | **<0.001** | 0.20 (0.07 – 0.34) | **0.003** |
| Current smoking | |  |  | 0.13 (0.06 – 0.20) | **<0.001** | 0.17 (0.10 – 0.25) | **<0.001** | 0.17 (0.09 – 0.25) | **<0.001** | 0.15 (0.01 – 0.29) | **0.040** |
| BMI | |  |  | -0.00 (-0.01 – 0.00) | 0.189 | -0.01 (-0.01 – 0.00) | 0.142 | -0.01 (-0.01 – 0.00) | 0.168 | -0.00 (-0.02 – 0.01) | 0.609 |
| Additives | **Milk** |  |  |  |  | 0.00 (-0.07 – 0.07) | 0.931 |  |  |  |  |
|  | **Milk + Sugar** |  |  |  |  | -0.15 (-0.25 – -0.04) | **0.007** |  |  |  |  |
|  | **Milk + Sweetener** |  |  |  |  | -0.09 (-0.27 – 0.08) | 0.297 |  |  |  |  |
|  | **Sugar** |  |  |  |  | -0.04 (-0.26 – 0.18) | 0.716 |  |  |  |  |
|  | **Sweetener** |  |  |  |  | -0.19 (-0.48 – 0.10) | 0.200 |  |  |  |  |
|  | **Binary** |  |  |  |  |  |  | -0.03 (-0.09 – 0.04) | 0.418 |  |  |

**Supplementary Table 110. Multivariable linear regression analysis for the association of mild/moderate/high coffee consumption and NT proBNP (only male).** “Not daily” coffee consumption (<1 cups/d) served as the reference group. Adjustment was performed for: A) unadjusted; B) main cardiovascular risk factors; C) additional adjustment for additives (separately), D) additional adjustment for additives (binary), E) additional adjustment for lipid lowering drugs, F) same adjustment as for group A, but only for subjects who consumed coffee without any additives. Abbreviations as in Table

|  | | A)SBP | | B)SBP | | C)SBP | | D)SBP | | E)SBP | |
| --- | --- | --- | --- | --- | --- | --- | --- | --- | --- | --- | --- |
|  | | *Estimates* | *p* | *Estimates* | *p* | *Estimates* | *p* | *Estimates* | *p* | *Estimates* | *p* |
| Coffee consumption 3-4 cups/day | | -4.52 (-5.98 – -3.05) | **<0.001** | -2.10 (-3.57 – -0.63) | **0.005** | -2.27 (-3.84 – -0.70) | **0.005** | -2.17 (-3.74 – -0.59) | **0.007** | -3.16 (-5.90 – -0.42) | **0.024** |
| Coffee consumption >4 cups/day | | -5.89 (-7.79 – -3.99) | **<0.001** | -3.37 (-5.28 – -1.45) | **0.001** | -3.82 (-5.87 – -1.76) | **<0.001** | -3.53 (-5.60 – -1.47) | **0.001** | -4.45 (-7.71 – -1.18) | **0.008** |
| Age | |  |  | 0.63 (0.55 – 0.70) | **<0.001** | 0.58 (0.50 – 0.67) | **<0.001** | 0.59 (0.51 – 0.67) | **<0.001** | 0.55 (0.39 – 0.70) | **<0.001** |
| Diabetes mellitus | |  |  | 4.12 (1.99 – 6.25) | **<0.001** | 4.11 (1.78 – 6.44) | **0.001** | 4.53 (2.21 – 6.85) | **<0.001** | 3.75 (-0.29 – 7.78) | 0.069 |
| Current smoking | |  |  | -0.65 (-2.26 – 0.96) | 0.431 | -0.35 (-2.08 – 1.39) | 0.694 | -0.63 (-2.37 – 1.10) | 0.475 | -0.69 (-3.72 – 2.34) | 0.655 |
| BMI | |  |  | 0.73 (0.57 – 0.88) | **<0.001** | 0.65 (0.48 – 0.81) | **<0.001** | 0.67 (0.50 – 0.83) | **<0.001** | 0.82 (0.54 – 1.10) | **<0.001** |
| Additives | **Milk** |  |  |  |  | -0.26 (-1.79 – 1.27) | 0.738 |  |  |  |  |
|  | **Milk + Sugar** |  |  |  |  | -3.63 (-5.97 – -1.28) | **0.002** |  |  |  |  |
|  | **Milk + Sweetener** |  |  |  |  | 2.34 (-1.46 – 6.14) | 0.228 |  |  |  |  |
|  | **Sugar** |  |  |  |  | -2.25 (-7.01 – 2.52) | 0.355 |  |  |  |  |
|  | **Sweetener** |  |  |  |  | 2.02 (-4.40 – 8.45) | 0.537 |  |  |  |  |
|  | **Binary** |  |  |  |  |  |  | -0.66 (-2.12 – 0.80) | 0.377 |  |  |

**Supplementary Table 111. Multivariable linear regression analysis for the association of moderate/high coffee consumption and systolic blood pressure (only male).** Not daily coffee consumption (<1 cups/d) served as the reference group. Adjustment was performed for: A) unadjusted; B) main cardiovascular risk factors; C) additional adjustment for additives (separately), D) additional adjustment for additives (binary), E) same adjustment as for group A, but only for subjects who consumed coffee without any additives. Abbreviations as in Table 1.

|  | | A)DBP | | B)DBP | | C)DBP | | D)DBP | | E)DBP | |
| --- | --- | --- | --- | --- | --- | --- | --- | --- | --- | --- | --- |
|  | | *Estimates* | *p* | *Estimates* | *p* | *Estimates* | *p* | *Estimates* | *p* | *Estimates* | *p* |
| Coffee consumption3 -4 cups/day | | -0.86 (-1.65 – -0.07) | **0.033** | -0.70 (-1.53 – 0.13) | 0.097 | -0.94 (-1.83 – -0.06) | **0.036** | -0.91 (-1.80 – -0.03) | **0.044** | -1.42 (-3.02 – 0.17) | 0.079 |
| Coffee consumption >4 cups/day | | -1.31 (-2.33 – -0.29) | **0.012** | -1.64 (-2.72 – -0.57) | **0.003** | -2.16 (-3.32 – -1.00) | **<0.001** | -2.10 (-3.27 – -0.94) | **<0.001** | -2.25 (-4.15 – -0.35) | **0.020** |
| Age | |  |  | -0.02 (-0.06 – 0.02) | 0.372 | -0.05 (-0.10 – -0.00) | **0.041** | -0.05 (-0.09 – -0.00) | **0.049** | -0.07 (-0.16 – 0.02) | 0.143 |
| Diabetes mellitus | |  |  | -0.10 (-1.29 – 1.10) | 0.873 | 0.14 (-1.18 – 1.46) | 0.836 | 0.42 (-0.90 – 1.73) | 0.533 | 0.28 (-2.07 – 2.62) | 0.818 |
| Current smoking | |  |  | -0.32 (-1.23 – 0.58) | 0.484 | -0.25 (-1.23 – 0.73) | 0.613 | -0.32 (-1.30 – 0.66) | 0.525 | -0.66 (-2.43 – 1.10) | 0.462 |
| BMI | |  |  | 0.47 (0.38 – 0.55) | **<0.001** | 0.41 (0.32 – 0.50) | **<0.001** | 0.42 (0.32 – 0.51) | **<0.001** | 0.44 (0.28 – 0.60) | **<0.001** |
| Additives | **Milk** |  |  |  |  | -0.38 (-1.24 – 0.48) | 0.389 |  |  |  |  |
|  | **Milk + Sugar** |  |  |  |  | -1.71 (-3.03 – -0.39) | **0.011** |  |  |  |  |
|  | **Milk + Sweetener** |  |  |  |  | 1.59 (-0.55 – 3.74) | 0.146 |  |  |  |  |
|  | **Sugar** |  |  |  |  | -0.11 (-2.80 – 2.58) | 0.937 |  |  |  |  |
|  | **Sweetener** |  |  |  |  | -0.59 (-4.22 – 3.04) | 0.750 |  |  |  |  |
|  | **Binary** |  |  |  |  |  |  | -0.47 (-1.29 – 0.36) | 0.267 |  |  |

**Supplementary Table 112. Multivariable linear regression analysis for the association of moderate/high coffee consumption and diastolic blood pressure (only male).** Not daily coffee consumption (<1 cups/d) served as the reference group. Adjustment was performed for: A) unadjusted; B) main cardiovascular risk factors; C) additional adjustment for additives (separately), D) additional adjustment for additives (binary), E) same adjustment as for group A, but only for subjects who consumed coffee without any additives. Abbreviations as in Table 1.

|  | | A)Heart rate | | B)Heart rate | | C)Heart rate | | D)Heart rate | | E)Heart rate | |
| --- | --- | --- | --- | --- | --- | --- | --- | --- | --- | --- | --- |
|  | | *Estimates* | *p* | *Estimates* | *p* | *Estimates* | *p* | *Estimates* | *p* | *Estimates* | *p* |
| Coffee consumption 3-4 cups/day | | -0.60 (-1.43 – 0.23) | 0.154 | -0.29 (-1.16 – 0.58) | 0.511 | -0.38 (-1.32 – 0.56) | 0.430 | -0.40 (-1.34 – 0.55) | 0.409 | 0.24 (-1.45 – 1.93) | 0.781 |
| Coffee consumption >4 cups/day | | 0.41 (-0.65 – 1.48) | 0.445 | 0.25 (-0.88 – 1.38) | 0.668 | 0.26 (-0.98 – 1.49) | 0.682 | 0.20 (-1.04 – 1.44) | 0.750 | 1.72 (-0.31 – 3.75) | 0.097 |
| Age | |  |  | 0.00 (-0.05 – 0.05) | 0.966 | -0.01 (-0.06 – 0.05) | 0.846 | -0.00 (-0.06 – 0.05) | 0.876 | -0.04 (-0.13 – 0.06) | 0.472 |
| Diabetes mellitus | |  |  | 3.91 (2.66 – 5.15) | **<0.001** | 4.19 (2.81 – 5.58) | **<0.001** | 4.21 (2.83 – 5.59) | **<0.001** | 3.35 (0.89 – 5.80) | **0.008** |
| Arterial hypertension | |  |  | 2.42 (1.55 – 3.30) | **<0.001** | 2.61 (1.65 – 3.56) | **<0.001** | 2.60 (1.64 – 3.56) | **<0.001** | 3.27 (1.50 – 5.04) | **<0.001** |
| Current smoking | |  |  | 2.01 (1.06 – 2.96) | **<0.001** | 1.82 (0.77 – 2.86) | **0.001** | 1.85 (0.81 – 2.89) | **0.001** | 3.17 (1.30 – 5.05) | **0.001** |
| BMI | |  |  | 0.36 (0.26 – 0.45) | **<0.001** | 0.35 (0.25 – 0.46) | **<0.001** | 0.35 (0.25 – 0.45) | **<0.001** | 0.38 (0.20 – 0.56) | **<0.001** |
| Additives | **Milk** |  |  |  |  | 0.40 (-0.52 – 1.32) | 0.393 |  |  |  |  |
|  | **Milk + Sugar** |  |  |  |  | 0.56 (-0.85 – 1.97) | 0.437 |  |  |  |  |
|  | **Milk + Sweetener** |  |  |  |  | 1.79 (-0.51 – 4.08) | 0.127 |  |  |  |  |
|  | **Sugar** |  |  |  |  | 1.61 (-1.26 – 4.48) | 0.271 |  |  |  |  |
|  | **Sweetener** |  |  |  |  | -1.06 (-4.92 – 2.80) | 0.590 |  |  |  |  |
|  | **Binary** |  |  |  |  |  |  | 0.50 (-0.38 – 1.37) | 0.264 |  |  |

**Supplementary Table 113. Multivariable linear regression analysis for the association of mild/moderate/high coffee consumption and heart rate (only male).** Not daily coffee consumption (<1 cups/d) served as the reference group. Adjustment was performed for: A) unadjusted; B) main cardiovascular risk factors; C) additional adjustment for additives (separately), D) additional adjustment for additives (binary), E) same adjustment as for group A, but only for subjects who consumed coffee without any additives. Abbreviations as in Table 1.

|  | | A)PQ interval | | B)PQ interval | | C)PQ interval | | D)PQ interval | | E)PQ interval | |
| --- | --- | --- | --- | --- | --- | --- | --- | --- | --- | --- | --- |
|  | | *Estimates* | *p* | *Estimates* | *p* | *Estimates* | *p* | *Estimates* | *p* | *Estimates* | *p* |
| Coffee consumption 3-4 cups/day | | -3.21 (-5.26 – -1.16) | **0.002** | -0.16 (-2.36 – 2.04) | 0.885 | -0.05 (-2.42 – 2.32) | 0.967 | 0.02 (-2.36 – 2.40) | 0.987 | 0.07 (-4.41 – 4.54) | 0.977 |
| Coffee consumption >4 cups/day | | -3.84 (-6.45 – -1.23) | **0.004** | -0.88 (-3.72 – 1.96) | 0.544 | -1.31 (-4.39 – 1.77) | 0.404 | -1.17 (-4.26 – 1.93) | 0.460 | 0.83 (-4.53 – 6.18) | 0.762 |
| Age | |  |  | 0.69 (0.57 – 0.81) | **<0.001** | 0.70 (0.57 – 0.83) | **<0.001** | 0.71 (0.58 – 0.84) | **<0.001** | 0.81 (0.55 – 1.07) | **<0.001** |
| Diabetes mellitus | |  |  | 3.17 (-0.02 – 6.36) | 0.052 | 2.73 (-0.81 – 6.27) | 0.130 | 2.77 (-0.77 – 6.30) | 0.125 | 8.70 (2.09 – 15.32) | **0.010** |
| Arterial hypertension | |  |  | 0.21 (-1.99 – 2.41) | 0.851 | 0.41 (-1.98 – 2.80) | 0.738 | 0.43 (-1.97 – 2.83) | 0.726 | -0.96 (-5.58 – 3.67) | 0.685 |
| Current smoking | |  |  | -2.69 (-5.08 – -0.29) | **0.028** | -2.60 (-5.21 – 0.01) | 0.051 | -2.82 (-5.43 – -0.21) | **0.034** | -4.34 (-9.23 – 0.55) | 0.082 |
| BMI | |  |  | 0.35 (0.11 – 0.59) | **0.004** | 0.31 (0.05 – 0.57) | **0.018** | 0.31 (0.05 – 0.57) | **0.018** | 0.03 (-0.44 – 0.49) | 0.915 |
| Additives | **Milk** |  |  |  |  | 1.40 (-0.92 – 3.72) | 0.238 |  |  |  |  |
|  | **Milk + Sugar** |  |  |  |  | -1.81 (-5.36 – 1.75) | 0.320 |  |  |  |  |
|  | **Milk + Sweetener** |  |  |  |  | -2.10 (-7.94 – 3.74) | 0.481 |  |  |  |  |
|  | **Sugar** |  |  |  |  | -1.36 (-8.53 – 5.80) | 0.709 |  |  |  |  |
|  | **Sweetener** |  |  |  |  | -0.03 (-9.46 – 9.40) | 0.995 |  |  |  |  |
|  | **Binary** |  |  |  |  |  |  | 0.71 (-1.51 – 2.92) | 0.533 |  |  |

**Supplementary Table 114. Multivariable linear regression analysis for the association of mild/moderate/high coffee consumption and PQ interval (only female).** Not daily coffee consumption (<1 cups/d) served as the reference group. Adjustment was performed for: A) unadjusted; B) main cardiovascular risk factors; C) additional adjustment for additives (separately), D) additional adjustment for additives (binary), E) same adjustment as for group A, but only for subjects who consumed coffee without any additives. Abbreviations as in Table 1.

|  | | A)QRS interval | | B)QRS interval | | C)QRS interval | | D)QRS interval | | E)QRS interval | |
| --- | --- | --- | --- | --- | --- | --- | --- | --- | --- | --- | --- |
|  | | *Estimates* | *p* | *Estimates* | *p* | *Estimates* | *p* | *Estimates* | *p* | *Estimates* | *p* |
| Coffee consumption 3-4 cups/day | | -0.10 (-1.23 – 1.04) | 0.869 | 0.77 (-0.47 – 2.00) | 0.225 | 0.85 (-0.45 – 2.16) | 0.199 | 0.87 (-0.44 – 2.17) | 0.193 | 1.81 (-0.59 – 4.21) | 0.139 |
| Coffee consumption >4 cups/day | | -1.30 (-2.75 – 0.14) | 0.078 | -0.54 (-2.15 – 1.06) | 0.506 | -0.26 (-1.96 – 1.44) | 0.767 | -0.27 (-1.98 – 1.43) | 0.752 | 0.59 (-2.30 – 3.49) | 0.688 |
| Age | |  |  | 0.17 (0.10 – 0.23) | **<0.001** | 0.18 (0.10 – 0.25) | **<0.001** | 0.18 (0.11 – 0.25) | **<0.001** | 0.13 (-0.00 – 0.27) | 0.058 |
| Diabetes mellitus | |  |  | -0.94 (-2.70 – 0.83) | 0.299 | -1.51 (-3.43 – 0.40) | 0.121 | -1.60 (-3.51 – 0.30) | 0.099 | -1.39 (-4.84 – 2.06) | 0.430 |
| Arterial hypertension | |  |  | 1.36 (0.11 – 2.60) | **0.033** | 1.27 (-0.05 – 2.59) | 0.060 | 1.23 (-0.09 – 2.56) | 0.069 | 0.99 (-1.52 – 3.51) | 0.439 |
| Current smoking | |  |  | -1.18 (-2.54 – 0.17) | 0.086 | -0.84 (-2.27 – 0.60) | 0.254 | -0.88 (-2.32 – 0.55) | 0.227 | -1.87 (-4.52 – 0.77) | 0.164 |
| BMI | |  |  | 0.22 (0.09 – 0.36) | **0.001** | 0.18 (0.04 – 0.32) | **0.014** | 0.18 (0.03 – 0.32) | **0.014** | 0.30 (0.05 – 0.55) | **0.018** |
| Additives | **Milk** |  |  |  |  | 0.47 (-0.80 – 1.75) | 0.465 |  |  |  |  |
|  | **Milk + Sugar** |  |  |  |  | -0.31 (-2.28 – 1.65) | 0.754 |  |  |  |  |
|  | **Milk + Sweetener** |  |  |  |  | -1.39 (-4.58 – 1.80) | 0.392 |  |  |  |  |
|  | **Sugar** |  |  |  |  | 0.15 (-3.81 – 4.10) | 0.941 |  |  |  |  |
|  | **Sweetener** |  |  |  |  | 2.68 (-2.43 – 7.80) | 0.304 |  |  |  |  |
|  | **Binary** |  |  |  |  |  |  | 0.27 (-0.94 – 1.49) | 0.659 |  |  |

**Supplementary Table 115. Multivariable linear regression analysis for the association of mild/moderate/high coffee consumption and QRS interval (only male).** Not daily coffee consumption (<1 cups/d) served as the reference group. Adjustment was performed for: A) unadjusted; B) main cardiovascular risk factors; C) additional adjustment for additives (separately), D) additional adjustment for additives (binary), E) same adjustment as for group A, but only for subjects who consumed coffee without any additives. Abbreviations as in Table

|  | | A)QTc interval | | B)QTc interval | | C)QTc interval | | D)QTc interval | | E)QTc interval | |
| --- | --- | --- | --- | --- | --- | --- | --- | --- | --- | --- | --- |
|  | | *Estimates* | *p* | *Estimates* | *p* | *Estimates* | *p* | *Estimates* | *p* | *Estimates* | *p* |
| Coffee consumption 3-4 cups/day | | -4.18 (-9.06 – 0.70) | 0.093 | -2.72 (-8.22 – 2.79) | 0.333 | -3.38 (-9.67 – 2.91) | 0.292 | -3.40 (-9.71 – 2.91) | 0.291 | -1.36 (-5.39 – 2.67) | 0.508 |
| Coffee consumption >4 cups/day | | -6.63 (-12.91 – -0.35) | **0.038** | -5.49 (-12.65 – 1.68) | 0.133 | -5.51 (-13.74 – 2.71) | 0.189 | -5.77 (-14.04 – 2.51) | 0.172 | 2.00 (-2.89 – 6.90) | 0.422 |
| Age | |  |  | 0.39 (0.10 – 0.69) | **0.009** | 0.38 (0.04 – 0.72) | **0.030** | 0.38 (0.03 – 0.72) | **0.032** | 0.45 (0.22 – 0.69) | **<0.001** |
| Diabetes mellitus | |  |  | 13.11 (5.16 – 21.05) | **0.001** | 15.55 (6.15 – 24.95) | **0.001** | 15.04 (5.67 – 24.41) | **0.002** | 9.23 (3.29 – 15.17) | **0.002** |
| Arterial hypertension | |  |  | 2.10 (-3.41 – 7.60) | 0.456 | 1.97 (-4.35 – 8.28) | 0.541 | 1.94 (-4.40 – 8.28) | 0.549 | 3.26 (-0.94 – 7.46) | 0.128 |
| Current smoking | |  |  | 0.19 (-5.81 – 6.19) | 0.950 | 0.20 (-6.71 – 7.10) | 0.956 | 0.04 (-6.88 – 6.95) | 0.992 | 2.44 (-1.95 – 6.83) | 0.276 |
| BMI | |  |  | 1.49 (0.90 – 2.09) | **<0.001** | 1.62 (0.94 – 2.30) | **<0.001** | 1.59 (0.91 – 2.28) | **<0.001** | 0.47 (0.04 – 0.90) | **0.033** |
| Additives | **Milk** |  |  |  |  | 3.95 (-2.19 – 10.10) | 0.207 |  |  |  |  |
|  | **Milk + Sugar** |  |  |  |  | 3.42 (-5.96 – 12.80) | 0.475 |  |  |  |  |
|  | **Milk + Sweetener** |  |  |  |  | -3.60 (-18.89 – 11.68) | 0.644 |  |  |  |  |
|  | **Sugar** |  |  |  |  | 4.55 (-14.44 – 23.53) | 0.639 |  |  |  |  |
|  | **Sweetener** |  |  |  |  | -7.78 (-32.57 – 17.01) | 0.538 |  |  |  |  |
|  | **Binary** |  |  |  |  |  |  | 3.36 (-2.52 – 9.23) | 0.262 |  |  |

**Supplementary Table 116. Multivariable linear regression analysis for the association of mild/moderate/high coffee consumption and QTc interval (only male).** Not daily coffee consumption (<1 cups/d) served as the reference group. Adjustment was performed for: A) unadjusted; B) main cardiovascular risk factors; C) additional adjustment for additives (separately), D) additional adjustment for additives (binary), E) same adjustment as for group A, but only for subjects who consumed coffee without any additives. Abbreviations as in Table 1.

|  | | A)LVEF | | B)LVEF | | C)LVEF | | D)LVEF | | E)LVEF | |
| --- | --- | --- | --- | --- | --- | --- | --- | --- | --- | --- | --- |
|  | | *Estimates* | *p* | *Estimates* | *p* | *Estimates* | *p* | *Estimates* | *p* | *Estimates* | *p* |
| Coffee consumption 3-4 cups/day | | -0.03 (-0.46 – 0.40) | 0.891 | -0.24 (-0.71 – 0.22) | 0.306 | -0.21 (-0.71 – 0.28) | 0.395 | -0.24 (-0.73 – 0.25) | 0.343 | 0.12 (-0.72 – 0.96) | 0.778 |
| Coffee consumption >4 cups/day | | 0.15 (-0.39 – 0.70) | 0.583 | 0.03 (-0.57 – 0.63) | 0.915 | -0.07 (-0.72 – 0.58) | 0.832 | -0.11 (-0.76 – 0.54) | 0.742 | -0.36 (-1.36 – 0.65) | 0.488 |
| Age | |  |  | -0.03 (-0.06 – -0.01) | **0.014** | -0.03 (-0.06 – -0.01) | **0.020** | -0.03 (-0.06 – -0.01) | **0.018** | -0.01 (-0.06 – 0.04) | 0.595 |
| Diabetes mellitus | |  |  | -0.98 (-1.65 – -0.31) | **0.004** | -0.95 (-1.69 – -0.22) | **0.011** | -0.89 (-1.62 – -0.16) | **0.017** | -0.90 (-2.13 – 0.34) | 0.154 |
| Arterial hypertension | |  |  | -0.14 (-0.60 – 0.33) | 0.569 | -0.05 (-0.55 – 0.45) | 0.852 | -0.02 (-0.52 – 0.48) | 0.937 | -0.10 (-0.95 – 0.75) | 0.826 |
| Current smoking | |  |  | -0.40 (-0.91 – 0.12) | 0.132 | -0.47 (-1.03 – 0.08) | 0.094 | -0.45 (-1.00 – 0.10) | 0.111 | -0.49 (-1.43 – 0.45) | 0.309 |
| BMI | |  |  | -0.14 (-0.20 – -0.09) | **<0.001** | -0.15 (-0.21 – -0.09) | **<0.001** | -0.15 (-0.20 – -0.09) | **<0.001** | -0.18 (-0.27 – -0.09) | **<0.001** |
| Additives | **Milk** |  |  |  |  | -0.20 (-0.68 – 0.28) | 0.424 |  |  |  |  |
|  | **Milk + Sugar** |  |  |  |  | -0.07 (-0.82 – 0.69) | 0.865 |  |  |  |  |
|  | **Milk + Sweetener** |  |  |  |  | 0.97 (-0.23 – 2.16) | 0.114 |  |  |  |  |
|  | **Sugar** |  |  |  |  | -0.30 (-1.82 – 1.22) | 0.698 |  |  |  |  |
|  | **Sweetener** |  |  |  |  | -0.28 (-2.36 – 1.79) | 0.789 |  |  |  |  |
|  | **Binary** |  |  |  |  |  |  | -0.12 (-0.58 – 0.34) | 0.603 |  |  |

**Supplementary Table 117. Multivariable linear regression analysis for the association of mild/moderate/high coffee consumption and LVEF (only male).** Not daily coffee consumption (<1 cups/d) served as the reference group. Adjustment was performed for: A) unadjusted; B) main cardiovascular risk factors; C) additional adjustment for additives (separately), D) additional adjustment for additives (binary), E) same adjustment as for group A, but only for subjects who consumed coffee without any additives. Abbreviations as in Table 1.

|  | | A)LV mass index | | B)LV mass index | | C)LV mass index | | D)LV mass index | | E)LV mass index | |
| --- | --- | --- | --- | --- | --- | --- | --- | --- | --- | --- | --- |
|  | | *Estimates* | *p* | *Estimates* | *p* | *Estimates* | *p* | *Estimates* | *p* | *Estimates* | *p* |
| Coffee consumption 3-4 cups/day | | -2.17 (-3.99 – -0.36) | **0.019** | -1.29 (-3.16 – 0.57) | 0.174 | -1.41 (-3.41 – 0.59) | 0.167 | -1.20 (-3.20 – 0.80) | 0.239 | -2.86 (-6.48 – 0.76) | 0.121 |
| Coffee consumption >4 cups/day | | -1.09 (-3.37 – 1.19) | 0.349 | -0.28 (-2.66 – 2.10) | 0.816 | -1.24 (-3.83 – 1.35) | 0.347 | -0.99 (-3.59 – 1.61) | 0.454 | -0.19 (-4.42 – 4.04) | 0.931 |
| Age | |  |  | 0.27 (0.17 – 0.37) | **<0.001** | 0.26 (0.15 – 0.37) | **<0.001** | 0.27 (0.16 – 0.38) | **<0.001** | 0.16 (-0.04 – 0.37) | 0.121 |
| Diabetes mellitus | |  |  | -0.34 (-3.01 – 2.33) | 0.802 | -1.33 (-4.30 – 1.65) | 0.382 | -1.33 (-4.29 – 1.63) | 0.378 | -1.22 (-6.75 – 4.31) | 0.665 |
| Arterial hypertension | |  |  | 4.81 (2.94 – 6.67) | **<0.001** | 5.10 (3.08 – 7.12) | **<0.001** | 5.14 (3.11 – 7.16) | **<0.001** | 3.11 (-0.54 – 6.76) | 0.094 |
| Current smoking | |  |  | 2.07 (-0.00 – 4.13) | 0.050 | 2.94 (0.69 – 5.18) | **0.010** | 2.76 (0.52 – 5.01) | **0.016** | 3.44 (-0.60 – 7.48) | 0.095 |
| BMI | |  |  | 1.22 (1.01 – 1.42) | **<0.001** | 1.17 (0.95 – 1.39) | **<0.001** | 1.17 (0.95 – 1.39) | **<0.001** | 1.50 (1.11 – 1.89) | **<0.001** |
| Additives | **Milk** |  |  |  |  | -1.49 (-3.44 – 0.45) | 0.132 |  |  |  |  |
|  | **Milk + Sugar** |  |  |  |  | -4.74 (-7.77 – -1.70) | **0.002** |  |  |  |  |
|  | **Milk + Sweetener** |  |  |  |  | -3.48 (-8.37 – 1.40) | 0.162 |  |  |  |  |
|  | **Sugar** |  |  |  |  | -3.72 (-9.84 – 2.41) | 0.234 |  |  |  |  |
|  | **Sweetener** |  |  |  |  | -1.83 (-9.96 – 6.30) | 0.659 |  |  |  |  |
|  | **Binary** |  |  |  |  |  |  | -2.16 (-4.02 – -0.30) | **0.023** |  |  |

**Supplementary Table 118. Multivariable linear regression analysis for the association of mild/moderate/high coffee consumption and LV mass index (only male).** Not daily coffee consumption (<1 cups/d) served as the reference group. Adjustment was performed for: A) unadjusted; B) main cardiovascular risk factors; C) additional adjustment for additives (separately), D) additional adjustment for additives (binary), E) same adjustment as for group A, but only for subjects who consumed coffee without any additives. Abbreviations as in Table 1.

|  | | A)E/e‘ ratio | | B)E/e‘ ratio | | C)E/e‘ ratio | | D)E/e‘ ratio | | E)E/e‘ ratio | |
| --- | --- | --- | --- | --- | --- | --- | --- | --- | --- | --- | --- |
|  | | *Estimates* | *p* | *Estimates* | *p* | *Estimates* | *p* | *Estimates* | *p* | *Estimates* | *p* |
| Coffee consumption 3-4 cups/day | | -0.31 (-0.48 – -0.14) | **<0.001** | -0.09 (-0.27 – 0.08) | 0.292 | -0.13 (-0.32 – 0.06) | 0.169 | -0.13 (-0.32 – 0.06) | 0.187 | -0.38 (-0.74 – -0.02) | **0.038** |
| Coffee consumption >4 cups/day | | -0.34 (-0.55 – -0.12) | **0.002** | -0.12 (-0.34 – 0.11) | 0.308 | -0.20 (-0.45 – 0.04) | 0.102 | -0.19 (-0.44 – 0.05) | 0.123 | -0.14 (-0.58 – 0.29) | 0.518 |
| Age | |  |  | 0.06 (0.05 – 0.07) | **<0.001** | 0.06 (0.05 – 0.07) | **<0.001** | 0.06 (0.05 – 0.07) | **<0.001** | 0.06 (0.04 – 0.08) | **<0.001** |
| Diabetes mellitus | |  |  | 0.67 (0.42 – 0.92) | **<0.001** | 0.71 (0.43 – 0.99) | **<0.001** | 0.72 (0.45 – 1.00) | **<0.001** | 1.19 (0.67 – 1.72) | **<0.001** |
| Arterial hypertension | |  |  | 0.53 (0.36 – 0.71) | **<0.001** | 0.51 (0.32 – 0.70) | **<0.001** | 0.51 (0.32 – 0.70) | **<0.001** | 0.36 (-0.01 – 0.73) | 0.053 |
| Current smoking [Yes] | |  |  | 0.29 (0.10 – 0.48) | **0.003** | 0.28 (0.06 – 0.49) | **0.011** | 0.27 (0.05 – 0.48) | **0.014** | 0.06 (-0.34 – 0.47) | 0.755 |
| BMI | |  |  | 0.08 (0.06 – 0.10) | **<0.001** | 0.08 (0.06 – 0.10) | **<0.001** | 0.08 (0.06 – 0.10) | **<0.001** | 0.09 (0.05 – 0.13) | **<0.001** |
| Additives | **Milk** |  |  |  |  | -0.05 (-0.23 – 0.14) | 0.629 |  |  |  |  |
|  | **Milk + Sugar** |  |  |  |  | -0.15 (-0.44 – 0.13) | 0.294 |  |  |  |  |
|  | **Milk + Sweetener** |  |  |  |  | 0.11 (-0.35 – 0.57) | 0.647 |  |  |  |  |
|  | **Sugar** |  |  |  |  | -0.25 (-0.83 – 0.34) | 0.408 |  |  |  |  |
|  | **Sweetener** |  |  |  |  | -0.25 (-1.04 – 0.54) | 0.537 |  |  |  |  |
|  | **Binary** |  |  |  |  |  |  | -0.06 (-0.24 – 0.11) | 0.483 |  |  |

**Supplementary Table 119. Multivariable linear regression analysis for the association of mild/moderate/high coffee consumption and E/e’ ratio (only male).** Not daily coffee consumption (<1 cups/d) served as the reference group. Adjustment was performed for: A) unadjusted; B) main cardiovascular risk factors; C) additional adjustment for additives (separately), D) additional adjustment for additives (binary), E) same adjustment as for group A, but only for subjects who consumed coffee without any additives. Abbreviations as in Table 1.

|  | | A)TR Vmax in m/s | | B)TR Vmax in m/s | | C)TR Vmax in m/s | | D)TR Vmax in m/s | | E)TR Vmax in m/s | |
| --- | --- | --- | --- | --- | --- | --- | --- | --- | --- | --- | --- |
|  | | *Estimates* | *p* | *Estimates* | *p* | *Estimates* | *p* | *Estimates* | *p* | *Estimates* | *p* |
| Coffee consumption 3-4 cups/day | | -0.03 (-0.06 – 0.01) | 0.171 | -0.01 (-0.05 – 0.02) | 0.484 | -0.01 (-0.05 – 0.03) | 0.644 | -0.01 (-0.05 – 0.03) | 0.712 | -0.06 (-0.14 – 0.03) | 0.182 |
| Coffee consumption >4 cups/day | | -0.03 (-0.08 – 0.02) | 0.195 | 0.00 (-0.05 – 0.06) | 0.869 | -0.01 (-0.07 – 0.05) | 0.780 | -0.01 (-0.07 – 0.05) | 0.801 | -0.02 (-0.13 – 0.08) | 0.649 |
| Age | |  |  | 0.01 (0.00 – 0.01) | **<0.001** | 0.01 (0.00 – 0.01) | **<0.001** | 0.01 (0.00 – 0.01) | **<0.001** | 0.01 (0.00 – 0.01) | **0.003** |
| Diabetes mellitus | |  |  | 0.03 (-0.03 – 0.10) | 0.304 | 0.01 (-0.06 – 0.08) | 0.688 | 0.02 (-0.05 – 0.09) | 0.630 | -0.04 (-0.17 – 0.09) | 0.565 |
| Arterial hypertension | |  |  | 0.03 (-0.01 – 0.07) | 0.144 | 0.03 (-0.02 – 0.07) | 0.243 | 0.03 (-0.01 – 0.07) | 0.192 | -0.03 (-0.12 – 0.06) | 0.454 |
| Current smoking | |  |  | -0.04 (-0.08 – 0.01) | 0.133 | -0.03 (-0.08 – 0.02) | 0.226 | -0.04 (-0.09 – 0.01) | 0.134 | 0.01 (-0.10 – 0.12) | 0.919 |
| BMI | |  |  | 0.00 (-0.00 – 0.01) | 0.894 | 0.00 (-0.00 – 0.01) | 0.502 | 0.00 (-0.00 – 0.01) | 0.536 | 0.01 (-0.00 – 0.02) | 0.112 |
| Additives | **Milk** |  |  |  |  | -0.03 (-0.07 – 0.02) | 0.236 |  |  |  |  |
|  | **Milk + Sugar** |  |  |  |  | -0.04 (-0.11 – 0.03) | 0.273 |  |  |  |  |
|  | **Milk + Sweetener** |  |  |  |  | 0.03 (-0.07 – 0.13) | 0.590 |  |  |  |  |
|  | **Sugar** |  |  |  |  | 0.06 (-0.08 – 0.20) | 0.408 |  |  |  |  |
|  | **Sweetener** |  |  |  |  | 0.09 (-0.11 – 0.29) | 0.379 |  |  |  |  |
|  | **Binary** |  |  |  |  |  |  | -0.02 (-0.06 – 0.02) | 0.294 |  |  |

**Supplementary Table 120. Multivariable linear regression analysis for the association of mild/moderate/high coffee consumption and TR Vmax in m/s (only male).** Not daily coffee consumption (<1 cups/d) served as the reference group. Adjustment was performed for: A) unadjusted; B) main cardiovascular risk factors; C) additional adjustment for additives (separately), D) additional adjustment for additives (binary), E) same adjustment as for group A, but only for subjects who consumed coffee without any additives. Abbreviations as in Table 1.

|  | | A)TAPSE in mm | | B)TAPSE in mm | | C)TAPSE in mm | | D)TAPSE in mm | | E)TAPSE in mm | |
| --- | --- | --- | --- | --- | --- | --- | --- | --- | --- | --- | --- |
|  | | *Estimates* | *p* | *Estimates* | *p* | *Estimates* | *p* | *Estimates* | *p* | *Estimates* | *p* |
| Coffee consumption3 -4 cups/day | | 0.43 (0.03 – 0.83) | **0.036** | 0.25 (-0.19 – 0.68) | 0.267 | 0.33 (-0.13 – 0.79) | 0.161 | 0.34 (-0.12 – 0.81) | 0.144 | 0.37 (-0.45 – 1.18) | 0.376 |
| Coffee consumption >4 cups/day | | 0.54 (0.05 – 1.04) | **0.032** | 0.37 (-0.18 – 0.92) | 0.186 | 0.34 (-0.25 – 0.93) | 0.255 | 0.35 (-0.24 – 0.94) | 0.244 | 0.22 (-0.72 – 1.17) | 0.642 |
| Age | |  |  | -0.04 (-0.06 – -0.01) | **0.003** | -0.04 (-0.06 – -0.01) | **0.005** | -0.04 (-0.06 – -0.01) | **0.005** | -0.01 (-0.06 – 0.03) | 0.561 |
| Diabetes mellitus | |  |  | -1.21 (-1.85 – -0.57) | **<0.001** | -1.08 (-1.80 – -0.36) | **0.003** | -1.17 (-1.89 – -0.46) | **0.001** | -1.57 (-2.82 – -0.31) | **0.015** |
| Arterial hypertension | |  |  | -0.38 (-0.81 – 0.06) | 0.092 | -0.41 (-0.88 – 0.06) | 0.085 | -0.43 (-0.90 – 0.04) | 0.074 | -0.76 (-1.58 – 0.06) | 0.070 |
| Current smoking | |  |  | -0.58 (-1.07 – -0.10) | **0.019** | -0.49 (-1.01 – 0.03) | 0.063 | -0.50 (-1.02 – 0.02) | 0.060 | -0.84 (-1.75 – 0.07) | 0.070 |
| BMI | |  |  | 0.01 (-0.04 – 0.06) | 0.612 | 0.04 (-0.02 – 0.09) | 0.169 | 0.04 (-0.02 – 0.09) | 0.177 | 0.08 (-0.02 – 0.17) | 0.116 |
| Additives | **Milk** |  |  |  |  | -0.02 (-0.47 – 0.44) | 0.940 |  |  |  |  |
|  | **Milk + Sugar** |  |  |  |  | 0.12 (-0.58 – 0.83) | 0.731 |  |  |  |  |
|  | **Milk + Sweetener** |  |  |  |  | -0.56 (-1.65 – 0.53) | 0.315 |  |  |  |  |
|  | **Sugar** |  |  |  |  | 0.50 (-0.89 – 1.89) | 0.480 |  |  |  |  |
|  | **Sweetener** |  |  |  |  | 1.58 (-0.56 – 3.72) | 0.148 |  |  |  |  |
|  | **Binary** |  |  |  |  |  |  | -0.01 (-0.44 – 0.43) | 0.982 |  |  |

**Supplementary Table 121. Multivariable linear regression analysis for the association of mild/moderate/high coffee consumption and TAPSE in mm (only male).** Not daily coffee consumption (<1 cups/d) served as the reference group. Adjustment was performed for: A) unadjusted; B) main cardiovascular risk factors; C) additional adjustment for additives (separately), D) additional adjustment for additives (binary), E) same adjustment as for group A, but only for subjects who consumed coffee without any additives. Abbreviations as in Table 1.

|  |  | A)LASV in ml | | B)LASV in ml | | C)LASV in ml | | D)LASV in ml | | E)LASV in ml | |
| --- | --- | --- | --- | --- | --- | --- | --- | --- | --- | --- | --- |
|  |  | *Estimates* | *p* | *Estimates* | *p* | *Estimates* | *p* | *Estimates* | *p* | *Estimates* | *p* |
| Coffee consumption 3-4 cups/day |  | -0.60 (-1.43 – 0.23) | 0.155 | -0.02 (-0.89 – 0.84) | 0.956 | -0.06 (-0.99 – 0.87) | 0.900 | 0.04 (-0.89 – 0.97) | 0.933 | -0.25 (-1.96 – 1.46) | 0.771 |
| Coffee consumption >4 cups/day |  | -0.99 (-2.05 – 0.06) | 0.065 | -0.13 (-1.24 – 0.98) | 0.824 | -0.22 (-1.43 – 0.98) | 0.717 | -0.21 (-1.42 – 1.00) | 0.734 | -0.68 (-2.66 – 1.31) | 0.504 |
| Age |  |  |  | 0.16 (0.12 – 0.21) | **<0.001** | 0.17 (0.12 – 0.22) | **<0.001** | 0.17 (0.12 – 0.22) | **<0.001** | 0.16 (0.06 – 0.26) | **0.002** |
| Diabetes mellitus |  |  |  | -0.23 (-1.46 – 1.01) | 0.716 | -0.44 (-1.82 – 0.93) | 0.526 | -0.55 (-1.91 – 0.82) | 0.431 | 1.27 (-1.21 – 3.75) | 0.315 |
| Arterial hypertension |  |  |  | 0.81 (-0.06 – 1.68) | 0.068 | 0.66 (-0.29 – 1.61) | 0.175 | 0.69 (-0.26 – 1.64) | 0.154 | 0.10 (-1.66 – 1.85) | 0.914 |
| Current smoking |  |  |  | -1.56 (-2.52 – -0.60) | **0.001** | -1.26 (-2.30 – -0.21) | **0.018** | -1.39 (-2.44 – -0.35) | **0.009** | -0.65 (-2.51 – 1.21) | 0.492 |
| BMI |  |  |  | 0.34 (0.24 – 0.44) | **<0.001** | 0.35 (0.24 – 0.46) | **<0.001** | 0.35 (0.24 – 0.46) | **<0.001** | 0.38 (0.19 – 0.57) | **<0.001** |
| Additives | **Milk** |  |  |  |  | 0.05 (-0.86 – 0.96) | 0.920 |  |  |  |  |
|  | **Milk + Sugar** |  |  |  |  | -1.33 (-2.77 – 0.11) | 0.070 |  |  |  |  |
|  | **Milk + Sweetener** |  |  |  |  | -3.09 (-5.36 – -0.81) | **0.008** |  |  |  |  |
|  | **Sugar** |  |  |  |  | -1.23 (-4.03 – 1.57) | 0.389 |  |  |  |  |
|  | **Sweetener** |  |  |  |  | 3.69 (-0.39 – 7.78) | 0.077 |  |  |  |  |
|  | **Binary** |  |  |  |  |  |  | -0.34 (-1.22 – 0.53) | 0.439 |  |  |

**Supplementary Table 122. Multivariable linear regression analysis for the association of mild/moderate/high coffee consumption and LASV in ml (only male).** Not daily coffee consumption (<1 cups/d) served as the reference group. Adjustment was performed for: A) unadjusted; B) main cardiovascular risk factors; C) additional adjustment for additives (separately), D) additional adjustment for additives (binary), E) same adjustment as for group A, but only for subjects who consumed coffee without any additives. Abbreviations as in Table 1.

|  | | A)LBB | | B)LBB | | C)LBB | | D)LBB | | E)LBB | |
| --- | --- | --- | --- | --- | --- | --- | --- | --- | --- | --- | --- |
|  | | *Odds Ratios* | *p* | *Odds Ratios* | *p* | *Odds Ratios* | *p* | *Odds Ratios* | *p* | *Odds Ratios* | *p* |
| Coffee consumption 3-4 cups/day | | 0.74 (0.54 – 0.99) | **0.048** | 0.87 (0.62 – 1.19) | 0.386 | 0.91 (0.62 – 1.31) | 0.622 | 0.84 (0.59 – 1.18) | 0.330 | 0.91 (0.49 – 1.66) | 0.761 |
| Coffee consumption >4 cups/day | | 0.77 (0.52 – 1.11) | 0.181 | 0.78 (0.49 – 1.19) | 0.261 | 0.80 (0.47 – 1.30) | 0.382 | 0.76 (0.46 – 1.20) | 0.260 | 0.87 (0.39 – 1.78) | 0.707 |
| Age | |  |  | 1.03 (1.01 – 1.05) | **0.001** | 1.02 (1.00 – 1.04) | 0.058 | 1.03 (1.01 – 1.05) | **0.007** | 1.00 (0.97 – 1.04) | 0.985 |
| Diabetes mellitus | |  |  | 0.70 (0.43 – 1.10) | 0.139 | 0.69 (0.37 – 1.18) | 0.200 | 0.66 (0.38 – 1.08) | 0.120 |  |  |
| Arterial hypertension | |  |  | 1.06 (0.77 – 1.50) | 0.711 | 1.08 (0.73 – 1.60) | 0.714 | 1.01 (0.72 – 1.44) | 0.953 | 1.25 (0.64 – 2.59) | 0.527 |
| Current smoking | |  |  | 0.79 (0.53 – 1.14) | 0.216 | 0.86 (0.54 – 1.31) | 0.498 | 0.86 (0.57 – 1.26) | 0.464 | 0.97 (0.46 – 1.86) | 0.919 |
| BMI | |  |  | 1.04 (1.01 – 1.07) | **0.020** | 1.03 (0.99 – 1.07) | 0.148 | 1.03 (1.00 – 1.07) | 0.071 | 1.05 (0.99 – 1.11) | 0.070 |
| Additives | **Milk** |  |  |  |  | 1.19 (0.86 – 1.67) | 0.303 |  |  |  |  |
|  | **Binary** |  |  |  |  |  |  | 1.16 (0.85 – 1.61) | 0.371 |  |  |

**Supplementary Table 123. Multivariable logistic regression analysis for the association of mild/moderate/high coffee consumption and LBBB (only male).** Not daily coffee consumption (<1 cups/d) served as the reference group. Adjustment was performed for: A) unadjusted; B) main cardiovascular risk factors; C) additional adjustment for additives (separately), D) additional adjustment for additives (binary), E) same adjustment as for group A, but only for subjects who consumed coffee without any additives. Abbreviations as in Table 1.

|  | | A)AV_block | | B)AV_block | | C)AV block | | D)AV_block | | E)AV block | |
| --- | --- | --- | --- | --- | --- | --- | --- | --- | --- | --- | --- |
|  | | *Odds Ratios* | *p* | *Odds Ratios* | *p* | *Odds Ratios* | *p* | *Odds Ratios* | *p* | *Odds Ratios* | *p* |
| Coffee consumption 3-4 cups/day | | 0.77 (0.59 – 0.99) | **0.049** | 1.01 (0.75 – 1.34) | 0.952 | 1.04 (0.76 – 1.41) | 0.800 | 1.03 (0.76 – 1.39) | 0.856 | 1.01 (0.57 – 1.73) | 0.985 |
| Coffee consumption >4 cups/day | | 0.58 (0.39 – 0.83) | **0.004** | 0.82 (0.54 – 1.22) | 0.345 | 0.77 (0.48 – 1.19) | 0.266 | 0.81 (0.52 – 1.24) | 0.348 | 1.11 (0.56 – 2.09) | 0.758 |
| Age | |  |  | 1.08 (1.06 – 1.10) | **<0.001** | 1.09 (1.07 – 1.11) | **<0.001** | 1.08 (1.06 – 1.10) | **<0.001** | 1.09 (1.06 – 1.13) | **<0.001** |
| Diabetes mellitus | |  |  | 1.53 (1.10 – 2.11) | **0.010** | 1.51 (1.03 – 2.17) | **0.032** | 1.54 (1.08 – 2.17) | **0.015** | 2.58 (1.40 – 4.62) | **0.002** |
| Arterial hypertension | |  |  | 1.06 (0.78 – 1.45) | 0.735 | 1.05 (0.75 – 1.48) | 0.775 | 1.08 (0.78 – 1.52) | 0.631 | 0.57 (0.32 – 1.02) | 0.052 |
| Current smoking | |  |  | 0.75 (0.52 – 1.05) | 0.104 | 0.82 (0.55 – 1.18) | 0.296 | 0.82 (0.56 – 1.17) | 0.281 | 0.55 (0.25 – 1.10) | 0.113 |
| BMI | |  |  | 1.03 (1.00 – 1.06) | **0.046** | 1.03 (1.00 – 1.06) | 0.062 | 1.03 (1.00 – 1.06) | 0.057 | 1.02 (0.97 – 1.08) | 0.433 |
| Additives | **Milk** |  |  |  |  | 1.09 (0.82 – 1.46) | 0.548 |  |  |  |  |
|  | **Milk + Sugar** |  |  |  |  | 1.10 (0.69 – 1.72) | 0.687 |  |  |  |  |
|  | **Binary** |  |  |  |  |  |  | 1.07 (0.81 – 1.41) | 0.652 |  |  |

**Supplementary Table 124. Multivariable logistic regression analysis for the association of mild/moderate/high coffee consumption and AV block (only male).** Not daily coffee consumption (<1 cups/d) served as the reference group. Adjustment was performed for: A) unadjusted; B) main cardiovascular risk factors; C) additional adjustment for additives (separately), D) additional adjustment for additives (binary), E) same adjustment as for group A, but only for subjects who consumed coffee without any additives. Abbreviations as in Table 1.

|  | | A)Atrial_fibrillation | | B)Atrial_fibrillation | | C)Atrial_fibrillation | | D)Atrial_fibrillation | | E)Atrial_fibrillation | |
| --- | --- | --- | --- | --- | --- | --- | --- | --- | --- | --- | --- |
|  | | *Odds Ratios* | *p* | *Odds Ratios* | *p* | *Odds Ratios* | *p* | *Odds Ratios* | *p* | *Odds Ratios* | *p* |
| Coffee consumption 3-4 cups/day | | 0.68 (0.51 – 0.89) | **0.007** | 1.02 (0.74 – 1.38) | 0.919 | 0.92 (0.65 – 1.29) | 0.634 | 0.92 (0.66 – 1.27) | 0.625 | 0.93 (0.55 – 1.53) | 0.771 |
| Coffee consumption >4 cups/day | | 0.45 (0.29 – 0.68) | **<0.001** | 0.74 (0.45 – 1.15) | 0.200 | 0.64 (0.38 – 1.05) | 0.093 | 0.71 (0.43 – 1.12) | 0.155 | 0.52 (0.23 – 1.05) | 0.087 |
| Age | |  |  | 1.10 (1.07 – 1.12) | **<0.001** | 1.10 (1.07 – 1.12) | **<0.001** | 1.10 (1.07 – 1.12) | **<0.001** | 1.12 (1.08 – 1.16) | **<0.001** |
| Diabetes mellitus | |  |  | 1.39 (0.98 – 1.95) | 0.062 | 1.33 (0.89 – 1.94) | 0.150 | 1.26 (0.87 – 1.81) | 0.212 | 1.61 (0.88 – 2.87) | 0.112 |
| Arterial hypertension | |  |  | 3.71 (2.32 – 6.31) | **<0.001** | 4.15 (2.41 – 7.78) | **<0.001** | 3.92 (2.39 – 6.90) | **<0.001** |  |  |
| Current smoking | |  |  | 0.65 (0.43 – 0.97) | **0.040** | 0.79 (0.50 – 1.20) | 0.280 | 0.70 (0.45 – 1.05) | 0.098 | 0.57 (0.26 – 1.12) | 0.130 |
| BMI | |  |  | 1.03 (1.00 – 1.06) | 0.063 | 1.02 (0.99 – 1.06) | 0.183 | 1.03 (1.00 – 1.06) | 0.071 | 1.03 (0.97 – 1.08) | 0.339 |
| Additives | **Milk** |  |  |  |  | 0.66 (0.49 – 0.89) | **0.006** |  |  |  |  |
|  | **Milk + Sweetener** |  |  |  |  | 0.62 (0.27 – 1.30) | 0.239 |  |  |  |  |
|  | **Sweetener** |  |  |  |  | 0.37 (0.06 – 1.29) | 0.184 |  |  |  |  |
|  | **Binary** |  |  |  |  |  |  | 0.69 (0.52 – 0.91) | **0.008** |  |  |

**Supplementary Table 125. Multivariable logistic regression analysis for the association of mild/moderate/high coffee consumption and atrial fibrillation (only male).** Not daily coffee consumption (<1 cups/d) served as the reference group. Adjustment was performed for: A) unadjusted; B) main cardiovascular risk factors; C) additional adjustment for additives (separately), D) additional adjustment for additives (binary), E) same adjustment as for group A, but only for subjects who consumed coffee without any additives. Abbreviations as in Table 1.

|  | | A)Diabetes | | B)Diabetes | | C)Diabetes | | D)Diabetes | | E)Diabetes | |
| --- | --- | --- | --- | --- | --- | --- | --- | --- | --- | --- | --- |
|  | | *Odds Ratios* | *p* | *Odds Ratios* | *p* | *Odds Ratios* | *p* | *Odds Ratios* | *p* | *Odds Ratios* | *p* |
| Coffee consumption3 -4 cups/day | | 0.67 (0.52 – 0.86) | **0.002** | 0.80 (0.60 – 1.04) | 0.099 | 0.78 (0.58 – 1.05) | 0.105 | 0.82 (0.61 – 1.10) | 0.188 | 0.72 (0.43 – 1.19) | 0.211 |
| Coffee consumption >4 cups/day | | 0.72 (0.52 – 0.98) | **0.040** | 0.80 (0.56 – 1.13) | 0.222 | 0.86 (0.59 – 1.25) | 0.448 | 0.92 (0.63 – 1.32) | 0.653 | 0.85 (0.46 – 1.50) | 0.582 |
| Age | |  |  | 1.05 (1.03 – 1.06) | **<0.001** | 1.06 (1.04 – 1.07) | **<0.001** | 1.06 (1.04 – 1.07) | **<0.001** | 1.06 (1.03 – 1.09) | **<0.001** |
| Arterial hypertension | |  |  | 2.21 (1.58 – 3.17) | **<0.001** | 2.31 (1.58 – 3.49) | **<0.001** | 2.39 (1.64 – 3.61) | **<0.001** | 1.90 (1.01 – 3.92) | 0.060 |
| Current smoking | |  |  | 1.36 (1.03 – 1.79) | **0.029** | 1.44 (1.05 – 1.95) | **0.021** | 1.45 (1.06 – 1.95) | **0.017** | 1.46 (0.84 – 2.47) | 0.162 |
| BMI | |  |  | 1.15 (1.12 – 1.17) | **<0.001** | 1.14 (1.11 – 1.17) | **<0.001** | 1.14 (1.12 – 1.17) | **<0.001** | 1.14 (1.09 – 1.19) | **<0.001** |
| Additives | **Milk** |  |  |  |  | 0.95 (0.72 – 1.24) | 0.683 |  |  |  |  |
|  | **Milk + Sugar** |  |  |  |  | 0.41 (0.22 – 0.70) | **0.002** |  |  |  |  |
|  | **Milk + Sweetener** |  |  |  |  | 3.89 (2.41 – 6.22) | **<0.001** |  |  |  |  |
|  | **Sweetener** |  |  |  |  | 3.04 (1.37 – 6.41) | **0.004** |  |  |  |  |
|  | **Binary** |  |  |  |  |  |  | 0.99 (0.77 – 1.28) | 0.940 |  |  |

**Supplementary Table 126. Multivariable logistic regression analysis for the association of mild/moderate/high coffee consumption and diabetes (only male).** Not daily coffee consumption (<1 cups/d) served as the reference group. Adjustment was performed for: A) unadjusted; B) main cardiovascular risk factors; C) additional adjustment for additives (separately), D) additional adjustment for additives (binary), E) same adjustment as for group A, but only for subjects who consumed coffee without any additives. Abbreviations as in Table 1

|  | | A)Obesity | | B)Obesity | | C)Obesity | | D)Obesity | | E)Obesity | |
| --- | --- | --- | --- | --- | --- | --- | --- | --- | --- | --- | --- |
|  | | *Odds Ratios* | *p* | *Odds Ratios* | *p* | *Odds Ratios* | *p* | *Odds Ratios* | *p* | *Odds Ratios* | *p* |
| Coffee consumption 3-4 cups/day | | 0.97 (0.81 – 1.15) | 0.713 | 1.07 (0.88 – 1.30) | 0.471 | 1.05 (0.86 – 1.29) | 0.613 | 1.08 (0.88 – 1.32) | 0.470 | 1.00 (0.70 – 1.42) | 0.993 |
| Coffee consumption >4 cups/day | | 1.01 (0.81 – 1.26) | 0.928 | 1.17 (0.91 – 1.49) | 0.209 | 1.13 (0.87 – 1.46) | 0.361 | 1.14 (0.88 – 1.48) | 0.318 | 1.11 (0.73 – 1.68) | 0.616 |
| Age | |  |  | 0.99 (0.98 – 1.00) | **0.007** | 0.99 (0.98 – 1.00) | **0.026** | 0.99 (0.98 – 1.00) | **0.035** | 0.98 (0.96 – 1.00) | **0.042** |
| Diabetes mellitus | |  |  | 2.90 (2.31 – 3.62) | **<0.001** | 2.70 (2.11 – 3.46) | **<0.001** | 2.85 (2.23 – 3.64) | **<0.001** | 3.08 (2.00 – 4.73) | **<0.001** |
| Arterial hypertension | |  |  | 3.10 (2.47 – 3.92) | **<0.001** | 3.03 (2.38 – 3.92) | **<0.001** | 3.09 (2.41 – 3.99) | **<0.001** | 3.68 (2.40 – 5.86) | **<0.001** |
| Current smoking | |  |  | 0.95 (0.76 – 1.17) | 0.606 | 0.92 (0.73 – 1.16) | 0.499 | 0.92 (0.73 – 1.16) | 0.494 | 0.90 (0.60 – 1.32) | 0.589 |
| Additives | **Milk** |  |  |  |  | 0.77 (0.64 – 0.94) | **0.009** |  |  |  |  |
|  | **Milk + Sugar** |  |  |  |  | 0.58 (0.41 – 0.81) | **0.002** |  |  |  |  |
|  | **Milk + Sweetener** |  |  |  |  | 0.91 (0.57 – 1.43) | 0.695 |  |  |  |  |
|  | **Sugar** |  |  |  |  | 0.93 (0.48 – 1.69) | 0.828 |  |  |  |  |
|  | **Sweetener** |  |  |  |  | 2.34 (1.16 – 4.68) | **0.016** |  |  |  |  |
|  | **Binary** |  |  |  |  |  |  | 0.76 (0.63 – 0.92) | **0.004** |  |  |

**Supplementary Table 127. Multivariable logistic regression analysis for the association of mild/moderate/high coffee consumption and obesity (only male).** Not daily coffee consumption (<1 cups/d) served as the reference group. Adjustment was performed for: A) unadjusted; B) main cardiovascular risk factors; C) additional adjustment for additives (separately), D) additional adjustment for additives (binary), E) same adjustment as for group A, but only for subjects who consumed coffee without any additives. Abbreviations as in Table 1.

|  | | A)CAD | | B)CAD | | C)CAD | | D)CAD | | E)CAD | |
| --- | --- | --- | --- | --- | --- | --- | --- | --- | --- | --- | --- |
|  | | *Odds Ratios* | *p* | *Odds Ratios* | *p* | *Odds Ratios* | *p* | *Odds Ratios* | *p* | *Odds Ratios* | *p* |
| Coffee consumption 3-4 cups/day | | 0.67 (0.52 – 0.85) | **0.001** | 0.94 (0.71 – 1.23) | 0.641 | 0.94 (0.70 – 1.26) | 0.688 | 0.93 (0.69 – 1.24) | 0.612 | 0.84 (0.51 – 1.36) | 0.474 |
| Coffee consumption >4 cups/day | | 0.75 (0.55 – 1.01) | 0.067 | 1.09 (0.76 – 1.54) | 0.645 | 1.12 (0.76 – 1.62) | 0.567 | 1.08 (0.73 – 1.57) | 0.692 | 1.18 (0.66 – 2.06) | 0.561 |
| Age | |  |  | 1.08 (1.06 – 1.09) | **<0.001** | 1.08 (1.06 – 1.10) | **<0.001** | 1.08 (1.06 – 1.10) | **<0.001** | 1.07 (1.04 – 1.10) | **<0.001** |
| Diabetes mellitus | |  |  | 1.59 (1.17 – 2.15) | **0.002** | 1.49 (1.05 – 2.08) | **0.022** | 1.49 (1.06 – 2.07) | **0.018** | 2.20 (1.26 – 3.80) | **0.005** |
| Arterial hypertension | |  |  | 5.11 (3.34 – 8.22) | **<0.001** | 4.47 (2.86 – 7.40) | **<0.001** | 4.38 (2.82 – 7.16) | **<0.001** | 3.70 (1.83 – 8.54) | **0.001** |
| Current smoking | |  |  | 0.97 (0.70 – 1.32) | 0.839 | 0.99 (0.70 – 1.39) | 0.955 | 1.04 (0.74 – 1.44) | 0.832 | 0.96 (0.53 – 1.65) | 0.886 |
| BMI | |  |  | 1.05 (1.02 – 1.07) | **0.001** | 1.05 (1.02 – 1.08) | **0.001** | 1.05 (1.02 – 1.08) | **0.001** | 1.05 (1.00 – 1.10) | **0.042** |
| Additives | **Milk** |  |  |  |  | 0.90 (0.69 – 1.17) | 0.427 |  |  |  |  |
|  | **Milk + Sugar** |  |  |  |  | 0.61 (0.36 – 0.98) | **0.048** |  |  |  |  |
|  | **Milk + Sweetener** |  |  |  |  | 0.56 (0.25 – 1.14) | 0.131 |  |  |  |  |
|  | **Sweetener** |  |  |  |  | 1.10 (0.38 – 2.75) | 0.853 |  |  |  |  |
|  | **Binary** |  |  |  |  |  |  | 0.84 (0.65 – 1.08) | 0.170 |  |  |

**Supplementary Table 128. Multivariable logistic regression analysis for the association of mild/moderate/high coffee consumption and CAD (only male).** Not daily coffee consumption (<1 cups/d) served as the reference group. Adjustment was performed for: A) unadjusted; B) main cardiovascular risk factors; C) additional adjustment for additives (separately), D) additional adjustment for additives (binary), E) same adjustment as for group A, but only for subjects who consumed coffee without any additives. Abbreviations as in Table 1.

|  | | A)PAD | | B)PAD | | C)PAD | | D)PAD | | E)PAD | |
| --- | --- | --- | --- | --- | --- | --- | --- | --- | --- | --- | --- |
|  | | *Odds Ratios* | *p* | *Odds Ratios* | *p* | *Odds Ratios* | *p* | *Odds Ratios* | *p* | *Odds Ratios* | *p* |
| Coffee consumption 3-4 cups/day | | 0.89 (0.69 – 1.14) | 0.360 | 1.14 (0.86 – 1.51) | 0.359 | 1.09 (0.80 – 1.48) | 0.575 | 1.11 (0.82 – 1.51) | 0.482 | 1.01 (0.59 – 1.70) | 0.966 |
| Coffee consumption >4 cups/day | | 0.83 (0.59 – 1.16) | 0.297 | 0.98 (0.66 – 1.42) | 0.911 | 0.90 (0.59 – 1.35) | 0.619 | 0.91 (0.60 – 1.36) | 0.655 | 1.28 (0.68 – 2.33) | 0.431 |
| Age | |  |  | 1.05 (1.03 – 1.06) | **<0.001** | 1.05 (1.03 – 1.07) | **<0.001** | 1.04 (1.03 – 1.06) | **<0.001** | 1.05 (1.02 – 1.08) | **0.004** |
| Diabetes mellitus | |  |  | 2.34 (1.67 – 3.26) | **<0.001** | 2.17 (1.49 – 3.15) | **<0.001** | 2.11 (1.46 – 3.03) | **<0.001** | 2.52 (1.28 – 4.88) | **0.007** |
| Arterial hypertension | |  |  | 1.32 (0.98 – 1.79) | 0.074 | 1.32 (0.95 – 1.85) | 0.102 | 1.34 (0.97 – 1.87) | 0.082 | 1.10 (0.64 – 1.95) | 0.728 |
| Current smoking | |  |  | 1.81 (1.35 – 2.42) | **<0.001** | 1.98 (1.43 – 2.71) | **<0.001** | 1.96 (1.43 – 2.68) | **<0.001** | 1.47 (0.83 – 2.54) | 0.171 |
| BMI | |  |  | 1.06 (1.03 – 1.09) | **<0.001** | 1.07 (1.03 – 1.10) | **<0.001** | 1.06 (1.03 – 1.10) | **<0.001** | 1.07 (1.02 – 1.13) | **0.011** |
| Additives | **Milk** |  |  |  |  | 0.92 (0.69 – 1.23) | 0.553 |  |  |  |  |
|  | **Milk + Sugar** |  |  |  |  | 0.79 (0.49 – 1.25) | 0.334 |  |  |  |  |
|  | **Milk + Sweetener** |  |  |  |  | 0.95 (0.46 – 1.87) | 0.887 |  |  |  |  |
|  | **Sweetener** |  |  |  |  | 0.54 (0.12 – 1.81) | 0.365 |  |  |  |  |
|  | **Binary** |  |  |  |  |  |  | 0.92 (0.70 – 1.21) | 0.546 |  |  |

**Supplementary Table 129. Multivariable logistic regression analysis for the association of mild/moderate/high coffee consumption and PAD (only male).** Not daily coffee consumption (<1 cups/d) served as the reference group. Adjustment was performed for: A) unadjusted; B) main cardiovascular risk factors; C) additional adjustment for additives (separately), D) additional adjustment for additives (binary), E) same adjustment as for group A, but only for subjects who consumed coffee without any additives. Abbreviations as in Table 1.

|  | | A)Heart failure | | B)Heart failure | | C)Heart failure | | D)Heart failure | | E)Heart failure | |
| --- | --- | --- | --- | --- | --- | --- | --- | --- | --- | --- | --- |
|  | | *Odds Ratios* | *p* | *Odds Ratios* | *p* | *Odds Ratios* | *p* | *Odds Ratios* | *p* | *Odds Ratios* | *p* |
| Coffee consumption 3-4 cups/day | | 0.62 (0.42 – 0.90) | **0.015** | 0.83 (0.53 – 1.25) | 0.373 | 0.81 (0.51 – 1.26) | 0.372 | 0.83 (0.53 – 1.29) | 0.422 | 0.50 (0.20 – 1.12) | 0.110 |
| Coffee consumption >4 cups/day | | 0.70 (0.42 – 1.10) | 0.138 | 1.13 (0.65 – 1.86) | 0.652 | 0.92 (0.49 – 1.63) | 0.794 | 0.87 (0.46 – 1.55) | 0.644 | 0.59 (0.19 – 1.53) | 0.310 |
| Age | |  |  | 1.08 (1.06 – 1.11) | **<0.001** | 1.08 (1.05 – 1.11) | **<0.001** | 1.08 (1.05 – 1.11) | **<0.001** | 1.12 (1.06 – 1.18) | **<0.001** |
| Diabetes mellitus | |  |  | 1.47 (0.94 – 2.23) | 0.081 | 1.63 (1.01 – 2.58) | **0.042** | 1.54 (0.95 – 2.42) | 0.071 | 2.51 (1.14 – 5.25) | **0.018** |
| Arterial hypertension | |  |  | 2.99 (1.68 – 5.82) | **<0.001** | 2.96 (1.59 – 6.17) | **0.002** | 3.06 (1.64 – 6.35) | **0.001** |  |  |
| Current smoking | |  |  | 1.25 (0.78 – 1.94) | 0.337 | 1.40 (0.83 – 2.26) | 0.188 | 1.34 (0.80 – 2.15) | 0.250 | 2.31 (0.93 – 5.37) | 0.058 |
| BMI | |  |  | 1.11 (1.07 – 1.16) | **<0.001** | 1.11 (1.06 – 1.16) | **<0.001** | 1.11 (1.06 – 1.15) | **<0.001** | 1.14 (1.07 – 1.23) | **<0.001** |
| Additives | **Milk** |  |  |  |  | 0.98 (0.66 – 1.47) | 0.913 |  |  |  |  |
|  | **Milk + Sugar** |  |  |  |  | 0.84 (0.40 – 1.64) | 0.619 |  |  |  |  |
|  | **Milk + Sweetener** |  |  |  |  | 0.26 (0.04 – 0.88) | 0.069 |  |  |  |  |
|  | **Sweetener** |  |  |  |  | 2.06 (0.45 – 6.79) | 0.279 |  |  |  |  |
|  | **Binary** |  |  |  |  |  |  | 0.91 (0.62 – 1.36) | 0.645 |  |  |

**Supplementary Table 130. Multivariable logistic regression analysis for the association of mild/moderate/high coffee consumption and heart failure (only male).** Not daily coffee consumption (<1 cups/d) served as the reference group. Adjustment was performed for: A) unadjusted; B) main cardiovascular risk factors; C) additional adjustment for additives (separately), D) additional adjustment for additives (binary), E) same adjustment as for group A, but only for subjects who consumed coffee without any additives. Abbreviations as in Table 1.

|  | | A)HF(m)rEF | | B)HF(m)rEF | | C)HF(m)rEF | | D)HF(m)rEF | | E)HF(m)rEF | |
| --- | --- | --- | --- | --- | --- | --- | --- | --- | --- | --- | --- |
|  | | *Odds Ratios* | *p* | *Odds Ratios* | *p* | *Odds Ratios* | *p* | *Odds Ratios* | *p* | *Odds Ratios* | *p* |
| Coffee consumption 3-4 cups/day | | 0.74 (0.47 – 1.14) | 0.186 | 0.92 (0.55 – 1.48) | 0.735 | 0.98 (0.56 – 1.64) | 0.930 | 0.99 (0.57 – 1.65) | 0.961 | 0.73 (0.23 – 2.00) | 0.560 |
| Coffee consumption >4 cups/day | | 0.73 (0.39 – 1.25) | 0.279 | 1.10 (0.57 – 2.01) | 0.758 | 1.13 (0.54 – 2.18) | 0.726 | 1.06 (0.49 – 2.09) | 0.869 | 0.85 (0.19 – 2.87) | 0.816 |
| Age | |  |  | 1.08 (1.05 – 1.11) | **<0.001** | 1.07 (1.04 – 1.11) | **<0.001** | 1.08 (1.04 – 1.11) | **<0.001** | 1.14 (1.06 – 1.23) | **0.001** |
| Diabetes mellitus | |  |  | 1.47 (0.87 – 2.41) | 0.133 | 1.59 (0.88 – 2.75) | 0.109 | 1.50 (0.83 – 2.59) | 0.161 | 2.43 (0.86 – 6.22) | 0.075 |
| Arterial hypertension | |  |  | 3.58 (1.74 – 8.67) | **0.002** | 3.46 (1.59 – 9.10) | **0.005** | 3.45 (1.59 – 9.05) | **0.005** |  |  |
| Current smoking  BMI | |  |  | 1.20 (0.68 – 2.02) | 0.505 | 1.29 (0.68 – 2.28) | 0.410 | 1.27 (0.68 – 2.25) | 0.428 | 1.76 (0.47 – 5.43) | 0.356 |
|  |  |  |  | 1.10 (1.05 – 1.15) | **<0.001** | 1.09 (1.03 – 1.14) | **0.001** | 1.08 (1.03 – 1.14) | **0.002** | 1.10 (1.00 – 1.20) | **0.043** |
| Additives | **Milk** |  |  |  |  | 1.36 (0.82 – 2.30) | 0.242 |  |  |  |  |
|  | **Milk + Sugar** |  |  |  |  | 1.18 (0.48 – 2.62) | 0.702 |  |  |  |  |
|  | **Milk + Sweetener** |  |  |  |  | 0.26 (0.01 – 1.28) | 0.190 |  |  |  |  |
|  | **Sweetener** |  |  |  |  | 3.77 (0.82 – 12.69) | 0.050 |  |  |  |  |
|  | **Binary** |  |  |  |  |  |  | 1.24 (0.76 – 2.07) | 0.403 |  |  |

**Supplementary Table 131. Multivariable logistic regression analysis for the association of mild/moderate/high coffee consumption and HF(m)rEF (only male).** Not daily coffee consumption (<1 cups/d) served as the reference group. Adjustment was performed for: A) unadjusted; B) main cardiovascular risk factors; C) additional adjustment for additives (separately), D) additional adjustment for additives (binary), E) same adjustment as for group A, but only for subjects who consumed coffee without any additives. Abbreviations as in Table 1.

|  | | A)Total cholesterol | | B)Total cholesterol | | C)Total cholesterol | | D)Total cholesterol | | E)Total cholesterol | | F)Total cholesterol | |
| --- | --- | --- | --- | --- | --- | --- | --- | --- | --- | --- | --- | --- | --- |
|  | | *Estimates* | *p* | *Estimates* | *p* | *Estimates* | *p* | *Estimates* | *p* | *Estimates* | *p* | *Estimates* | *p* |
| Coffee consumption 3-4 cups/day | | 3.97 (-1.12 – 9.06) | 0.126 | 4.57 (-0.72 – 9.87) | 0.090 | 3.93 (-1.60 – 9.45) | 0.163 | 3.96 (-1.59 – 9.50) | 0.162 | 3.83 (-1.13 – 8.78) | 0.130 | -1.14 (-11.43 – 9.14) | 0.827 |
| Coffee consumption >4 cups/day | | 0.78 (-6.15 – 7.72) | 0.824 | 5.32 (-2.11 – 12.74) | 0.160 | 4.52 (-3.36 – 12.40) | 0.261 | 4.51 (-3.40 – 12.42) | 0.264 | 5.49 (-1.44 – 12.43) | 0.120 | 0.31 (-12.66 – 13.28) | 0.962 |
| Age | |  |  | -0.11 (-0.40 – 0.18) | 0.455 | -0.15 (-0.46 – 0.16) | 0.353 | -0.14 (-0.45 – 0.17) | 0.388 | 0.31 (0.03 – 0.59) | **0.029** | -0.28 (-0.88 – 0.31) | 0.352 |
| Female | |  |  | 24.02 (19.36 – 28.68) | **<0.001** | 24.91 (20.00 – 29.83) | **<0.001** | 24.95 (20.03 – 29.88) | **<0.001** | 22.01 (17.64 – 26.37) | **<0.001** | 22.98 (13.96 – 32.00) | **<0.001** |
| Diabetes mellitus | |  |  | -24.20 (-32.49 – -15.90) | **<0.001** | -22.03 (-30.90 – -13.17) | **<0.001** | -22.44 (-31.39 – -13.50) | **<0.001** | -13.80 (-21.75 – -5.86) | **0.001** | -25.30 (-41.85 – -8.74) | **0.003** |
| Arterial hypertension | |  |  | 10.20 (4.72 – 15.68) | **<0.001** | 9.72 (3.94 – 15.50) | **0.001** | 9.52 (3.72 – 15.31) | **0.001** | 13.54 (8.40 – 18.68) | **<0.001** | 15.71 (5.16 – 26.25) | **0.004** |
| Current smoking | |  |  | -3.81 (-9.35 – 1.74) | 0.179 | -4.73 (-10.67 – 1.22) | 0.119 | -4.59 (-10.54 – 1.36) | 0.130 | -2.36 (-7.55 – 2.83) | 0.372 | -9.60 (-20.72 – 1.53) | 0.091 |
| BMI | |  |  | -0.09 (-0.60 – 0.41) | 0.714 | -0.15 (-0.69 – 0.38) | 0.575 | -0.15 (-0.69 – 0.38) | 0.576 | -0.06 (-0.53 – 0.42) | 0.811 | -0.46 (-1.41 – 0.49) | 0.341 |
| Additives | **Milk** |  |  |  |  | -5.35 (-10.81 – 0.11) | 0.055 |  |  |  |  |  |  |
|  | **Milk + Sugar** |  |  |  |  | -4.49 (-13.85 – 4.87) | 0.347 |  |  |  |  |  |  |
|  | **Milk + Sweetener** |  |  |  |  | -2.16 (-15.25 – 10.93) | 0.746 |  |  |  |  |  |  |
|  | **Sugar** |  |  |  |  | -0.17 (-19.63 – 19.28) | 0.986 |  |  |  |  |  |  |
|  | **Sweetener** |  |  |  |  | 0.79 (-16.99 – 18.57) | 0.930 |  |  |  |  |  |  |
|  | **Binary** |  |  |  |  |  |  | -4.96 (-10.19 – 0.28) | 0.063 |  |  |  |  |
| Lipid lowering drugs | |  |  |  |  |  |  |  |  | -40.67 (-46.56 – -34.77) | **<0.001** |  |  |

**Supplementary Table 132. Multivariable linear regression analysis for the association of mild/moderate/high coffee consumption and total cholesterol (excluding black and green tea consumption).** “Not daily” coffee consumption (<1 cups/d) served as the reference group. Adjustment was performed for: A) unadjusted; B) main cardiovascular risk factors; C) additional adjustment for additives (separately), D) additional adjustment for additives (binary), E) additional adjustment for lipid lowering drugs, F) same adjustment as for group A, but only for subjects who consumed coffee without any additives. Abbreviations as in Table 1

|  | | A)LDL | | B)LDL | | C)LDL | | D)LDL | | E)LDL | | F)LDL | |
| --- | --- | --- | --- | --- | --- | --- | --- | --- | --- | --- | --- | --- | --- |
|  | | *Estimates* | *p* | *Estimates* | *p* | *Estimates* | *p* | *Estimates* | *p* | *Estimates* | *p* | *Estimates* | *p* |
| Coffee consumption 3-4 cups/day | | 4.22 (-0.29 – 8.72) | 0.067 | 4.03 (-0.75 – 8.81) | 0.098 | 2.98 (-1.99 – 7.95) | 0.240 | 2.83 (-2.16 – 7.83) | 0.266 | 3.40 (-1.07 – 7.88) | 0.136 | 1.89 (-7.26 – 11.05) | 0.684 |
| Coffee consumption >4 cups/day | | 6.82 (0.61 – 13.03) | **0.031** | 7.66 (0.89 – 14.43) | **0.027** | 6.60 (-0.57 – 13.77) | 0.071 | 6.24 (-0.97 – 13.45) | 0.090 | 7.84 (1.51 – 14.18) | **0.015** | 4.75 (-6.83 – 16.33) | 0.421 |
| Age | |  |  | -0.18 (-0.44 – 0.09) | 0.187 | -0.20 (-0.48 – 0.08) | 0.160 | -0.20 (-0.49 – 0.08) | 0.153 | 0.20 (-0.06 – 0.45) | 0.125 | -0.28 (-0.81 – 0.25) | 0.303 |
| Female | |  |  | 11.17 (6.95 – 15.38) | **<0.001** | 11.64 (7.20 – 16.08) | **<0.001** | 11.54 (7.09 – 15.99) | **<0.001** | 9.41 (5.45 – 13.36) | **<0.001** | 9.18 (1.15 – 17.21) | **0.025** |
| Diabetes mellitus | |  |  | -24.90 (-32.56 – -17.24) | **<0.001** | -21.98 (-30.15 – -13.81) | **<0.001** | -22.06 (-30.30 – -13.81) | **<0.001** | -15.69 (-23.04 – -8.35) | **<0.001** | -25.15 (-40.37 – -9.93) | **0.001** |
| Arterial hypertension | |  |  | 7.02 (2.07 – 11.96) | **0.005** | 6.42 (1.21 – 11.62) | **0.016** | 6.25 (1.03 – 11.48) | **0.019** | 9.98 (5.33 – 14.63) | **<0.001** | 10.77 (1.40 – 20.15) | **0.024** |
| Current smoking | |  |  | -1.52 (-6.55 – 3.50) | 0.552 | -2.13 (-7.49 – 3.24) | 0.437 | -1.74 (-7.11 – 3.63) | 0.525 | -0.20 (-4.91 – 4.50) | 0.932 | -5.46 (-15.39 – 4.46) | 0.280 |
| BMI | |  |  | 0.52 (0.05 – 0.98) | **0.028** | 0.47 (-0.01 – 0.96) | 0.055 | 0.48 (-0.01 – 0.97) | 0.054 | 0.55 (0.12 – 0.98) | **0.013** | 0.21 (-0.64 – 1.05) | 0.631 |
| Additives | **Milk** |  |  |  |  | -3.49 (-8.41 – 1.43) | 0.164 |  |  |  |  |  |  |
|  | **Milk + Sugar** |  |  |  |  | -1.28 (-9.69 – 7.14) | 0.766 |  |  |  |  |  |  |
|  | **Milk + Sweetener** |  |  |  |  | 1.59 (-10.41 – 13.58) | 0.795 |  |  |  |  |  |  |
|  | **Sugar** |  |  |  |  | 1.26 (-16.73 – 19.25) | 0.891 |  |  |  |  |  |  |
|  | **Sweetener** |  |  |  |  | -0.84 (-17.14 – 15.46) | 0.919 |  |  |  |  |  |  |
|  | **Binary** |  |  |  |  |  |  | -2.87 (-7.60 – 1.86) | 0.234 |  |  |  |  |
| Lipid lowering drugs | |  |  |  |  |  |  |  |  | -36.35 (-41.71 – -30.98) | **<0.001** |  |  |

**Supplementary Table 133. Multivariable linear regression analysis for the association of mild/moderate/high coffee consumption and LDL (excluding black and green tea consumption).** “Not daily” coffee consumption (<1 cups/d) served as the reference group. Adjustment was performed for: A) unadjusted; B) main cardiovascular risk factors; C) additional adjustment for additives (separately), D) additional adjustment for additives (binary), E) additional adjustment for lipid lowering drugs, F) same adjustment as for group A, but only for subjects who consumed coffee without any additives. Abbreviations as in Table 1

|  | | A)HDL | | B)HDL | | C)HDL | | D)HDL | | E)HDL | | F)HDL | |
| --- | --- | --- | --- | --- | --- | --- | --- | --- | --- | --- | --- | --- | --- |
|  | | *Estimates* | *p* | *Estimates* | *p* | *Estimates* | *p* | *Estimates* | *p* | *Estimates* | *p* | *Estimates* | *p* |
| Coffee consumption3-4 cups/day | | 0.76 (-1.51 – 3.02) | 0.514 | 2.40 (0.35 – 4.44) | **0.022** | 2.37 (0.20 – 4.54) | **0.032** | 2.46 (0.27 – 4.64) | **0.028** | 2.33 (0.29 – 4.38) | **0.025** | -0.04 (-4.25 – 4.16) | 0.984 |
| Coffee consumption >4 cups/day | | -8.08 (-11.17 – -4.99) | **<0.001** | -2.12 (-5.00 – 0.75) | 0.148 | -2.67 (-5.76 – 0.43) | 0.091 | -2.60 (-5.72 – 0.52) | 0.102 | -2.08 (-4.94 – 0.78) | 0.154 | -1.41 (-6.72 – 3.89) | 0.601 |
| Age | |  |  | 0.14 (0.03 – 0.26) | **0.013** | 0.09 (-0.03 – 0.22) | 0.130 | 0.11 (-0.01 – 0.23) | 0.077 | 0.19 (0.07 – 0.31) | **0.001** | 0.09 (-0.16 – 0.33) | 0.481 |
| Female | |  |  | 15.67 (13.87 – 17.47) | **<0.001** | 15.59 (13.66 – 17.52) | **<0.001** | 15.74 (13.79 – 17.68) | **<0.001** | 15.47 (13.67 – 17.26) | **<0.001** | 16.22 (12.53 – 19.91) | **<0.001** |
| Diabetes mellitus | |  |  | -6.74 (-9.95 – -3.53) | **<0.001** | -7.19 (-10.67 – -3.71) | **<0.001** | -7.47 (-11.00 – -3.94) | **<0.001** | -5.71 (-8.99 – -2.43) | **0.001** | -10.85 (-17.62 – -4.08) | **0.002** |
| Arterial hypertension | |  |  | -0.42 (-2.54 – 1.71) | 0.701 | -0.11 (-2.38 – 2.16) | 0.921 | -0.14 (-2.42 – 2.15) | 0.906 | -0.04 (-2.16 – 2.08) | 0.970 | 1.84 (-2.47 – 6.15) | 0.401 |
| Current smoking | |  |  | -5.93 (-8.08 – -3.78) | **<0.001** | -5.85 (-8.18 – -3.51) | **<0.001** | -6.03 (-8.38 – -3.69) | **<0.001** | -5.76 (-7.90 – -3.62) | **<0.001** | -7.28 (-11.82 – -2.73) | **0.002** |
| BMI | |  |  | -1.25 (-1.44 – -1.05) | **<0.001** | -1.28 (-1.49 – -1.07) | **<0.001** | -1.29 (-1.50 – -1.08) | **<0.001** | -1.24 (-1.44 – -1.05) | **<0.001** | -1.33 (-1.72 – -0.94) | **<0.001** |
| Additives | **Milk** |  |  |  |  | -2.34 (-4.48 – -0.19) | **0.033** |  |  |  |  |  |  |
|  | **Milk + Sugar** |  |  |  |  | -4.97 (-8.64 – -1.29) | **0.008** |  |  |  |  |  |  |
|  | **Milk + Sweetener** |  |  |  |  | -6.00 (-11.14 – -0.86) | **0.022** |  |  |  |  |  |  |
|  | **Sugar** |  |  |  |  | -2.18 (-9.82 – 5.47) | 0.577 |  |  |  |  |  |  |
|  | **Sweetener** |  |  |  |  | 0.39 (-6.59 – 7.37) | 0.913 |  |  |  |  |  |  |
|  | **Binary** |  |  |  |  |  |  | -2.84 (-4.91 – -0.78) | **0.007** |  |  |  |  |
| Lipid lowering drugs | |  |  |  |  |  |  |  |  | -4.55 (-6.98 – -2.12) | **<0.001** |  |  |

**Supplementary Table 134. Multivariable linear regression analysis for the association of mild/moderate/high coffee consumption and HDL (excluding black and green tea consumption).** “Not daily” coffee consumption (<1 cups/d) served as the reference group. Adjustment was performed for: A) unadjusted; B) main cardiovascular risk factors; C) additional adjustment for additives (separately), D) additional adjustment for additives (binary), E) additional adjustment for lipid lowering drugs, F) same adjustment as for group A, but only for subjects who consumed coffee without any additives. Abbreviations as in Table 1

|  | | A)NT pro-BNP | | B)NT pro-BNP | | C)NT pro-BNP | | D)NT pro-BNP | | E)NT pro-BNP | |
| --- | --- | --- | --- | --- | --- | --- | --- | --- | --- | --- | --- |
|  | | *Estimates* | *p* | *Estimates* | *p* | *Estimates* | *p* | *Estimates* | *p* | *Estimates* | *p* |
| Coffee consumption 3-4 cups/day | | -0.25 (-0.36 – -0.14) | **<0.001** | -0.11 (-0.22 – -0.00) | **0.043** | -0.10 (-0.21 – 0.01) | 0.073 | -0.09 (-0.20 – 0.02) | 0.102 | 0.03 (-0.18 – 0.24) | 0.777 |
| Coffee consumption >4 cups/day | | -0.38 (-0.53 – -0.23) | **<0.001** | -0.15 (-0.30 – 0.00) | 0.051 | -0.14 (-0.30 – 0.02) | 0.087 | -0.14 (-0.30 – 0.01) | 0.076 | -0.04 (-0.30 – 0.22) | 0.750 |
| Age | |  |  | 0.05 (0.04 – 0.05) | **<0.001** | 0.05 (0.04 – 0.06) | **<0.001** | 0.05 (0.04 – 0.06) | **<0.001** | 0.06 (0.04 – 0.07) | **<0.001** |
| Female | |  |  | 0.35 (0.25 – 0.44) | **<0.001** | 0.32 (0.22 – 0.42) | **<0.001** | 0.32 (0.22 – 0.42) | **<0.001** | 0.28 (0.09 – 0.46) | **0.003** |
| Diabetes mellitus | |  |  | -0.01 (-0.18 – 0.15) | 0.870 | -0.05 (-0.23 – 0.13) | 0.588 | -0.03 (-0.20 – 0.15) | 0.773 | 0.22 (-0.10 – 0.55) | 0.176 |
| Arterial hypertension | |  |  | 0.08 (-0.03 – 0.19) | 0.130 | 0.10 (-0.01 – 0.22) | 0.082 | 0.10 (-0.01 – 0.22) | 0.085 | 0.11 (-0.10 – 0.32) | 0.294 |
| Current smoking | |  |  | 0.08 (-0.03 – 0.19) | 0.146 | 0.10 (-0.02 – 0.22) | 0.108 | 0.10 (-0.02 – 0.22) | 0.094 | 0.15 (-0.07 – 0.37) | 0.174 |
| BMI | |  |  | -0.01 (-0.02 – 0.00) | 0.058 | -0.01 (-0.02 – -0.00) | **0.044** | -0.01 (-0.02 – -0.00) | **0.044** | -0.01 (-0.03 – 0.01) | 0.523 |
| Additives | **Milk** |  |  |  |  | 0.01 (-0.10 – 0.12) | 0.898 |  |  |  |  |
|  | **Milk + Sugar** |  |  |  |  | -0.05 (-0.24 – 0.14) | 0.626 |  |  |  |  |
|  | **Milk + Sweetener** |  |  |  |  | -0.24 (-0.51 – 0.02) | 0.071 |  |  |  |  |
|  | **Sugar** |  |  |  |  | -0.09 (-0.48 – 0.30) | 0.657 |  |  |  |  |
|  | **Sweetener** |  |  |  |  | -0.15 (-0.50 – 0.21) | 0.423 |  |  |  |  |
|  | **Binary** |  |  |  |  |  |  | -0.02 (-0.12 – 0.09) | 0.737 |  |  |

**Supplementary Table 135. Multivariable linear regression analysis for the association of mild/moderate/high coffee consumption and NT proBNP (excluding black and green tea consumption).** “Not daily” coffee consumption (<1 cups/d) served as the reference group. Adjustment was performed for: A) unadjusted; B) main cardiovascular risk factors; C) additional adjustment for additives (separately), D) additional adjustment for additives (binary), E) additional adjustment for lipid lowering drugs, F) same adjustment as for group A, but only for subjects who consumed coffee without any additives. Abbreviations as in Table 1

|  | | A)SBP | | B)SBP | | C)SBP | | D)SBP | | E)SBP | |
| --- | --- | --- | --- | --- | --- | --- | --- | --- | --- | --- | --- |
|  | | *Estimates* | *p* | *Estimates* | *p* | *Estimates* | *p* | *Estimates* | *p* | *Estimates* | *p* |
| Coffee consumption 3-4 cups/day | | -4.71 (-7.35 – -2.07) | **<0.001** | -2.01 (-4.58 – 0.56) | 0.126 | -2.13 (-4.83 – 0.58) | 0.123 | -2.29 (-4.99 – 0.41) | 0.097 | -3.33 (-8.19 – 1.53) | 0.179 |
| Coffee consumption >4 cups/day | | -3.57 (-7.19 – 0.05) | 0.053 | -2.06 (-5.64 – 1.52) | 0.260 | -2.80 (-6.64 – 1.04) | 0.153 | -2.55 (-6.39 – 1.28) | 0.192 | -5.67 (-11.77 – 0.43) | 0.068 |
| Age | |  |  | 0.82 (0.68 – 0.95) | **<0.001** | 0.81 (0.67 – 0.96) | **<0.001** | 0.81 (0.67 – 0.95) | **<0.001** | 0.73 (0.46 – 1.00) | **<0.001** |
| Female | |  |  | -4.63 (-6.88 – -2.38) | **<0.001** | -4.58 (-6.97 – -2.18) | **<0.001** | -4.80 (-7.19 – -2.42) | **<0.001** | -2.44 (-6.71 – 1.83) | 0.262 |
| Diabetes mellitus | |  |  | 3.83 (-0.19 – 7.84) | 0.062 | 2.49 (-1.83 – 6.82) | 0.259 | 2.46 (-1.89 – 6.80) | 0.267 | 4.24 (-3.45 – 11.92) | 0.279 |
| Current smoking | |  |  | -2.06 (-4.76 – 0.64) | 0.134 | -2.08 (-4.99 – 0.83) | 0.162 | -2.11 (-5.00 – 0.79) | 0.153 | -1.04 (-6.27 – 4.20) | 0.697 |
| BMI | |  |  | 0.47 (0.23 – 0.70) | **<0.001** | 0.45 (0.20 – 0.70) | **<0.001** | 0.45 (0.20 – 0.70) | **<0.001** | 0.59 (0.16 – 1.01) | **0.007** |
| Additives | **Milk** |  |  |  |  | 0.19 (-2.48 – 2.86) | 0.888 |  |  |  |  |
|  | **Milk + Sugar** |  |  |  |  | 0.04 (-4.52 – 4.60) | 0.985 |  |  |  |  |
|  | **Milk + Sweetener** |  |  |  |  | 0.10 (-6.33 – 6.54) | 0.974 |  |  |  |  |
|  | **Sugar** |  |  |  |  | 3.30 (-6.17 – 12.77) | 0.494 |  |  |  |  |
|  | **Sweetener** |  |  |  |  | 7.05 (-2.01 – 16.11) | 0.127 |  |  |  |  |
|  | **Binary** |  |  |  |  |  |  | 0.32 (-2.23 – 2.86) | 0.808 |  |  |

**Supplementary Table 136. Multivariable linear regression analysis for the association of moderate/high coffee consumption and systolic blood pressure (excluding black and green tea consumption).** Not daily coffee consumption (<1 cups/d) served as the reference group. Adjustment was performed for: A) unadjusted; B) main cardiovascular risk factors; C) additional adjustment for additives (separately), D) additional adjustment for additives (binary), E) same adjustment as for group A, but only for subjects who consumed coffee without any additives. Abbreviations as in Table 1

|  | | A)DBP | | B)DBP | | C)DBP | | D)DBP | | E)DBP | |
| --- | --- | --- | --- | --- | --- | --- | --- | --- | --- | --- | --- |
|  | | *Estimates* | *p* | *Estimates* | *p* | *Estimates* | *p* | *Estimates* | *p* | *Estimates* | *p* |
| Coffee consumption 3-4 cups/day | | -1.07 (-2.46 – 0.32) | 0.133 | -0.57 (-2.03 – 0.89) | 0.443 | -0.91 (-2.43 – 0.62) | 0.244 | -1.03 (-2.56 – 0.49) | 0.184 | -1.81 (-4.57 – 0.94) | 0.196 |
| Coffee consumption >4 cups/day | | -0.48 (-2.39 – 1.42) | 0.620 | -0.88 (-2.91 – 1.16) | 0.399 | -1.60 (-3.77 – 0.57) | 0.148 | -1.68 (-3.85 – 0.50) | 0.130 | -2.91 (-6.38 – 0.56) | 0.100 |
| Age | |  |  | 0.05 (-0.02 – 0.13) | 0.159 | 0.04 (-0.04 – 0.12) | 0.339 | 0.04 (-0.04 – 0.12) | 0.317 | 0.00 (-0.15 – 0.16) | 0.970 |
| Female | |  |  | -2.63 (-3.90 – -1.35) | **<0.001** | -2.77 (-4.12 – -1.41) | **<0.001** | -2.81 (-4.16 – -1.46) | **<0.001** | -3.00 (-5.42 – -0.58) | **0.015** |
| Diabetes mellitus | |  |  | -0.03 (-2.31 – 2.25) | 0.979 | 0.21 (-2.24 – 2.65) | 0.867 | 0.29 (-2.17 – 2.75) | 0.818 | 2.89 (-1.47 – 7.26) | 0.193 |
| Current smoking | |  |  | -1.73 (-3.26 – -0.20) | **0.027** | -1.53 (-3.17 – 0.12) | 0.069 | -1.46 (-3.10 – 0.18) | 0.081 | -2.24 (-5.22 – 0.74) | 0.141 |
| BMI | |  |  | 0.37 (0.23 – 0.50) | **<0.001** | 0.35 (0.20 – 0.49) | **<0.001** | 0.33 (0.19 – 0.48) | **<0.001** | 0.23 (-0.00 – 0.47) | 0.053 |
| Additives | **Milk** |  |  |  |  | 0.36 (-1.15 – 1.87) | 0.637 |  |  |  |  |
|  | **Milk + Sugar** |  |  |  |  | 0.55 (-2.03 – 3.13) | 0.674 |  |  |  |  |
|  | **Milk + Sweetener** |  |  |  |  | -0.14 (-3.78 – 3.50) | 0.940 |  |  |  |  |
|  | **Sugar** |  |  |  |  | 0.37 (-4.99 – 5.72) | 0.893 |  |  |  |  |
|  | **Sweetener** |  |  |  |  | 1.50 (-3.62 – 6.63) | 0.565 |  |  |  |  |
|  | **Binary** |  |  |  |  |  |  | 0.35 (-1.10 – 1.79) | 0.638 |  |  |

**Supplementary Table 137. Multivariable linear regression analysis for the association of moderate/high coffee consumption and diastolic blood pressure (excluding black and green tea consumption).** Not daily coffee consumption (<1 cups/d) served as the reference group. Adjustment was performed for: A) unadjusted; B) main cardiovascular risk factors; C) additional adjustment for additives (separately), D) additional adjustment for additives (binary), E) same adjustment as for group A, but only for subjects who consumed coffee without any additives. Abbreviations as in Table 1

|  | | A)Heart rate | | B)Heart rate | | C)Heart rate | | D)Heart rate | | E)Heart rate | |
| --- | --- | --- | --- | --- | --- | --- | --- | --- | --- | --- | --- |
|  | | *Estimates* | *p* | *Estimates* | *p* | *Estimates* | *p* | *Estimates* | *p* | *Estimates* | *p* |
| Coffee consumption 3-4 cups/day | | -0.40 (-1.72 – 0.93) | 0.557 | -0.21 (-1.60 – 1.19) | 0.771 | -0.18 (-1.66 – 1.29) | 0.808 | -0.39 (-1.87 – 1.09) | 0.603 | -0.22 (-2.89 – 2.46) | 0.872 |
| Coffee consumption >4 cups/day | | 0.02 (-1.79 – 1.83) | 0.985 | 0.25 (-1.70 – 2.20) | 0.800 | 0.98 (-1.11 – 3.08) | 0.357 | 0.79 (-1.31 – 2.89) | 0.462 | 2.29 (-1.09 – 5.67) | 0.184 |
| Age | |  |  | 0.04 (-0.03 – 0.12) | 0.254 | 0.05 (-0.03 – 0.14) | 0.209 | 0.05 (-0.03 – 0.13) | 0.225 | 0.01 (-0.15 – 0.16) | 0.921 |
| Female | |  |  | 2.75 (1.53 – 3.98) | **<0.001** | 2.99 (1.68 – 4.30) | **<0.001** | 3.00 (1.69 – 4.31) | **<0.001** | 5.14 (2.78 – 7.49) | **<0.001** |
| Diabetes mellitus | |  |  | 2.86 (0.69 – 5.02) | **0.010** | 3.19 (0.85 – 5.53) | **0.008** | 3.48 (1.12 – 5.83) | **0.004** | 2.55 (-1.63 – 6.73) | 0.231 |
| Arterial hypertension | |  |  | 1.51 (0.07 – 2.95) | **0.041** | 1.37 (-0.16 – 2.91) | 0.080 | 1.31 (-0.23 – 2.85) | 0.096 | 1.88 (-0.87 – 4.62) | 0.179 |
| Current smoking | |  |  | 1.28 (-0.19 – 2.74) | 0.087 | 1.13 (-0.46 – 2.72) | 0.163 | 1.43 (-0.16 – 3.01) | 0.078 | 1.45 (-1.43 – 4.34) | 0.323 |
| BMI | |  |  | 0.32 (0.19 – 0.46) | **<0.001** | 0.35 (0.21 – 0.49) | **<0.001** | 0.36 (0.21 – 0.50) | **<0.001** | 0.51 (0.26 – 0.75) | **<0.001** |
| Additives | **Milk** |  |  |  |  | 0.84 (-0.62 – 2.29) | 0.260 |  |  |  |  |
|  | **Milk + Sugar** |  |  |  |  | 1.29 (-1.19 – 3.78) | 0.307 |  |  |  |  |
|  | **Milk + Sweetener** |  |  |  |  | 4.66 (1.16 – 8.17) | **0.009** |  |  |  |  |
|  | **Sugar** |  |  |  |  | 5.82 (0.66 – 10.98) | **0.027** |  |  |  |  |
|  | **Sweetener** |  |  |  |  | 3.61 (-1.32 – 8.55) | 0.151 |  |  |  |  |
|  | **Binary** |  |  |  |  |  |  | 1.21 (-0.18 – 2.60) | 0.089 |  |  |

**Supplementary Table 138. Multivariable linear regression analysis for the association of mild/moderate/high coffee consumption and heart rate (excluding black and green tea consumption).** Not daily coffee consumption (<1 cups/d) served as the reference group. Adjustment was performed for: A) unadjusted; B) main cardiovascular risk factors; C) additional adjustment for additives (separately), D) additional adjustment for additives (binary), E) same adjustment as for group A, but only for subjects who consumed coffee without any additives. Abbreviations as in Table 1.

|  | | A)PQ interval | | B)PQ interval | | C)PQ interval | | D)PQ interval | | E)PQ interval | |
| --- | --- | --- | --- | --- | --- | --- | --- | --- | --- | --- | --- |
|  | | *Estimates* | *p* | *Estimates* | *p* | *Estimates* | *p* | *Estimates* | *p* | *Estimates* | *p* |
| Coffee consumption 3-4 cups/day | | -3.00 (-6.37 – 0.37) | 0.081 | -1.72 (-5.30 – 1.86) | 0.345 | -1.07 (-4.83 – 2.70) | 0.578 | -1.00 (-4.80 – 2.79) | 0.604 | -6.20 (-13.15 – 0.75) | 0.080 |
| Coffee consumption >4 cups/day | | -1.06 (-5.64 – 3.51) | 0.648 | -0.47 (-5.49 – 4.56) | 0.856 | 0.18 (-5.24 – 5.59) | 0.949 | 0.01 (-5.45 – 5.48) | 0.996 | -3.13 (-12.16 – 5.91) | 0.496 |
| Age | |  |  | 0.62 (0.42 – 0.81) | **<0.001** | 0.66 (0.44 – 0.87) | **<0.001** | 0.65 (0.44 – 0.86) | **<0.001** | 1.00 (0.60 – 1.40) | **<0.001** |
| Female | |  |  | -8.45 (-11.59 – -5.32) | **<0.001** | -8.35 (-11.70 – -5.01) | **<0.001** | -8.56 (-11.93 – -5.19) | **<0.001** | -11.65 (-17.76 – -5.54) | **<0.001** |
| Diabetes mellitus | |  |  | 5.24 (-0.34 – 10.82) | 0.066 | 3.15 (-2.90 – 9.20) | 0.308 | 2.81 (-3.33 – 8.96) | 0.369 | 7.45 (-3.54 – 18.45) | 0.183 |
| Arterial hypertension | |  |  | -1.07 (-4.74 – 2.60) | 0.566 | -0.91 (-4.83 – 3.02) | 0.650 | -1.22 (-5.18 – 2.73) | 0.544 | -2.94 (-9.99 – 4.11) | 0.413 |
| Current smoking | |  |  | -1.73 (-5.47 – 2.01) | 0.365 | -1.16 (-5.23 – 2.91) | 0.576 | -1.49 (-5.58 – 2.60) | 0.476 | -1.80 (-9.26 – 5.66) | 0.635 |
| BMI | |  |  | 0.29 (-0.05 – 0.63) | 0.097 | 0.38 (0.02 – 0.75) | **0.040** | 0.37 (0.01 – 0.74) | **0.047** | -0.01 (-0.62 – 0.61) | 0.986 |
| Additives | **Milk** |  |  |  |  | 0.13 (-3.61 – 3.86) | 0.947 |  |  |  |  |
|  | **Milk + Sugar** |  |  |  |  | 1.79 (-4.55 – 8.14) | 0.579 |  |  |  |  |
|  | **Milk + Sweetener** |  |  |  |  | -8.89 (-17.77 – -0.01) | **0.050** |  |  |  |  |
|  | **Sugar** |  |  |  |  | -3.67 (-16.77 – 9.44) | 0.583 |  |  |  |  |
|  | **Sweetener** |  |  |  |  | -6.78 (-18.70 – 5.14) | 0.265 |  |  |  |  |
|  | **Binary** |  |  |  |  |  |  | -0.37 (-3.97 – 3.22) | 0.839 |  |  |

**Supplementary Table 139. Multivariable linear regression analysis for the association of mild/moderate/high coffee consumption and PQ interval (excluding black and green tea consumption).** Not daily coffee consumption (<1 cups/d) served as the reference group. Adjustment was performed for: A) unadjusted; B) main cardiovascular risk factors; C) additional adjustment for additives (separately), D) additional adjustment for additives (binary), E) same adjustment as for group A, but only for subjects who consumed coffee without any additives. Abbreviations as in Table 1.

|  | | A)QRS interval | | B)QRS interval | | C)QRS interval | | D)QRS interval | | E)QRS interval | |
| --- | --- | --- | --- | --- | --- | --- | --- | --- | --- | --- | --- |
|  | | *Estimates* | *p* | *Estimates* | *p* | *Estimates* | *p* | *Estimates* | *p* | *Estimates* | *p* |
| Coffee consumption 3-4 cups/day | | 0.35 (-1.23 – 1.94) | 0.662 | 0.65 (-0.98 – 2.28) | 0.431 | 0.42 (-1.30 – 2.14) | 0.633 | 0.43 (-1.30 – 2.15) | 0.627 | 2.79 (-0.44 – 6.02) | 0.091 |
| Coffee consumption >4 cups/day | | 0.58 (-1.60 – 2.75) | 0.603 | -1.25 (-3.57 – 1.07) | 0.292 | -1.73 (-4.23 – 0.77) | 0.175 | -2.08 (-4.58 – 0.43) | 0.105 | -0.48 (-4.78 – 3.82) | 0.826 |
| Age | |  |  | 0.09 (-0.00 – 0.18) | 0.051 | 0.07 (-0.03 – 0.17) | 0.163 | 0.08 (-0.02 – 0.17) | 0.110 | 0.15 (-0.04 – 0.34) | 0.112 |
| Female | |  |  | -7.99 (-9.43 – -6.56) | **<0.001** | -8.49 (-10.02 – -6.96) | **<0.001** | -8.40 (-9.93 – -6.87) | **<0.001** | -10.58 (-13.45 – -7.71) | **<0.001** |
| Diabetes mellitus | |  |  | -1.90 (-4.43 – 0.64) | 0.142 | -1.68 (-4.43 – 1.06) | 0.229 | -1.47 (-4.24 – 1.30) | 0.297 | -2.58 (-7.62 – 2.45) | 0.313 |
| Arterial hypertension | |  |  | 0.65 (-1.04 – 2.34) | 0.452 | 0.35 (-1.46 – 2.15) | 0.707 | 0.22 (-1.59 – 2.03) | 0.815 | -1.84 (-5.20 – 1.51) | 0.281 |
| Current smoking | |  |  | -0.30 (-2.02 – 1.42) | 0.733 | 0.27 (-1.60 – 2.14) | 0.778 | 0.18 (-1.69 – 2.05) | 0.851 | -0.83 (-4.39 – 2.73) | 0.647 |
| BMI | |  |  | 0.11 (-0.05 – 0.27) | 0.167 | 0.08 (-0.08 – 0.25) | 0.330 | 0.08 (-0.09 – 0.24) | 0.368 | -0.03 (-0.32 – 0.26) | 0.843 |
| Additives | **Milk** |  |  |  |  | 0.12 (-1.59 – 1.83) | 0.890 |  |  |  |  |
|  | **Milk + Sugar** |  |  |  |  | -1.93 (-4.85 – 0.99) | 0.195 |  |  |  |  |
|  | **Milk + Sweetener** |  |  |  |  | -3.11 (-7.17 – 0.95) | 0.133 |  |  |  |  |
|  | **Sugar** |  |  |  |  | -2.58 (-8.64 – 3.49) | 0.405 |  |  |  |  |
|  | **Sweetener** |  |  |  |  | 3.88 (-1.63 – 9.39) | 0.168 |  |  |  |  |
|  | **Binary** |  |  |  |  |  |  | -0.32 (-1.96 – 1.31) | 0.698 |  |  |

**Supplementary Table 140. Multivariable linear regression analysis for the association of mild/moderate/high coffee consumption and QRS interval (excluding black and green tea consumption).** Not daily coffee consumption (<1 cups/d) served as the reference group. Adjustment was performed for: A) unadjusted; B) main cardiovascular risk factors; C) additional adjustment for additives (separately), D) additional adjustment for additives (binary), E) same adjustment as for group A, but only for subjects who consumed coffee without any additives. Abbreviations as in Table 1.

|  | | A)QTc interval | | B)QTc interval | | C)QTc interval | | D)QTc interval | | E)QTc interval | |
| --- | --- | --- | --- | --- | --- | --- | --- | --- | --- | --- | --- |
|  | | *Estimates* | *p* | *Estimates* | *p* | *Estimates* | *p* | *Estimates* | *p* | *Estimates* | *p* |
| Coffee consumption 3-4 cups/day | | -6.16 (-9.25 – -3.07) | **<0.001** | -4.81 (-8.16 – -1.46) | **0.005** | -5.98 (-9.55 – -2.41) | **0.001** | -6.08 (-9.66 – -2.50) | **0.001** | -3.17 (-10.97 – 4.63) | 0.424 |
| Coffee consumption >4 cups/day | | -3.88 (-8.18 – 0.42) | 0.077 | -2.30 (-7.11 – 2.52) | 0.350 | -1.26 (-6.47 – 3.95) | 0.634 | -2.12 (-7.35 – 3.12) | 0.428 | 2.31 (-8.10 – 12.71) | 0.663 |
| Age | |  |  | 0.27 (0.09 – 0.45) | **0.004** | 0.24 (0.04 – 0.44) | **0.019** | 0.22 (0.02 – 0.42) | **0.034** | 0.04 (-0.42 – 0.49) | 0.867 |
| Female | |  |  | 5.42 (2.47 – 8.37) | **<0.001** | 5.02 (1.84 – 8.19) | **0.002** | 5.00 (1.82 – 8.19) | **0.002** | 8.65 (1.73 – 15.58) | **0.015** |
| Diabetes mellitus | |  |  | 4.93 (-0.30 – 10.17) | 0.065 | 5.29 (-0.45 – 11.02) | 0.071 | 6.20 (0.41 – 12.00) | **0.036** | 9.00 (-3.16 – 21.16) | 0.146 |
| Arterial hypertension | |  |  | 2.91 (-0.55 – 6.37) | 0.099 | 3.01 (-0.72 – 6.74) | 0.113 | 3.01 (-0.73 – 6.75) | 0.115 | 2.50 (-5.61 – 10.61) | 0.544 |
| Current smoking | |  |  | 2.55 (-1.02 – 6.11) | 0.162 | 2.06 (-1.84 – 5.97) | 0.300 | 2.89 (-1.02 – 6.80) | 0.147 | 3.68 (-4.84 – 12.19) | 0.396 |
| BMI | |  |  | 0.37 (0.05 – 0.69) | **0.025** | 0.35 (0.00 – 0.70) | **0.048** | 0.36 (0.01 – 0.71) | **0.042** | 0.61 (-0.10 – 1.33) | 0.093 |
| Additives | **Milk** |  |  |  |  | 2.23 (-1.31 – 5.77) | 0.217 |  |  |  |  |
|  | **Milk + Sugar** |  |  |  |  | 6.50 (0.49 – 12.52) | **0.034** |  |  |  |  |
|  | **Milk + Sweetener** |  |  |  |  | 5.23 (-3.38 – 13.84) | 0.234 |  |  |  |  |
|  | **Sugar** |  |  |  |  | 6.19 (-6.45 – 18.84) | 0.337 |  |  |  |  |
|  | **Sweetener** |  |  |  |  | 4.17 (-7.55 – 15.88) | 0.485 |  |  |  |  |
|  | **Binary** |  |  |  |  |  |  | 3.08 (-0.32 – 6.47) | 0.076 |  |  |

**Supplementary Table 141. Multivariable linear regression analysis for the association of mild/moderate/high coffee consumption and QTc interval (excluding black and green tea consumption).** Not daily coffee consumption (<1 cups/d) served as the reference group. Adjustment was performed for: A) unadjusted; B) main cardiovascular risk factors; C) additional adjustment for additives (separately), D) additional adjustment for additives (binary), E) same adjustment as for group A, but only for subjects who consumed coffee without any additives. Abbreviations as in Table 1.

|  | | A)LVEF | | B)LVEF | | C)LVEF | | D)LVEF | | E)LVEF | |
| --- | --- | --- | --- | --- | --- | --- | --- | --- | --- | --- | --- |
|  | | *Estimates* | *p* | *Estimates* | *p* | *Estimates* | *p* | *Estimates* | *p* | *Estimates* | *p* |
| Coffee consumption 3-4 cups/day | | 0.44 (-0.26 – 1.14) | 0.215 | 0.59 (-0.16 – 1.34) | 0.121 | 0.47 (-0.33 – 1.27) | 0.247 | 0.45 (-0.35 – 1.25) | 0.268 | 0.81 (-0.65 – 2.26) | 0.276 |
| Coffee consumption >4 cups/day | | -0.01 (-0.94 – 0.92) | 0.976 | 0.43 (-0.60 – 1.46) | 0.415 | 0.12 (-1.01 – 1.24) | 0.839 | 0.18 (-0.94 – 1.31) | 0.748 | -1.77 (-3.65 – 0.11) | 0.065 |
| Age | |  |  | 0.02 (-0.02 – 0.07) | 0.241 | 0.01 (-0.03 – 0.06) | 0.631 | 0.01 (-0.03 – 0.06) | 0.572 | 0.04 (-0.04 – 0.13) | 0.334 |
| Female | |  |  | 1.59 (0.94 – 2.24) | **<0.001** | 1.36 (0.66 – 2.06) | **<0.001** | 1.38 (0.68 – 2.08) | **<0.001** | 1.02 (-0.25 – 2.28) | 0.114 |
| Diabetes mellitus | |  |  | -0.52 (-1.74 – 0.71) | 0.407 | -0.37 (-1.70 – 0.96) | 0.584 | -0.48 (-1.83 – 0.86) | 0.480 | -1.93 (-4.42 – 0.56) | 0.128 |
| Arterial hypertension | |  |  | -0.71 (-1.46 – 0.05) | 0.067 | -0.55 (-1.36 – 0.27) | 0.189 | -0.54 (-1.35 – 0.28) | 0.197 | -0.08 (-1.53 – 1.37) | 0.915 |
| Current smoking | |  |  | -0.11 (-0.89 – 0.68) | 0.786 | -0.14 (-1.01 – 0.72) | 0.743 | -0.18 (-1.03 – 0.68) | 0.687 | -0.54 (-2.18 – 1.09) | 0.515 |
| BMI | |  |  | -0.13 (-0.21 – -0.06) | **0.001** | -0.11 (-0.19 – -0.03) | **0.008** | -0.11 (-0.18 – -0.03) | **0.010** | -0.15 (-0.29 – -0.00) | **0.045** |
| Additives | **Milk** |  |  |  |  | 0.14 (-0.64 – 0.92) | 0.725 |  |  |  |  |
|  | **Milk + Sugar** |  |  |  |  | -0.22 (-1.58 – 1.14) | 0.749 |  |  |  |  |
|  | **Milk + Sweetener** |  |  |  |  | 0.11 (-1.80 – 2.02) | 0.910 |  |  |  |  |
|  | **Sugar** |  |  |  |  | -0.26 (-2.90 – 2.38) | 0.844 |  |  |  |  |
|  | **Sweetener** |  |  |  |  | -0.19 (-3.41 – 3.03) | 0.907 |  |  |  |  |
|  | **Binary** |  |  |  |  |  |  | 0.10 (-0.65 – 0.84) | 0.798 |  |  |

**Supplementary Table 142. Multivariable linear regression analysis for the association of mild/moderate/high coffee consumption and LVEF (excluding black and green tea consumption).** Not daily coffee consumption (<1 cups/d) served as the reference group. Adjustment was performed for: A) unadjusted; B) main cardiovascular risk factors; C) additional adjustment for additives (separately), D) additional adjustment for additives (binary), E) same adjustment as for group A, but only for subjects who consumed coffee without any additives. Abbreviations as in Table 1.

|  | | A)LV mass index | | B)LV mass index | | C)LV mass index | | D)LV mass index | | E)LV mass index | |
| --- | --- | --- | --- | --- | --- | --- | --- | --- | --- | --- | --- |
|  | | *Estimates* | *p* | *Estimates* | *p* | *Estimates* | *p* | *Estimates* | *p* | *Estimates* | *p* |
| Coffee consumption 3-4 cups/day | | 0.47 (-2.56 – 3.51) | 0.759 | 0.55 (-2.27 – 3.37) | 0.703 | 0.29 (-2.65 – 3.22) | 0.846 | 0.52 (-2.45 – 3.49) | 0.732 | -0.99 (-6.52 – 4.55) | 0.725 |
| Coffee consumption >4 cups/day | | 3.59 (-0.45 – 7.63) | 0.082 | 0.52 (-3.32 – 4.37) | 0.789 | -0.70 (-4.82 – 3.43) | 0.741 | -0.79 (-4.97 – 3.39) | 0.709 | 1.86 (-5.13 – 8.86) | 0.600 |
| Age | |  |  | 0.29 (0.14 – 0.45) | **<0.001** | 0.32 (0.15 – 0.48) | **<0.001** | 0.31 (0.14 – 0.47) | **<0.001** | 0.30 (-0.02 – 0.62) | 0.069 |
| Female | |  |  | -15.29 (-17.77 – -12.82) | **<0.001** | -15.45 (-18.07 – -12.83) | **<0.001** | -15.41 (-18.05 – -12.77) | **<0.001** | -18.15 (-22.93 – -13.36) | **<0.001** |
| Diabetes mellitus | |  |  | 2.38 (-2.25 – 7.01) | 0.313 | 2.65 (-2.34 – 7.64) | 0.298 | 2.43 (-2.65 – 7.52) | 0.348 | 10.84 (0.28 – 21.40) | **0.044** |
| Arterial hypertension | |  |  | 3.09 (0.22 – 5.95) | **0.035** | 3.00 (-0.02 – 6.02) | 0.051 | 2.91 (-0.14 – 5.96) | 0.062 | 0.74 (-4.78 – 6.25) | 0.792 |
| Current smoking | |  |  | 2.70 (-0.26 – 5.66) | 0.073 | 4.38 (1.17 – 7.59) | **0.007** | 3.77 (0.55 – 6.99) | **0.022** | 5.25 (-0.99 – 11.49) | 0.099 |
| BMI | |  |  | 0.78 (0.49 – 1.06) | **<0.001** | 0.71 (0.42 – 1.01) | **<0.001** | 0.70 (0.39 – 1.00) | **<0.001** | 0.55 (0.00 – 1.10) | **0.049** |
| Additives | **Milk** |  |  |  |  | -0.06 (-2.94 – 2.82) | 0.967 |  |  |  |  |
|  | **Milk + Sugar** |  |  |  |  | -0.50 (-5.67 – 4.67) | 0.850 |  |  |  |  |
|  | **Milk + Sweetener** |  |  |  |  | -11.33 (-19.56 – -3.10) | **0.007** |  |  |  |  |
|  | **Sugar** |  |  |  |  | -9.51 (-20.13 – 1.11) | 0.079 |  |  |  |  |
|  | **Sweetener** |  |  |  |  | -7.94 (-17.52 – 1.65) | 0.104 |  |  |  |  |
|  | **Binary** |  |  |  |  |  |  | -0.90 (-3.71 – 1.90) | 0.527 |  |  |

**Supplementary Table 143. Multivariable linear regression analysis for the association of mild/moderate/high coffee consumption and LV mass index (excluding black and green tea consumption).** Not daily coffee consumption (<1 cups/d) served as the reference group. Adjustment was performed for: A) unadjusted; B) main cardiovascular risk factors; C) additional adjustment for additives (separately), D) additional adjustment for additives (binary), E) same adjustment as for group A, but only for subjects who consumed coffee without any additives. Abbreviations as in Table 1.

|  | | A)E/e‘ ratio | | B)E/e‘ ratio | | C)E/e‘ ratio | | D)E/e‘ ratio | | E)E/e‘ ratio | |
| --- | --- | --- | --- | --- | --- | --- | --- | --- | --- | --- | --- |
|  | | *Estimates* | *p* | *Estimates* | *p* | *Estimates* | *p* | *Estimates* | *p* | *Estimates* | *p* |
| Coffee consumption 3-4 cups/day | | -0.43 (-0.71 – -0.15) | **0.003** | -0.29 (-0.57 – 0.00) | 0.050 | -0.33 (-0.64 – -0.03) | **0.033** | -0.31 (-0.62 – -0.01) | **0.046** | -0.34 (-0.96 – 0.27) | 0.275 |
| Coffee consumption >4 cups/day | | -0.49 (-0.88 – -0.10) | **0.013** | -0.37 (-0.77 – 0.04) | 0.076 | -0.31 (-0.76 – 0.14) | 0.171 | -0.30 (-0.75 – 0.15) | 0.190 | -0.06 (-0.89 – 0.76) | 0.877 |
| Age | |  |  | 0.06 (0.04 – 0.07) | **<0.001** | 0.06 (0.04 – 0.07) | **<0.001** | 0.06 (0.04 – 0.07) | **<0.001** | 0.06 (0.02 – 0.10) | **0.001** |
| Female | |  |  | 0.59 (0.34 – 0.84) | **<0.001** | 0.63 (0.36 – 0.91) | **<0.001** | 0.65 (0.37 – 0.92) | **<0.001** | 0.94 (0.40 – 1.48) | **0.001** |
| Diabetes mellitus | |  |  | 0.53 (0.07 – 0.98) | **0.024** | 0.53 (0.02 – 1.04) | **0.040** | 0.55 (0.04 – 1.06) | **0.035** | 0.52 (-0.50 – 1.54) | 0.317 |
| Arterial hypertension | |  |  | 0.52 (0.22 – 0.81) | **0.001** | 0.52 (0.20 – 0.84) | **0.001** | 0.54 (0.22 – 0.86) | **0.001** | 0.57 (-0.05 – 1.20) | 0.072 |
| Current smoking | |  |  | 0.41 (0.11 – 0.71) | **0.008** | 0.46 (0.12 – 0.79) | **0.008** | 0.44 (0.11 – 0.78) | **0.010** | 0.64 (-0.06 – 1.34) | 0.074 |
| BMI | |  |  | 0.04 (0.01 – 0.07) | **0.003** | 0.04 (0.01 – 0.07) | **0.005** | 0.05 (0.02 – 0.08) | **0.003** | 0.10 (0.04 – 0.16) | **0.001** |
| Additives | **Milk** |  |  |  |  | -0.20 (-0.50 – 0.11) | 0.206 |  |  |  |  |
|  | **Milk + Sugar** |  |  |  |  | -0.25 (-0.77 – 0.27) | 0.351 |  |  |  |  |
|  | **Milk + Sweetener** |  |  |  |  | 0.36 (-0.40 – 1.12) | 0.353 |  |  |  |  |
|  | **Sugar** |  |  |  |  | -0.20 (-1.29 – 0.88) | 0.715 |  |  |  |  |
|  | **Sweetener** |  |  |  |  | -0.49 (-1.62 – 0.64) | 0.396 |  |  |  |  |
|  | **Binary** |  |  |  |  |  |  | -0.18 (-0.47 – 0.12) | 0.240 |  |  |

**Supplementary Table 144. Multivariable linear regression analysis for the association of mild/moderate/high coffee consumption and E/e’ ratio (excluding black and green tea consumption).** Not daily coffee consumption (<1 cups/d) served as the reference group. Adjustment was performed for: A) unadjusted; B) main cardiovascular risk factors; C) additional adjustment for additives (separately), D) additional adjustment for additives (binary), E) same adjustment as for group A, but only for subjects who consumed coffee without any additives. Abbreviations as in Table 1.

|  | | A)TR Vmax in m/s | | B)TR Vmax in m/s | | C)TR Vmax in m/s | | D)TR Vmax in m/s | | E)TR Vmax in m/s | |
| --- | --- | --- | --- | --- | --- | --- | --- | --- | --- | --- | --- |
|  | | *Estimates* | *p* | *Estimates* | *p* | *Estimates* | *p* | *Estimates* | *p* | *Estimates* | *p* |
| Coffee consumption 3-4 cups/day | | -0.02 (-0.07 – 0.04) | 0.574 | -0.00 (-0.06 – 0.06) | 0.903 | -0.00 (-0.07 – 0.06) | 0.942 | -0.00 (-0.07 – 0.06) | 0.893 | -0.08 (-0.21 – 0.05) | 0.218 |
| Coffee consumption >4 cups/day | | -0.02 (-0.10 – 0.06) | 0.640 | -0.01 (-0.09 – 0.08) | 0.887 | -0.01 (-0.11 – 0.09) | 0.820 | -0.02 (-0.12 – 0.08) | 0.749 | 0.04 (-0.14 – 0.22) | 0.642 |
| Age | |  |  | 0.01 (0.00 – 0.01) | **0.002** | 0.01 (0.00 – 0.01) | **0.007** | 0.01 (0.00 – 0.01) | **0.004** | 0.00 (-0.00 – 0.01) | 0.434 |
| Female | |  |  | -0.04 (-0.09 – 0.02) | 0.175 | -0.04 (-0.10 – 0.02) | 0.204 | -0.04 (-0.10 – 0.02) | 0.194 | -0.04 (-0.15 – 0.07) | 0.457 |
| Diabetes mellitus | |  |  | -0.03 (-0.12 – 0.07) | 0.608 | -0.02 (-0.12 – 0.08) | 0.703 | -0.03 (-0.14 – 0.07) | 0.517 | -0.20 (-0.46 – 0.05) | 0.112 |
| Arterial hypertension | |  |  | 0.07 (0.00 – 0.13) | **0.038** | 0.06 (-0.01 – 0.13) | 0.090 | 0.06 (-0.01 – 0.13) | 0.069 | 0.12 (-0.00 – 0.25) | 0.056 |
| Current smoking | |  |  | 0.05 (-0.02 – 0.13) | 0.139 | 0.05 (-0.03 – 0.14) | 0.219 | 0.06 (-0.02 – 0.15) | 0.163 | -0.10 (-0.29 – 0.10) | 0.327 |
| BMI | |  |  | 0.00 (-0.01 – 0.01) | 0.789 | 0.00 (-0.00 – 0.01) | 0.363 | 0.00 (-0.00 – 0.01) | 0.493 | 0.01 (-0.01 – 0.02) | 0.418 |
| Additives | **Milk** |  |  |  |  | -0.07 (-0.13 – -0.01) | **0.032** |  |  |  |  |
|  | **Milk + Sugar** |  |  |  |  | -0.09 (-0.20 – 0.03) | 0.135 |  |  |  |  |
|  | **Milk + Sweetener** |  |  |  |  | -0.05 (-0.20 – 0.11) | 0.551 |  |  |  |  |
|  | **Sugar** |  |  |  |  | 0.08 (-0.16 – 0.31) | 0.513 |  |  |  |  |
|  | **Sweetener** |  |  |  |  | 0.17 (-0.10 – 0.45) | 0.209 |  |  |  |  |
|  | **Binary** |  |  |  |  |  |  | -0.07 (-0.13 – -0.00) | **0.035** |  |  |

**Supplementary Table 145. Multivariable linear regression analysis for the association of mild/moderate/high coffee consumption and TR Vmax in m/s (excluding black and green tea consumption).** Not daily coffee consumption (<1 cups/d) served as the reference group. Adjustment was performed for: A) unadjusted; B) main cardiovascular risk factors; C) additional adjustment for additives (separately), D) additional adjustment for additives (binary), E) same adjustment as for group A, but only for subjects who consumed coffee without any additives. Abbreviations as in Table 1.

|  | | A)TAPSE in mm | | B)TAPSE in mm | | C)TAPSE in mm | | D)TAPSE in mm | | E)TAPSE in mm | |
| --- | --- | --- | --- | --- | --- | --- | --- | --- | --- | --- | --- |
|  | | *Estimates* | *p* | *Estimates* | *p* | *Estimates* | *p* | *Estimates* | *p* | *Estimates* | *p* |
| Coffee consumption 3-4 cups/day | | 0.64 (-0.02 – 1.30) | 0.057 | 0.50 (-0.23 – 1.23) | 0.176 | 0.46 (-0.31 – 1.23) | 0.244 | 0.43 (-0.35 – 1.20) | 0.282 | 0.33 (-1.05 – 1.72) | 0.636 |
| Coffee consumption >4 cups/day | | 0.92 (0.06 – 1.79) | **0.037** | 0.60 (-0.37 – 1.57) | 0.223 | 0.40 (-0.66 – 1.46) | 0.455 | 0.40 (-0.66 – 1.46) | 0.457 | 0.71 (-0.98 – 2.40) | 0.406 |
| Age | |  |  | -0.09 (-0.13 – -0.05) | **<0.001** | -0.10 (-0.15 – -0.06) | **<0.001** | -0.10 (-0.14 – -0.06) | **<0.001** | -0.15 (-0.23 – -0.07) | **<0.001** |
| Female | |  |  | -0.53 (-1.18 – 0.11) | 0.104 | -0.58 (-1.27 – 0.12) | 0.102 | -0.57 (-1.26 – 0.13) | 0.109 | -1.31 (-2.50 – -0.12) | **0.031** |
| Diabetes mellitus | |  |  | -1.25 (-2.47 – -0.02) | **0.046** | -1.31 (-2.67 – 0.05) | 0.059 | -1.29 (-2.67 – 0.08) | 0.065 | -0.90 (-3.36 – 1.55) | 0.468 |
| Arterial hypertension | |  |  | -0.01 (-0.75 – 0.73) | 0.977 | -0.09 (-0.89 – 0.71) | 0.826 | -0.13 (-0.92 – 0.67) | 0.757 | -0.17 (-1.53 – 1.20) | 0.811 |
| Current smoking | |  |  | -0.19 (-0.96 – 0.58) | 0.633 | 0.08 (-0.77 – 0.93) | 0.850 | 0.07 (-0.77 – 0.91) | 0.872 | -0.54 (-2.12 – 1.04) | 0.501 |
| BMI | |  |  | 0.03 (-0.04 – 0.10) | 0.408 | 0.05 (-0.03 – 0.13) | 0.257 | 0.05 (-0.03 – 0.12) | 0.264 | 0.07 (-0.07 – 0.21) | 0.330 |
| Additives | **Milk** |  |  |  |  | -0.25 (-1.01 – 0.51) | 0.519 |  |  |  |  |
|  | **Milk + Sugar** |  |  |  |  | -0.64 (-2.00 – 0.72) | 0.354 |  |  |  |  |
|  | **Milk + Sweetener** |  |  |  |  | -0.32 (-2.34 – 1.71) | 0.760 |  |  |  |  |
|  | **Sugar** |  |  |  |  | -0.38 (-2.80 – 2.05) | 0.761 |  |  |  |  |
|  | **Sweetener** |  |  |  |  | 0.14 (-2.51 – 2.79) | 0.918 |  |  |  |  |
|  | **Binary** |  |  |  |  |  |  | -0.29 (-1.03 – 0.44) | 0.434 |  |  |

**Supplementary Table 146. Multivariable linear regression analysis for the association of mild/moderate/high coffee consumption and TAPSE in mm (excluding black and green tea consumption).** Not daily coffee consumption (<1 cups/d) served as the reference group. Adjustment was performed for: A) unadjusted; B) main cardiovascular risk factors; C) additional adjustment for additives (separately), D) additional adjustment for additives (binary), E) same adjustment as for group A, but only for subjects who consumed coffee without any additives. Abbreviations as in Table 1.

|  | | A)LASV in ml | | B)LASV in m | | C)LASV in ml | | D)LASV in ml | | E)LASV in ml | |
| --- | --- | --- | --- | --- | --- | --- | --- | --- | --- | --- | --- |
|  | | *Estimates* | *p* | *Estimates* | *p* | *Estimates* | *p* | *Estimates* | *p* | *Estimates* | *p* |
| Coffee consumption3 -4 cups/day | | 0.31 (-1.00 – 1.63) | 0.641 | 0.78 (-0.59 – 2.15) | 0.266 | 0.87 (-0.60 – 2.33) | 0.245 | 0.92 (-0.56 – 2.39) | 0.221 | 2.62 (-0.46 – 5.71) | 0.095 |
| Coffee consumption >4 cups/day | | -0.15 (-1.92 – 1.62) | 0.869 | -0.09 (-1.98 – 1.80) | 0.927 | -0.00 (-2.07 – 2.07) | 1.000 | -0.07 (-2.14 – 2.00) | 0.948 | -0.33 (-4.13 – 3.47) | 0.864 |
| Age | |  |  | 0.09 (0.02 – 0.17) | **0.016** | 0.10 (0.02 – 0.18) | **0.017** | 0.10 (0.02 – 0.18) | **0.020** | 0.18 (0.01 – 0.35) | **0.042** |
| Female | |  |  | -2.22 (-3.42 – -1.02) | **<0.001** | -2.34 (-3.65 – -1.03) | **<0.001** | -2.38 (-3.69 – -1.07) | **<0.001** | -1.43 (-4.05 – 1.19) | 0.282 |
| Diabetes mellitus | |  |  | -1.16 (-3.31 – 0.99) | 0.291 | -1.67 (-4.02 – 0.68) | 0.164 | -1.86 (-4.24 – 0.52) | 0.124 | -0.67 (-5.84 – 4.50) | 0.798 |
| Arterial hypertension | |  |  | 0.81 (-0.57 – 2.18) | 0.248 | 0.51 (-0.97 – 2.00) | 0.498 | 0.58 (-0.91 – 2.08) | 0.445 | 0.81 (-2.09 – 3.70) | 0.583 |
| Current smoking | |  |  | -1.18 (-2.63 – 0.27) | 0.110 | -0.76 (-2.38 – 0.85) | 0.353 | -0.84 (-2.44 – 0.76) | 0.302 | 1.19 (-2.10 – 4.48) | 0.476 |
| BMI | |  |  | 0.18 (0.04 – 0.32) | **0.010** | 0.18 (0.03 – 0.33) | **0.018** | 0.17 (0.02 – 0.32) | **0.025** | 0.02 (-0.27 – 0.30) | 0.896 |
| Additives | **Milk** |  |  |  |  | -0.12 (-1.56 – 1.32) | 0.865 |  |  |  |  |
|  | **Milk + Sugar** |  |  |  |  | 0.28 (-2.32 – 2.88) | 0.832 |  |  |  |  |
|  | **Milk + Sweetener** |  |  |  |  | -3.97 (-7.69 – -0.25) | **0.037** |  |  |  |  |
|  | **Sugar** |  |  |  |  | 1.44 (-3.76 – 6.64) | 0.587 |  |  |  |  |
|  | **Sweetener** |  |  |  |  | -1.21 (-6.72 – 4.29) | 0.666 |  |  |  |  |
|  | **Binary** |  |  |  |  |  |  | -0.22 (-1.62 – 1.17) | 0.755 |  |  |

**Supplementary Table 147. Multivariable linear regression analysis for the association of mild/moderate/high coffee consumption and LASV in ml (excluding black and green tea consumption).** Not daily coffee consumption (<1 cups/d) served as the reference group. Adjustment was performed for: A) unadjusted; B) main cardiovascular risk factors; C) additional adjustment for additives (separately), D) additional adjustment for additives (binary), E) same adjustment as for group A, but only for subjects who consumed coffee without any additives. Abbreviations as in Table 1.

|  | | A)LBB | | B)LBB | | C)LBB | | D)LBB | | E)LBB | |
| --- | --- | --- | --- | --- | --- | --- | --- | --- | --- | --- | --- |
|  | | *Odds Ratios* | *p* | *Odds Ratios* | *p* | *Odds Ratios* | *p* | *Odds Ratios* | *p* | *Odds Ratios* | *p* |
| Coffee consumption 3-4 cups/day | | 0.92 (0.45 – 1.80) | 0.818 | 1.02 (0.45 – 2.15) | 0.955 | 1.07 (0.44 – 2.41) | 0.880 | 0.91 (0.38 – 2.01) | 0.821 | 1.69 (0.38 – 7.52) | 0.477 |
| Coffee consumption >4 cups/day | | 1.83 (0.83 – 3.74) | 0.112 | 1.55 (0.59 – 3.69) | 0.341 | 1.25 (0.39 – 3.42) | 0.683 | 1.29 (0.44 – 3.29) | 0.609 | 3.21 (0.67 – 15.23) | 0.132 |
| Age | |  |  | 1.04 (1.00 – 1.09) | 0.053 | 1.01 (0.96 – 1.06) | 0.762 | 1.03 (0.99 – 1.08) | 0.211 | 1.03 (0.95 – 1.11) | 0.498 |
| Female | |  |  | 0.36 (0.17 – 0.72) | **0.006** | 0.30 (0.12 – 0.67) | **0.006** | 0.26 (0.10 – 0.57) | **0.002** | 0.26 (0.04 – 1.04) | 0.090 |
| Diabetes mellitus | |  |  | 0.41 (0.10 – 1.20) | 0.151 | 0.66 (0.15 – 2.04) | 0.517 | 0.53 (0.12 – 1.59) | 0.311 |  |  |
| Arterial hypertension | |  |  | 1.87 (0.77 – 5.27) | 0.192 | 1.87 (0.70 – 5.97) | 0.243 | 1.53 (0.61 – 4.40) | 0.388 | 1.72 (0.35 – 12.78) | 0.535 |
| Current smoking | |  |  | 0.78 (0.31 – 1.75) | 0.577 | 0.94 (0.34 – 2.29) | 0.906 | 0.94 (0.36 – 2.15) | 0.884 | 1.69 (0.41 – 6.08) | 0.434 |
| BMI | |  |  | 1.06 (0.99 – 1.13) | 0.080 | 1.07 (1.00 – 1.15) | **0.050** | 1.07 (0.99 – 1.14) | 0.058 | 1.07 (0.96 – 1.17) | 0.172 |
| Additives | **Milk** |  |  |  |  | 1.06 (0.51 – 2.30) | 0.885 |  |  |  |  |
|  | **Binary** |  |  |  |  |  |  | 0.95 (0.47 – 2.02) | 0.895 |  |  |

**Supplementary Table 148. Multivariable logistic regression analysis for the association of mild/moderate/high coffee consumption and LBBB (excluding black and green tea consumption).** Not daily coffee consumption (<1 cups/d) served as the reference group. Adjustment was performed for: A) unadjusted; B) main cardiovascular risk factors; C) additional adjustment for additives (separately), D) additional adjustment for additives (binary), E) same adjustment as for group A, but only for subjects who consumed coffee without any additives. Abbreviations as in Table 1.

|  | | A)AV_block | | B)AV_block | | C)AV block | | D)AV_block | | E)AV block | |
| --- | --- | --- | --- | --- | --- | --- | --- | --- | --- | --- | --- |
|  | | *Odds Ratios* | *p* | *Odds Ratios* | *p* | *Odds Ratios* | *p* | *Odds Ratios* | *p* | *Odds Ratios* | *p* |
| Coffee consumption 3-4 cups/day | | 0.52 (0.27 – 0.92) | **0.031** | 0.62 (0.30 – 1.16) | 0.154 | 0.64 (0.31 – 1.22) | 0.194 | 0.64 (0.31 – 1.23) | 0.202 | 0.30 (0.07 – 1.04) | 0.084 |
| Coffee consumption >4 cups/day | | 0.77 (0.35 – 1.52) | 0.483 | 1.24 (0.53 – 2.64) | 0.589 | 1.10 (0.42 – 2.56) | 0.832 | 1.15 (0.46 – 2.57) | 0.747 | 0.71 (0.14 – 2.63) | 0.632 |
| Age | |  |  | 1.10 (1.06 – 1.14) | **<0.001** | 1.11 (1.07 – 1.16) | **<0.001** | 1.10 (1.06 – 1.15) | **<0.001** | 1.12 (1.05 – 1.21) | **0.002** |
| Female | |  |  | 0.40 (0.22 – 0.68) | **0.001** | 0.43 (0.24 – 0.76) | **0.005** | 0.41 (0.22 – 0.71) | **0.002** | 0.27 (0.08 – 0.77) | **0.021** |
| Diabetes mellitus | |  |  | 1.68 (0.81 – 3.28) | 0.143 | 1.57 (0.69 – 3.31) | 0.251 | 1.35 (0.60 – 2.81) | 0.437 | 1.49 (0.30 – 5.88) | 0.591 |
| Arterial hypertension | |  |  | 0.63 (0.34 – 1.21) | 0.152 | 0.65 (0.33 – 1.33) | 0.222 | 0.64 (0.33 – 1.28) | 0.199 | 0.25 (0.07 – 0.79) | **0.019** |
| Current smoking | |  |  | 0.70 (0.32 – 1.39) | 0.338 | 0.84 (0.37 – 1.75) | 0.662 | 0.86 (0.39 – 1.73) | 0.686 | 1.38 (0.35 – 4.50) | 0.614 |
| BMI | |  |  | 1.00 (0.94 – 1.06) | 0.928 | 1.00 (0.93 – 1.06) | 0.999 | 1.00 (0.94 – 1.06) | 0.965 | 1.02 (0.91 – 1.12) | 0.694 |
| Additives | **Milk** |  |  |  |  | 0.96 (0.54 – 1.76) | 0.889 |  |  |  |  |
|  | **Milk + Sugar** |  |  |  |  | 1.74 (0.64 – 4.30) | 0.251 |  |  |  |  |
|  | **Binary** |  |  |  |  |  |  | 0.98 (0.56 – 1.75) | 0.938 |  |  |

**Supplementary Table 149. Multivariable logistic regression analysis for the association of mild/moderate/high coffee consumption and AV block (excluding black and green tea consumption).** Not daily coffee consumption (<1 cups/d) served as the reference group. Adjustment was performed for: A) unadjusted; B) main cardiovascular risk factors; C) additional adjustment for additives (separately), D) additional adjustment for additives (binary), E) same adjustment as for group A, but only for subjects who consumed coffee without any additives. Abbreviations as in Table 1.

|  | | A)Atrial_fibrillation | | B)Atrial_fibrillation | | C)Atrial_fibrillation | | D)Atrial_fibrillation | | E)Atrial_fibrillation | |
| --- | --- | --- | --- | --- | --- | --- | --- | --- | --- | --- | --- |
|  | | *Odds Ratios* | *p* | *Odds Ratios* | *p* | *Odds Ratios* | *p* | *Odds Ratios* | *p* | *Odds Ratios* | *p* |
| Coffee consumption 3-4 cups/day | | 1.13 (0.69 – 1.81) | 0.612 | 1.73 (0.99 – 3.00) | 0.052 | 1.27 (0.68 – 2.30) | 0.442 | 1.41 (0.77 – 2.51) | 0.256 | 0.93 (0.34 – 2.39) | 0.880 |
| Coffee consumption >4 cups/day | | 0.65 (0.27 – 1.37) | 0.298 | 0.86 (0.31 – 2.04) | 0.743 | 0.64 (0.21 – 1.64) | 0.393 | 0.79 (0.28 – 1.92) | 0.625 | 0.78 (0.16 – 2.85) | 0.727 |
| Age | |  |  | 1.11 (1.07 – 1.16) | **<0.001** | 1.11 (1.07 – 1.16) | **<0.001** | 1.11 (1.07 – 1.16) | **<0.001** | 1.16 (1.08 – 1.25) | **<0.001** |
| Female | |  |  | 0.53 (0.30 – 0.89) | **0.019** | 0.48 (0.26 – 0.83) | **0.011** | 0.46 (0.26 – 0.80) | **0.008** | 0.28 (0.10 – 0.71) | **0.011** |
| Diabetes mellitus | |  |  | 1.29 (0.66 – 2.40) | 0.442 | 1.20 (0.57 – 2.37) | 0.611 | 1.35 (0.66 – 2.59) | 0.391 | 1.39 (0.43 – 4.14) | 0.564 |
| Arterial hypertension | |  |  | 2.95 (1.23 – 8.78) | **0.027** | 2.38 (0.97 – 7.14) | 0.082 | 2.56 (1.05 – 7.65) | 0.059 |  |  |
| Current smoking | |  |  | 0.96 (0.47 – 1.84) | 0.917 | 1.00 (0.45 – 2.03) | 0.992 | 1.04 (0.49 – 2.06) | 0.912 | 0.96 (0.25 – 2.96) | 0.941 |
| BMI | |  |  | 1.07 (1.01 – 1.12) | **0.011** | 1.07 (1.01 – 1.13) | **0.013** | 1.07 (1.01 – 1.13) | **0.011** | 1.05 (0.96 – 1.13) | 0.244 |
| Additives | **Milk** |  |  |  |  | 0.67 (0.38 – 1.18) | 0.164 |  |  |  |  |
|  | **Milk + Sweetener** |  |  |  |  | 0.59 (0.09 – 2.22) | 0.497 |  |  |  |  |
|  | **Sweetener** |  |  |  |  | 0.93 (0.13 – 3.89) | 0.931 |  |  |  |  |
|  | **Binary** |  |  |  |  |  |  | 0.66 (0.39 – 1.15) | 0.136 |  |  |

**Supplementary Table 150. Multivariable logistic regression analysis for the association of mild/moderate/high coffee consumption and atrial fibrillation (excluding black and green tea consumption).** Not daily coffee consumption (<1 cups/d) served as the reference group. Adjustment was performed for: A) unadjusted; B) main cardiovascular risk factors; C) additional adjustment for additives (separately), D) additional adjustment for additives (binary), E) same adjustment as for group A, but only for subjects who consumed coffee without any additives. Abbreviations as in Table 1.

|  | | A)Diabetes | | B)Diabetes | | C)Diabetes | | D)Diabetes | | E)Diabetes | |
| --- | --- | --- | --- | --- | --- | --- | --- | --- | --- | --- | --- |
|  | | *Odds Ratios* | *p* | *Odds Ratios* | *p* | *Odds Ratios* | *p* | *Odds Ratios* | *p* | *Odds Ratios* | *p* |
| Coffee consumption 3-4 cups/day | | 0.78 (0.50 – 1.20) | 0.267 | 1.05 (0.64 – 1.69) | 0.843 | 1.11 (0.65 – 1.85) | 0.692 | 1.12 (0.66 – 1.88) | 0.663 | 1.22 (0.47 – 2.99) | 0.669 |
| Coffee consumption >4 cups/day | | 1.03 (0.57 – 1.74) | 0.929 | 1.18 (0.62 – 2.16) | 0.608 | 1.13 (0.55 – 2.20) | 0.735 | 1.33 (0.66 – 2.55) | 0.414 | 1.07 (0.29 – 3.35) | 0.916 |
| Age | |  |  | 1.07 (1.04 – 1.11) | **<0.001** | 1.08 (1.05 – 1.12) | **<0.001** | 1.08 (1.05 – 1.12) | **<0.001** | 1.10 (1.04 – 1.17) | **0.002** |
| Female | |  |  | 0.61 (0.40 – 0.93) | **0.022** | 0.64 (0.40 – 1.00) | 0.054 | 0.69 (0.43 – 1.08) | 0.106 | 0.82 (0.36 – 1.82) | 0.620 |
| Arterial hypertension | |  |  | 2.59 (1.34 – 5.52) | **0.008** | 3.14 (1.48 – 7.75) | **0.006** | 3.08 (1.45 – 7.63) | **0.007** | 2.98 (0.79 – 19.45) | 0.160 |
| Current smoking | |  |  | 1.25 (0.74 – 2.04) | 0.393 | 1.22 (0.68 – 2.10) | 0.492 | 1.15 (0.63 – 2.00) | 0.638 | 0.59 (0.16 – 1.78) | 0.390 |
| BMI | |  |  | 1.14 (1.10 – 1.18) | **<0.001** | 1.12 (1.07 – 1.17) | **<0.001** | 1.13 (1.08 – 1.17) | **<0.001** | 1.15 (1.07 – 1.23) | **<0.001** |
| Additives | **Milk** |  |  |  |  | 1.00 (0.61 – 1.66) | 0.993 |  |  |  |  |
|  | **Milk + Sugar** |  |  |  |  | 0.73 (0.24 – 1.88) | 0.549 |  |  |  |  |
|  | **Milk + Sweetener** |  |  |  |  | 2.66 (1.05 – 6.29) | **0.030** |  |  |  |  |
|  | **Sweetener** |  |  |  |  | 2.51 (0.72 – 7.64) | 0.121 |  |  |  |  |
|  | **Binary** |  |  |  |  |  |  | 1.08 (0.68 – 1.75) | 0.752 |  |  |

**Supplementary Table 151. Multivariable logistic regression analysis for the association of mild/moderate/high coffee consumption and diabetes (excluding black and green tea consumption).** Not daily coffee consumption (<1 cups/d) served as the reference group. Adjustment was performed for: A) unadjusted; B) main cardiovascular risk factors; C) additional adjustment for additives (separately), D) additional adjustment for additives (binary), E) same adjustment as for group A, but only for subjects who consumed coffee without any additives. Abbreviations as in Table 1

|  | | A)Obesity | | B)Obesity | | C)Obesity | | D)Obesity | | E)Obesity | |
| --- | --- | --- | --- | --- | --- | --- | --- | --- | --- | --- | --- |
|  | | *Odds Ratios* | *p* | *Odds Ratios* | *p* | *Odds Ratios* | *p* | *Odds Ratios* | *p* | *Odds Ratios* | *p* |
| Coffee consumption 3-4 cups/day | | 0.80 (0.60 – 1.07) | 0.141 | 0.84 (0.60 – 1.17) | 0.313 | 0.85 (0.59 – 1.20) | 0.348 | 0.85 (0.59 – 1.20) | 0.360 | 0.70 (0.35 – 1.34) | 0.286 |
| Coffee consumption >4 cups/day | | 0.93 (0.63 – 1.37) | 0.733 | 1.11 (0.71 – 1.71) | 0.646 | 1.04 (0.64 – 1.67) | 0.861 | 1.05 (0.64 – 1.69) | 0.838 | 1.10 (0.48 – 2.42) | 0.815 |
| Age | |  |  | 0.98 (0.96 – 1.00) | **0.024** | 0.98 (0.96 – 1.00) | **0.031** | 0.98 (0.96 – 1.00) | 0.068 | 0.98 (0.95 – 1.02) | 0.396 |
| Female | |  |  | 1.15 (0.86 – 1.52) | 0.349 | 1.13 (0.84 – 1.53) | 0.425 | 1.13 (0.83 – 1.54) | 0.423 | 1.11 (0.63 – 1.94) | 0.726 |
| Diabetes mellitus | |  |  | 3.63 (2.41 – 5.47) | **<0.001** | 3.53 (2.27 – 5.50) | **<0.001** | 3.59 (2.30 – 5.61) | **<0.001** | 7.30 (3.25 – 17.26) | **<0.001** |
| Arterial hypertension | |  |  | 3.50 (2.42 – 5.17) | **<0.001** | 3.51 (2.35 – 5.36) | **<0.001** | 3.58 (2.39 – 5.51) | **<0.001** | 4.96 (2.28 – 12.13) | **<0.001** |
| Current smoking | |  |  | 1.00 (0.71 – 1.40) | 0.998 | 0.98 (0.67 – 1.41) | 0.901 | 1.00 (0.68 – 1.45) | 0.994 | 0.92 (0.44 – 1.83) | 0.816 |
| Additives | **Milk** |  |  |  |  | 0.89 (0.63 – 1.24) | 0.480 |  |  |  |  |
|  | **Milk + Sugar** |  |  |  |  | 0.89 (0.48 – 1.59) | 0.698 |  |  |  |  |
|  | **Milk + Sweetener** |  |  |  |  | 0.95 (0.43 – 1.98) | 0.896 |  |  |  |  |
|  | **Sugar** |  |  |  |  | 0.70 (0.15 – 2.34) | 0.602 |  |  |  |  |
|  | **Sweetener** |  |  |  |  | 2.55 (0.98 – 6.53) | 0.051 |  |  |  |  |
|  | **Binary** |  |  |  |  |  |  | 0.89 (0.65 – 1.23) | 0.485 |  |  |

**Supplementary Table 152. Multivariable logistic regression analysis for the association of mild/moderate/high coffee consumption and obesity (excluding black and green tea consumption).** Not daily coffee consumption (<1 cups/d) served as the reference group. Adjustment was performed for: A) unadjusted; B) main cardiovascular risk factors; C) additional adjustment for additives (separately), D) additional adjustment for additives (binary), E) same adjustment as for group A, but only for subjects who consumed coffee without any additives. Abbreviations as in Table 1

|  | | A)CAD | | B)CAD | | C)CAD | | D)CAD | | E)CAD | |
| --- | --- | --- | --- | --- | --- | --- | --- | --- | --- | --- | --- |
|  | | *Odds Ratios* | *p* | *Odds Ratios* | *p* | *Odds Ratios* | *p* | *Odds Ratios* | *p* | *Odds Ratios* | *p* |
| Coffee consumption 3-4 cups/day | | 0.49 (0.29 – 0.78) | **0.004** | 0.59 (0.33 – 1.02) | 0.064 | 0.61 (0.33 – 1.07) | 0.091 | 0.62 (0.34 – 1.09) | 0.106 | 0.56 (0.17 – 1.64) | 0.309 |
| Coffee consumption >4 cups/day | | 0.95 (0.53 – 1.60) | 0.842 | 1.17 (0.59 – 2.23) | 0.635 | 0.98 (0.46 – 1.98) | 0.950 | 1.05 (0.49 – 2.11) | 0.900 | 1.08 (0.30 – 3.57) | 0.902 |
| Age | |  |  | 1.06 (1.03 – 1.09) | **<0.001** | 1.07 (1.04 – 1.10) | **<0.001** | 1.07 (1.04 – 1.10) | **<0.001** | 1.04 (0.98 – 1.11) | 0.195 |
| Female | |  |  | 0.16 (0.09 – 0.28) | **<0.001** | 0.18 (0.10 – 0.32) | **<0.001** | 0.20 (0.11 – 0.34) | **<0.001** | 0.11 (0.02 – 0.36) | **0.001** |
| Diabetes mellitus | |  |  | 1.76 (0.98 – 3.12) | 0.053 | 1.45 (0.75 – 2.72) | 0.256 | 1.49 (0.78 – 2.79) | 0.214 | 5.61 (1.67 – 20.18) | **0.006** |
| Arterial hypertension | |  |  | 4.65 (2.17 – 11.54) | **<0.001** | 4.38 (1.94 – 11.76) | **0.001** | 4.46 (1.99 – 11.96) | **0.001** | 8.92 (1.57 – 169.77) | **0.043** |
| Current smoking | |  |  | 0.86 (0.47 – 1.51) | 0.606 | 0.93 (0.48 – 1.72) | 0.822 | 0.97 (0.51 – 1.77) | 0.915 | 2.02 (0.67 – 5.84) | 0.197 |
| BMI | |  |  | 1.00 (0.95 – 1.05) | 0.923 | 1.00 (0.95 – 1.06) | 0.898 | 1.01 (0.95 – 1.06) | 0.803 | 0.93 (0.82 – 1.03) | 0.182 |
| Additives | **Milk** |  |  |  |  | 1.14 (0.67 – 1.95) | 0.639 |  |  |  |  |
|  | **Milk + Sugar** |  |  |  |  | 1.36 (0.51 – 3.32) | 0.514 |  |  |  |  |
|  | **Milk + Sweetener** |  |  |  |  | 0.89 (0.19 – 2.98) | 0.862 |  |  |  |  |
|  | **Sweetener** |  |  |  |  | 4.61 (1.18 – 16.78) | **0.022** |  |  |  |  |
|  | **Binary** |  |  |  |  |  |  | 1.21 (0.73 – 2.03) | 0.469 |  |  |

**Supplementary Table 152. Multivariable logistic regression analysis for the association of mild/moderate/high coffee consumption and CAD (excluding black and green tea consumption).** Not daily coffee consumption (<1 cups/d) served as the reference group. Adjustment was performed for: A) unadjusted; B) main cardiovascular risk factors; C) additional adjustment for additives (separately), D) additional adjustment for additives (binary), E) same adjustment as for group A, but only for subjects who consumed coffee without any additives. Abbreviations as in Table 1

|  | | A)PAD | | B)PAD | | C)PAD | | D)PAD | | E)PAD | |
| --- | --- | --- | --- | --- | --- | --- | --- | --- | --- | --- | --- |
|  | | *Odds Ratios* | *p* | *Odds Ratios* | *p* | *Odds Ratios* | *p* | *Odds Ratios* | *p* | *Odds Ratios* | *p* |
| Coffee consumption 3-4 cups/day | | 0.82 (0.54 – 1.23) | 0.352 | 1.05 (0.66 – 1.64) | 0.834 | 0.99 (0.60 – 1.60) | 0.960 | 1.01 (0.62 – 1.61) | 0.983 | 1.11 (0.43 – 2.76) | 0.824 |
| Coffee consumption > 4 cups/day | | 0.73 (0.40 – 1.27) | 0.288 | 0.89 (0.46 – 1.66) | 0.722 | 0.77 (0.37 – 1.54) | 0.475 | 0.73 (0.35 – 1.44) | 0.376 | 1.62 (0.55 – 4.66) | 0.370 |
| Age | |  |  | 1.04 (1.02 – 1.07) | **0.001** | 1.05 (1.02 – 1.08) | **0.001** | 1.05 (1.02 – 1.08) | **0.001** | 1.07 (1.02 – 1.14) | **0.014** |
| Female | |  |  | 1.43 (0.96 – 2.13) | 0.075 | 1.50 (0.98 – 2.30) | 0.064 | 1.47 (0.97 – 2.25) | 0.071 | 1.46 (0.65 – 3.26) | 0.357 |
| Diabetes mellitus | |  |  | 2.20 (1.20 – 4.03) | **0.011** | 2.12 (1.10 – 4.05) | **0.023** | 2.13 (1.11 – 4.03) | **0.021** | 1.98 (0.60 – 6.31) | 0.249 |
| Arterial hypertension | |  |  | 1.39 (0.86 – 2.28) | 0.187 | 1.24 (0.73 – 2.13) | 0.424 | 1.23 (0.74 – 2.10) | 0.429 | 0.84 (0.31 – 2.32) | 0.729 |
| Current smoking | |  |  | 1.55 (0.96 – 2.47) | 0.067 | 1.83 (1.09 – 3.04) | **0.021** | 1.78 (1.07 – 2.93) | **0.024** | 2.34 (0.89 – 6.07) | 0.081 |
| BMI | |  |  | 1.03 (0.99 – 1.08) | 0.131 | 1.04 (0.99 – 1.09) | 0.092 | 1.04 (0.99 – 1.09) | 0.095 | 1.09 (0.99 – 1.19) | 0.066 |
| Additives | **Milk** |  |  |  |  | 1.08 (0.68 – 1.74) | 0.734 |  |  |  |  |
|  | **Milk + Sugar** |  |  |  |  | 0.62 (0.24 – 1.46) | 0.302 |  |  |  |  |
|  | **Milk + Sweetener** |  |  |  |  | 1.00 (0.25 – 3.24) | 0.997 |  |  |  |  |
|  | **Sweetener** |  |  |  |  | 0.88 (0.12 – 4.45) | 0.883 |  |  |  |  |
|  | **Binary** |  |  |  |  |  |  | 1.02 (0.65 – 1.61) | 0.939 |  |  |

**Supplementary Table 154. Multivariable logistic regression analysis for the association of mild/moderate/high coffee consumption and PAD (excluding black and green tea consumption).** Not daily coffee consumption (<1 cups/d) served as the reference group. Adjustment was performed for: A) unadjusted; B) main cardiovascular risk factors; C) additional adjustment for additives (separately), D) additional adjustment for additives (binary), E) same adjustment as for group A, but only for subjects who consumed coffee without any additives. Abbreviations as in Table 1

|  | | A)Heart failure | | B)Heart failure | | C)Heart failure | | D)Heart failure | | E)Heart failure | |
| --- | --- | --- | --- | --- | --- | --- | --- | --- | --- | --- | --- |
|  | | *Odds Ratios* | *p* | *Odds Ratios* | *p* | *Odds Ratios* | *p* | *Odds Ratios* | *p* | *Odds Ratios* | *p* |
| Coffee consumption 3-4 cups/day | | 0.25 (0.09 – 0.59) | **0.004** | 0.25 (0.07 – 0.66) | **0.012** | 0.20 (0.05 – 0.59) | **0.010** | 0.20 (0.05 – 0.59) | **0.010** | 0.23 (0.01 – 1.43) | 0.184 |
| Coffee consumption >4 cups/day | | 1.39 (0.68 – 2.66) | 0.336 | 1.36 (0.58 – 2.99) | 0.458 | 1.11 (0.40 – 2.76) | 0.837 | 0.77 (0.26 – 2.00) | 0.615 | 1.32 (0.21 – 6.98) | 0.755 |
| Age | |  |  | 1.07 (1.03 – 1.12) | **0.003** | 1.08 (1.03 – 1.14) | **0.004** | 1.08 (1.03 – 1.14) | **0.004** | 1.13 (1.02 – 1.27) | **0.030** |
| Female | |  |  | 0.44 (0.22 – 0.85) | **0.017** | 0.61 (0.29 – 1.23) | 0.176 | 0.57 (0.27 – 1.16) | 0.128 | 0.52 (0.12 – 1.91) | 0.340 |
| Diabetes mellitus | |  |  | 2.55 (1.17 – 5.27) | **0.014** | 3.15 (1.34 – 7.05) | **0.006** | 3.08 (1.32 – 6.82) | **0.007** | 6.85 (1.40 – 33.91) | **0.015** |
| Arterial hypertension | |  |  | 2.75 (1.02 – 9.63) | 0.071 | 4.70 (1.29 – 30.36) | **0.043** | 4.40 (1.23 – 28.21) | 0.051 |  |  |
| Current smoking | |  |  | 1.74 (0.78 – 3.67) | 0.158 | 1.94 (0.77 – 4.68) | 0.145 | 2.57 (1.04 – 6.13) | **0.036** | 8.61 (1.62 – 54.08) | **0.014** |
| BMI | |  |  | 1.12 (1.05 – 1.19) | **0.001** | 1.12 (1.04 – 1.20) | **0.002** | 1.11 (1.03 – 1.19) | **0.005** | 1.10 (0.96 – 1.24) | 0.153 |
| Additives | **Milk** |  |  |  |  | 1.30 (0.61 – 2.93) | 0.504 |  |  |  |  |
|  | **Milk + Sugar** |  |  |  |  | 2.62 (0.74 – 8.35) | 0.112 |  |  |  |  |
|  | **Milk + Sweetener** |  |  |  |  | 0.42 (0.02 – 2.53) | 0.433 |  |  |  |  |
|  | **Sweetener** |  |  |  |  | 1.95 (0.09 – 16.09) | 0.589 |  |  |  |  |
|  | **Binary** |  |  |  |  |  |  | 1.26 (0.61 – 2.75) | 0.540 |  |  |

**Supplementary Table 155. Multivariable logistic regression analysis for the association of mild/moderate/high coffee consumption and heart failure (excluding black and green tea consumption).** Not daily coffee consumption (<1 cups/d) served as the reference group. Adjustment was performed for: A) unadjusted; B) main cardiovascular risk factors; C) additional adjustment for additives (separately), D) additional adjustment for additives (binary), E) same adjustment as for group A, but only for subjects who consumed coffee without any additives. Abbreviations as in Table 1

|  | | A)HF(m)rEF | | B)HF(m)rEF | | C)HF(m)rEF | | D)HF(m)rEF | | E)HF(m)rEF | |
| --- | --- | --- | --- | --- | --- | --- | --- | --- | --- | --- | --- |
|  | | *Odds Ratios* | *p* | *Odds Ratios* | *p* | *Odds Ratios* | *p* | *Odds Ratios* | *p* | *Odds Ratios* | *p* |
| Coffee consumption 3-4 cups/day | | 0.35 (0.10 – 0.93) | 0.056 | 0.30 (0.07 – 0.90) | 0.057 | 0.36 (0.08 – 1.14) | 0.119 | 0.36 (0.08 – 1.14) | 0.120 | 0.79 (0.04 – 7.21) | 0.847 |
| Coffee consumption >4 cups/day | | 1.56 (0.64 – 3.43) | 0.292 | 1.48 (0.54 – 3.68) | 0.416 | 1.33 (0.40 – 3.86) | 0.615 | 0.98 (0.26 – 3.01) | 0.971 | 7.90 (0.95 – 77.31) | 0.056 |
| Age | |  |  | 1.04 (0.99 – 1.10) | 0.111 | 1.06 (1.00 – 1.13) | 0.065 | 1.06 (1.00 – 1.13) | 0.064 | 1.16 (1.02 – 1.36) | **0.037** |
| Female | |  |  | 0.15 (0.04 – 0.38) | **<0.001** | 0.21 (0.06 – 0.57) | **0.005** | 0.21 (0.06 – 0.57) | **0.005** | 0.18 (0.01 – 1.19) | 0.130 |
| Diabetes mellitus | |  |  | 1.46 (0.50 – 3.72) | 0.450 | 1.71 (0.52 – 4.82) | 0.335 | 1.97 (0.60 – 5.45) | 0.219 | 4.79 (0.55 – 35.59) | 0.121 |
| Arterial hypertension | |  |  | 4.26 (1.16 – 27.54) | 0.059 | 6.71 (1.27 – 124.20) | 0.072 | 6.48 (1.23 – 119.75) | 0.077 |  |  |
| Current smoking | |  |  | 1.24 (0.46 – 3.04) | 0.654 | 1.48 (0.47 – 4.19) | 0.477 | 1.98 (0.64 – 5.63) | 0.212 | 1.85 (0.18 – 17.68) | 0.585 |
| BMI | |  |  | 1.10 (1.01 – 1.19) | **0.025** | 1.07 (0.97 – 1.17) | 0.159 | 1.06 (0.96 – 1.16) | 0.236 | 1.06 (0.86 – 1.25) | 0.535 |
| Additives | **Milk** |  |  |  |  | 1.29 (0.50 – 3.58) | 0.609 |  |  |  |  |
|  | **Milk + Sugar** |  |  |  |  | 2.12 (0.42 – 8.57) | 0.313 |  |  |  |  |
|  | **Milk + Sweetener** |  |  |  |  | 0.94 (0.05 – 6.03) | 0.956 |  |  |  |  |
|  | **Sweetener** |  |  |  |  | 4.22 (0.18 – 38.46) | 0.251 |  |  |  |  |
|  | **Binary** |  |  |  |  |  |  | 1.30 (0.54 – 3.50) | 0.578 |  |  |

**Supplementary Table 156. Multivariable logistic regression analysis for the association of mild/moderate/high coffee consumption and HF(m)rEF (excluding black and green tea consumption).** Not daily coffee consumption (<1 cups/d) served as the reference group. Adjustment was performed for: A) unadjusted; B) main cardiovascular risk factors; C) additional adjustment for additives (separately), D) additional adjustment for additives (binary), E) same adjustment as for group A, but only for subjects who consumed coffee without any additives. Abbreviations as in Table 1

|  | | A)Total cholesterol | | B)Total cholesterol | | C)Total cholesterol | | D)Total cholesterol | | E)Total cholesterol | | F)Total cholesterol | | G)Total cholesterol | |
| --- | --- | --- | --- | --- | --- | --- | --- | --- | --- | --- | --- | --- | --- | --- | --- |
|  | | *Estimates* | *p* | *Estimates* | *p* | *Estimates* | *p* | *Estimates* | *p* | *Estimates* | *p* | *Estimates* | *p* | *Estimates* | *p* |
| Coffee consumption 3-4 cups/day | | 0.37 (-1.66 – 2.39) | 0.721 | 1.66 (-0.45 – 3.78) | 0.123 | 1.19 (-1.04 – 3.42) | 0.295 | 1.09 (-1.14 – 3.33) | 0.337 | 1.26 (-0.76 – 3.29) | 0.220 | 1.58 (-2.58 – 5.73) | 0.457 | 1.59 (-0.54 – 3.72) | 0.143 |
| Coffee consumption >4cups/day | | 0.20 (-2.65 – 3.06) | 0.889 | 4.80 (1.78 – 7.83) | **0.002** | 4.91 (1.70 – 8.12) | **0.003** | 4.78 (1.56 – 8.00) | **0.004** | 4.30 (1.41 – 7.18) | **0.003** | 2.06 (-3.42 – 7.55) | 0.461 | 4.70 (1.66 – 7.74) | **0.002** |
| Age | |  |  | 0.12 (-0.00 – 0.23) | 0.050 | 0.10 (-0.03 – 0.22) | 0.122 | 0.10 (-0.03 – 0.22) | 0.129 | 0.42 (0.30 – 0.53) | **<0.001** | 0.09 (-0.16 – 0.33) | 0.490 | 0.13 (0.02 – 0.25) | **0.025** |
| Female | |  |  | 20.63 (18.81 – 22.44) | **<0.001** | 21.42 (19.48 – 23.36) | **<0.001** | 21.37 (19.43 – 23.30) | **<0.001** | 18.81 (17.07 – 20.54) | **<0.001** | 24.78 (21.10 – 28.46) | **<0.001** | 20.59 (18.76 – 22.42) | **<0.001** |
| Diabetes mellitus | |  |  | -20.53 (-23.94 – -17.12) | **<0.001** | -20.62 (-24.26 – -16.98) | **<0.001** | -20.87 (-24.52 – -17.22) | **<0.001** | -14.12 (-17.44 – -10.81) | **<0.001** | -23.41 (-30.16 – -16.66) | **<0.001** | -21.05 (-24.50 – -17.61) | **<0.001** |
| Arterial hypertension | |  |  | 1.93 (-0.13 – 4.00) | 0.067 | 2.18 (-0.00 – 4.37) | 0.050 | 2.32 (0.12 – 4.51) | **0.038** | 4.73 (2.75 – 6.71) | **<0.001** | 3.77 (-0.42 – 7.95) | 0.078 | 1.88 (-0.19 – 3.96) | 0.076 |
| Current smoking | |  |  | -1.48 (-3.82 – 0.85) | 0.212 | -2.57 (-5.06 – -0.08) | **0.043** | -2.45 (-4.95 – 0.04) | 0.054 | -0.43 (-2.66 – 1.79) | 0.704 | -3.47 (-8.16 – 1.23) | 0.148 | -1.57 (-3.91 – 0.78) | 0.190 |
| BMI | |  |  | -0.26 (-0.46 – -0.05) | **0.015** | -0.28 (-0.50 – -0.06) | **0.013** | -0.29 (-0.51 – -0.07) | **0.010** | -0.09 (-0.29 – 0.10) | 0.356 | -0.35 (-0.77 – 0.06) | 0.094 | -0.26 (-0.47 – -0.05) | **0.015** |
| Additives | **Milk** |  |  |  |  | -2.19 (-4.38 – -0.00) | **0.049** |  |  |  |  |  |  |  |  |
|  | **Milk + Sugar** |  |  |  |  | -1.33 (-4.94 – 2.29) | 0.471 |  |  |  |  |  |  |  |  |
|  | **Milk + Sweetener** |  |  |  |  | -5.88 (-11.35 – -0.42) | **0.035** |  |  |  |  |  |  |  |  |
|  | **Sugar** |  |  |  |  | -0.57 (-8.26 – 7.12) | 0.884 |  |  |  |  |  |  |  |  |
|  | **Sweetener** |  |  |  |  | 4.24 (-4.59 – 13.07) | 0.347 |  |  |  |  |  |  |  |  |
|  | **Binary** |  |  |  |  |  |  | -2.18 (-4.29 – -0.07) | **0.043** |  |  |  |  |  |  |
| Lipid lowering drugs | |  |  |  |  |  |  |  |  | -33.49 (-35.88 – -31.09) | **<0.001** |  |  |  |  |
| Diet | **Vegan** |  |  |  |  |  |  |  |  |  |  |  |  | -11.83 (-30.17 – 6.51) | 0.206 |
|  | **Vegetarian** |  |  |  |  |  |  |  |  |  |  |  |  | -3.04 (-7.84 – 1.75) | 0.213 |
[truncated: 73,003 more chars]
